# Novel amphiphilic pyridinium ionic liquids-supported Schiff bases: ultrasound assisted synthesis, molecular docking and anticancer evaluation

**DOI:** 10.1186/s13065-018-0489-z

**Published:** 2018-11-22

**Authors:** Fawzia Faleh Al-Blewi, Nadjet Rezki, Salsabeel Abdullah Al-Sodies, Sanaa K. Bardaweel, Dima A. Sabbah, Mouslim Messali, Mohamed Reda Aouad

**Affiliations:** 1 Department of Chemistry, Faculty of Science, Taibah University, Al-Madinah Al-Munawarah, Medina, 30002 Saudi Arabia; 2 Department of Chemistry, Faculty of Sciences, University of Sciences and Technology Mohamed Boudiaf, Laboratoire de Chimie et Electrochimie des Complexes Metalliques (LCECM) USTO-MB, P.O. Box 1505, El M‘nouar, 31000 Oran, Algeria; 3 Department of Pharmaceutical Sciences, Faculty of Pharmacy, University of Jordan, Amman, 11942 Jordan; 4 Faculty of Pharmacy, Al-Zaytoonah University, Amman, 11733 Jordan

**Keywords:** Cationic, Amphiphilic, Pyridinium, Hydrazones, Ultrasound, Anticancer, QPLD docking

## Abstract

**Background:**

Pyridinium Schiff bases and ionic liquids have attracted increasing interest in medicinal chemistry.

**Results:**

A library of 32 cationic fluorinated pyridinium hydrazone-based amphiphiles tethering fluorinated counteranions was synthesized by alkylation of 4-fluoropyridine hydrazone with various long alkyl iodide exploiting lead quaternization and metathesis strategies. All compounds were assessed for their anticancer inhibition activity towards different cancer cell lines and the results revealed that increasing the length of the hydrophobic chain of the synthesized analogues appears to significantly enhance their anticancer activities. Substantial increase in caspase-3 activity was demonstrated upon treatment with the most potent compounds, namely **8**, **28**, **29** and **32** suggesting an apoptotic cellular death pathway.

**Conclusions:**

Quantum-polarized ligand docking studies against phosphoinositide 3-kinase α displayed that compounds **2**–**6** bind to the kinase site and form H-bond with S774, K802, H917 and D933. 
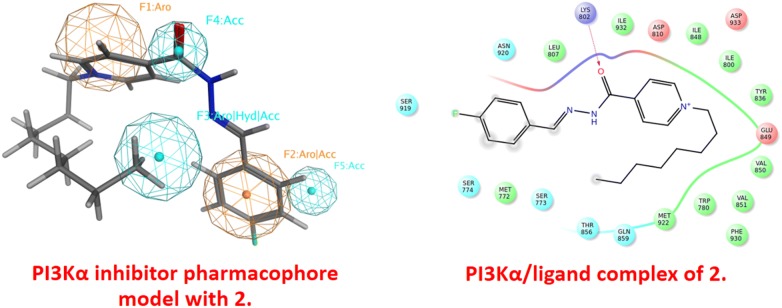

**Electronic supplementary material:**

The online version of this article (10.1186/s13065-018-0489-z) contains supplementary material, which is available to authorized users.

## Introduction

Schiff bases have been widely investigated due to a broad spectrum of relevant properties in biological and pharmaceutical areas [1]. In addition, a number of molecules having azomethine Schiff base skeleton are the clinically approved drugs [2]. Meanwhile, carbohydrazide hydrazone and their derivatives an interesting class of Schiff bases, represented reliable and highly efficient pharmacophores in drug discovery and played a vital role in medical chemistry due to their potency to exhibit significant antimicrobial [3], anticancer [4, 5], anti-HIV [6], and anticandidal [7] activities. Azomethine hydrazone linkages (RCONHN=CR^1^R^2^) are one of the versatile and attractive functional groups in organic synthesis [8, 9]. Their ability to react with electrophilic and nucleophilic reagents make them valuable candidates for the construction of diverse heterocyclic scaffolds [10]. Some pyridine hydrazones have been reported to possess fascinating chemotherapeutic properties [11, 12]. On the other hand, biological and toxicity of pyridinium salts have been well documented due to their increasing applications. More specifically, pyridinium salts carrying long alkyl chains were found to be outstanding bioactive agents as antimicrobial [13], anticancer [14] and biodegradable [15] agents. Recently, we have reported a green ultrasound synthesis of novel fluorinated pyridinium hydrazones using a series of alkyl halides ranging from C2 to C7 [16]. The biological screening results revealed that the activity increased with increasing the length of the alkyl side chains, especially for hydrazones tethering fluorinated counteranions (PF_6_^−^, BF_4_^−^ and CF_3_COO^−^). Encouraged by these findings and in continuation of our efforts in designing highly active heterocyclic hydrazones [17–19], we aim to introduce a lipophilic long alkyl chain to a hydrazone skeleton to develop a new class of bioactive molecules. In the present work, a series of novel cationic fluorinated pyridinium hydrazone-based amphiphiles tethering different fluorinated counteranions were designed, synthesized and screened for their anticancer activities against four different cell lines. Additionally, their activities were further characterized via investigating the Caspase-3 signaling pathway, a hallmark of apoptosis that is commonly studied to understand the mechanism of cellular death.

Molecular quantum-polarized ligand docking (QPLD) studies were carried out employing MAESTRO [20] software against the kinase domain of phosphoinositide 3-kinase α (PI3Kα) [21] to identify their structural-basis of binding and ligand/receptor complex formation.

## Results and discussion

### Synthesis

**Scheme 1:**
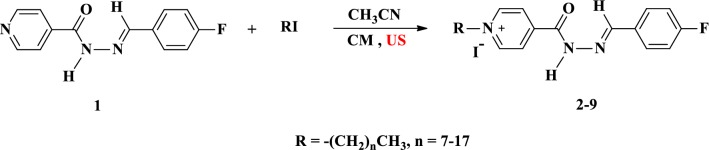
Synthesis of pyridinium hydrazones **2**–**9** carrying iodide counter anion

The methodology for affecting the sequence of reactions utilized ultrasound irradiations which have been widely used by our team as an alternative source of energy. Starting from fluorinated pyridine hydrazone **1**, the quaternization of pyridine ring through its conventional alkylation with various long alkyl iodide with chain ranging from C_8_ to C_18_, in boiling acetonitrile as well as under ultrasound irradiation and gave the desired cationic fluorinated pyridinium hydrazones **2**–**9** tethering lipophilic side chain and iodide counteranion in good yields (Scheme 1). Short reactions time were required (10–12 h) when the ultrasound irradiations were used as an alternative energy source (Table 1).

**Table 1:** Times and yields of halogenated pyridinium hydrazones **2**–**9** under conventional and ultrasound

Compound no	R	Conventional method CM		Ultrasound method US	
		Time (h)	Yield (%)	Time (h)	Yield (%)
**2**	C_8_H_17_	72	84	10	92
**3**	C_9_H_19_	72	90	10	96
**4**	C_10_H_21_	72	88	12	92
**5**	C_11_H_23_	72	92	12	98
**6**	C_12_H_25_	72	88	12	92
**7**	C_14_H_29_	72	85	12	92
**8**	C_16_H_33_	72	89	12	94
**9**	C_18_H_37_	72	83	12	96

The structure of newly designed pyridinium cationic surfactants **2**–**9** have been elucidated based on their spectral data (IR, NMR, Mass). Their IR spectra revealed the appearance of new characteristic bands at 2870–2969 cm^−1^ attributed to the aliphatic C-H stretching which confirmed the presence of alkyl side chain in this structure. The ^1^H NMR analysis showed one methyl and methylene groups resonating as two multiplets between δ_H_ 0.74–0.87 ppm and 1.16–1.32 ppm, respectively. The spectra also showed the presence of characteristic triplet and/or doublet of doublet ranging between δ_H_ 4.68–4.78 ppm assigned to NC**H**_**2**_ protons.

In addition, the imine proton (H–C=N) resonated as two set of singlets at δ_H_ 8.15–8.50 ppm with a 1:3 ratio. The presence of such pairing of signals confirmed that these compounds exist as *E*/cis and *E*/trans diastereomers.

The ^13^C NMR data also confirmed the appearance of *E*/cis and *E*/trans diastereomers through the presence of two peaks at δ_H_ 58.60 and 62.74 ppm for N**C**H_2_. In the downfield region between δ_C_ 156.38–165.76 ppm, the carbonyl and the imine carbons of the hydrazone linkage resonated as two sets of signals.

In their ^19^F NMR spectra, the aromatic fluorine atom appeared as two mutiplet signals between δ_H_ (− 107.98 to − 109.89 ppm) and (− 107.72 to − 109.37 ppm).

**Scheme 2:**
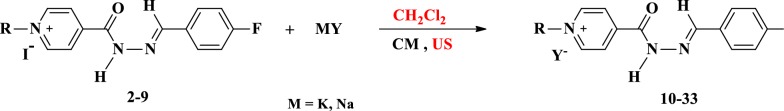
Synthesis of pyridinium hydrazones **10**–**33** carrying fluorinated counteranions

Treatment of the halogenated pyridinium hydrazones **2**–**9** with fluorinated metal salts (KPF_6_, NaBF_4_ or NaOOCCF_3_) afforded the targeted cationic amphiphilic fluorinated pyridinium hydrazones **10**–**33** carrying variant fluorinated counteranions (Scheme 2). The reaction involved the anion exchange and was carried out in short time (6 h) under ultrasound irradiation and gave comparative yields with those obtained using classical heating (16 h) (Table 2).

**Table 2:** Times and yields of pyridinium hydrazones **10**–**33** carrying fluorinated counter anions under conventional and ultrasound

Compound no	R	Y	Conventional method CM		Ultrasound method US	
			Time (h)	Yield (%)	Time (h)	Yield (%)
**10**	C_8_H_17_	PF_6_	16	83	6	90
**11**	C_8_H_17_	BF_4_	16	98	6	98
**12**	C_8_H_17_	COOCF_3_	16	80	6	88
**13**	C_9_H_19_	PF_6_	16	90	6	94
**14**	C_9_H_19_	BF_4_	16	85	6	90
**15**	C_9_H_19_	COOCF_3_	16	87	6	92
**16**	C_10_H_21_	PF_6_	16	98	6	98
**17**	C_10_H_21_	BF_4_	16	88	6	90
**18**	C_10_H_21_	COOCF_3_	16	86	6	92
**19**	C_11_H_23_	PF_6_	16	94	6	98
**20**	C_11_H_23_	BF_4_	16	93	6	94
**21**	C_11_H_23_	COOCF_3_	16	90	6	94
**22**	C_12_H_25_	PF_6_	16	87	6	90
**23**	C_12_H_25_	BF_4_	16	82	6	90
**24**	C_12_H_25_	COOCF_3_	16	88	6	92
**25**	C_14_H_29_	PF_6_	16	95	6	98
**26**	C_14_H_29_	BF_4_	16	93	6	96
**27**	C_14_H_29_	COOCF_3_	16	97	6	98
**28**	C_16_H_33_	PF_6_	16	89	6	92
**29**	C_16_H_33_	BF_4_	16	90	6	94
**30**	C_16_H_33_	COOCF_3_	16	88	6	92
**31**	C_18_H_37_	PF_6_	16	88	6	92
**32**	C_18_H_37_	BF_4_	16	87	6	90
**33**	C_18_H_37_	COOCF_3_	16	84	6	90

Structural differentiation between the metathetical products **10**–**33** and their halogenated precursors **2**–**9** was very difficult on the basis of their ^1^H NMR and ^13^C NMR spectra because they displayed virtually the same characteristic proton and carbon signals.

Consequently, other spectroscopic techniques (^19^F, ^31^P, ^11^B NMR and mass spectroscopy) have been adopted to confirm the presence of fluorinated counteranions (PF_6_^−^, BF_4_^−^ and CF_3_COO^−^) in the structure of the resulted ILs **10**–**33**.

Thus, the presence of PF_6_^−^ in ILs **10**, **13**, **16**, **19**, **22**, **25**, **28** and **31** has been established by their ^31^P and ^19^F NMR analysis. Thus, the resonance of a diagnostic multiplet between δ_P_ − 152.70 and − 135.76 ppm in the ^31^P NMR spectra confirmed the presence of  PF_6_^−^ in their structure.

On the other hand, the ^19^F NMR analysis of the same compounds revealed the appearance of new doublet at δ_F_ − 70.39 and − 69.21 ppm attributed to the six fluorine atoms in PF_6_^−^ anions.

The formation of ionic liquids **11**, **14**, **17**, **20**, **23**, **26**, **29** and **32** carrying BF_4_^−^ in their structures were supported by the ^11^B and ^19^F NMR experiments. Thus, their ^11^B NMR spectra exhibited a multiplet between δ_B_ − 1.30 and − 1.29 ppm confirming the presence of boron atom in its BF_4_^−^ form. Two doublets were recorded at δ_F_ − 149.12 and − 148.12 ppm in their ^19^F NMR spectra.

Structural elucidation of the ionic liquids containing trifluoroacetate (C**F**_**3**_COO^−^) was investigated by the ^19^F NMR analysis which revealed the presence of characteristic singlet ranging from − 73.50 to − 75.30 ppm.

The physical (state of product and melting points) and photochemical (fluorescence and λ_max_ in UV) data of the synthesized pyridinium hydrazones **2**–**33** were investigated and recorded in Table 3.

**Table 3:** Physical and analytical data for the newly synthesized pyridinium hydrazones **2**–**33** 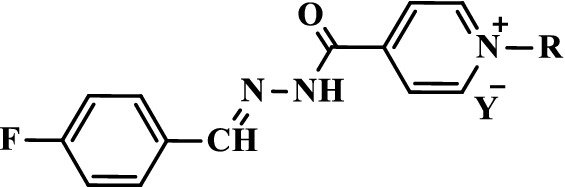

Comp no	R	Y	mp °C	λ_max_ (nm)	Fluorescence
**2**	C_8_H_17_	I	104–105	222, 330, 430	+
**3**	C_9_H_19_	I	91–93	220, 332, 432	+
**4**	C_10_H_21_	I	110–112	220, 332, 430	+
**5**	C_11_H_23_	I	82–83	220, 332, 430	+
**6**	C_12_H_25_	I	72–73	220, 330, 430	+
**7**	C_14_H_29_	I	86–88	220, 332, 430	+
**8**	C_16_H_33_	I	78–80	220, 332, 430	+
**9**	C_18_H_37_	I	98–99	220, 332, 430	+
**10**	C_8_H_17_	PF_6_	Yellow crystals 64–65	220, 330, 430	+
**11**	C_8_H_17_	BF_4_	Yellow crystals 80–82	220, 332, 430	+
**12**	C_8_H_17_	COOCF_3_	Yellow crystals 74–76	220, 332, 430	+
**13**	C_9_H_19_	PF_6_	Yellow crystals 69–70	220, 330, 428	+
**14**	C_9_H_19_	BF_4_	Yellow crystals 88–90	222, 328, 426	+
**15**	C_9_H_19_	COOCF_3_	Yellow crystals 96–98	222, 332, 424	+
**16**	C_10_H_21_	PF_6_	Yellow syrup	220, 330, 428	+
**17**	C_10_H_21_	BF_4_	Colorless syrup	220, 330, 428	+
**18**	C_10_H_21_	COOCF_3_	Yellow syrup	222, 334, 432	+
**19**	C_11_H_23_	PF_6_	Yellow syrup	220, 330, 428	+
**20**	C_11_H_23_	BF_4_	Yellow syrup	220, 330, 426	+
**21**	C_11_H_23_	COOCF_3_	Colorless syrup	222, 332, 430	+
**22**	C_12_H_25_	PF_6_	Yellow syrup	222, 330, 430	+
**23**	C_12_H_25_	BF_4_	Yellow syrup	218, 332, 430	+
**24**	C_12_H_25_	COOCF_3_	Colorless syrup	220, 336, 428	+
**25**	C_14_H_29_	PF_6_	Yellow syrup	220, 332, 428	+
**26**	C_14_H_29_	BF_4_	Yellow syrup	220, 336, 430	+
**27**	C_14_H_29_	COOCF_3_	Colorless syrup	220, 330, 428	+
**28**	C_16_H_33_	PF_6_	Yellow syrup	220, 338, 432	+
**29**	C_16_H_33_	BF_4_	Yellow syrup	218, 332, 428	+
**30**	C_16_H_33_	COOCF_3_	Colorless syrup	220, 334, 430	+
**31**	C_18_H_37_	PF_6_	Yellow syrup	220, 330, 428	+
**32**	C_18_H_37_	BF_4_	Yellow syrup	220, 330, 432	+
**33**	C_18_H_37_	COOCF_3_	Colorless syrup	220, 332, 430	+

### Biological results

Attempting to characterize any potential biological activity associated with the newly synthesized compounds, an in vitro assessment of their antiproliferative activity was conducted on four different human cancerous cell lines; the human breast adenocarcinoma (MCF-7), human breast carcinoma (T47D), human colon epithelial (Caco-2) and human uterine cervical carcinoma (Hela) cell lines. Only compounds shown in Table 4 demonstrated a reasonably high antiproliferative activity against the model cancer cell lines used.

**Table 4:** IC_50_ values (µM) on 4 different cancer cell lines

Code	MCF-7	T47D	Caco-2	Hela
**4**	153 ± 12	145 ± 10	156 ± 9	155 ± 11
**5**	136 ± 7	134 ± 10	139 ± 9	142 ± 6
**6**	134 ± 9	139 ± 7	139 ± 9	129 ± 11
**7**	120 ± 6	123 ± 7	128 ± 7	119 ± 8
**8**	61 ± 5	59 ± 7	67 ± 6	68 ± 5
**9**	20 ± 3	23 ± 4	18 ± 3	25 ± 3
**16**	179 ± 15	172 ± 13	171 ± 19	177 ± 10
**17**	176 ± 12	170 ± 10	168 ± 12	177 ± 11
**19**	137 ± 8	133 ± 11	139 ± 6	141 ± 10
**20**	132 ± 4	139 ± 9	134 ± 5	138 ± 5
**21**	178 ± 10	176 ± 19	171 ± 15	169 ± 17
**22**	129 ± 4	129 ± 8	125 ± 9	124 ± 13
**23**	128 ± 10	120 ± 9	121 ± 14	128 ± 11
**24**	131 ± 10	139 ± 6	145 ± 7	132 ± 12
**25**	134 ± 10	133 ± 9	132 ± 5	131 ± 9
**26**	123 ± 10	127 ± 15	127 ± 12	129 ± 11
**27**	67 ± 4	61 ± 2	67 ± 4	68 ± 6
**28**	39 ± 5	40 ± 6	32 ± 4	36 ± 4
**29**	21 ± 3	20 ± 4	19 ± 1	26 ± 2
**30**	45 ± 6	46 ± 4	41 ± 3	48 ± 6
**31**	71 ± 3	77 ± 8	74 ± 5	79 ± 2
**32**	39 ± 7	34 ± 4	38 ± 7	35 ± 7
**33**	41 ± 5	48 ± 7	44 ± 3	49 ± 5

Values are expressed as mean ± SD of three experiments

Remarkably, increasing the length of the hydrophobic chain appears to significantly potentiate the antiproliferative activities associated with the examined analogues, probably owing to their better penetration into the cellular compartment.

**Fig. 1:**
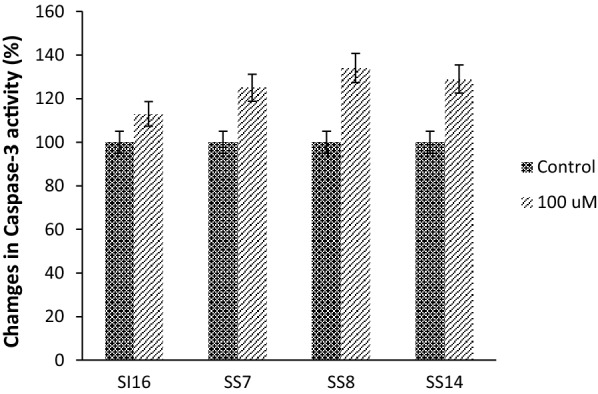
Caspase3 activity in MCF7 cells after 48 h. The results are the means of two independent experiments. P < 0.05 was considered significant

To determine the apoptotic effects of cytotoxic compounds and to evaluate modulators of the cell death cascade, activation of the caspase-3 pathway, a hallmark of apoptosis, can be employed in cellular assays. According to the demonstrated results (Fig. 1) and in response to 48 h treatment with the most potent compounds, significant increase in caspase-3 activity is yielded suggesting that the antiproliferative activities of the examined compounds are most likely mediated by an apoptotic cellular death pathway.

Further exploration of possible pathways by which these compounds exert their antiproliferative activities should shed light onto prospective molecular targets with which the compounds may interrelate.

### Docking results

In order to explain the anticancer activity of the verified compounds **2**–**9** against the examined cancer cell lines, we recruited the crystal structure of PI3Kα (PDB ID: 2RD0) [21] to determine the binding interaction of these compounds in PI3Kα kinase domain. Noting that these cell lines express phosphatidylinositol 3-kinase (PI3Kα) particularly MCF-7 [22–26], T47D [22, 25–32], Caco-2 [33–35] and Hela [36–38].

The binding site of 2RD0 is composed of M772, K776, W780, I800, K802, L807, D810, Y836, I848, E849, V850, V851, S854, T856, Q859, M922, F930, I932 and D933 [39]. The hydrophobic and polar residues are located in the binding domain. It’s worth noting that the exposed hydrophilic and hydrophobic surface areas of the co-crystallized ligand agree with the surrounding residues. The polar residues furnish hydrogen-bonding, ion–dipole and dipole–dipole interactions.

Furthermore, the polar acidic or basic residues mediate an ionic (electrostatic) bonding. The nonpolar motif such as the aromatic and/or hydrophobic residue affords π-stacking aromatic and hydrophobic (van der Waals) interaction, respectively.

**Fig. 2:**
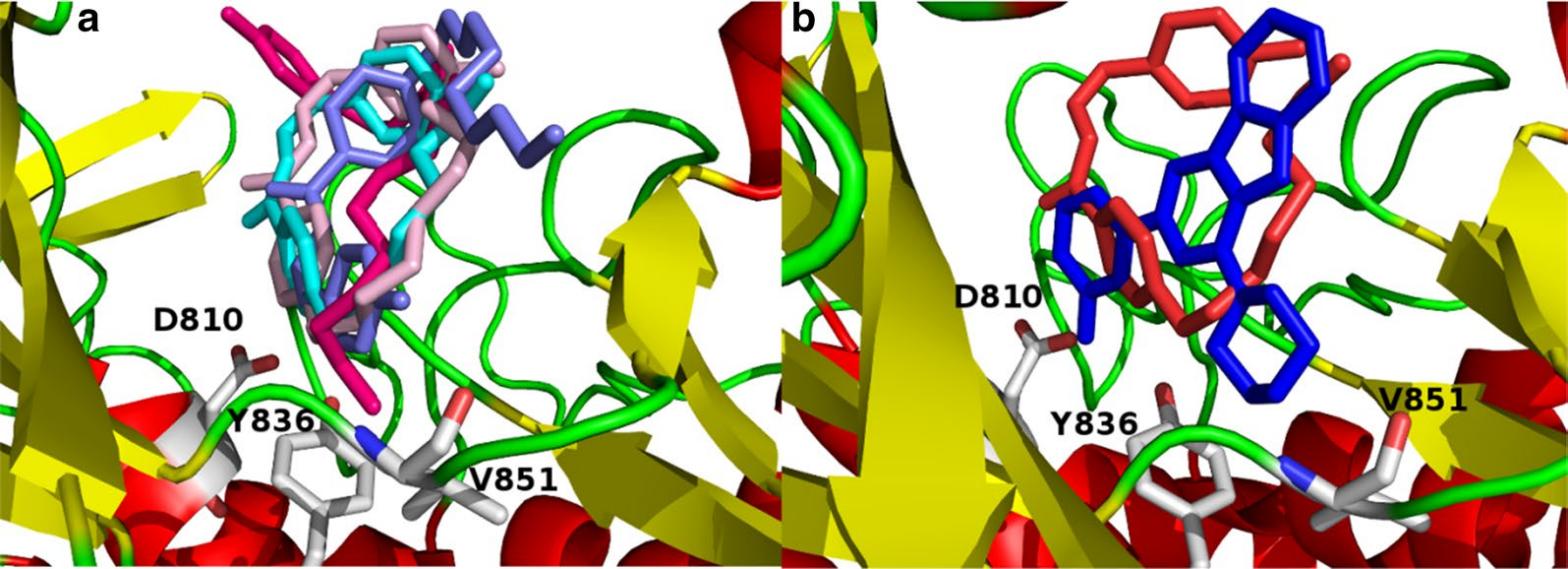
The catalytic kinase domain of (**a**) 2RD0 harbors the QPLD docked poses of some of the verified molecules **2**–**9** and (**b**) superposition of the QPLD docked pose **2** and the co-crystallized ligand represented in red and blue colors, respectively

In order to identify the structural-basis of PI3Kα/ligand interaction of the verified compounds in the catalytic kinase domain of PI3Kα, we employed QPLD docking [40, 41] against the kinase cleft of 2RD0. Our QPLD docking data show that some of the synthesized molecules **2**–**9** bind to the kinase domain of PI3Kα (Fig. 2, part a). Indeed, compounds having side chain alkyl group more than twelve carbon atoms **7**–**9** extend beyond the binding cleft boundary.

Moreover, a part of the docked pose of **2** superposes that of the co-crystalized ligand (Fig. 2, part b).

**Fig. 3:**
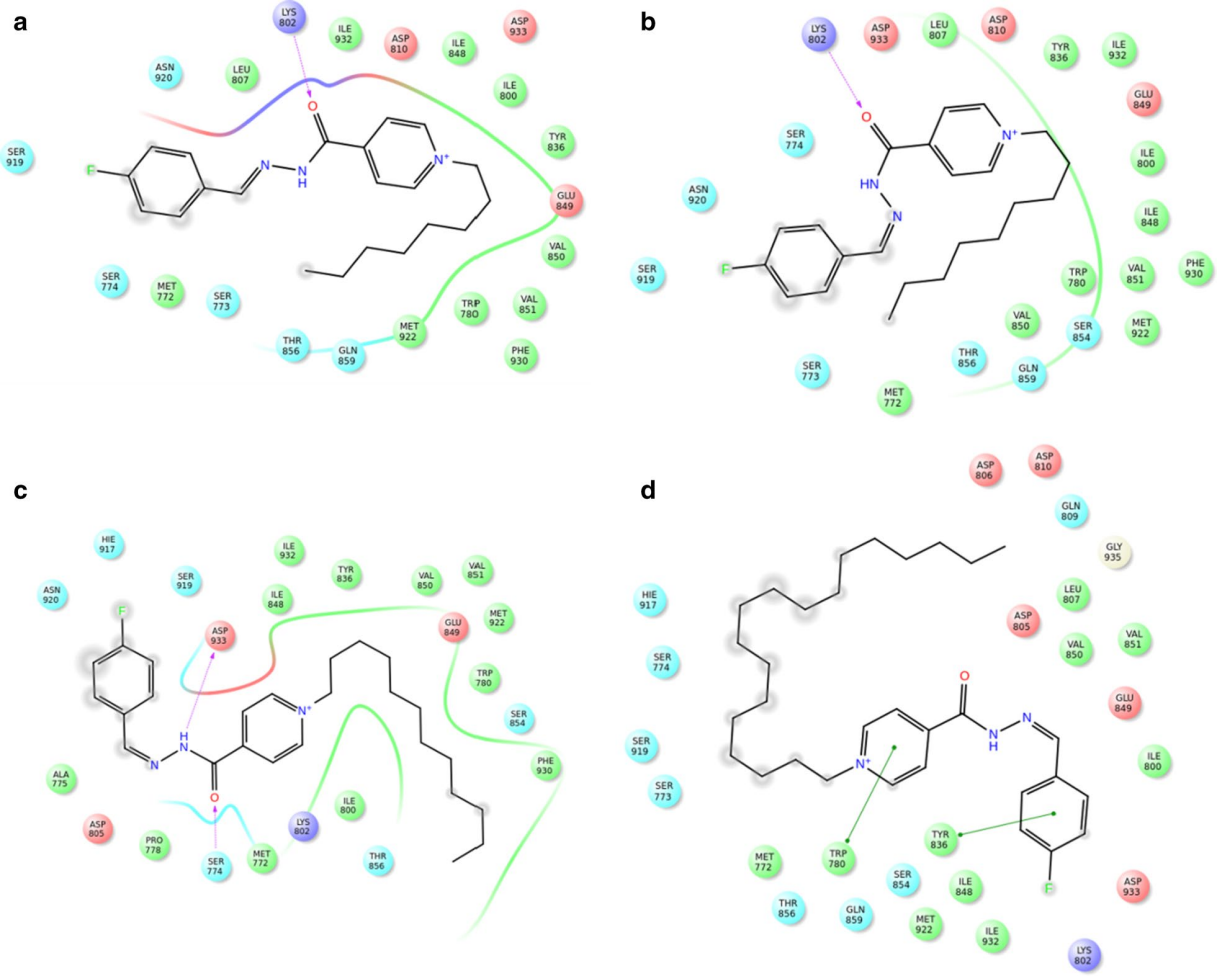
The ligand/protein complex of **a 2**, **b 3**, **c 6**, and **d 9**

Some of key binding residues are shown and H atoms are hidden for clarity purpose. Picture is captured by PYMOL. The backbones of **2**–**9** tend to form H-bond with S774, K802, H917, and D933 (Table 5) (Fig. 3). Additionally, **2**–**9** showed comparable QPLD binding affinity thus referring that the flexibility of the side-chain carbon atoms might ameliorate the steric effect. Other computational [41–45] and experimental studies [21] reported the significance of these residues in PI3Kα/ligand formation.

**Table 5:** The QPLD docking scores (Kcal/mol) and H-bond interactions between the verified compounds **2**–**9** and PI3Kα

Compound no	Docking score (Kcal/mol)	H-bond
**2**	− 6.03	K802
**3**	− 5.93	K802
**4**	− 5.78	D933
**5**	− 6.16	H917, D933
**6**	− 5.69	S774, D933
**7**	− 5.68	NA
**8**	− 5.36	K802
**9**	− 4.58	NA

Noticing that the whole synthesized compounds, **2**–**18** and **22**–**23**, share the core nucleus but differs in the side-chain carbon atoms number as well as the counterpart anion, for example **2** matches **10**, **11**, and **12**. It’s worth noting that the effect of salt enhances compound solubility and assists for better biological investigation.

**Fig. 4:**
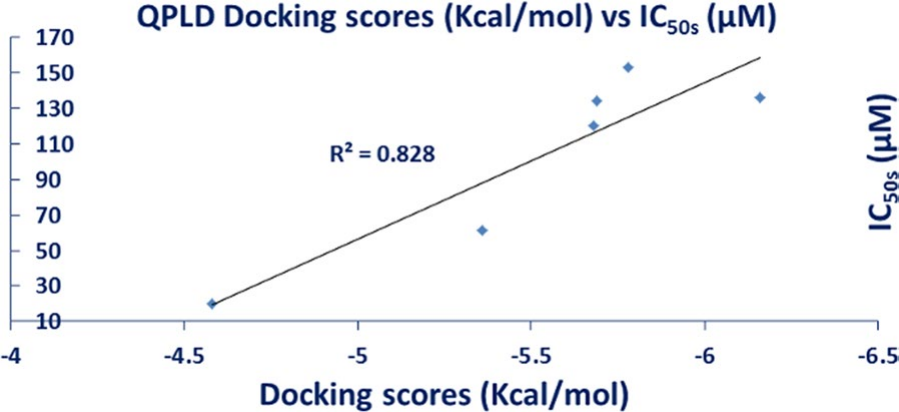
The correlation between the QPLD docking scores and between IC_50_ for the tested compounds

Contrarily, in silico modeling neglects the effect of the counterpart anion thus we carried out the docking studies for **2**–**9** as representative models for the whole dataset. Figure 4 shows that there is a positive correlation factor (R^2^ = 0.828) between the QPLD docking scores against PI3Kα and IC_50_.

**Fig. 5:**
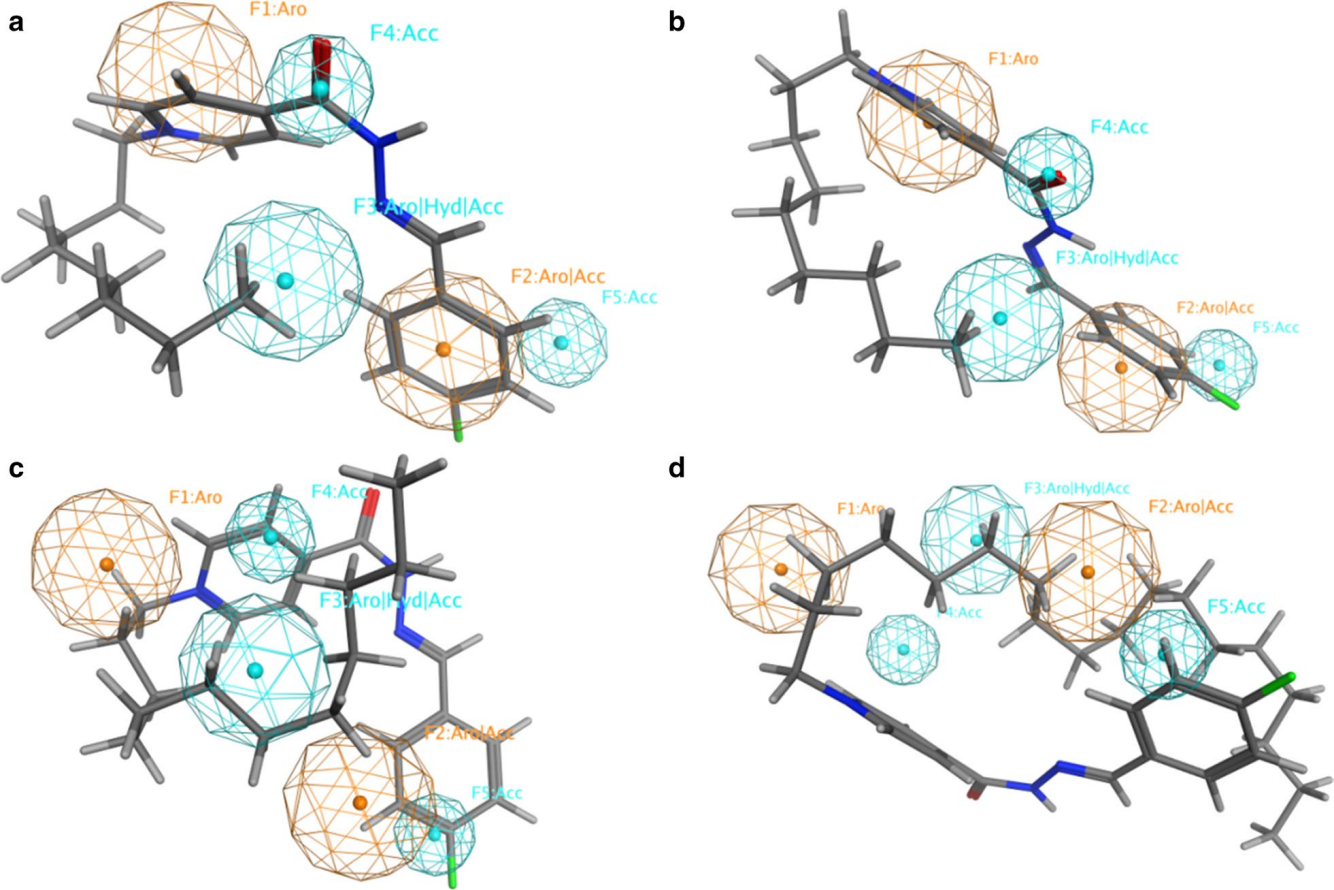
PI3Kα inhibitor pharmacophore model with **a 2**, **b 3**, **c 6**, and **d 9**. Aro stands for aromatic ring; Acc for H-bond acceptor; and Hyd for hydrophobic group. Picture made by MOE^52^

In order to get further details about the functionalities of **2**–**9**, we screened them against a reported PI3Kα inhibitor pharmacophore model [42]. The verified compounds **2**–**9** sparingly match the fingerprint of active PI3Kα inhibitors; three out of five functionalities for **2**–**9** (Fig. 5a, b) whereas two out of five functionalities for **6**–**9** (Fig. 5c, d). This finding explains their moderate to weak PI3Kα inhibitory activity and recommends optimizing the core skeleton of this library aiming to improve the biological activity.

Strikingly, the biological activity of **8**–**9** would suggest that the hydrophobicity of the attached alkyl group as well as the lipid membrane solubility parameter might affect their attachment to the cell line membrane.

**Fig. 6:**
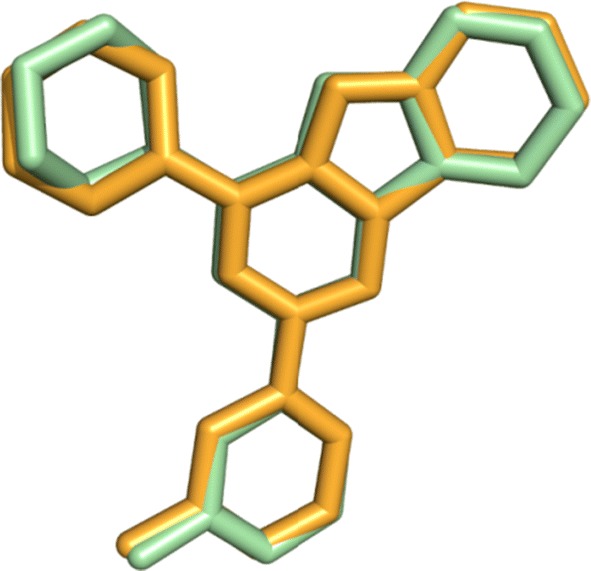
The superposition of KWT QPLD-docked pose and its native conformation in 3HHM. The native coordinates are represented in orange and the docked pose in green color. Picture visualized by PYMOL

In order to evaluate the performance of QPLD program, we compared the QPLD-docked pose of KWT in the mutant H1047R PI3Kα (PDB ID: 3HHM) [46] to its native conformation in the crystal structure. Figure 6 shows the superposition of the QPLD-generated KWT pose and the native conformation in 3HHM. The RMSD for heavy atoms of KWT between QPLD-generated docked pose and the native pose was 0.409 Å. This demonstrates that QPLD dock is able to reproduce the native conformation in the crystal structure and can reliably predict the ligand binding conformation.

## Experimental

### Apparatus and analysis

The Stuart Scientific SMP1 apparatus (Stuart, Red Hill, UK) was used in recording of the uncorrected melting points.

The SHIMADZU FTIR-8400S spectrometer (SHIMADZU, Boston, MA, USA) was used on the IR measurement.

The Bruker spectrometer (400 and 600 MHz, Brucker, Fällanden, Switzerland) was used in the NMR analysis using Tetramethylsilane (TMS) (0.00 ppm) as an internal standard.

The Finnigan LCQ and Finnigan MAT 95XL spectrometers (Finnigan, Darmstadt, Germany) were used in the ESI and EI measurement, respectively.

The Kunshan KQ-250B ultrasound cleaner (50 kHz, 240 W, Kunshan Ultrasonic Instrument, Kunshan, China) was used for carrying out all reactions.

### General alkylation procedure for the synthesis of cationic amphiphilic fluorinated pyridinium hydrazones **2**–**9**

#### Conventional method (CM)

To a mixture of pyridine hydrazone **1** (1 mmol) in acetonitrile (30 ml) was added an appropriate long alkyl iodides with chain ranging from C_8_ to C_18_ (1.5 mmol) under stirring. The mixture was refluxed for 72 h, then the solvent was reduced under pressure. The obtained solid was collected by filtration and washed with acetonitrile to give the target ILs **2**–**9**.

#### Ultrasound method (US)

To a mixture of pyridine hydrazone **1** (1 mmol) in acetonitrile (30 ml) was added an appropriate long alkyl iodides with chain ranging from C_8_ to C_18_ (1.5 mmol) under stirring. The mixture was irradiated by ultrasound irradiation for 10–12 h. The reaction was processed as described above to give the same target ILs **2**–**9**.

##### *4*-*(2*-*(4*-*Fluorobenzylidene) hydrazinecarbonyl)*-*1*-*octylpyridin*-*1*-*ium iodide (****2****)*

It was obtained as yellow crystals; mp: 104–105 °C. FT-IR (KBr), cm^−1^: ῡ = 1595 (C=N), 1670 (C=O), 2870, 2960 (Al–H), 3071 (Ar–H). ^1^H NMR (400 MHz, DMSO-*d*_6_): δ_H_ = 0.83–0.87 (m, 3H, C**H**_3_), 1.25–1.32 (m, 10H, 5× C**H**_2_), 1.94–1.99 (m, 2H, NCH_2_C**H**_2_), 4.68 (t, 2H, *J* = 8 Hz, NC**H**_2_), 7.22 (t, 0.5H, *J* = 8 Hz, Ar–**H**), 7.34 (t, 1.5H, *J* = 8 Hz, Ar–**H**), 7.62 (dd, 0.5H, *J* = 4 Hz, 8 Hz, Ar–**H**), 7.88 (dd, 1.5H, *J* = 4 Hz, 8 Hz, Ar–**H**), 8.16 (s, 0.25H, **H**–C=N), 8.39 (d, 0.5H, *J* = 4 Hz, Ar–**H**), 8.50 (s, 0.75H, **H**–C=N), 8.53 (d, 1.5H, *J* = 8 Hz, Ar–**H**), 9.25 (d, 0.5H, *J* = 8 Hz, Ar–**H**), 9.33 (d, 1.5H, *J* = 4 Hz, Ar–**H**), 12.47 (bs, 1H, CON**H**). ^13^C NMR (100 MHz, DMSO-*d*_6_): δ_C_ = 13.89 (**C**H_3_), 21.99, 25.36, 25.41, 28.30, 28.40, 30.50, 30.63, 31.08 (6×**C**H_2_), 60.95, 61.02 (N**C**H_2_), 115.74, 115.95, 116.17, 126.14, 127.11, 129.36, 129.44, 129.73, 129.81, 130.21, 130.24, 145.08, 145.67, 147.33, 149.36, 149.63 (Ar–**C**), 158.76, 162.28, 164.75, 165.21 (**C**=N, **C**=O). ^19^F NMR (377 MHz, DMSO-*d*_6_): δ_F_ = (− 109.72 to − 109.65), (− 109.20 to − 109.12) (2m, 1F, Ar–**F**). MS (ES) *m/z *= 483.32 [M^+^].

##### *4*-*(2*-*(4*-*Fluorobenzylidene) hydrazinecarbonyl)*-*1*-*nonylpyridin*-*1*-*ium iodide (****3****)*

It was obtained as yellow crystals; mp: 91–93 °C. FT-IR (KBr), cm^−1^: ῡ= 1598 (C=N), 1682 (C=O), 2872, 2969 (Al–H), 3078 (Ar–H). ^1^H NMR (400 MHz, DMSO-*d*_6_): δ_H_ = 0.83–0.87 (m, 3H, C**H**_3_), 1.25–1.32 (m, 12H, 6× C**H**_2_), 1.94–1.99 (m, 2H, NCH_2_C**H**_2_), 4.69 (dd, 2H, *J* = 4 Hz, 8 Hz, NC**H**_2_), 7.25 (dd, 0.5H, *J* = 8 Hz, 12 Hz, Ar–**H**), 7.37 (dd, 1.5H, *J* = 8 Hz, 12 Hz, Ar–**H**), 7.62 (dd, 0.5H, *J* = 4 Hz, 8 Hz, Ar–**H**), 7.89 (dd, 1.5H, *J* = 4 Hz, 8 Hz, Ar–**H**), 8.15 (s, 0.25H, **H**–C=N), 8.40 (d, 0.5H, *J* = 8 Hz, Ar–**H**), 8.50 (s, 0.75H, **H**–C=N), 8.53 (d, 1.5H, *J* = 8 Hz, Ar–**H**), 9.25 (d, 0.5H, *J* = 8 Hz, Ar–**H**), 9.33 (d, 1.5H, *J* = 8 Hz, Ar–**H**), 12.46 (s, 0.75H, CON**H**), 12.51 (s, 0.25H, CON**H**). ^13^C NMR (100 MHz, DMSO-*d*_6_): δ_C_ = 13.92 (**C**H_3_), 22.03, 25.36, 25.41, 28.35, 28.52, 28.70, 30.51, 30.64, 31.18 (7×**C**H_2_), 60.93, 61.01 (N**C**H_2_), 115.74, 115.96, 116.18, 126.15, 127.11, 129.35, 129.43, 129.73, 129.82, 130.20, 130.23, 145.06, 145.69, 147.31, 149.33, 149.64 (Ar–**C**), 158.75, 162.28, 164.76, 165.23 (**C**=N, **C**=O). ^19^F NMR (377 MHz, DMSO-*d*_6_): δ_F_ = (− 109.94 to − 109.86), (− 109.42 to − 109.34) (2m, 1F, Ar–**F**). MS (ES) *m/z *= 497.10 [M^+^].

##### *1*-*Decyl*-*4*-*(2*-*(4*-*fluorobenzylidene) hydrazinecarbonyl)pyridin*-*1*-*ium iodide (****4****)*

It was obtained as yellow crystals; mp: 110–112 °C. FT-IR (KBr), cm^−1^: ῡ = 1615 (C=N), 1690 (C=O), 2873, 2966 (Al–H), 3074 (Ar–H). ^1^H NMR (400 MHz, DMSO-*d*_6_): δ_H_ = 0.83–0.87 (m, 3H, C**H**_3_), 1.25–1.32 (m, 14H, 7× C**H**_2_), 1.94–1.99 (m, 2H, NCH_2_C**H**_2_), 4.68 (t, 2H, *J* = 8 Hz, NC**H**_2_), 7.23 (t, 0.5H, *J* = 8 Hz, Ar–**H**), 7.38 (dd, 1.5H, *J* = 8 Hz, 12 Hz, Ar–**H**), 7.62 (dd, 0.5H, *J* = 4 Hz, 8 Hz, Ar–**H**), 7.89 (dd, 1.5H, *J* = 4 Hz, 8 Hz, Ar–**H**), 8.16 (s, 0.25H, **H**–C=N), 8.40 (d, 0.5H, *J* = 4 Hz, Ar–**H**), 8.50 (s, 0.75H, **H**–C=N), 8.54 (d, 1.5H, *J* = 8 Hz, Ar–**H**), 9.25 (d, 0.5H, *J* = 4 Hz, Ar–**H**), 9.34 (d, 1.5H, *J* = 8 Hz, Ar–**H**), 12.48 (bs, 1H, CON**H**). ^13^C NMR (100 MHz, DMSO-*d*_6_): δ_C_ = 12.40, 12.42 (**C**H_3_), 20.55, 23.85, 23.89, 26.84, 27.11, 27.24, 27.28, 27.32, 28.99, 29.13, 29.72 (8×**C**H_2_), 59.42, 59.49 (N**C**H_2_), 114.24, 114.46, 114.68, 124.63, 125.59, 127.84, 127.92, 128.22, 128.31, 128.55, 128.68, 128.71, 143.54, 144.18, 145.78, 147.80, 148.12 (Ar–**C**), 157.25, 160.77, 163.24, 163.73 (**C**=N, **C**=O). ^19^F NMR (377 MHz, DMSO-*d*_6_): δ_F_ = (− 109.94 to − 109.85), (− 109.42 to − 109.34) (2m, 1F, Ar–**F**). MS (ES) *m/z *= 511.05 [M^+^].

##### *4*-*(2*-*(4*-*Fluorobenzylidene)hydrazinecarbonyl)*-*1*-*undecylpyridin*-*1*-*ium iodide (****5****)*

It was obtained as yellow crystals; mp: 82–83 °C. FT-IR (KBr), cm^−1^: ῡ = 1598 (C=N), 1677 (C=O), 2872, 2967 (Al–H), 3078 (Ar–H). ^1^H NMR (400 MHz, DMSO-*d*_6_): δ_H_ = 0.83–0.87 (m, 3H, C**H**_3_), 1.24–1.32 (m, 16H, 8× C**H**_2_), 1.96–1.99 (m, 2H, NCH_2_C**H**_2_), 4.68 (t, 2H, *J* = 8 Hz, NC**H**_2_), 7.22 (t, 0.5H, *J* = 8 Hz, Ar–**H**), 7.34 (t, 1.5H, *J* = 8 Hz, Ar–**H**), 7.62 (dd, 0.5H, *J* = 4 Hz, 8 Hz, Ar–**H**), 7.89 (dd, 1.5H, *J* = 4 Hz, 8 Hz, Ar–**H**), 8.16 (s, 0.25H, **H**–C=N), 8.39 (d, 0.5H, *J* = 4 Hz, Ar–**H**), 8.50 (s, 0.75H, **H**–C=N), 8.53 (d, 1.5H, *J* = 8 Hz, Ar–**H**), 9.25 (d, 0.5H, *J* = 8 Hz, Ar–**H**), 9.34 (d, 1.5H, *J* = 8 Hz, Ar–**H**), 12.45 (bs, 1H, CON**H**). ^13^C NMR (100 MHz, DMSO-*d*_6_): δ_C_ = 12.39 (**C**H_3_), 20.53, 23.86, 26.83, 27.13, 27.23, 27.37, 27.40, 28.98, 29.12, 29.74 (9×**C**H_2_), 59.46, 59.53 (N**C**H_2_), 114.23, 114.44, 114.66, 124.63, 125.61, 127.85, 127.93, 128.22, 128.31, 128.53, 128.56, 128.71, 128.74, 143.58, 144.18, 145.82, 147.88, 148.15 (Ar–**C**), 157.23, 160.78, 163.26, 163.69 (**C**=N, **C**=O). ^19^F NMR (377 MHz, DMSO-*d*_6_): δ_F_ = (− 109.95 to − 109.88), (− 109.35 to − 109.37) (2m, 1F, Ar–**F**). MS (ES) *m/z *= 525.10 [M^+^].

##### *1*-*Dodecyl*-*4*-*(2*-*(4*-*fluorobenzylidene) hydrazinecarbonyl)pyridin*-*1*-*ium iodide (****6****)*

It was obtained as yellow crystals; mp: 72–73 °C. FT-IR (KBr), cm^−1^: ῡ = 1605 (C=N), 1688 (C=O), 2883, 2961 (Al–H), 3074 (Ar–H). ^1^H NMR (400 MHz, DMSO-*d*_6_): δ_H_ = 0.83–0.87 (m, 3H, C**H**_3_), 1.24–1.32 (m, 18H, 9× C**H**_2_), 1.96–1.99 (m, 2H, NCH_2_C**H**_2_), 4.70 (dd, 2H, *J* = 4 Hz, 8 Hz, NC**H**_2_), 7.22 (t, 0.5H, *J* = 8 Hz, Ar–**H**), 7.34 (t, 1.5H, *J* = 8 Hz, Ar–**H**), 7.62 (dd, 0.5H, *J* = 4 Hz, 8 Hz, Ar–**H**), 7.88 (dd, 1.5H, *J* = 4 Hz, 8 Hz, Ar–**H**), 8.16 (s, 0.25H, **H**–C=N), 8.39 (d, 0.5H, *J* = 4 Hz, Ar–**H**), 8.50 (s, 0.75H, **H**–C=N), 8.53 (d, 1.5H, *J* = 8 Hz, Ar–**H**), 9.25 (d, 0.5H, *J* = 4 Hz, Ar–**H**), 9.34 (d, 1.5H, *J* = 8 Hz, Ar–**H**), 12.46 (bs, 1H, CON**H**). ^13^C NMR (100 MHz, DMSO-*d*_6_): δ_C_ = 11.54, 11.59 (**C**H_3_), 19.68, 23.00, 25.98, 26.30, 26.38, 26.51, 26.60, 28.13, 28.27, 28.88 (10× **C**H_2_), 58.60, 58.67 (N**C**H_2_), 113.37, 113.59, 113.80, 123.78, 124.75, 127.00, 127.08, 127.36, 127.45, 127.86, 127.89, 142.72, 143.33, 144.97, 147.02, 127.29 (Ar–**C**), 156.38, 159.93, 162.40, 162.83 (**C**=N, **C**=O). ^19^F NMR (377 MHz, DMSO-*d*_6_): δ_F_ = (− 109.95 to − 109.88), (− 109.44 to − 109.36) (2m, 1F, Ar–**F**). MS (ES) *m/z *= 539.40 [M^+^].

##### *4*-*(2*-*(4*-*Fluorobenzylidene)hydrazinecarbonyl)*-*1*-*tetradecylpyridin*-*1*-*ium iodide (****7****)*

It was obtained as yellow crystals; mp: 86–88 °C. FT-IR (KBr), cm^−1^: ῡ = 1590 (C=N), 1679 (C=O), 2878, 2964 (Al–H), 3078 (Ar–H). ^1^H NMR (400 MHz, DMSO-*d*_6_): δ_H_ = 0.83–0.86 (m, 3H, C**H**_3_), 1.24–1.32 (m, 22H, 11× C**H**_2_), 1.94–1.98 (m, 2H, NCH_2_C**H**_2_), 4.68 (t, 2H, *J* = 8 Hz, NC**H**_2_), 7.22 (t, 0.5H, *J* = 8 Hz, Ar–**H**), 7.34 (t, 1.5H, *J* = 8 Hz, Ar–**H**), 7.62 (dd, 0.5H, *J* = 4 Hz, 8 Hz, Ar–**H**), 7.89 (dd, 1.5H, *J* = 4 Hz, 8 Hz, Ar–**H**), 8.16 (s, 0.25H, **H**–C=N), 8.39 (d, 0.5H, *J* = 4 Hz, Ar–**H**), 8.50 (s, 0.75H, **H**–C=N), 8.53 (d, 1.5H, *J* = 8 Hz, Ar–**H**), 9.25 (d, 0.5H, *J* = 8 Hz, Ar–**H**), 9.33 (d, 1.5H, *J* = 4 Hz, Ar–**H**), 12.44 (s, 0.75H, CON**H**), 12.49 (s, 0.25H, CON**H**). ^13^C NMR (100 MHz, DMSO-*d*_6_): δ_C_ = 13.89 (**C**H_3_), 22.03, 25.36, 27.80, 28.34, 28.65, 28.74, 28.86, 28.96, 28.99, 29.77, 30.48, 30.62, 31.24, 32.85 (12×**C**H_2_), 60.96, 61.03 (N**C**H_2_), 115.73, 115.94, 116.16, 126.13, 127.11, 129.34, 129.43, 129.72, 129.81, 130.21, 130.24, 145.08, 145.68, 147.31, 149.38, 149.65 (Ar–**C**), 158.73, 162.29, 164.29, 165.18 (**C**=N, **C**=O). ^19^F NMR (377 MHz, DMSO-*d*_6_): δ_F_ = (− 109.96 to − 109.89), (− 109.44 to − 109.36) (2m, 1F, Ar–**F**). MS (ES) *m/z *= 567.20 [M^+^].

##### *4*-*(2*-*(4*-*Fluorobenzylidene)hydrazinecarbonyl)*-*1*-*hexadecylpyridin*-*1*-*ium iodide (****8****)*

It was obtained as yellow crystals; mp: 78–80 °C. FT-IR (KBr), cm^−1^: ῡ = 1610 (C=N), 1677 (C=O), 2887, 2969 (Al–H), 3076 (Ar–H). ^1^H NMR (400 MHz, DMSO-*d*_6_): δ_H_ = 0.83–0.86 (m, 3H, C**H**_3_), 1.23–1.30 (m, 26H, 13× C**H**_2_), 1.96–1.98 (m, 2H, NCH_2_C**H**_2_), 4.68 (t, 2H, *J* = 8 Hz, NC**H**_2_), 7.22 (t, 0.5H, *J* = 8 Hz, Ar–**H**), 7.34 (t, 1.5H, *J* = 8 Hz, Ar–**H**), 7.62 (dd, 0.5H, *J* = 4 Hz, 8 Hz, Ar–**H**), 7.89 (dd, 1.5H, *J* = 4 Hz, 8 Hz, Ar–**H**), 8.16 (s, 0.25H, **H**–C=N), 8.39 (d, 0.5H, *J* = 4 Hz, Ar–**H**), 8.50 (s, 0.75H, **H**–C=N), 8.53 (d, 1.5H, *J* = 8 Hz, Ar–**H**), 9.25 (d, 0.5H, *J* = 8 Hz, Ar–**H**), 9.34 (d, 1.5H, *J* = 4 Hz, Ar–**H**), 12.45 (s, 0.75H, CON**H**), 12.49 (s, 0.25H, CON**H**). ^13^C NMR (100 MHz, DMSO-*d*_6_): δ_C_ = 13.88 (**C**H_3_), 22.03, 25.36, 28.34, 28.64, 28.74, 28.87, 28.96, 29.00, 30.49, 30.62, 31.24 (12×**C**H_2_), 60.96, 61.03 (N**C**H_2_), 115.73, 115.94, 116.16, 126.14, 127.11, 129.34, 129.43, 129.72, 129.81, 130.04, 130.24, 145.08, 145.69, 147.31, 149.37 (Ar–**C**), 158.72, 162.29, 164.76, 165.18 (**C**=N, **C**=O). ^19^F NMR (377 MHz, DMSO-*d*_6_): δ_F_ = (− 109.97 to − 109.89), (− 109.45 to − 109.37) (2m, 1F, Ar–**F**). MS (ES) *m/z *= 595.30 [M^+^].

##### *4*-*(2*-*(4*-*Fluorobenzylidene)hydrazinecarbonyl)*-*1*-*octadecylpyridin*-*1*-*ium iodide (****9****)*

It was obtained as yellow crystals; mp: 98–99 °C. FT-IR (KBr), cm^−1^: ῡ= 1612 (C=N), 1678 (C=O), 2887, 2955 (Al–H), 3086 (Ar–H). ^1^H NMR (400 MHz, CDCl_3_): δ_H_ = 0.79–0.82 (m, 3H, C**H**_3_), 1.16–1.20 (m, 30H, 15× C**H**_2_), 1.96–2.00 (m, 2H, NCH_2_C**H**_2_), 4.78 (dd, 2H, *J* = 4 Hz, 8 Hz, NC**H**_2_), 6.97 (t, 2H, *J* = 8 Hz, Ar–**H**), 7.71 (dd, 2H, *J* = 4 Hz, 8 Hz, Ar–**H**), 8.87 (d, 2H, *J* = 4 Hz, Ar–**H**), 9.08 (s, 1H, **H**–C=N), 9.12 (d, 2H, *J* = 8 Hz, Ar–**H**), 12.18 (bs, 1H, CON**H**). ^13^C NMR (100 MHz, CDCl_3_): δ_C_ = 14.08 (**C**H_3_), 22.66, 26.10, 28.96, 29.31, 29.33, 29.48, 29.57, 29.63, 29.68, 31.67, 31.90 (16× **C**H_2_), 62.74 (N**C**H_2_), 115.85, 116.07, 127.88, 129.47, 130.14, 130.22, 144.82, 147.91, 151.67 (Ar–**C**), 158.57, 163.22, 163.25, 165.76 (**C**=N, **C**=O). ^19^F NMR (377 MHz, CDCl_3_): δ_F_ = (− 107.98 to − 107.89), (− 107.72 to − 107.65) (2 m, 1F, Ar–**F**). MS (ES) *m/z *= 623.30 [M^+^].

### General metathesis procedure for the synthesis of pyridinium hydrazones **10**–**33**

#### Conventional method (CM)

A mixture of equimolar of IL **2**–**9** (1 mmol) and fluorinated metal salt (KPF_6_, NaBF_4_ and/or NaCF_3_COO) (1 mmol) in dichloromethane (15 ml) was heated under reflux for 12 h. After cooling, the solid formed was collected by extraction and/or by filtration. The solid was washed by dichloromethane to afford the task-specific ILs **10**–**33**.

#### Ultrasound method (US)

A mixture of equimolar of IL **2**–**9** (1 mmol) and fluorinated metal salt (KPF_6_, NaBF_4_ and/or NaCF_3_COO) (1 mmol) in dichloromethane (15 ml) was irradiated by ultrasound irradiation for 6 h. The reaction was processed as described above to give the same task-specific ILs **10**–**33**.

##### *4*-*(2*-*(4*-*Fluorobenzylidene) hydrazinecarbonyl)*-*1*-*octylpyridin*-*1*-*ium hexafluorophosphate (****10****)*

It was obtained as yellow crystals; mp: 64–65 °C. ^1^H NMR (400 MHz, DMSO-*d*_6_): δ_H_ = 0.82–0.88 (m, 3H, C**H**_3_), 1.26–1.30 (m, 10H, 5×C**H**_2_), 1.94–2.00 (m, 2H, NCH_2_C**H**_2_), 4.68 (t, 2H, *J* = 8 Hz, NC**H**_2_), 7.26 (dd, 0.5H, *J* = 8 Hz, 12 Hz, Ar–**H**), 7.38 (dd, 1.5H, *J* = 8 Hz, 12 Hz, Ar–**H**), 7.62 (dd, 0.5H, *J* = 4 Hz, 8 Hz, Ar–**H**), 7.89 (dd, 1.5H, *J* = 4 Hz, 8 Hz, Ar–**H**), 8.16 (s, 0.25H, **H**–C=N), 8.40 (d, 0.5H, *J* = 4 Hz, Ar–**H**), 8.50 (s, 0.75H, **H**–C=N), 8.53 (d, 1.5H, *J* = 4 Hz, Ar–**H**), 9.25 (d, 0.5H, *J* = 4 Hz, Ar–**H**), 9.33 (d, 1.5H, *J* = 4 Hz, Ar–**H**), 12.50 (bs, 1H, CON**H**).^13^C NMR (100 MHz, DMSO-*d*_6_): δ_C_ = 13.09 (**C**H_3_), 22.00, 25.36, 25.41, 28.30, 28.40, 30.51, 30.64, 31.09 (6×**C**H_2_), 60.95, 61.02 (N**C**H_2_), 115.75, 115.96, 116.18, 126.14, 127.11, 129.35, 129.44, 129.73, 129.81, 130.05, 130.24, 130.24, 145.06, 145.67, 147.35, 149.35, 149.63 (Ar–**C**), 158.78, 162.28, 164.75, 165.22 (**C**=N, **C**=O). ^31^P NMR (162 MHz, DMSO-*d*_6_): δ_P_ = − 152.70 to − 135.29 (m, 1P, **P**F_6_). ^19^F NMR (377 MHz, DMSO-*d*_6_): δ_F_ = − 69.98 (d, 6F, P**F**_**6**_), (− 109.72 to − 109.65), (− 109.20 to − 109.12) (2m, 1F, Ar–**F**). MS (ES) *m/z *= 501.20 [M^+^].

##### *4*-*(2*-*(4*-*Fluorobenzylidene) hydrazinecarbonyl)*-*1*-*octylpyridin*-*1*-*ium tetrafluoroborate (****11****)*

It was obtained as yellow crystals; mp: 80–82 °C. ^1^H NMR (400 MHz, DMSO-*d*_6_): δ_H_ = 0.84–0.88 (m, 3H, C**H**_3_), 1.26–1.31 (m, 10H, 5×C**H**_2_), 1.95–2.00 (m, 2H, NCH_2_C**H**_2_), 4.70 (dd, 2H, *J* = 4 Hz, 8 Hz, NC**H**_2_), 7.26 (dd, 0.5H, *J* = 8 Hz, 12 Hz, Ar–**H**), 7.38 (dd, 1.5H, *J* = 8 Hz, 12 Hz, Ar–**H**), 7.63 (dd, 0.5H, *J* = 4 Hz, 8 Hz, Ar–**H**), 7.90 (dd, 1.5H, *J* = 4 Hz, 8 Hz, Ar–**H**), 8.16 (s, 0.25H, **H**–C=N), 8.41 (d, 0.5H, *J* = 8 Hz, Ar–**H**), 8.51 (s, 0.75H, **H**–C=N), 8.54 (d, 1.5H, *J* = 4 Hz, Ar–**H**), 9.27 (d, 0.5H, *J* = 8 Hz, Ar–**H**), 9.36 (d, 1.5H, *J* = 8 Hz, Ar–**H**), 12.49 (s, 0.75H, CON**H**), 12.53 (s, 0.25H, CON**H**).^13^C NMR (100 MHz, DMSO-*d*_6_): δ_C_ = 13.87 (**C**H_3_), 21.97, 25.32, 25.38, 28.27, 28.37, 28.40, 30.48, 30.61, 31.06 (6× **C**H_2_), 60.89, 60.96 (N**C**H_2_), 115.71, 115.92, 116.14, 126.10, 127.07, 129.33, 129.41, 129.69, 129.78, 130.01, 130.15, 130.18, 145.02, 145.65, 147.23, 149.28, 149.57 (Ar–**C**), 158.72, 161.89, 162.23, 164.70, 165.19 (**C**=N, **C**=O).^11^B NMR (128 MHz, DMSO-*d*_6_): δ_B_ = − 1.31 to − 1.30 (m, 1B, **B**F_4_). ^19^F NMR (377 MHz, DMSO-*d*_6_): δ_F_ = (− 109.82 to − 109.74), (− 109.29 to − 109.21) (2m, 1F, Ar–**F**); − 148.12, − 148.07 (2d, 4F, B**F**_4_). MS (ES) *m/z *= 443.20 [M^+^].

##### *4*-*(2*-*(4*-*Fluorobenzylidene) hydrazinecarbonyl)*-*1*-*octylpyridin*-*1*-*ium trifluoroacetate (****12****)*

It was obtained as yellow crystals; mp: 74–76 °C. ^1^H NMR (400 MHz, DMSO-*d*_6_): δ_H_ = 0.84–0.88 (m, 3H, C**H**_3_), 1.26–1.30 (m, 10H, 5×C**H**_2_), 1.95–1.97 (m, 2H, NCH_2_C**H**_2_), 4.69 (dd, 2H, *J* = 4 Hz, 8 Hz, NC**H**_2_), 7.26 (dd, 0.5H, *J* = 8 Hz, 12 Hz, Ar–**H**), 7.35 (t, 1.5H, *J* = 8 Hz, Ar–**H**), 7.62 (dd, 0.5H, *J* = 4 Hz, 8 Hz, Ar–**H**), 7.88 (dd, 1.5H, *J* = 4 Hz, 8 Hz, Ar–**H**), 8.16 (s, 0.25H, **H**–C=N), 8.40 (d, 0.5H, *J* = 4 Hz, Ar–**H**), 8.49 (s, 0.75H, **H**–C=N), 8.54 (d, 1.5H, *J* = 8 Hz, Ar–**H**), 9.25 (d, 0.5H, *J* = 4 Hz, Ar–**H**), 9.32 (d, 1.5H, *J* = 8 Hz, Ar–**H**), 12.54 (bs, 1H, CON**H**).^13^C NMR (100 MHz, DMSO-*d*_6_): δ_C_ = 13.85 (**C**H_3_), 21.95, 25.30, 25.35, 28.25, 28.35, 28.38, 30.46, 30.58, 31.03 (6× **C**H_2_), 60.85, 60.88 (N**C**H_2_), 115.69, 115.88, 116.10, 126.05, 127.04, 129.28, 129.36, 129.61, 129.70, 129.99, 130.24, 130.27, 144.98, 145.54, 147.59, 149.20, 149.56 (Ar–**C**), 158.84, 162.15, 165.19 (**C**=N, **C**=O). ^19^F NMR (377 MHz, DMSO-*d*_6_): δ_F_ = − 73.50 (s, 3F, C**F**_**3**_), (− 109.92 to − 109.84), (− 109.53 to − 109.45) (2m, 1F, Ar–**F**). MS (ESI) *m/z *= 467.10 [M^+^ + 1].

##### *4*-*(2*-*(4*-*Fluorobenzylidene) hydrazinecarbonyl)*-*1*-*nonylpyridin*-*1*-*ium hexafluorophosphate (****13****)*

It was obtained as yellow crystals; mp: 69–70 °C. ^1^H NMR (400 MHz, DMSO-*d*_6_): δ_H_ = 0.83–0.87 (m, 3H, C**H**_3_), 1.25–1.30 (m, 12H, 6×C**H**_2_), 1.94–1.99 (m, 2H, NCH_2_C**H**_2_), 4.69 (dd, 2H, *J* = 4 Hz, 8 Hz, NC**H**_2_), 7.25 (dd, 0.5H, *J* = 8 Hz, 12 Hz, Ar–**H**), 7.37 (dd, 1.5H, *J* = 8 Hz, 12 Hz, Ar–**H**), 7.62 (dd, 0.5H, *J* = 4 Hz, 8 Hz, Ar–**H**), 7.89 (dd, 1.5H, *J* = 4 Hz, 8 Hz, Ar–**H**), 8.15 (s, 0.25H, **H**–C=N), 8.40 (d, 0.5H, *J* = 8 Hz, Ar–**H**), 8.51 (s, 0.75H, **H**–C=N), 8.54 (d, 1.5H, *J* = 8 Hz, Ar–**H**), 9.24 (d, 0.5H, *J* = 4 Hz, Ar–**H**), 9.33 (d, 1.5H, *J* = 8 Hz, Ar–**H**), 12.51 (s, 1H, CON**H**). ^13^C NMR (100 MHz, DMSO-*d*_6_): δ_C_ = 13.92 (**C**H_3_), 22.03, 25.36, 25.41, 28.35, 28.52, 28.70, 28.74, 30.51, 30.64, 31.18 (7×**C**H_2_), 60.93, 61.01 (N**C**H_2_), 115.74, 115.96, 116.18, 126.16, 127.11, 129.34, 129.43, 129.72, 129.81, 130.21, 130.24, 145.06, 145.68, 147.30, 149.34 (Ar–**C**), 158.75, 162.28, 164.75, 165.23 (**C**=N, **C**=O). ^31^P NMR (162 MHz, DMSO-*d*_6_): δ_P_ = − 152.98 to − 135.42 (m, 1P, **P**F_6_). ^19^F NMR (377 MHz, DMSO-*d*_6_): δ_F_ = − 69.21 (d, 6F, P**F**_**6**_), (− 109.94 to − 109.86), (− 109.42 to − 109.34) (2m, 1F, Ar–**F**). MS (ES) *m/z *= 515.20 [M^+^].

##### *4*-*(2*-*(4*-*Fluorobenzylidene) hydrazinecarbonyl)*-*1*-*nonylpyridin*-*1*-*ium tetrafluoroborate (****14****)*

It was obtained as yellow crystals; mp: 88–90 °C. ^1^H NMR (400 MHz, DMSO-*d*_6_): δ_H_ = 0.83–0.87 (m, 3H, C**H**_3_), 1.25–1.30 (m, 12H, 6×C**H**_2_), 1.95–1.99 (m, 2H, NCH_2_C**H**_2_), 4.67 (t, 2H, *J* = 8 Hz, NC**H**_2_), 7.25 (dd, 0.5H, *J* = 8 Hz, 12 Hz, Ar–**H**), 7.35 (t, 1.5H, *J* = 8 Hz, Ar–**H**), 7.61 (dd, 0.5H, *J* = 4 Hz, 8 Hz, Ar–**H**), 7.89 (dd, 1.5H, *J* = 4 Hz, 8 Hz, Ar–**H**), 8.15 (s, 0.25H, **H**–C=N), 8.40 (d, 0.5H, *J* = 8 Hz, Ar–**H**), 8.51 (s, 0.75H, **H**–C=N), 8.53 (d, 1.5H, *J* = 4 Hz, Ar–**H**), 9.24 (d, 0.5H, *J* = 8 Hz, Ar–**H**), 9.32 (d, 1.5H, *J* = 8 Hz, Ar–**H**), 12.49 (bs, 1H, CON**H**). ^13^C NMR (100 MHz, DMSO-*d*_6_): δ_C_ = 13.92 (**C**H_3_), 22.03, 25.36, 28.35, 28.52, 28.70, 30.51, 30.64, 31.18 (7×**C**H_2_), 60.94, 61.02 (N**C**H_2_), 115.74, 115.97, 116.19, 126.16, 127.11, 129.34, 129.43, 129.72, 129.81, 130.21, 145.07, 145.68, 147.32, 149.34 (Ar–**C**), 158.75, 162.29, 164.76, 165.24 (**C**=N, **C**=O).^11^B NMR (128 MHz, DMSO-*d*_6_): δ_B_ = − 1.31 to − 1.30 (m, 1B, **B**F_4_). ^19^F NMR (377 MHz, DMSO-*d*_6_): δ_F_ = (− 109.94 to − 109.86), (− 109.42 to − 109.34) (2m, 1F, Ar–**F**); − 148.29, − 148.24 (2d, 4F, B**F**_4_). MS (ES) *m/z *= 457.15 [M^+^].

##### *4*-*(2*-*(4*-*Fluorobenzylidene) hydrazinecarbonyl)*-*1*-*nonylpyridin*-*1*-*ium trifluoroacetate (****15****)*

It was obtained as yellow crystals; mp: 96–98 °C. ^1^H NMR (400 MHz, DMSO-*d*_6_): δ_H_ = 0.83–0.87 (t, 3H, *J* = 4 Hz, C**H**_3_), 1.25–1.30 (m, 12H, 6×C**H**_2_), 1.94–1.99 (m, 2H, NCH_2_C**H**_2_), 4.68 (t, 2H, *J* = 8 Hz, NC**H**_2_), 7.25 (dd, 0.5H, *J* = 8 Hz, 12 Hz, Ar–**H**), 7.37 (dd, 1.5H, *J* = 8 Hz, 12 Hz, Ar–**H**), 7.62 (dd, 0.5H, *J* = 4 Hz, 8 Hz, Ar–**H**), 7.88 (dd, 1.5H, *J* = 4 Hz, 8 Hz, Ar–**H**), 8.16 (s, 0.25H, **H**–C=N), 8.40 (d, 0.5H, *J* = 8 Hz, Ar–**H**), 8.51 (s, 0.75H, **H**–C=N), 8.53 (d, 1.5H, *J* = 4 Hz, Ar–**H**), 9.25 (d, 0.5H, *J* = 8 Hz, Ar–**H**), 9.33 (d, 1.5H, *J* = 4 Hz, Ar–**H**), 12.50 (s, 0.75H, CON**H**), 12.51 (s, 0.25H, CON**H**). ^13^C NMR (100 MHz, DMSO-*d*_6_): δ_C_ = 13.91 (**C**H_3_), 22.03, 25.36, 28.34, 25.41, 28.34, 28.52, 28.70, 28.73, 30.51, 30.64, 31.18 (7×**C**H_2_), 60.93, 61.00 (N**C**H_2_), 115.74, 115.96, 116.18, 126.15, 127.11, 129.34, 129.43, 129.80, 130.04, 130.21, 130.24, 145.07, 145.69, 147.31, 149.35, 149.65 (Ar–**C**), 158.75, 162.28, 164.75, 165.23 (**C**=N, **C**=O). ^19^F NMR (377 MHz, DMSO-*d*_6_): δ_F_ = − 73.50 (s, 3F, C**F**_**3**_), (− 109.96 to − 109.88), (− 109.44 to − 109.36) (2 m, 1F, Ar–**F**). MS (ES) *m/z *= 483.20 [M^+^].

##### *1*-*Decyl*-*4*-*(2*-*(4*-*fluorobenzylidene) hydrazinecarbonyl)pyridin*-*1*-*ium hexafluorophosphate (****16****)*

It was obtained as yellow syrup. ^1^H NMR (400 MHz, DMSO-*d*_6_): δ_H_ = 0.83–0.88 (m, 3H, C**H**_3_), 1.25–1.30 (m, 14H, 7×C**H**_2_), 1.95–1.98 (m, 2H, NCH_2_C**H**_2_), 4.67 (t, 2H, *J* = 8 Hz, NC**H**_2_), 7.25 (dd, 0.5H, *J* = 8 Hz, 12 Hz, Ar–**H**), 7.35 (t, 1.5H, *J* = 8 Hz, Ar–**H**), 7.62 (dd, 0.5H, *J* = 4 Hz, 8 Hz, Ar–**H**), 7.89 (dd, 1.5H, *J* = 4 Hz, 8 Hz, Ar–**H**), 8.16 (s, 0.25H, **H**–C=N), 8.40 (d, 0.5H, *J* = 8 Hz, Ar–**H**), 8.50 (s, 0.75H, **H**–C=N), 8.53 (d, 1.5H, *J* = 4 Hz, Ar–**H**), 9.23 (d, 0.5H, *J* = 4 Hz, Ar–**H**), 9.31 (d, 1.5H, *J* = 8 Hz, Ar–**H**), 12.48 (bs, 1H, CON**H**). ^13^C NMR (100 MHz, DMSO-*d*_6_): δ_C_ = 13.90 (**C**H_3_), 22.04, 25.36, 25.40, 28.33, 28.60, 28.74, 28.77, 28.82, 30.50, 30.63, 31.23 (8×**C**H_2_), 60.96, 61.06 (N**C**H_2_), 115.72, 115.95, 116.16, 126.15, 127.12, 129.32, 129.41, 129.72, 129.81, 130.07, 130.21, 130.24, 145.05, 145.67, 147.34, 149.36, 149.67, (Ar–**C**), 158.75, 162.28, 164.77, 165.22 (**C**=N, **C**=O). ^31^P NMR (162 MHz, DMSO-*d*_6_): δ_P_ = − 157.37 to − 131.02 (m, 1P, **P**F_6_). ^19^F NMR (377 MHz, DMSO-*d*_6_): δ_F_ = − 69.22 (d, 6F, P**F**_**6**_), (− 109.94 to − 109.85), (− 109.42 to − 109.34) (2m, 1F, Ar–**F**). MS (ES) *m/z *= 529.70 [M^+^].

##### *1*-*Decyl*-*4*-*(2*-*(4*-*fluorobenzylidene) hydrazinecarbonyl)pyridin*-*1*-*ium tetrafluoroborate (****17****)*

It was obtained as colorless syrup. ^1^H NMR (400 MHz, DMSO-*d*_6_): δ_H_ = 0.83–0.87 (m, 3H, C**H**_3_), 1.25–1.30 (m, 14H, 7×C**H**_2_), 1.95–1.98 (m, 2H, NCH_2_C**H**_2_), 4.67 (t, 2H, *J* = 8 Hz, NC**H**_2_), 7.25 (dd, 0.5H, *J* = 8 Hz, 12 Hz, Ar–**H**), 7.35 (t, 1.5H, *J* = 8 Hz, Ar–**H**), 7.62 (dd, 0.5H, *J* = 8 Hz, 12 Hz, Ar–**H**), 7.89 (dd, 1.5H, *J* = 4 Hz, 8 Hz, Ar–**H**), 8.16 (s, 0.25H, **H**–C=N), 8.40 (d, 0.5H, *J* = 8 Hz, Ar–**H**), 8.52 (s, 0.75H, **H**–C=N), 8.55 (d, 1.5H, *J* = 8 Hz, Ar–**H**), 9.24 (d, 0.5H, *J* = 4 Hz, Ar–**H**), 9.32 (d, 1.5H, *J* = 4 Hz, Ar–**H**), 12.52 (bs, 1H, CON**H**). ^13^C NMR (100 MHz, DMSO-*d*_6_): δ_C_ = 13.90, 13.91 (**C**H_3_), 22.05, 25.36, 25.40, 28.34, 28.61, 28.75, 28.78, 28.83, 30.50, 30.63, 31.23, (8×**C**H_2_), 60.94, 61.01 (N**C**H_2_), 115.74, 115.96, 116.18, 126.16, 127.11, 129.34, 129.42, 129.71, 129.80, 130.07, 130.23, 130.26, 145.07, 145.67, 147.34, 149.35 (Ar–**C**), 158.76, 162.28, 164.75, 165.23, (**C**=N, **C**=O). ^11^B NMR (128 MHz, DMSO-*d*_6_): δ_B_ = − 1.31 to − 1.29 (m, 1B, **B**F_4_). ^19^F NMR (377 MHz, DMSO-*d*_6_): δ_F_ = (− 109.94 to − 109.88), (− 109.44 to − 109.36) (2m, 1F, Ar–**F**); − 148.30, − 148.24 (2d, 4F, B**F**_4_). MS (ES) *m/z *= 471.60 [M^+^].

##### *1*-*Decyl*-*4*-*(2*-*(4*-*fluorobenzylidene) hydrazinecarbonyl)pyridin*-*1*-*ium trifluoroacetate (****18****)*

It was obtained as yellow syrup. ^1^H NMR (400 MHz, DMSO-*d*_6_): δ_H_ = 0.83–0.87 (m, 3H, C**H**_3_), 1.25–1.30 (m, 14H, 7×C**H**_2_), 1.95–1.98 (m, 2H, NCH_2_C**H**_2_), 4.68 (t, 2H, *J* = 8 Hz, NC**H**_2_), 7.25 (dd, 0.5H, *J* = 8 Hz, 12 Hz, Ar–**H**), 7.37 (dd, 1.5H, *J* = 8 Hz, 12 Hz, Ar–**H**), 7.62 (dd, 0.5H, *J* = 4 Hz, 8 Hz, Ar–**H**), 7.88 (dd, 1.5H, *J* = 4 Hz, 8 Hz, Ar–**H**), 8.17 (s, 0.25H, **H**–C=N), 8.40 (d, 0.5H, *J* = 8 Hz, Ar–**H**), 8.52 (s, 0.75H, **H**–C=N), 8.55 (d, 1.5H, *J* = 8 Hz, Ar–**H**), 9.25 (d, 0.5H, *J* = 4 Hz, Ar–**H**), 9.33 (d, 1.5H, *J* = 8 Hz, Ar–**H**), 12.56 (bs, 1H, CON**H**). ^13^C NMR (100 MHz, DMSO-*d*_6_): δ_C_ = 13.89, 13.91 (**C**H_3_), 22.05, 25.36, 25.40, 28.34, 28.61, 28.74, 28.78, 28.82, 30.50, 30.64, 31.23 (8×**C**H_2_), 60.94, 60.98 (N**C**H_2_), 115.74, 115.95, 116.16, 126.13, 127.11, 129.33, 129.42, 129.69, 129.77, 130.07, 130.28, 130.31, 145.07, 145.65, 147.48, 149.35 (Ar–**C**), 158.82, 162.25, 164.73, 165.23 (**C**=N, **C**=O). ^19^F NMR (377 MHz, DMSO-*d*_6_): δ_F_ = − 73.52 (s, 3F, C**F**_**3**_), (− 109.95 to − 109.87), (− 109.50 to − 109.42) (2m, 1F, Ar–**F**). MS (ES) *m/z *= 497.33 [M^+^].

##### *4*-*(2*-*(4*-*Fluorobenzylidene)hydrazinecarbonyl)*-*1*-*undecylpyridin*-*1*-*ium hexafluorophosphate (****19****)*

It was obtained as yellow syrup. ^1^H NMR (400 MHz, DMSO-*d*_6_): δ_H_ = 0.83–0.87 (m, 3H, C**H**_3_), 1.24–1.30 (m, 16H, 8×C**H**_2_), 1.96–1.99 (m, 2H, NCH_2_C**H**_2_), 4.69 (dd, 2H, *J* = 4 Hz, 8 Hz, NC**H**_2_), 7.22 (t, 0.5H, *J* = 8 Hz, Ar–**H**), 7.36 (dd, 1.5H, *J* = 4 Hz, 8 Hz, Ar–**H**), 7.61 (dd, 0.5H, *J* = 4 Hz, 8 Hz, Ar–**H**), 7.88 (dd, 1.5H, *J* = 4 Hz, 8 Hz, Ar–**H**), 8.16 (s, 0.25H, **H**–C=N), 8.39 (d, 0.5H, *J* = 4 Hz, Ar–**H**), 8.53 (s, 0.75H, **H**–C=N), 8.54 (d, 1.5H, *J* = 4 Hz, Ar–**H**), 9.24 (d, 0.5H, *J* = 4 Hz, Ar–**H**), 9.33 (d, 1.5H, *J* = 8 Hz, Ar–**H**), 12.51 (bs, 1H, CON**H**). ^13^C NMR (100 MHz, DMSO-*d*_6_): δ_C_ = 13.90 (**C**H_3_), 22.04, 25.36, 28.34, 28.64, 28.74, 28.87, 28.91, 30.49, 30.63, 31.24 (9×**C**H_2_), 60.95, 61.03 (N**C**H_2_), 115.73, 115.95, 116.17, 126.16, 127.11, 129.34, 129.42, 129.71, 128.80, 130.07, 130.26, 145.08, 145.67, 147.32, 149.38, 149.66 (Ar–**C**), 158.73, 162.28, 164.76, 165.20 (**C**=N, **C**=O). ^31^P NMR (162 MHz, DMSO-*d*_6_): δ_P_ = − 152.97 to − 135.41 (m, 1P, **P**F_6_). ^19^F NMR (377 MHz, DMSO-*d*_6_): δ_F_ = − 69.24 (d, 6F, P**F**_**6**_), (− 109.95 to − 109.88), (− 109.35 to − 109.37) (2m, 1F, Ar–**F**). MS (ES) *m/z *= 543.40 [M^+^].

##### *4*-*(2*-*(4*-*Fluorobenzylidene)hydrazinecarbonyl)*-*1*-*undecylpyridin*-*1*-*ium tetrafluoroborate (****20****)*

It was obtained as yellow syrup. ^1^H NMR (400 MHz, DMSO-*d*_6_): δ_H_ = 0.83–0.87 (m, 3H, C**H**_3_), 1.24–1.30 (m, 16H, 8×C**H**_2_), 1.96–1.99 (m, 2H, NCH_2_C**H**_2_), 4.68 (t, 2H, *J* = 8 Hz, NC**H**_2_), 7.22 (t, 0.5H, *J* = 8 Hz, Ar–**H**), 7.34 (t, 1.5H, *J* = 8 Hz, Ar–**H**), 7.61 (dd, 0.5H, *J* = 4 Hz, 8 Hz, Ar–**H**), 7.88 (dd, 1.5H, *J* = 4 Hz, 8 Hz, Ar–**H**), 8.17 (s, 0.25H, **H**–C=N), 8.39 (d, 0.5H, *J* = 4 Hz, Ar–**H**), 8.56 (s, 0.75H, **H**–C=N), 8.58 (d, 1.5H, *J* = 8 Hz, Ar–**H**), 9.25 (d, 0.5H, *J* = 4 Hz, Ar–**H**), 9.34 (d, 1.5H, *J* = 8 Hz, Ar–**H**), 12.52 (s, 0.25H, CON**H**), 12.64 (s, 0.75H, CON**H**). ^13^C NMR (100 MHz, DMSO-*d*_6_): δ_C_ = 13.89 (**C**H_3_), 22.03, 25.36, 28.34, 28.64, 28.73, 28.87, 28.91, 30.49, 30.63, 31.24 (9×**C**H_2_), 60.95, 61.01 (N**C**H_2_), 115.73, 115.94, 116.16, 126.19, 127.10, 129.34, 129.43, 129.69, 129.78, 130.07, 130.25, 130.28, 145.08, 145.66, 147.25, 149.40, 149.66 (Ar–**C**), 158.70, 162.27, 164.74, 165.19 (**C**=N, **C**=O). ^11^B NMR (128 MHz, DMSO-*d*_6_): δ_B_ = − 1.30 to − 1.28 (m, 1B, **B**F_4_). ^19^F NMR (377 MHz, DMSO-*d*_6_): δ_F_ = (− 109.97 to − 109.89), (− 109.48 to − 109.40) (2m, 1F, Ar–**F**); − 148.36, − 148.30 (2d, 4F, B**F**_4_). MS (ES) *m/z *= 485.20 [M^+^].

##### *4*-*(2*-*(4*-*Fluorobenzylidene)hydrazinecarbonyl)*-*1*-*undecylpyridin*-*1*-*ium trifluoroacetate (****21****)*

It was obtained as colorless syrup. ^1^H NMR (400 MHz, DMSO-*d*_6_): δ_H_ = 0.83–0.87 (m, 3H, C**H**_3_), 1.24–1.30 (m, 16H, 8×C**H**_2_), 1.96–1.99 (m, 2H, NCH_2_C**H**_2_), 4.69 (dd, 2H, *J* = 4 Hz, 8 Hz, NC**H**_2_), 7.22 (t, 0.5H, *J* = 8 Hz, Ar–**H**), 7.36 (dd, 1.5H, *J* = 8 Hz, 12 Hz, Ar–**H**), 7.61 (dd, 0.5H, *J* = 4 Hz, 8 Hz, Ar–**H**), 7.87 (dd, 1.5H, *J* = 4 Hz, 8 Hz, Ar–**H**), 8.16 (s, 0.25H, **H**–C=N), 8.39 (d, 0.5H, *J* = 4 Hz, Ar–**H**), 8.51 (s, 0.75H, **H**–C=N), 8.54 (d, 1.5H, *J* = 8 Hz, Ar–**H**), 9.25 (d, 0.5H, *J* = 8 Hz, Ar–**H**), 9.32 (d, 1.5H, *J* = 4 Hz, Ar–**H**), 12.54 (bs, 1H, CON**H**). ^13^C NMR (100 MHz, DMSO-*d*_6_): δ_C_ = 13.89 (**C**H_3_), 22.03, 25.36, 28.33, 28.64, 28.73, 28.87, 28.91, 30.49, 30.63, 31.24 (9×**C**H_2_), 60.96, 60.99 (N**C**H_2_), 115.73, 115.93, 116.15, 126.12, 127.11, 129.34, 129.42, 129.67, 129.76, 130.05, 130.30, 130.33, 145.07, 145.63, 147.55, 149.38, 149.67 (Ar–**C**), 158.82, 162.25, 164.72, 165.20 (**C**=N, **C**=O). ^19^F NMR (377 MHz, DMSO-*d*_6_): δ_F_ = − 73.53 (s, 3F, C**F**_**3**_), (− 109.97 to − 109.89), (− 109.54 to − 109.46) (2 m, 1F, Ar–**F**). MS (ES) *m/z *= 511.30 [M^+^].

##### *1*-*Dodecyl*-*4*-*(2*-*(4*-*fluorobenzylidene) hydrazinecarbonyl)pyridin*-*1*-*ium hexafluorophosphate (****22****)*

It was obtained as yellow syrup. ^1^H NMR (400 MHz, DMSO-*d*_6_): δ_H_ = 0.83–0.87 (m, 3H, C**H**_3_), 1.24–1.30 (m, 18H, 9×C**H**_2_), 1.96–1.98 (m, 2H, NCH_2_C**H**_2_), 4.69 (dd, 2H, *J* = 4 Hz, 8 Hz, NC**H**_2_), 7.22 (t, 0.5H, *J* = 8 Hz, Ar–**H**), 7.37 (dd, 1.5H, *J* = 8 Hz, 12 Hz, Ar–**H**), 7.61 (dd, 0.5H, *J* = 4 Hz, 8 Hz, Ar–**H**), 7.89 (dd, 1.5H, *J* = 4 Hz, 8 Hz, Ar–**H**), 8.16 (s, 0.25H, **H**–C=N), 8.39 (d, 0.5H, *J* = 4 Hz, Ar–**H**), 8.51 (s, 0.75H, **H**–C=N), 8.53 (d, 1.5H, *J* = 4 Hz, Ar–**H**), 9.24 (d, 0.5H, *J* = 4 Hz, Ar–**H**), 9.33 (d, 1.5H, *J* = 8 Hz, Ar–**H**), 12.47 (bs, 1H, CON**H**). ^13^C NMR (100 MHz, DMSO-*d*_6_): δ_C_ = 13.89 (**C**H_3_), 22.03, 25.36, 28.33, 28.65, 28.73, 28.86, 28.95, 30.48, 30.62, 31.24 (10×**C**H_2_), 60.96, 61.03 (N**C**H_2_), 115.73, 115.95, 116.17, 126.14, 127.11, 129.34, 129.43, 129.72, 129.81, 130.04, 130.25, 145.09, 145.68, 147.34, 149.38, 149.66 (Ar–**C**), 158.74, 162.29, 164.76, 165.20 (**C**=N, **C**=O). ^31^P NMR (162 MHz, DMSO-*d*_6_): δ_P_ = − 157.37 to − 131.02 (m, 1P, **P**F_6_). ^19^F NMR (377 MHz, DMSO-*d*_6_): δ_F_ = − 69.25 (d, 6F, P**F**_**6**_), (− 109.95 to − 109.88), (− 109.44 to − 109.36) (2m, 1F, Ar–**F**). MS (ES) *m/z *= 557.30 [M^+^].

##### *1*-*Dodecyl*-*4*-*(2*-*(4*-*fluorobenzylidene) hydrazinecarbonyl)pyridin*-*1*-*ium tetrafluoroborate (****23****)*

It was obtained as yellow syrup. ^1^H NMR (400 MHz, DMSO-*d*_6_): δ_H_ = 0.83 (t, 3H, *J* = 8 Hz, C**H**_3_), 1.24–1.30 (m, 18H, 9×C**H**_2_), 1.96–1.98 (m, 2H, NCH_2_C**H**_2_), 4.68 (t, 2H, *J* = 8 Hz, NC**H**_2_), 7.22 (t, 0.5H, *J* = 8 Hz, Ar–**H**), 7.34 (t, 1.5H, *J* = 8 Hz, Ar–**H**), 7.62 (dd, 0.5H, *J* = 4 Hz, 8 Hz, Ar–**H**), 7.88 (dd, 1.5H, *J* = 4 Hz, 8 Hz, Ar–**H**), 8.16 (s, 0.25H, **H**–C=N), 8.39 (d, 0.5H, *J* = 4 Hz, Ar–**H**), 8.52 (s, 0.75H, **H**–C=N), 8.54 (d, 1.5H, *J* = 8 Hz, Ar–**H**), 9.25 (d, 0.5H, *J* = 8 Hz, Ar–**H**), 9.33 (d, 1.5H, *J* = 4 Hz, Ar–**H**), 12.48 (bs, 1H, CON**H**). ^13^C NMR (100 MHz, DMSO-*d*_6_): δ_C_ = 13.89 (**C**H_3_), 22.03, 25.36, 28.33, 28.65, 28.74, 28.86, 28.95, 30.48, 30.62, 31.24 (10×**C**H_2_), 60.96, 61.03 (N**C**H_2_), 115.73, 115.94, 116.16, 126.15, 127.11, 129.34, 129.43, 129.72, 129.80, 130.22, 130.25, 145.08, 145.69, 147.32, 149.38, 149.66 (Ar–**C**), 158.73, 162.29, 164.76, 165.19 (**C**=N, **C**=O). ^11^B NMR (128 MHz, DMSO-*d*_6_): δ_B_ = − 1.31 to − 1.28 (m, 1B, **B**F_4_). ^19^F NMR (377 MHz, DMSO-*d*_6_): δ_F_ = (− 109.96 to − 109.88), (− 109.45 to − 109.37) (2m, 1F, Ar–**F**); − 148.36, − 148.30 (2d, 4F, B**F**_4_). MS (ES) *m/z *= 499.20 [M^+^].

##### *1*-*Dodecyl*-*4*-*(2*-*(4*-*fluorobenzylidene) hydrazinecarbonyl)pyridin*-*1*-*ium trifluoroacetate (****24****)*

It was obtained as colorless syrup. ^1^H NMR (400 MHz, DMSO-*d*_6_): δ_H_ = 0.85 (t, 3H, *J* = 8 Hz, C**H**_3_), 1.24–1.30 (m, 18H, 9×C**H**_2_), 1.96–1.98 (m, 2H, NCH_2_C**H**_2_), 4.68 (t, 2H, *J* = 8 Hz, NC**H**_2_), 7.22 (t, 0.5H, *J* = 8 Hz, Ar–**H**), 7.34 (t, 1.5H, *J* = 8 Hz, Ar–**H**), 7.61 (dd, 0.5H, *J* = 4 Hz, 8 Hz, Ar–**H**), 7.88 (dd, 1.5H, *J* = 4 Hz, 8 Hz, Ar–**H**), 8.16 (s, 0.25H, **H**–C=N), 8.39 (d, 0.5H, *J* = 4 Hz, Ar–**H**), 8.53 (s, 0.75H, **H**–C=N), 8.54 (d, 1.5H, *J* = 4 Hz, Ar–**H**), 9.25 (d, 0.5H, *J* = 8 Hz, Ar–**H**), 9.33 (d, 1.5H, *J* = 4 Hz, Ar–**H**), 12.51 (bs, 1H, CON**H**). ^13^C NMR (100 MHz, DMSO-*d*_6_): δ_C_ = 13.89 (**C**H_3_), 22.03, 25.36, 28.33, 28.65, 28.73, 28.86, 28.95, 30.48, 30.63, 31.24 (10×**C**H_2_), 60.96, 61.01 (N**C**H_2_), 115.73, 115.94, 116.16, 126.14, 127.11, 129.34, 129.43, 129.70, 129.79, 130.25, 130.28, 145.08, 145.67, 147.37, 149.39, 149.66 (Ar–**C**), 158.75, 162.27, 164.75, 165.19 (**C**=N, **C**=O). ^19^F NMR (377 MHz, DMSO-*d*_6_): δ_F_ = − 73.53 (s, 3F, C**F**_**3**_), (− 109.97 to − 109.89), (− 109.48 to − 109.40) (2m, 1F, Ar–**F**). MS (ES) *m/z *= 525.20 [M^+^].

##### *4*-*(2*-*(4*-*Fluorobenzylidene)hydrazinecarbonyl)*-*1*-*tetradecylpyridin*-*1*-*ium hexafluorophosphte (****25****)*

It was obtained as yellow syrup. ^1^H NMR (400 MHz, DMSO-*d*_6_): δ_H_ = 0.83–0.87 (m, 3H, C**H**_3_), 1.24–1.30 (m, 22H, 11×C**H**_2_), 1.96–1.99 (m, 2H, NCH_2_C**H**_2_), 4.68 (t, 2H, *J* = 8 Hz, NC**H**_2_), 7.22 (t, 0.5H, *J* = 8 Hz, Ar–**H**), 7.34 (t, 1.5H, *J* = 8 Hz, Ar–**H**), 7.61 (dd, 0.5H, *J* = 4 Hz, 8 Hz, Ar–**H**), 7.89 (dd, 1.5H, *J* = 4 Hz, 8 Hz, Ar–**H**), 8.16 (s, 0.25H, **H**–C=N), 8.39 (d, 0.5H, *J* = 4 Hz, Ar–**H**), 8.50 (s, 0.75H, **H**–C=N), 8.53 (d, 1.5H, *J* = 8 Hz, Ar–**H**), 9.24 (d, 0.5H, *J* = 8 Hz, Ar–**H**), 9.33 (d, 1.5H, *J* = 8 Hz, Ar–**H**), 12.44 (s, 0.75H, CON**H**), 12.49 (s, 0.25H, CON**H**). ^13^C NMR (100 MHz, DMSO-*d*_6_): δ_C_ = 13.88 (**C**H_3_), 22.03, 25.36, 28.33, 28.65, 28.73, 28.86, 28.95, 28.99, 30.48, 30.62, 31.24, 32.84 (12×**C**H_2_), 60.97, 61.04 (N**C**H_2_), 115.73, 115.94, 116.16, 126.14, 127.11, 129.34, 129.43, 129.72, 129.81, 130.07, 130.21, 130.24, 145.07, 145.68, 147.32, 149.38 (Ar–**C**), 158.73, 162.29, 164.77, 165.19 (**C**=N, **C**=O). ^31^P NMR (162 MHz, DMSO-*d*_6_): δ_P_ = − 152.97 to − 135.41 (m, 1P, **P**F_6_). ^19^F NMR (377 MHz, DMSO-*d*_6_): δ_F_ = − 69.26 (d, 6F, P**F**_**6**_), (− 109.96 to − 109.89), (− 109.44 to − 109.36) (2m, 1F, Ar–**F**). MS (ES) *m/z *= 585.50 [M^+^].

##### *4*-*(2*-*(4*-*Fluorobenzylidene)hydrazinecarbonyl)*-*1*-*tetradecylpyridin*-*1*-*ium tetrafluoroborate (****26****)*

It was obtained as yellow syrup. ^1^H NMR (400 MHz, DMSO-*d*_6_): δ_H_ = 0.85 (t, 3H, *J* = 8 Hz, C**H**_3_), 1.24–1.30 (m, 22H, 11×C**H**_2_), 1.96–1.99 (m, 2H, NCH_2_C**H**_2_), 4.68 (t, 2H, *J* = 8 Hz, NC**H**_2_), 7.22 (t, 0.5H, *J* = 8 Hz, Ar–**H**), 7.34 (t, 1.5H, *J* = 8 Hz, Ar–**H**), 7.62 (dd, 0.5H, *J* = 4 Hz, 8 Hz, Ar–**H**), 7.89 (dd, 1.5H, *J* = 4 Hz, 8 Hz, Ar–**H**), 8.16 (s, 0.25H, **H**–C=N), 8.39 (d, 0.5H, *J* = 4 Hz, Ar–**H**), 8.50 (s, 0.75H, **H**–C=N), 8.53 (d, 1.5H, *J* = 8 Hz, Ar–**H**), 9.25 (d, 0.5H, *J* = 8 Hz, Ar–**H**), 9.33 (d, 1.5H, *J* = 4 Hz, Ar–**H**), 12.44 (s, 0.75H, CON**H**), 12.49 (s, 0.25H, CON**H**). ^13^C NMR (100 MHz, DMSO-*d*_6_): δ_C_ = 13.88 (**C**H_3_), 22.03, 25.36, 28.34, 28.65, 28.74, 28.87, 28.96, 28.99, 30.48, 30.62, 31.24 (12×**C**H_2_), 60.96, 61.03 (N**C**H_2_), 115.73, 115.94, 116.16, 126.14, 127.11, 129.34, 129.43, 129.72, 129.81, 130.07, 130.21, 130.24, 145.08, 145.69, 147.32, 149.38, 149.66 (Ar–**C**), 158.72, 162.29, 164.77, 165.19 (**C**=N, **C**=O). ^11^B NMR (128 MHz, DMSO-*d*_6_): δ_B_ = − 1.30 to − 1.29 (m, 1B, **B**F_4_). ^19^F NMR (377 MHz, DMSO-*d*_6_): δ_F_ = (− 109.97 to − 109.89), (− 109.45 to − 109.37) (2m, 1F, Ar–**F**); − 148.37, − 148.32 (2d, 4F, B**F**_4_). MS (ES) *m/z *= 527.40 [M^+^].

##### *4*-*(2*-*(4*-*Fluorobenzylidene)hydrazinecarbonyl)*-*1*-*tetradecylpyridin*-*1*-*ium trifluoroacetate (****27****)*

It was obtained as colorless syrup. ^1^H NMR (400 MHz, DMSO-*d*_6_): δ_H_ = 0.85 (t, 3H, *J* = 8 Hz, C**H**_3_), 1.24–1.30 (m, 22H, 11×C**H**_2_), 1.96–1.98 (m, 2H, NCH_2_C**H**_2_), 4.68 (t, 2H, *J* = 8 Hz, NC**H**_2_), 7.22 (t, 0.5H, *J* = 8 Hz, Ar–**H**), 7.34 (t, 1.5H, *J* = 8 Hz, Ar–**H**), 7.61 (dd, 0.5H, *J* = 4 Hz, 8 Hz, Ar–**H**), 7.88 (dd, 1.5H, *J* = 4 Hz, 8 Hz, Ar–**H**), 8.16 (s, 0.25H, **H**–C=N), 8.39 (d, 0.5H, *J* = 4 Hz, Ar–**H**), 8.51 (s, 0.75H, **H**–C=N), 8.53 (d, 1.5H, *J* = 4 Hz, Ar–**H**), 9.25 (d, 0.5H, *J* = 8 Hz, Ar–**H**), 9.33 (d, 1.5H, *J* = 4 Hz, Ar–**H**), 12.47 (s, 0.75H, CON**H**), 12.49 (s, 0.25H, CON**H**). ^13^C NMR (100 MHz, DMSO-*d*_6_): δ_C_ = 13.88 (**C**H_3_), 22.03, 25.36, 28.33, 28.65, 28.74, 28.86, 28.95, 28.99, 30.49, 30.62, 31.24 (12×**C**H_2_), 60.95, 61.03 (N**C**H_2_), 115.72, 115.94, 116.16, 126.14, 127.11, 129.34, 129.43, 129.71, 129.80, 130.22, 130.25, 145.08, 145.69, 147.32, 149.38, 149.66 (Ar–**C**), 158.73, 162.29, 164.76, 165.19 (**C**=N, **C**=O). ^19^F NMR (377 MHz, DMSO-*d*_6_): δ_F_ = − 73.55 (s, 3F, C**F**_**3**_), (− 109.97 to − 109.89), (− 109.45 to − 109.38) (2m, 1F, Ar–**F**). MS (ES) *m/z *= 553.30 [M^+^].

##### *4*-*(2*-*(4*-*Fluorobenzylidene)hydrazinecarbonyl)*-*1*-*hexadecylpyridin*-*1*-*ium hexaflurophosphate (****28****)*

It was obtained as yellow syrup. ^1^H NMR (400 MHz, DMSO-*d*_6_): δ_H_ = 0.83–0.88 (m, 3H, C**H**_3_), 1.23–1.30 (m, 26H, 13×C**H**_2_), 1.96–2.00 (m, 2H, NCH_2_C**H**_2_), 4.68 (t, 2H, *J* = 8 Hz, NC**H**_2_), 7.24 (dd, 0.5H, *J* = 8 Hz, 12 Hz, Ar–**H**), 7.34 (t, 1.5H, *J* = 8 Hz, Ar–**H**), 7.62 (dd, 0.5H, *J* = 4 Hz, 8 Hz, Ar–**H**), 7.89 (dd, 1.5H, *J* = 4 Hz, 8 Hz, Ar–**H**), 8.16 (s, 0.25H, **H**–C=N), 8.39 (d, 0.5H, *J* = 4 Hz, Ar–**H**), 8.51 (s, 0.75H, **H**–C=N), 8.53 (d, 1.5H, *J* = 4 Hz, Ar–**H**), 9.25 (d, 0.5H, *J* = 8 Hz, Ar–**H**), 9.33 (d, 1.5H, *J* = 4 Hz, Ar–**H**), 12.44 (s, 0.75H, CON**H**), 12.49 (s, 0.25H, CON**H**). ^13^C NMR (100 MHz, DMSO-*d*_6_): δ_C_ = 13.88 (**C**H_3_), 22.03, 25.36, 28.34, 28.64, 28.74, 28.87, 28.96, 29.00, 30.49, 30.62, 31.24 (14×**C**H_2_), 60.96, 61.03 (N**C**H_2_), 115.72, 115.94, 116.15, 126.13, 127.11, 129.34, 129.43, 129.72, 129.81, 130.21, 130.24, 145.07, 145.69, 147.32, 149.37, 149.65 (Ar–**C**), 158.71, 162.29, 164.76, 165.18 (**C**=N, **C**=O). ^31^P NMR (162 MHz, DMSO-*d*_6_): δ_P_ = − 152.97 to − 135.41 (m, 1P, **P**F_6_). ^19^F NMR (377 MHz, DMSO-*d*_6_): δ_F_ = − 69.26 (d, 6F, P**F**_**6**_), (− 109.97 to − 109.89), (− 109.45 to − 109.37) (2m, 1F, Ar–**F**). MS (ES) *m/z *= 613.30 [M^+^].

##### *4*-*(2*-*(4*-*Fluorobenzylidene)hydrazinecarbonyl)*-*1*-*hexadecylpyridin*-*1*-*ium tetrafluoroborate (****29****)*

It was obtained as yellow syrup. ^1^H NMR (400 MHz, DMSO-*d*_6_): δ_H_ = 0.83–0.87 (m, 3H, C**H**_3_), 1.23–1.30 (m, 26H, 13×C**H**_2_), 1.94–2.00 (m, 2H, NCH_2_C**H**_2_), 4.70 (dd, 2H, *J* = 4 Hz, 8 Hz, NC**H**_2_), 7.24 (dd, 0.5H, *J* = 8 Hz, 12 Hz, Ar–**H**), 7.34 (t, 1.5H, *J* = 8 Hz, Ar–**H**), 7.62 (dd, 0.5H, *J* = 4 Hz, 8 Hz, Ar–**H**), 7.88 (dd, 1.5H, *J* = 4 Hz, 8 Hz, Ar–**H**), 8.16 (s, 0.25H, **H**–C=N), 8.39 (d, 0.5H, *J* = 4 Hz, Ar–**H**), 8.51 (s, 0.75H, **H**–C=N), 8.53 (d, 1.5H, *J* = 4 Hz, Ar–**H**), 9.25 (d, 0.5H, *J* = 4 Hz, Ar–**H**), 9.34 (d, 1.5H, *J* = 4 Hz, Ar–**H**), 12.45 (s, 0.75H, CON**H**), 12.49 (s, 0.25H, CON**H**). ^13^C NMR (100 MHz, DMSO-*d*_6_): δ_C_ = 13.88 (**C**H_3_), 22.03, 25.36, 28.34, 28.65, 28.75, 28.87, 28.96, 29.00, 30.49, 30.63, 31.24 (14×**C**H_2_), 60.96, 61.03 (N**C**H_2_), 115.72, 115.93, 116.15, 126.13, 127.11, 129.35, 129.43, 129.72, 129.80, 130.04, 130.21, 130.24, 145.07, 145.69, 147.30, 149.37, 149.64 (Ar–**C**), 158.71, 162.28, 164.76, 165.17 (**C**=N, **C**=O). ^11^B NMR (128 MHz, DMSO-*d*_6_): δ_B_ = − 1.29 to − 1.28 (m, 1B, **B**F_4_). ^19^F NMR (377 MHz, DMSO-*d*_6_): δ_F_ = (− 109.97 to − 109.90), (− 109.46 to − 109.38) (2m, 1F, Ar–**F**); − 148.36, − 148.31 (2d, 4F, B**F**_4_). MS (ES) *m/z *= 555.35 [M^+^].

##### *4*-*(2*-*(4*-*Fluorobenzylidene)hydrazinecarbonyl)*-*1*-*hexadecylpyridin*-*1*-*ium trifluoroacetate (****30****)*

It was obtained as colorless syrup. ^1^H NMR (400 MHz, DMSO-*d*_6_): δ_H_ = 0.85 (t, 3H, *J* = 8 Hz, C**H**_3_), 1.23–1.30 (m, 26H, 13×C**H**_2_), 1.96–1.98 (m, 2H, NCH_2_C**H**_2_), 4.69 (dd, 2H, *J* = 4 Hz, 8 Hz, NC**H**_2_), 7.22 (t, 0.5H, *J* = 8 Hz, Ar–**H**), 7.34 (t, 1.5H, *J* = 8 Hz, Ar–**H**), 7.61 (dd, 0.5H, *J* = 4 Hz, 8 Hz, Ar–**H**), 7.88 (dd, 1.5H, *J* = 4 Hz, 8 Hz, Ar–**H**), 8.16 (s, 0.25H, **H**–C=N), 8.39 (d, 0.5H, *J* = 4 Hz, Ar–**H**), 8.52 (s, 0.75H, **H**–C=N), 8.54 (d, 1.5H, *J* = 8 Hz, Ar–**H**), 9.25 (d, 0.5H, *J* = 8 Hz, Ar–**H**), 9.33 (d, 1.5H, *J* = 8 Hz, Ar–**H**), 12.50 (s, 1H, CON**H**). ^13^C NMR (100 MHz, DMSO-*d*_6_): δ_C_ = 13.88 (**C**H_3_), 22.03, 25.35, 28.33, 28.64, 28.73, 28.86, 28.95, 29.00, 30.49, 30.62, 31.23 (14×**C**H_2_), 60.95, 61.02 (N**C**H_2_), 115.72, 115.94, 116.16, 126.14, 127.11, 129.33, 129.42, 129.71, 129.80, 130.08, 130.26, 145.08, 145.68, 147.33, 149.39 (Ar–**C**), 158.73, 162.29, 164.76, 165.19 (**C**=N, **C**=O). ^19^F NMR (377 MHz, DMSO-*d*_6_): δ_F_ = − 73.52 (s, 3F, C**F**_**3**_), (− 109.96 to − 109.88), (− 109.46 to − 109.38) (2m, 1F, Ar–**F**). MS (ES) *m/z *= 581.30 [M^+^].

##### *4*-*(2*-*(4*-*Fluorobenzylidene)hydrazinecarbonyl)*-*1*-*octadecylpyridin*-*1*-*ium hexafluorophosphate (****31****)*

It was obtained as yellow syrup. ^1^H NMR (400 MHz, CDCl_3_): δ_H_ = 0.82 (dd, 3H, *J* = 4 Hz, 8 Hz, C**H**_3_), 1.15–1.18 (m, 30H, 15×C**H**_2_), 1.94–1.98 (m, 2H, NCH_2_C**H**_2_), 4.72 (t, 2H, *J* = 8 Hz, NC**H**_2_), 6.95 (t, 2H, *J* = 8 Hz, Ar–**H**), 7.67 (dd, 2H, *J* = 4 Hz, 8 Hz, Ar–**H**), 8.82 (d, 2H, *J* = 4 Hz, Ar–**H**), 9.01 (s, 1H, **H**–C=N), 9.08 (d, 2H, *J* = 8 Hz, Ar–**H**), 12.14 (bs, 1H, CON**H**). ^13^C NMR (100 MHz, CDCl_3_): δ_C_ = 14.08 (**C**H_3_), 22.66, 26.09, 28.97, 29.33, 29.49, 29.59, 29.64, 29.68, 31.64, 31.90 (16×**C**H_2_), 62.69 (N**C**H_2_), 115.87, 116.09, 127.71, 129.45, 130.09, 130.18, 144.87, 147.76, 151.75 (Ar–**C**), 158.62, 163.23, 165.74 (**C**=N, **C**=O). ^31^P NMR (162 MHz, CDCl_3_): δ_P_ = − 153.38 to − 135.76 (m, 1P, **P**F_6_). ^19^F NMR (377 MHz, CDCl_3_): δ_F_ = − 70.39 (d, 6F, P**F**_**6**_), (− 107.98 to − 107.89), (− 107.72 to − 107.65) (2m, 1F, Ar–**F**). MS (ES) *m/z *= 641.55 [M^+^].

##### *4*-*(2*-*(4*-*Fluorobenzylidene)hydrazinecarbonyl)*-*1*-*octadecylpyridin*-*1*-*ium tetrafluoroborate (****32****)*

It was obtained as yellow syrup. ^1^H NMR (400 MHz, CDCl_3_): δ_H_ = 0.82 (dd, 3H, *J* = 4 Hz, 8 Hz, C**H**_3_), 1.16–1.20 (m, 30H, 15×C**H**_2_), 1.94–1.98 (m, 2H, NCH_2_C**H**_2_), 4.73 (t, 2H, *J* = 8 Hz, NC**H**_2_), 6.99 (dd, 2H, *J* = 8 Hz, 12 Hz, Ar–**H**), 7.69 (dd, 2H, *J* = 4 Hz, 8 Hz, Ar–**H**), 8.83 (d, 2H, *J* = 8 Hz, Ar–**H**), 9.00 (s, 1H, **H**–C=N), 9.06 (d, 2H, *J* = 4 Hz, Ar–**H**), 12.11 (bs, 1H, CON**H**). ^13^C NMR (100 MHz, CDCl_3_): δ_C_ = 14.08 (**C**H_3_), 22.66, 26.10, 28.97, 29.33, 29.48, 29.57, 29.63, 29.68, 31.66, 31.90 (16×**C**H_2_), 62.64 (N**C**H_2_), 115.85, 116.07, 127.76, 129.46, 130.12, 130.21, 144.82, 147.96, 151.72 (Ar–**C**), 158.57, 163.25, 165.76 (**C**=N, **C**=O). ^11^B NMR (128 MHz, CDCl_3_): δ_B_ = − 1.29 to 1.28 (m, 1B, **B**F_4_). ^19^F NMR (377 MHz, CDCl_3_): δ_F_ = (− 107.98 to − 107.85) to (107.82 to − 107.75) (2m, 1F, Ar–**F**); − 149.14, 149.19 (2d, 4F, B**F**_4_). MS (ES) *m/z *= 583.45 [M^+^].

##### *4*-*(2*-*(4*-*Fluorobenzylidene)hydrazinecarbonyl)*-*1*-*octadecylpyridin*-*1*-*ium trifluoroacetate (****33****)*

It was obtained as colorless syrup. ^1^H NMR (400 MHz, CDCl_3_): δ_H_ = 0.82 (dd, 3H, *J* = 4 Hz, 8 Hz, C**H**_3_), 1.16–1.19 (m, 30H, 15×C**H**_2_), 1.95–1.99 (m, 2H, NCH_2_C**H**_2_), 4.75 (t, 2H, *J* = 8 Hz, NC**H**_2_), 6.96 (t, 2H, *J* = 8 Hz, Ar–**H**), 7.68 (dd, 2H, *J* = 4 Hz, 8 Hz, Ar–**H**), 8.84 (d, 2H, *J* = 8 Hz, Ar–**H**), 8.94 (s, 1H, **H**–C=N), 9.12 (d, 2H, *J* = 4 Hz, Ar–**H**), 12.46 (bs, 1H, CON**H**). ^13^C NMR (100 MHz, CDCl_3_): δ_C_ = 14.07 (**C**H_3_), 22.66, 26.09, 28.96, 29.33, 29.47, 29.57, 29.63, 29.68, 31.66, 31.90 (16×**C**H_2_), 62.66 (N**C**H_2_), 115.85, 116.07, 127.72, 129.53, 130.09, 130.17, 144.87, 148.01, 151.77 (Ar–**C**), 158.62, 163.22, 165.73 (**C**=N, **C**=O). ^19^F NMR (377 MHz, CDCl_3_): δ_F_ = − 75.30 (s, 3F, C**F**_**3**_), (− 108.01 to − 107.94), (− 107.85 to − 107.78) (2m, 1F, Ar–**F**). MS (ES) *m/z *= 609.35 [M^+^].

### Biological studies

#### Antiproliferative activity

MCF-7, T47D, HeLa and Caco-II cell lines were cultivated in Dulbecco’s modified Eagles medium (DMEM, Biochrom, Berlin, Germany). Cell lines were maintained at 37 °C and all media were supplemented with 1% of 2 mM l-glutamine (Lonza), 10% fetal calf serum (Gibco, Paisley, UK), 50 IU/ml penicillin/streptomycin (Sigma, St. Louis, MO) and amphotericin B (Sigma, St. Louis, MO). Cells from passage number 10–16 were used. For the antiproliferative activity test, compounds under examination, dissolved in DMSO, were added to the culture medium and incubated for 48 h incubation period in an atmosphere of 5% CO_2_ and 95 relative humidity at 37 °C.

Cells were seeded at a density of 8 × 10^3^ cells per well in 96-well plates in appropriate medium. When the exposure period ends, Promega Cell Titer 96 Aqueous Non-Radioactive Cell Proliferation (MTS) assay was carried out according to the manufacturer’s protocol. Absorbance values of each well were determined with a microplate enzyme-linked immuno-assay (ELISA) reader equipped with a 492 nm filter. Survival rates of the controls were set to represent 100% viability. Untreated cultures were used as controls groups.

#### Caspase-3 enzyme activity

To assess changes in caspase-3 activity, the caspase-3 colorimetric assay kit (BioVision Research Products, Milpitas, CA) was used after treatment with 100 µM of each compound and incubation for 48 h. Briefly, apoptosis was provoked in treated cells before cells were collected by centrifugation at 1000 rpm for 10 min. Cells were lysed and supernatants were separated according to the manufacture’s protocol. Protein concentration in the supernatant was determined using the Bradford method. 50 µl of the reaction buffer, 200 µM of DEVD-pNA substrate were added to 50 µl supernatant in a 96-well plate and incubated at 37 °C for 2 h. After incubation, the plate was read under 405 nm wavelength using an ELISA reader (Tecan Group Ltd., Mannedorf, Switzerland).

### Computational methods

#### Preparation of protein structure

The crystal structure of apo PI3Kα (PDB ID: 2RD0) [(**2**)] was retrieved from the RCSB Protein Data Bank. The homology modeled structure of 2RD0 was adopted for this study [47]. The coordinates of wortmannin in 3HHM [48] were moved to 2RD0 and assigned as the ligand. Minimization of the protein side chains was applied to reduce the steric clashes recruiting MacroModel [20] module in MAESTRO. Further preparation of the coordinates was carried out using Protein Preparation [20] wizard in Schrödinger to maximize the H-bond interactions between residues.

#### Preparation of ligand structures

The synthesized compounds (ligands) were built based on the coordinates of wortmannin in 3 HHM. The ligands were built using MAESTRO [20] BUILD module and then subjected for energy minimization using OPLS2005 force field in MacroModel program.

#### Quantum–polarized ligand docking (QPLD)

QPLD [20, 45] (**3**, **4**) docking employed the combined QM/MM approach to determine ligand/protein complex formation. The Glide [49–51] docking was implemented in QPLD to generate a list of ligand docked poses that fit the protein binding site. The binding energy of the protein/newly generated ligand pose was derived using the molecular mechanical (MM) method for the protein coordinates while the quantum mechanical (QM) method was applied for ligand pose recruiting the QSite wizard in Schrödinger [45]. The Qsite program generated the atomic partial charges for the ligand pose within the protein environment. The ligand pose with QM-generated partial charges were redocked to the binding pocket using Glide [45] program with XP-scoring function. Specifically, the polarization effect of the protein binding pocket was accounted during the docking procedure. The ligand pose with the lowest root mean square deviation (RMSD) was investigated. The kinase binding domain was defined using the ligand as a centroid. The scaling of receptor Vander Waals for the non-polar atoms was set to 0.75.

## Conclusions

Novel cationic fluorinated pyridinium hydrazones tethering lipophilic side chain were designed and synthesized under both conventional and green ultrasound conditions. The synthesized compounds were assessed for their anticancer activities and the results revealed that adding to the length of the hydrophobic chain significantly enhances their anticancer activities. Considerable increase in caspase-3 activity was associated with the most potent compounds, namely **8**, **28**, **29** and **32** suggesting an apoptotic cellular death pathway. Molecular Docking studies employing QPLD approach against PI3Kα demonstrated that compounds **2**–**9** accommodate the kinase site and form H-bond with S774, K802, H917, and D933 (Additional file 1).

## Additional file

**Additional file 1.** Additional figures.
