# Supplementary material for: Novel amphiphilic pyridinium ionic liquids-supported Schiff bases: ultrasound assisted synthesis, molecular docking and anticancer evaluation
Source: Chem Cent J. 2018 Nov 22;12:118. doi: 10.1186/s13065-018-0489-z (PMC6768046; doi:10.1186/s13065-018-0489-z)
Supplement: Supplementary file 1 — Additional file 1. Additional figures. [file 13065_2018_489_MOESM1_ESM.pdf]

Novel Amphiphilic Pyridinium Ionic Liquids-Supported Schiff Bases. Ultrasound Assisted Synthesis, Molecular Docking and Anticancer Evaluation

Fawzia Faleh Al-Blewi[1], Nadjet Rezki[1,2,*], Salsabeel Abdullah Al-Sodies[1], Sanaa K. Bardaweel[3], Dima A. Sabbah[4], Mouslim Messali[1] and Mohamed Reda Aouad[1,*]

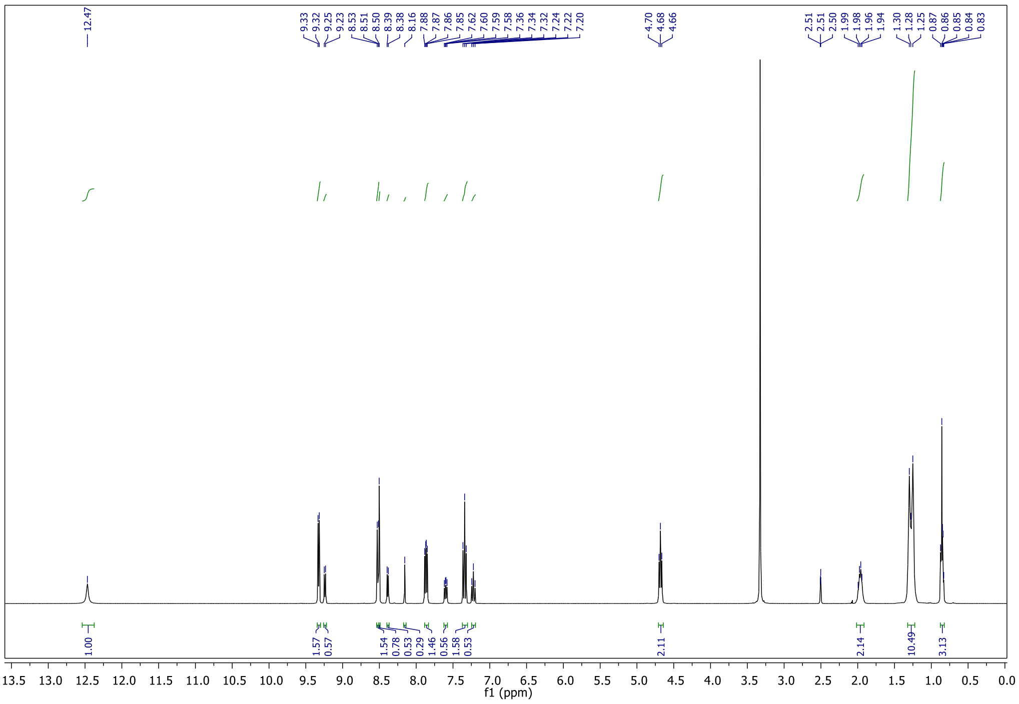

Figure S1. ^{1}H NMR of Compound **2**.

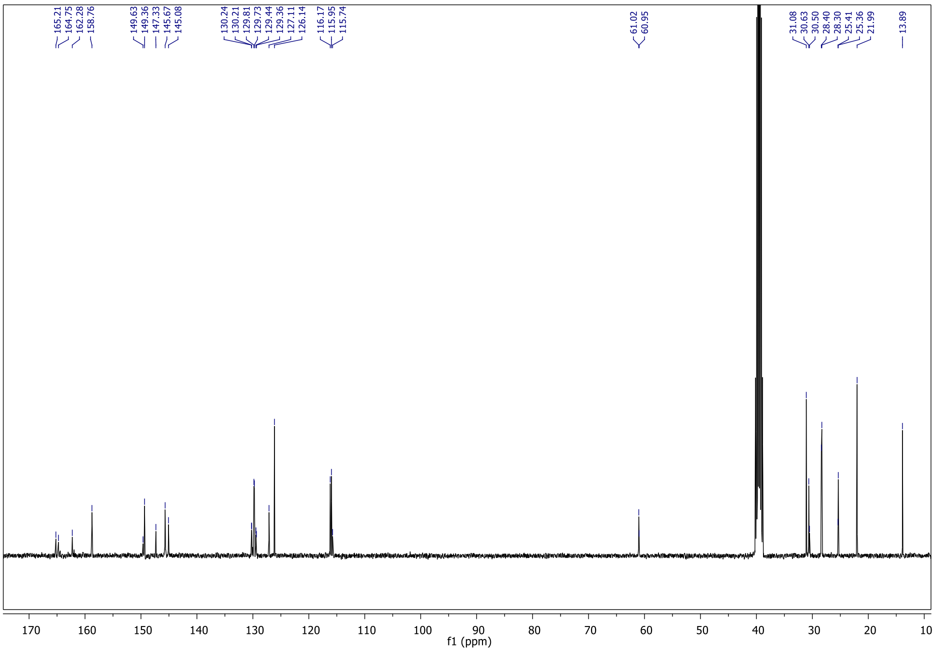

Figure S2. ^{13}C NMR of Compound 2

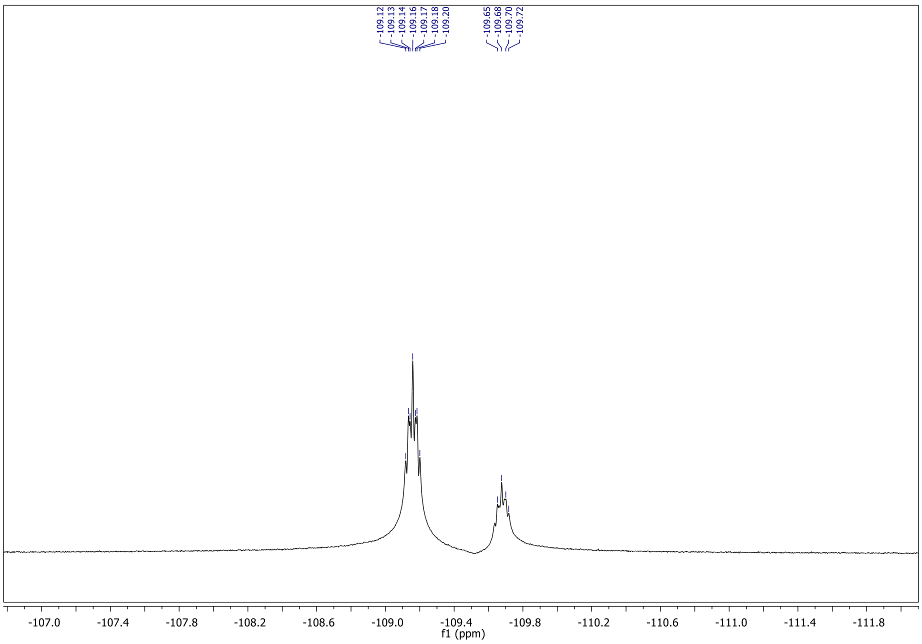

Figure S3. ^{19}FNMR of Compound 2

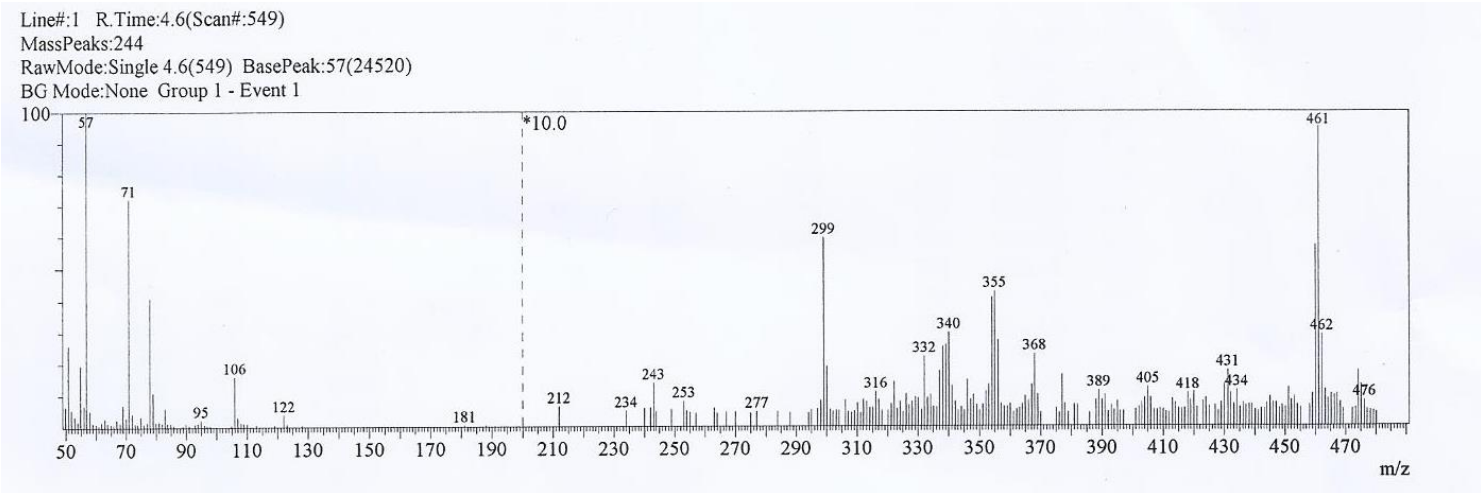

Figure S4. MS (ESI) of Compound 2

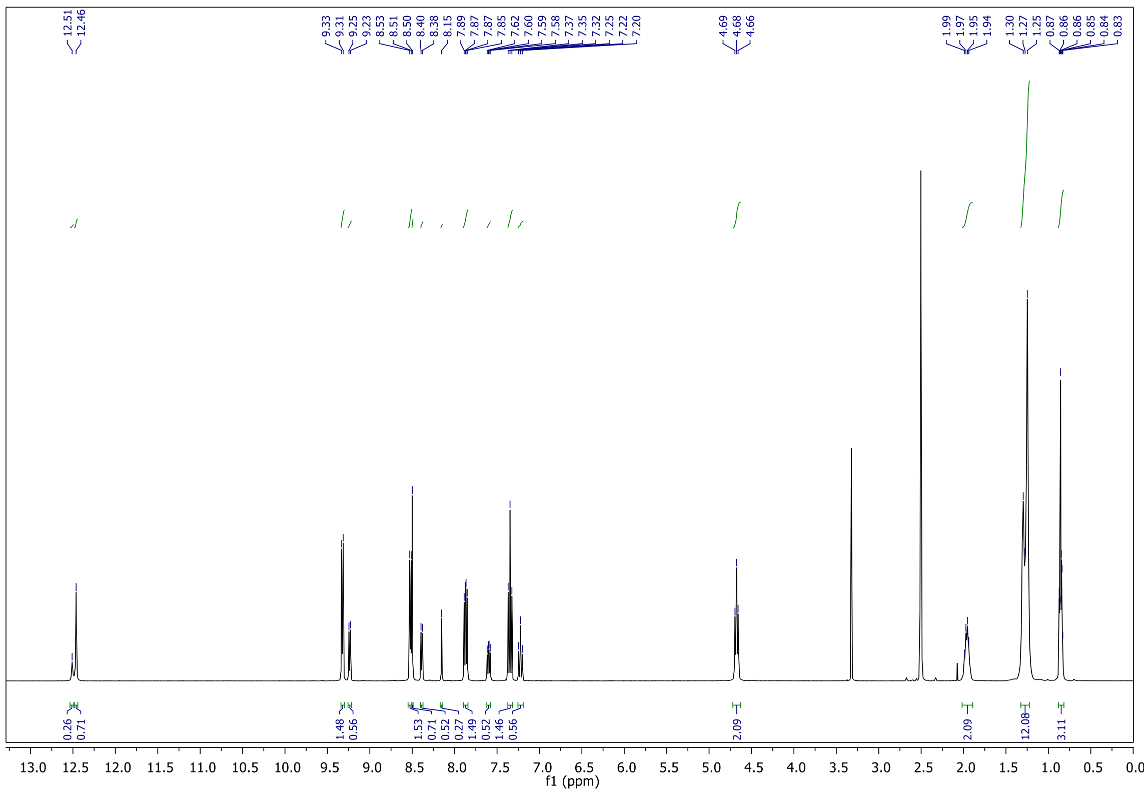

Figure S5. ^{1}HNMR of Compound 3

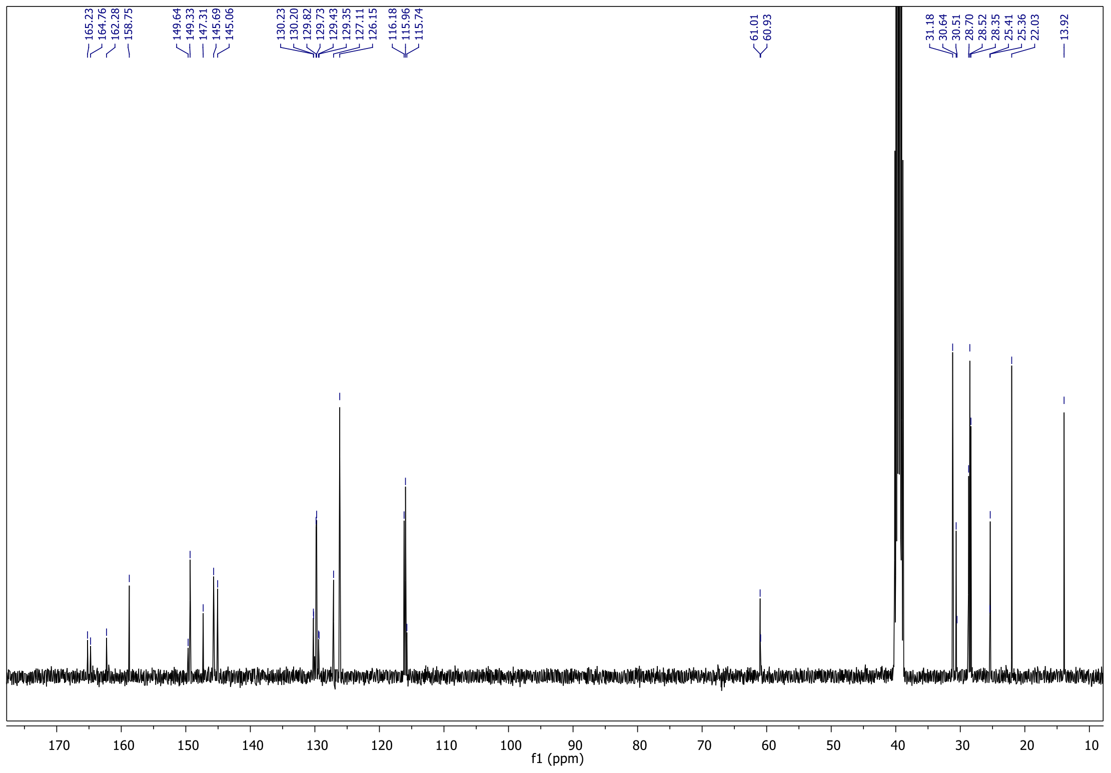

Figure S6. ^{13}CNMR of Compound 3

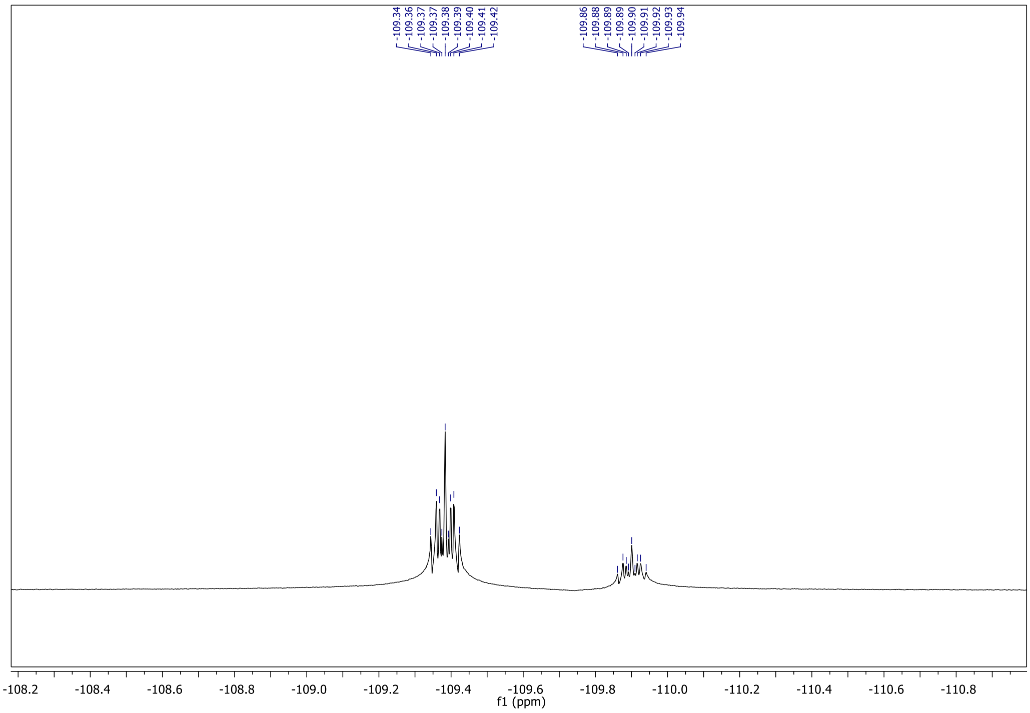

Figure S7. ^{19}FNMR of Compound 3

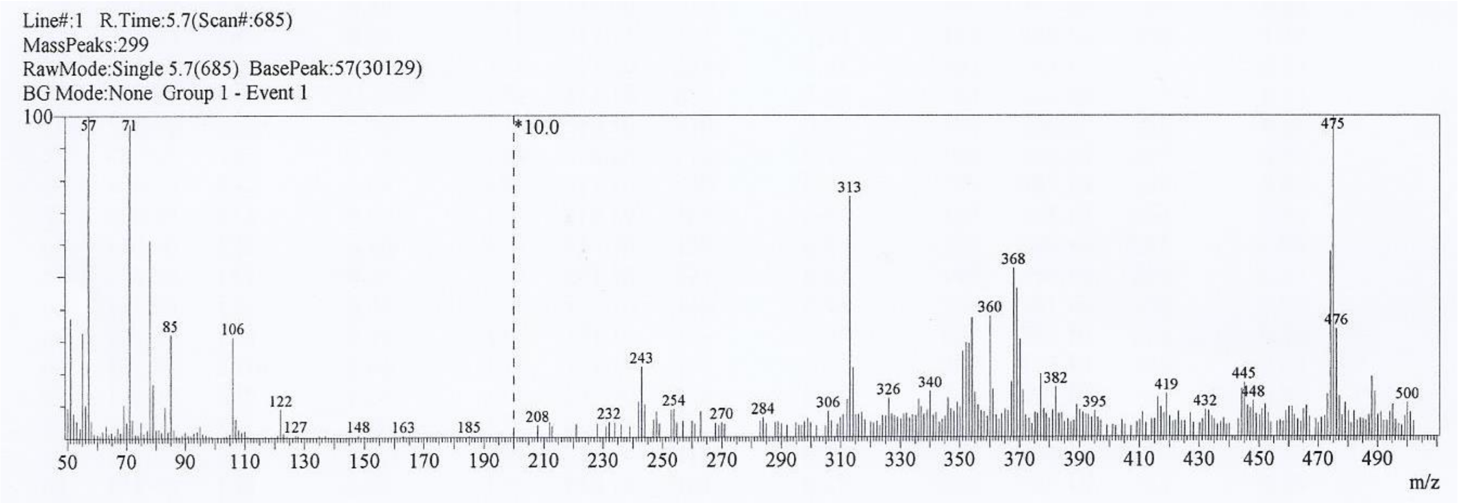

Figure S8. MS (ESI) of Compound 3

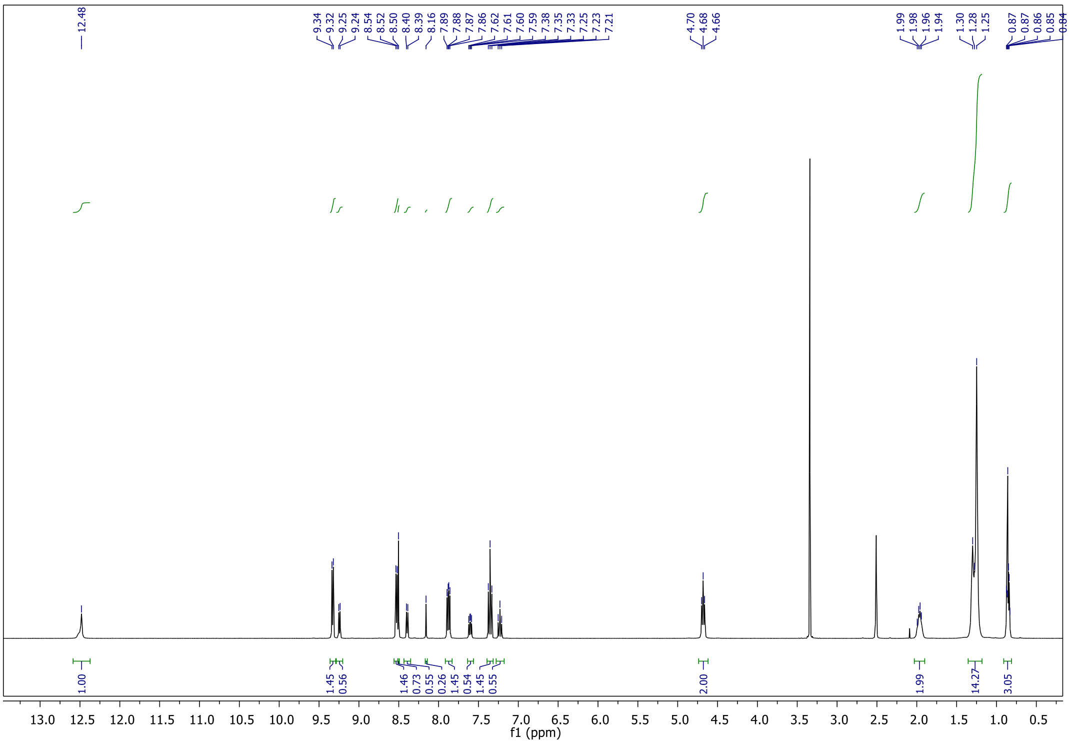

Figure S9. ^{1}HNMR of Compound 4

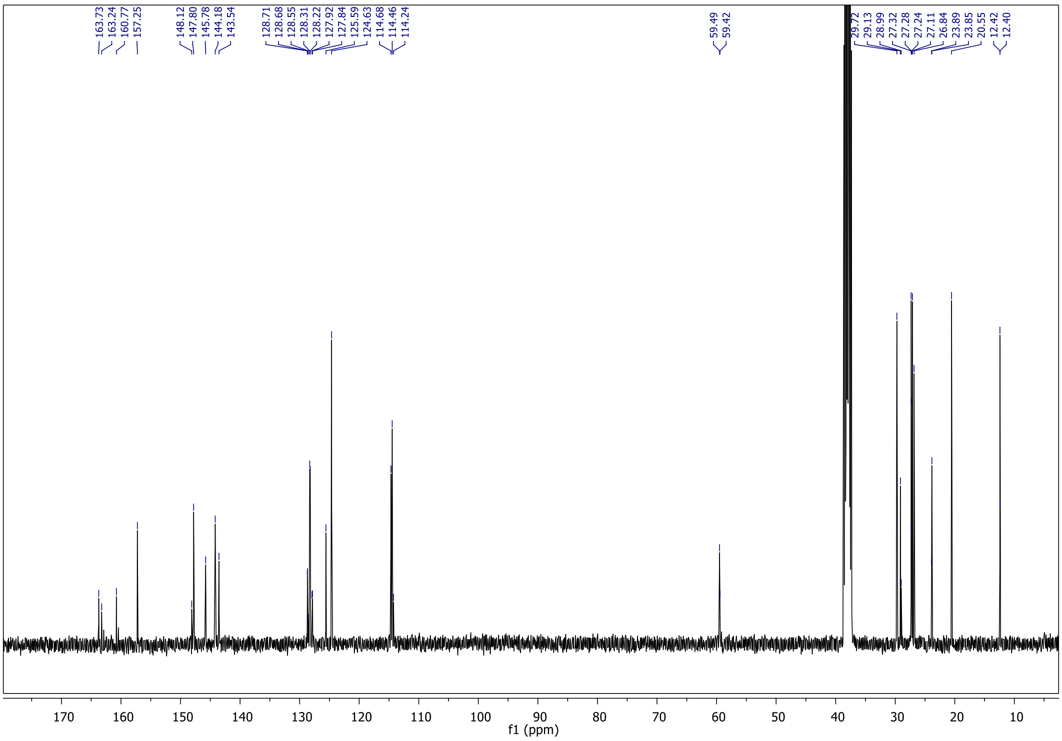

Figure S10. ^{13}CNMR of Compound 4

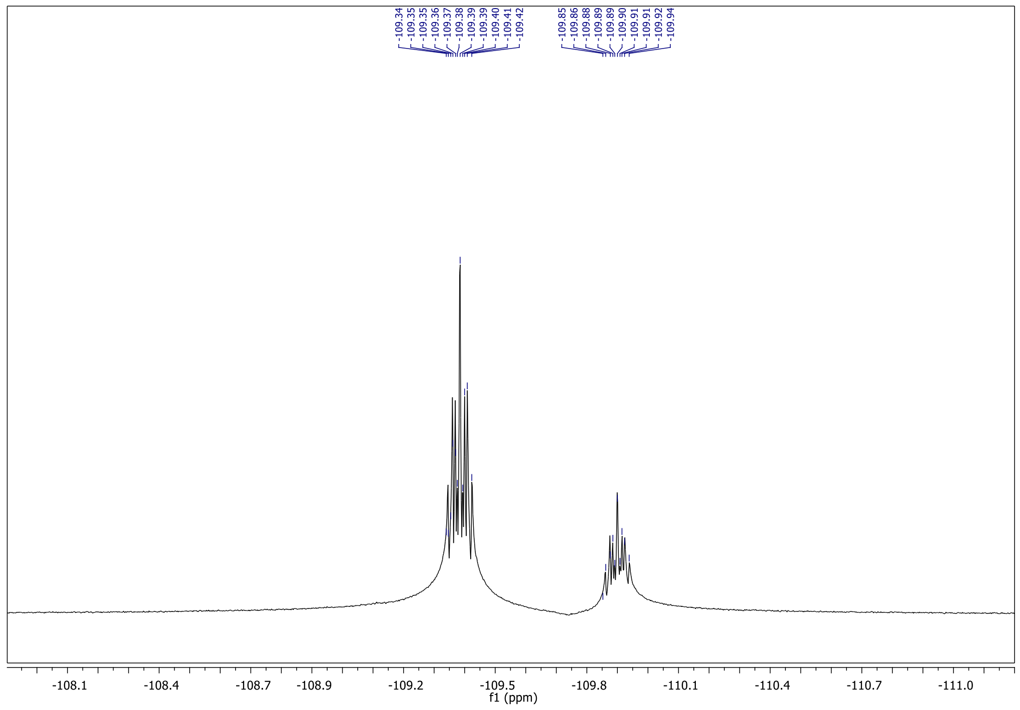

Figure S11. ^{19}FNMR of Compound 4

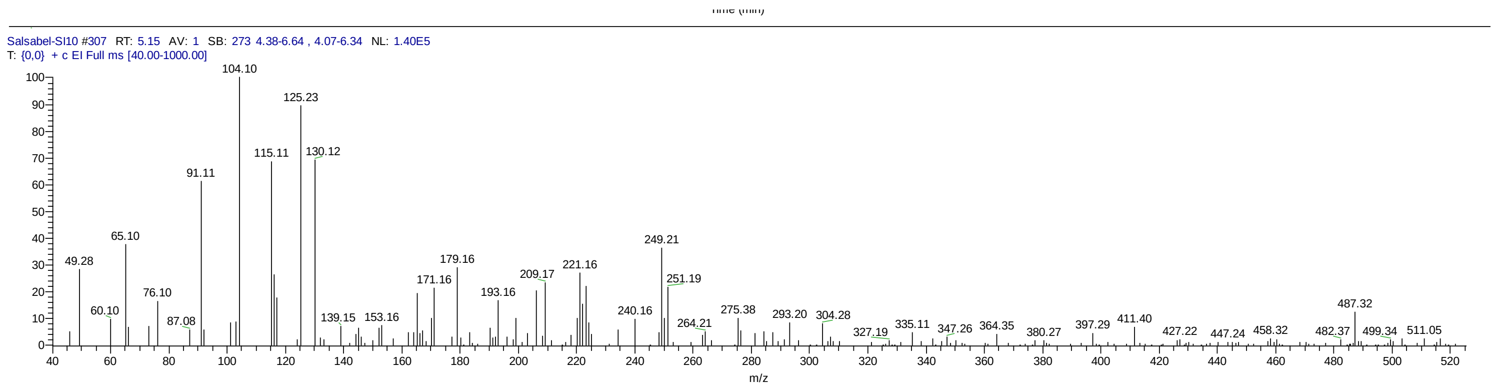

Figure S12. MS (ESI) of Compound 4

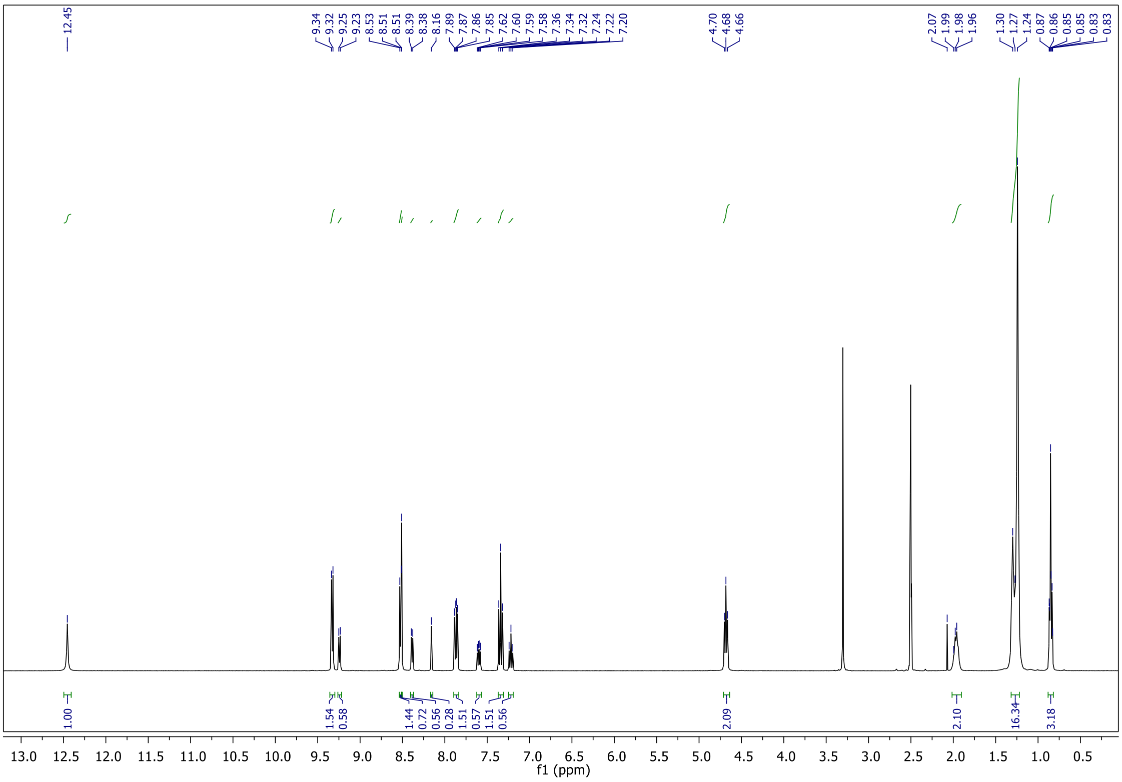

Figure S13. ^{1}HNMR of Compound 5

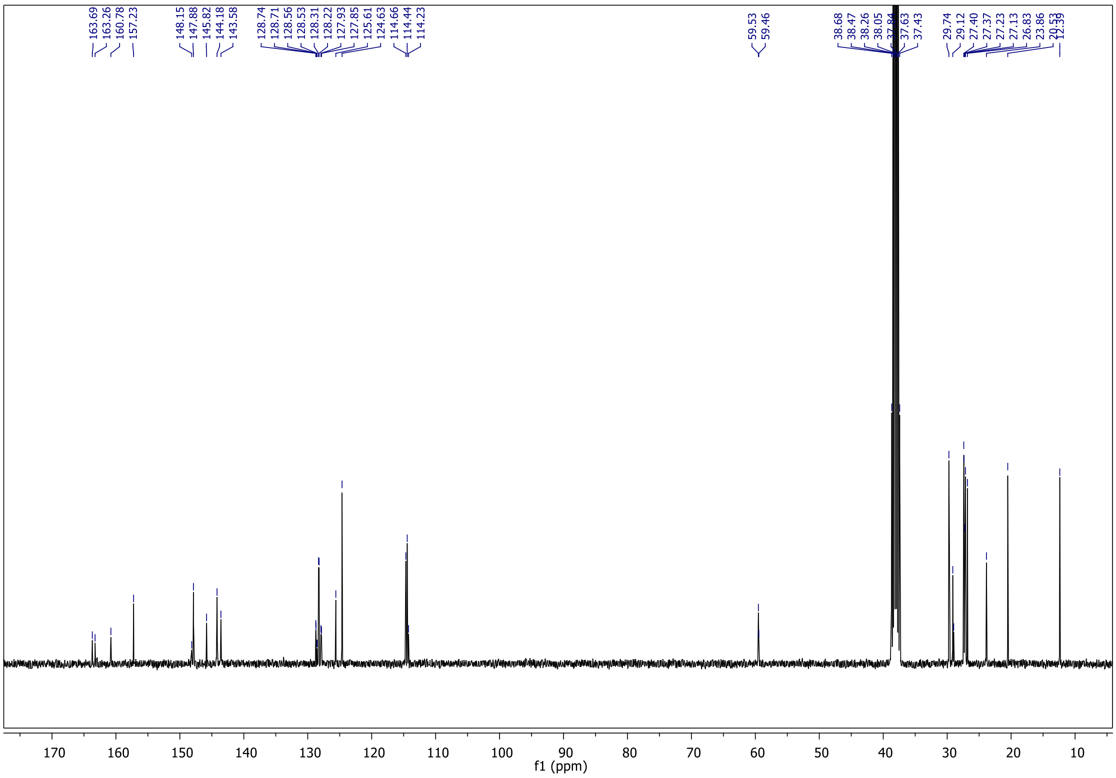

Figure S14. ^{13}CNMR of Compound 5

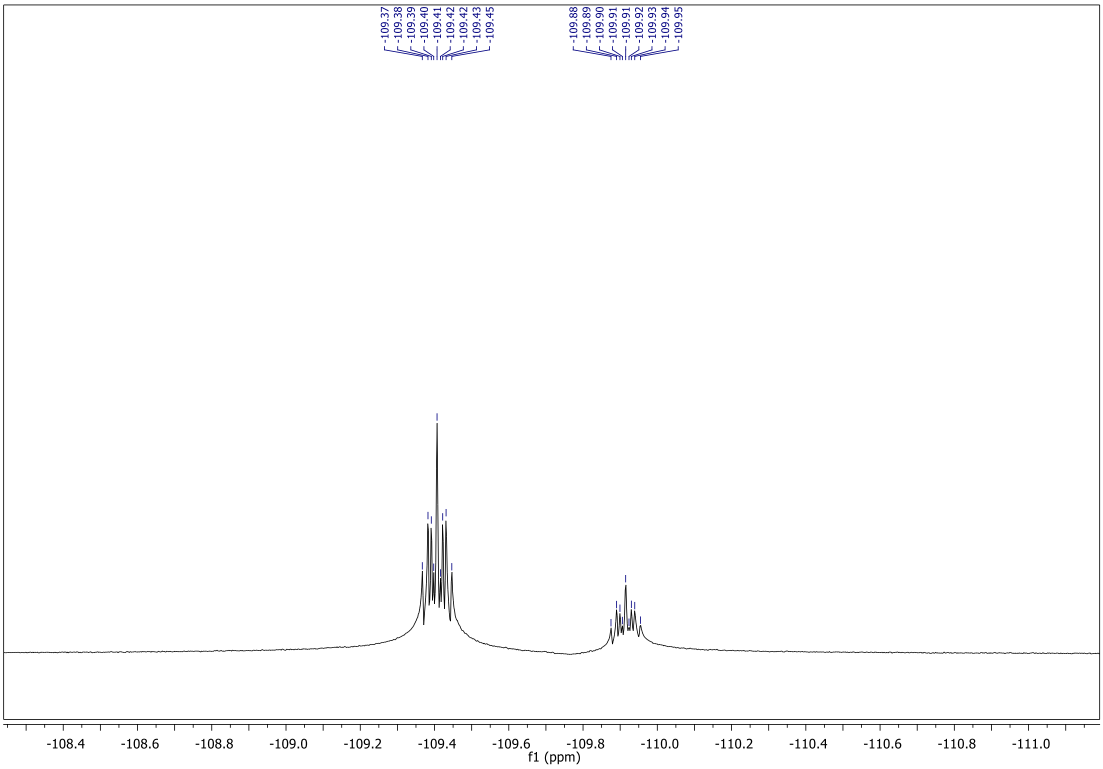

Figure S15. ^{19}FNMR of Compound 5

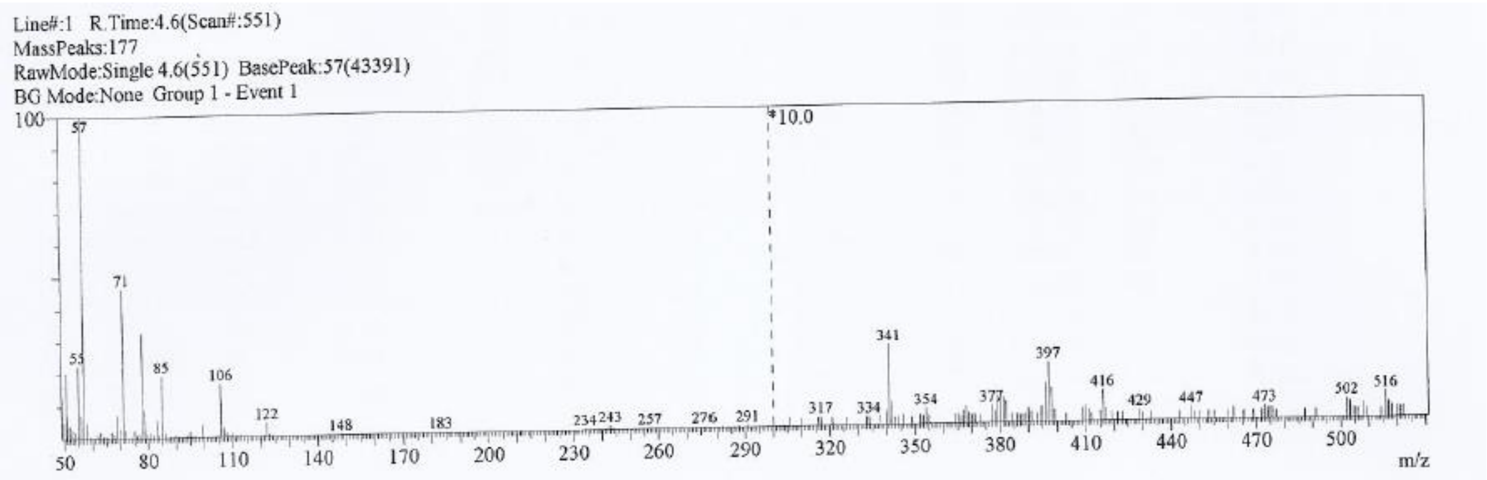

Figure S16. MS (ESI) of Compound 5

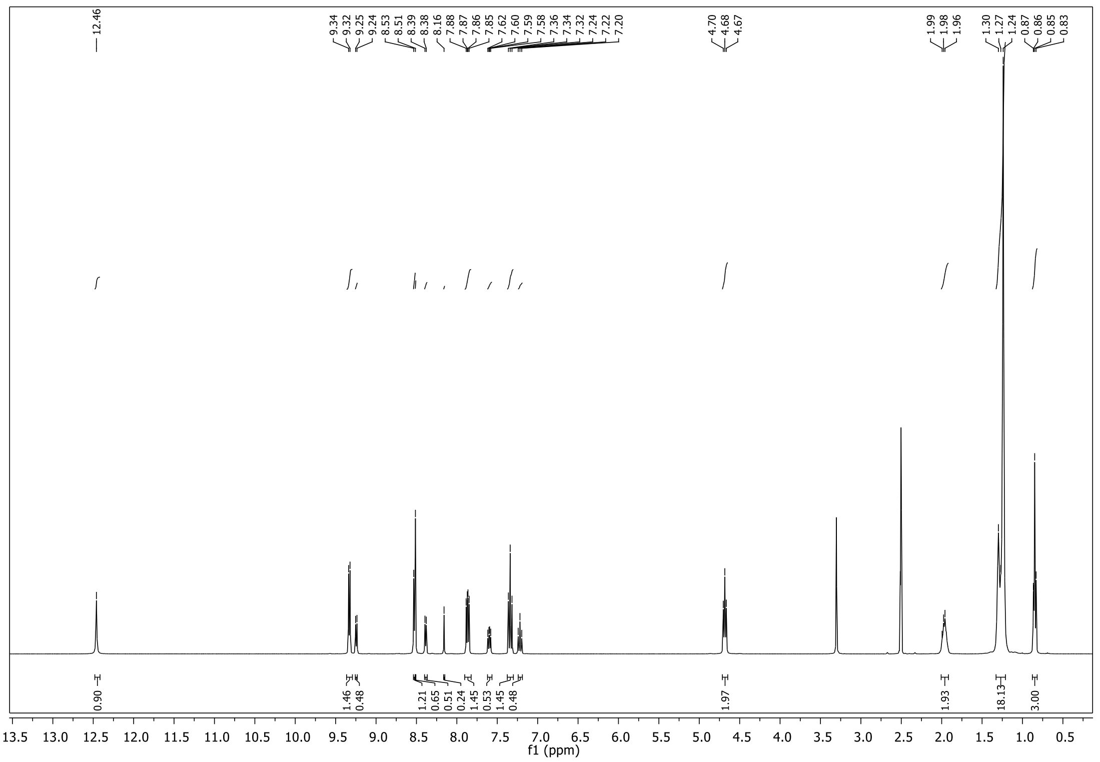

Figure S17. ^{1}HNMR of Compound 6

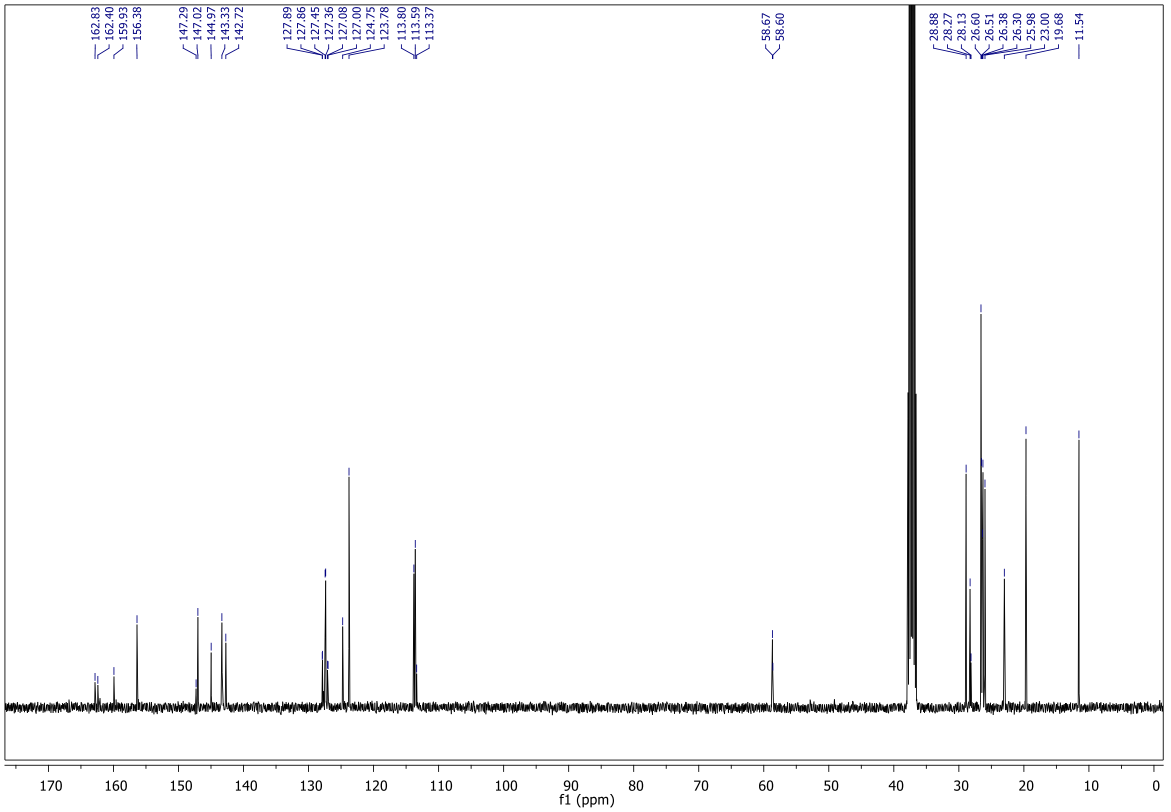

Figure S18. ^{13}CNMR of Compound 6

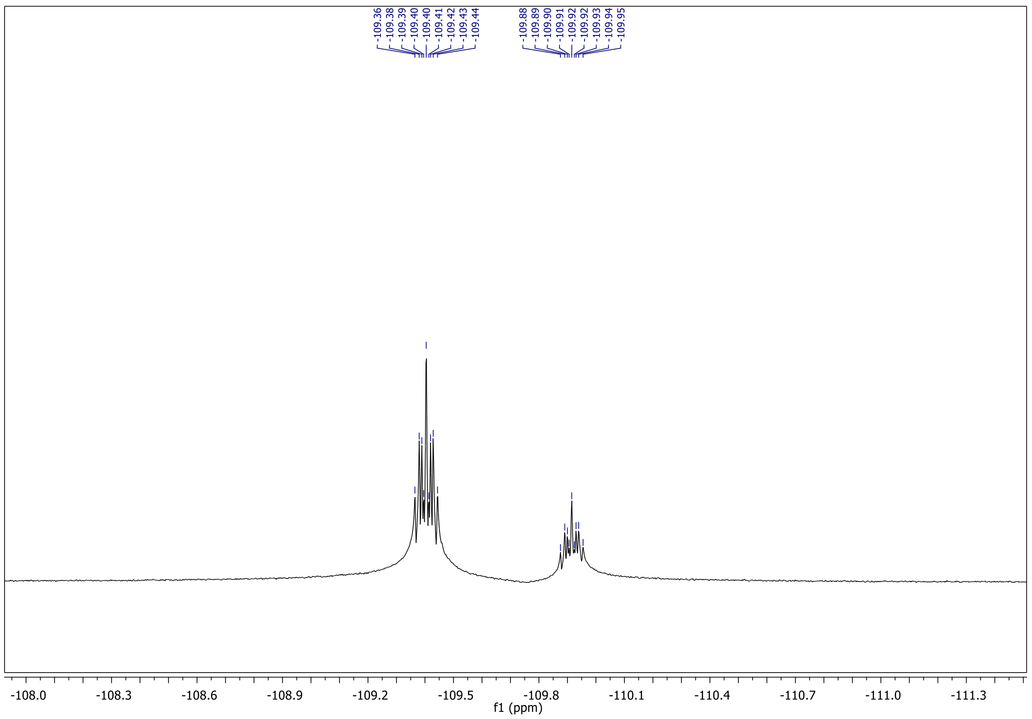

Figure S19. ^{19}FNMR of Compound 6

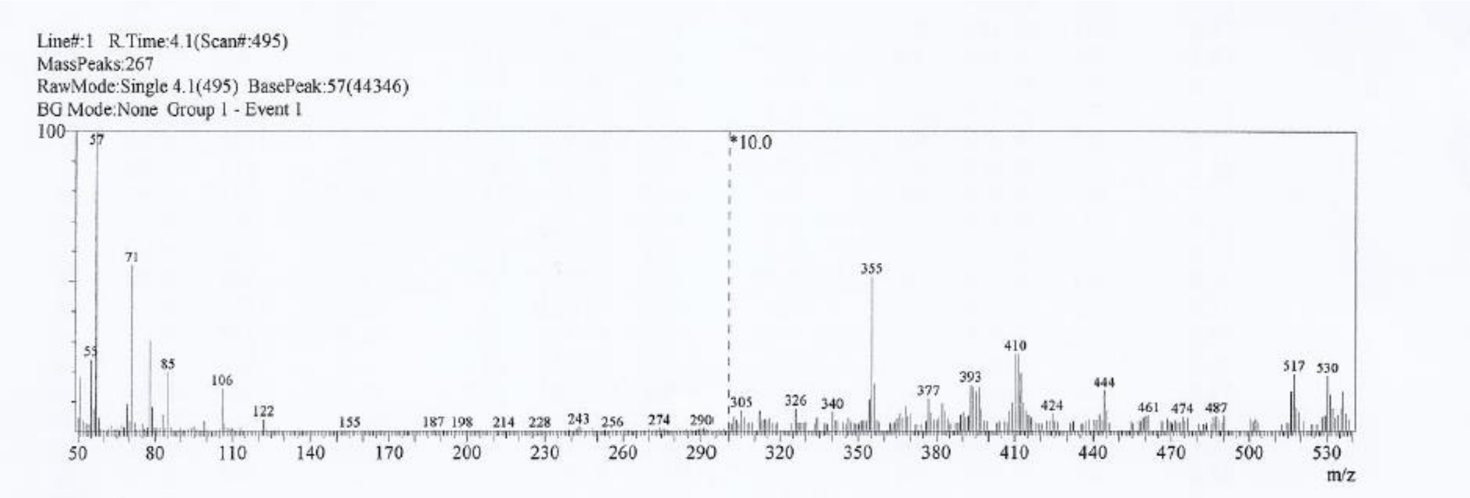

Figure S20. MS (ESI) of Compound 6

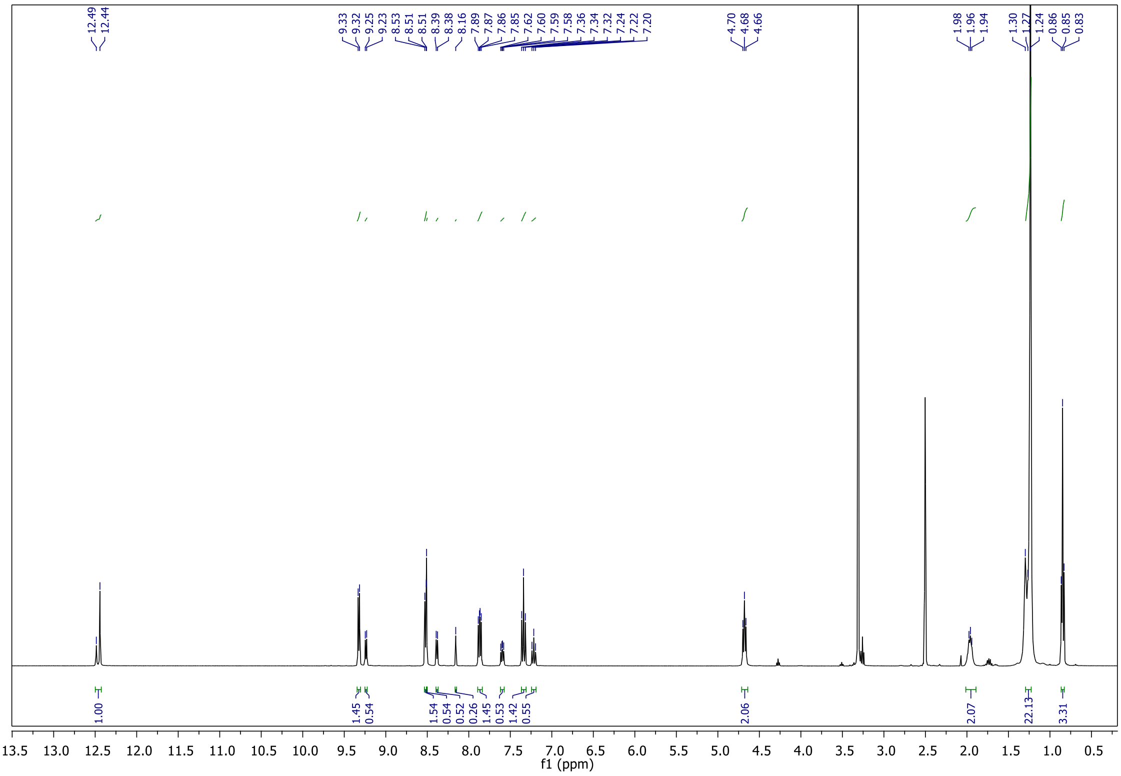

Figure S21. ^{1}HNMR of Compound 7

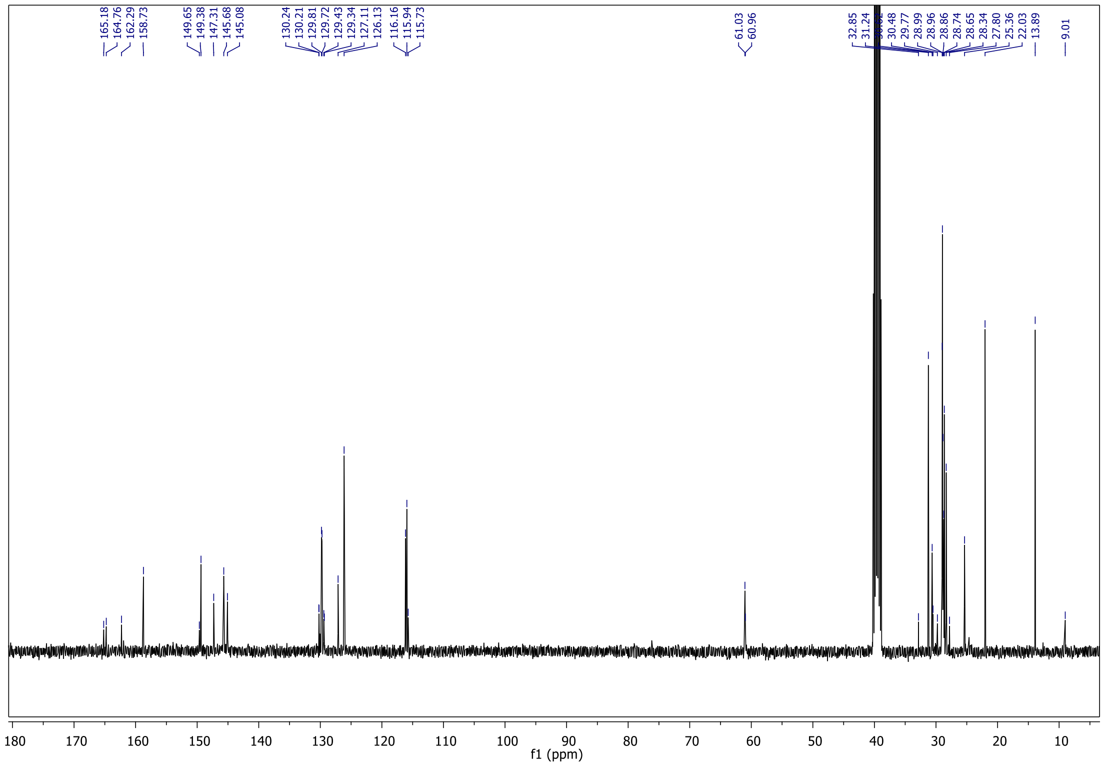

Figure S22. ^{13}CNMR of Compound 7

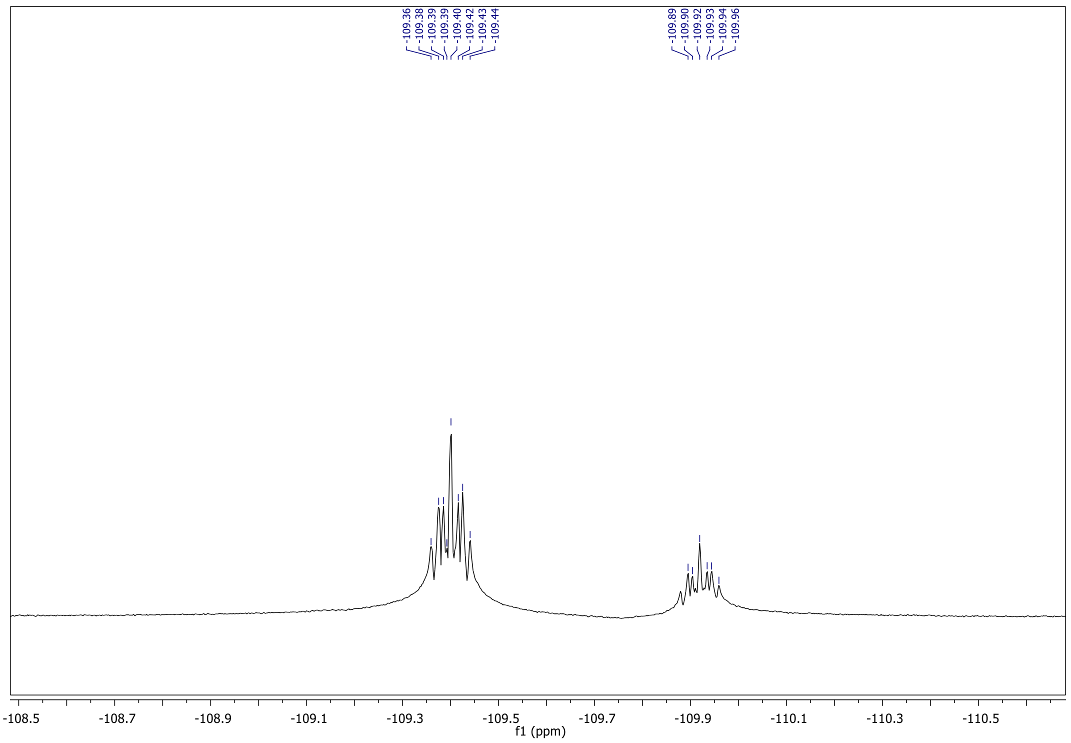

Figure S23. ^{19}FNMR of Compound 7

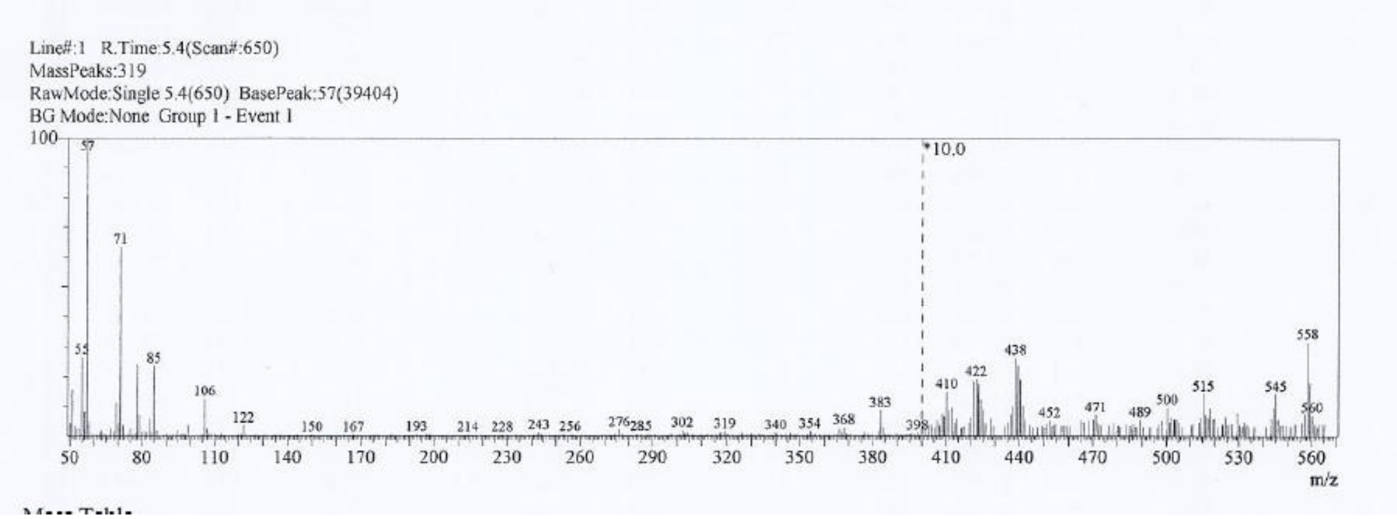

Figure S24. MS (ESI) of Compound 7

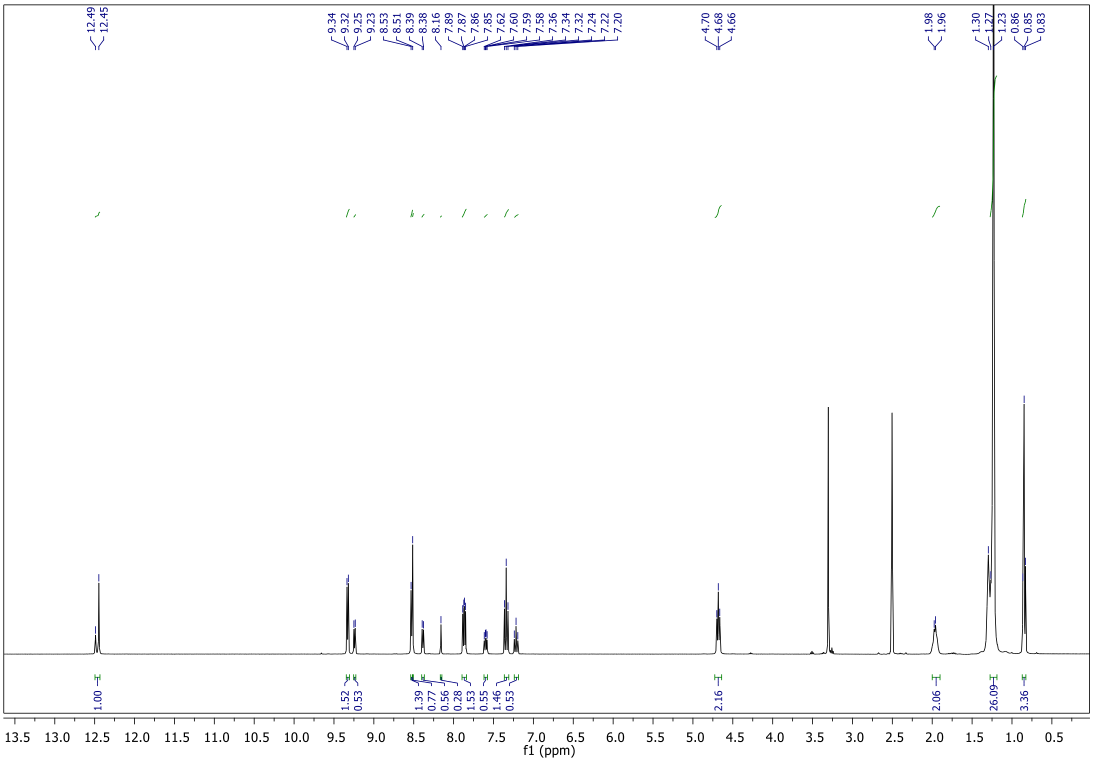

Figure S25. ¹HNMR of Compound 8

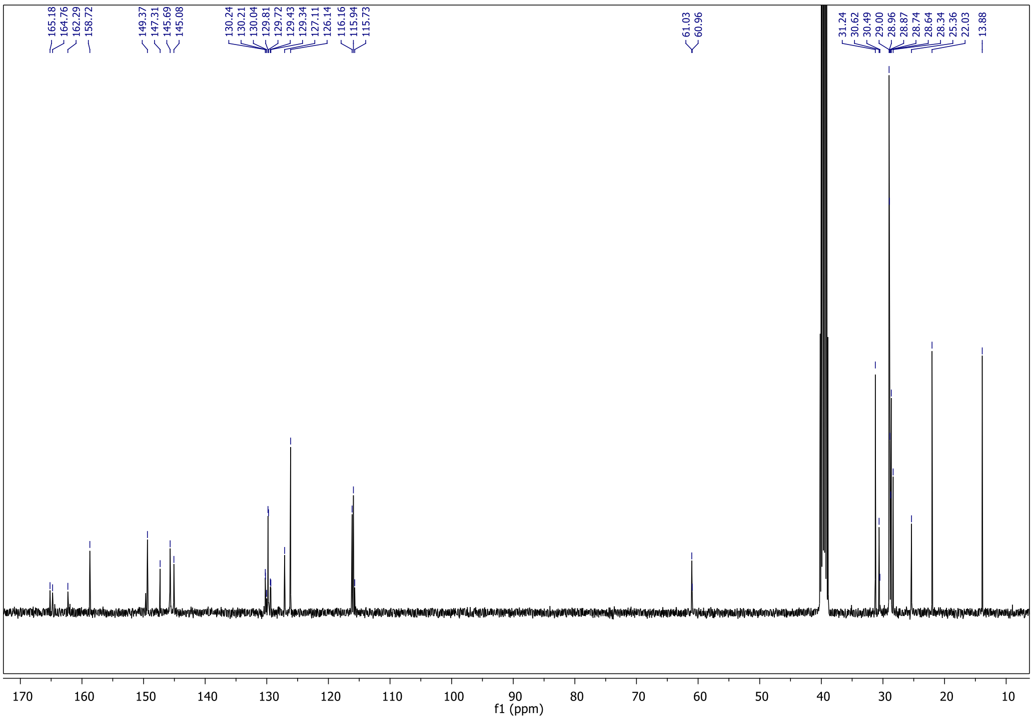

Figure S26. ¹³CNMR of Compound 8

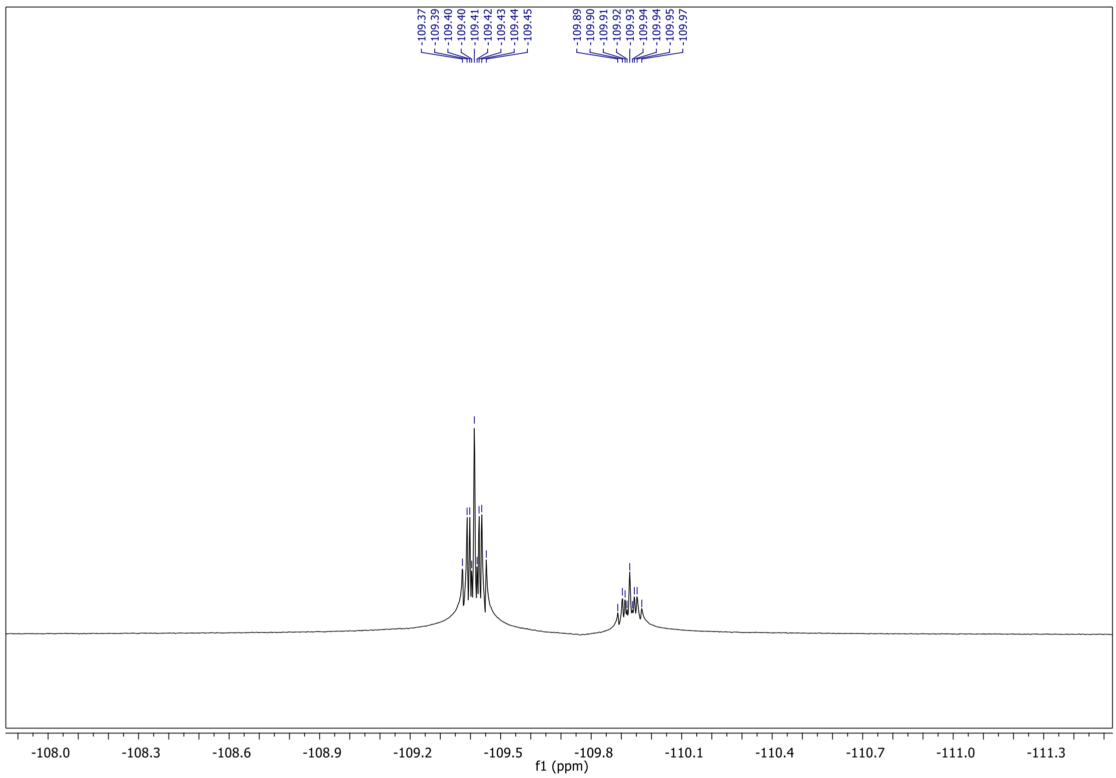

Figure S27. ^{19}FNMR of Compound 8

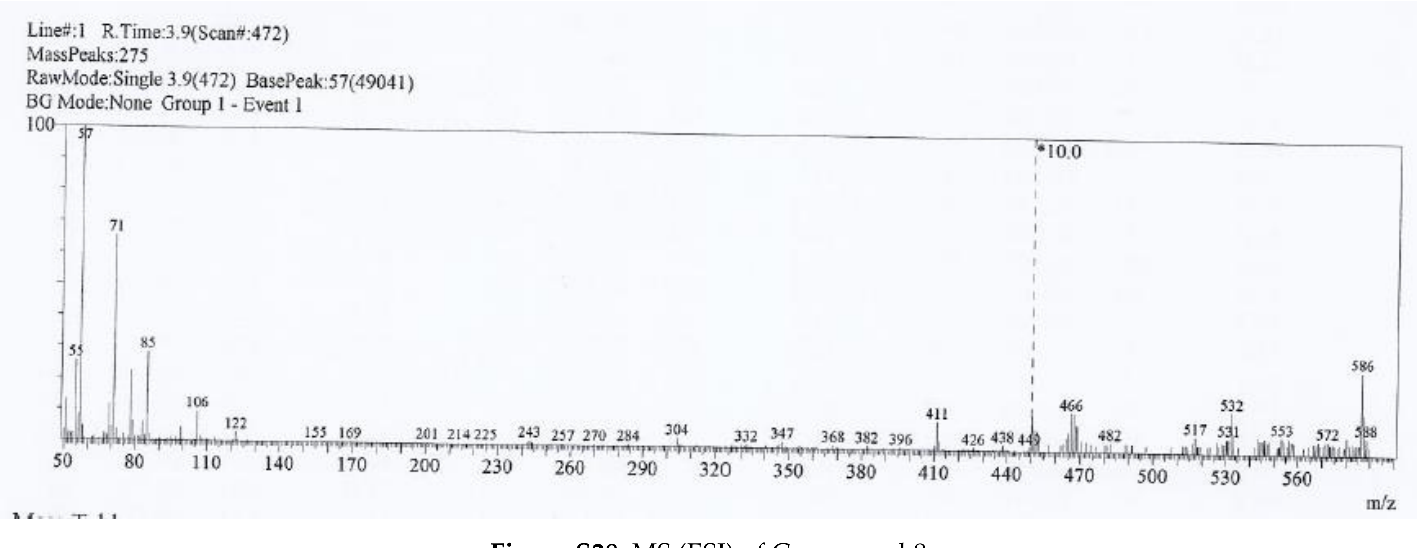

Figure S28. MS (ESI) of Compound 8

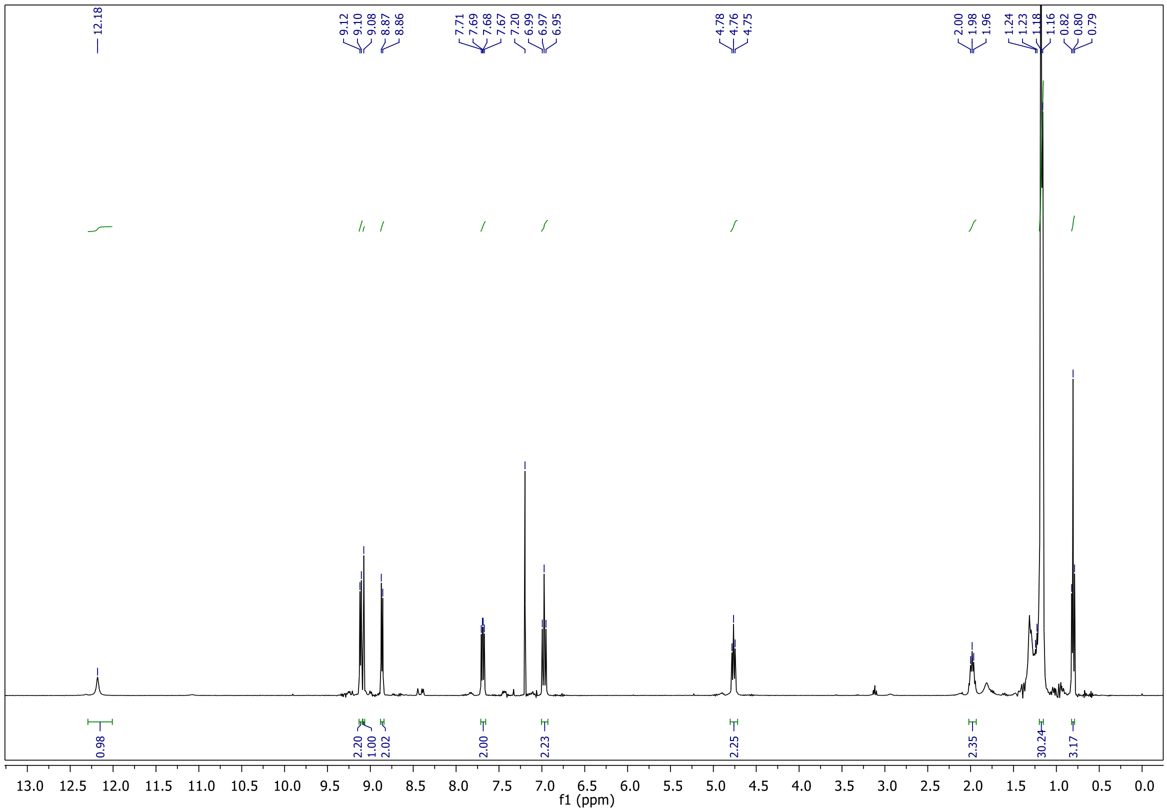

Figure S29. ^{1}HNMR of Compound 9

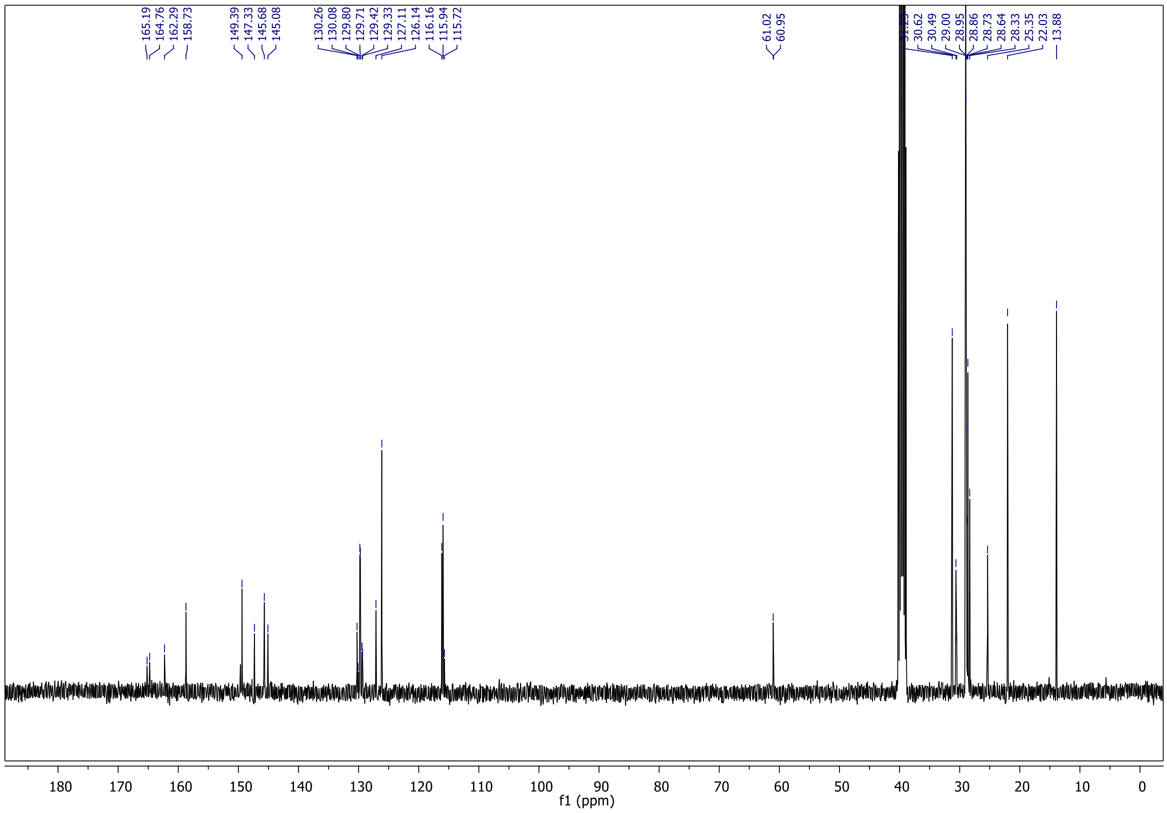

Figure S30. ^{13}CNMR of Compound 9

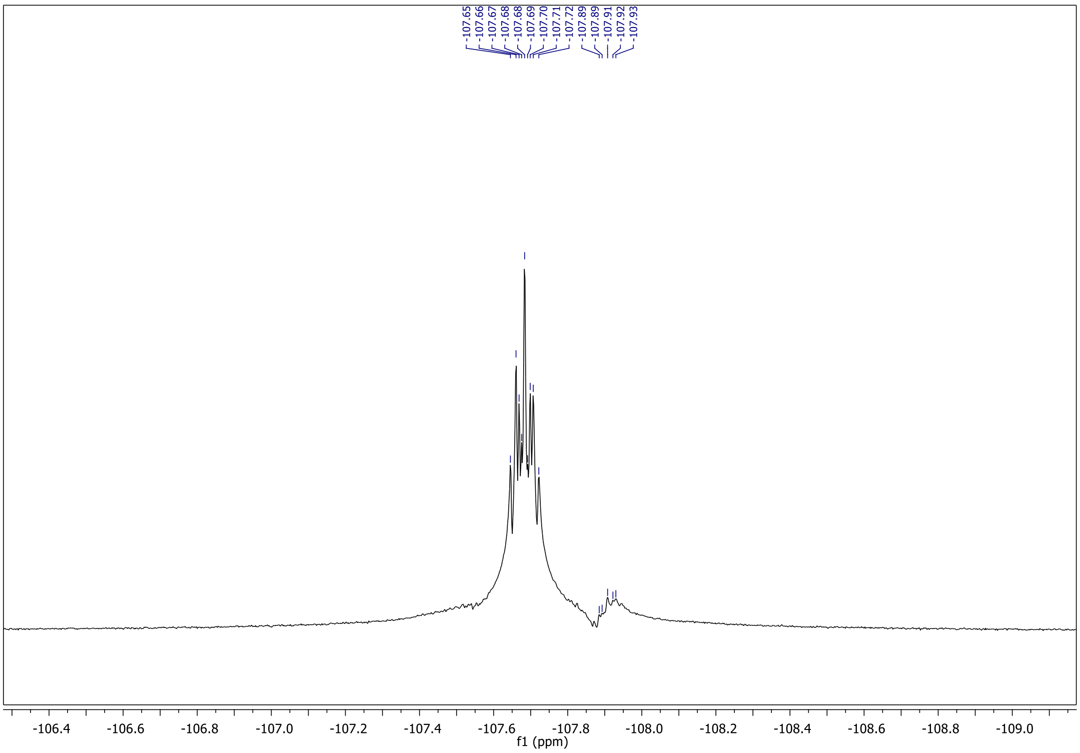

Figure S31. ^{19}FNMR of Compound 9

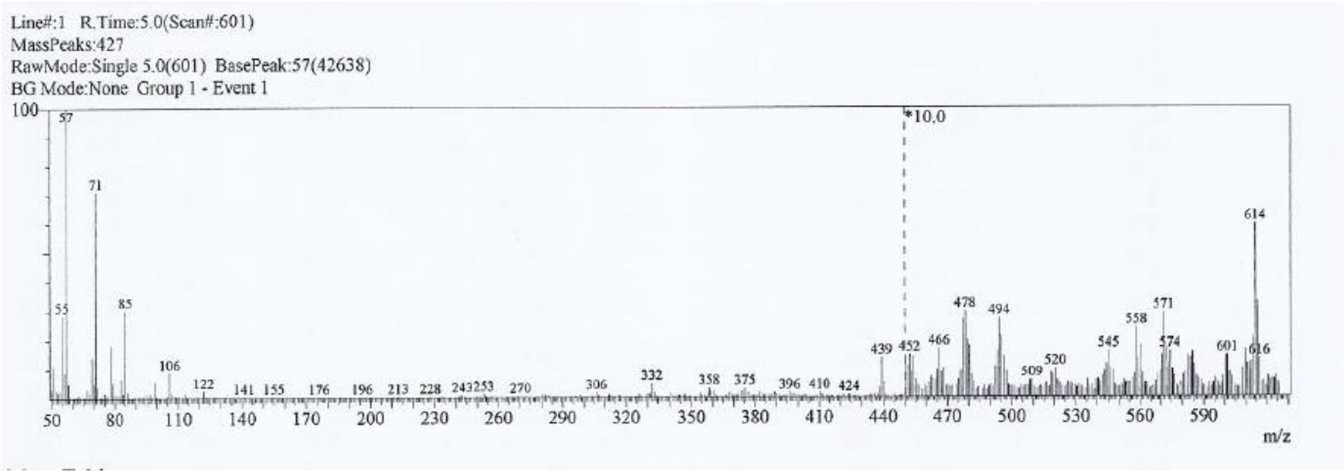

Figure S32. MS (ESI) of Compound 9

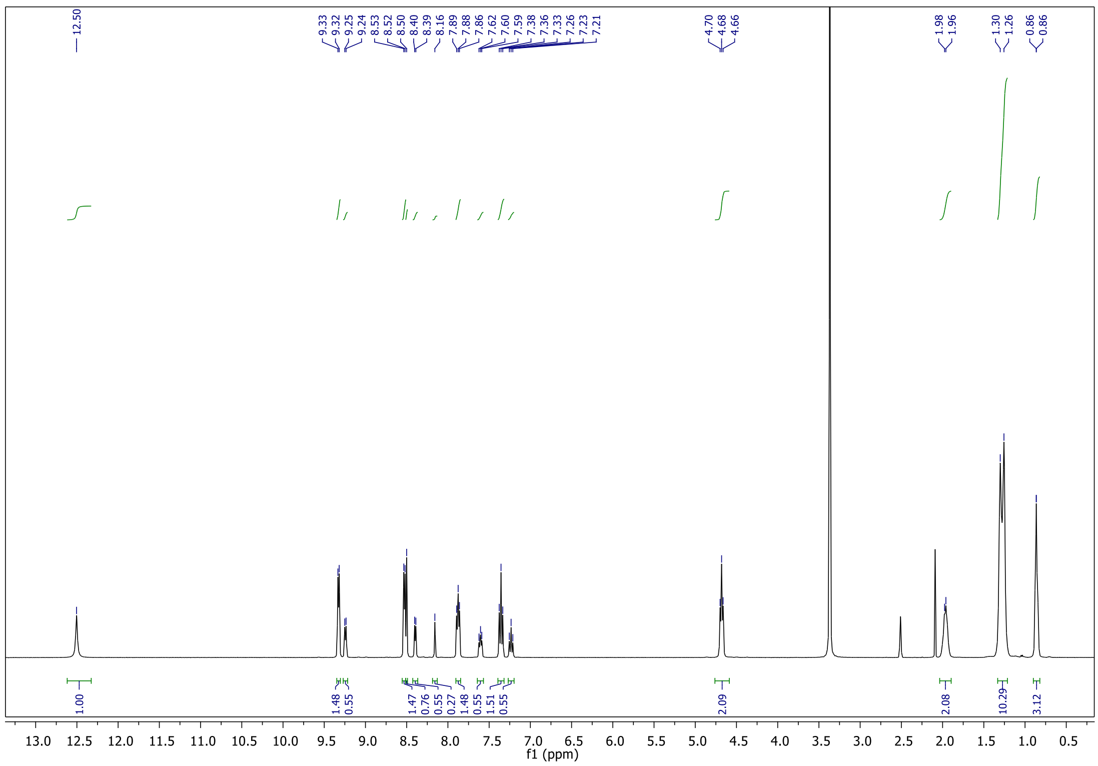

Figure S33. ^{1}HNMR of Compound 10

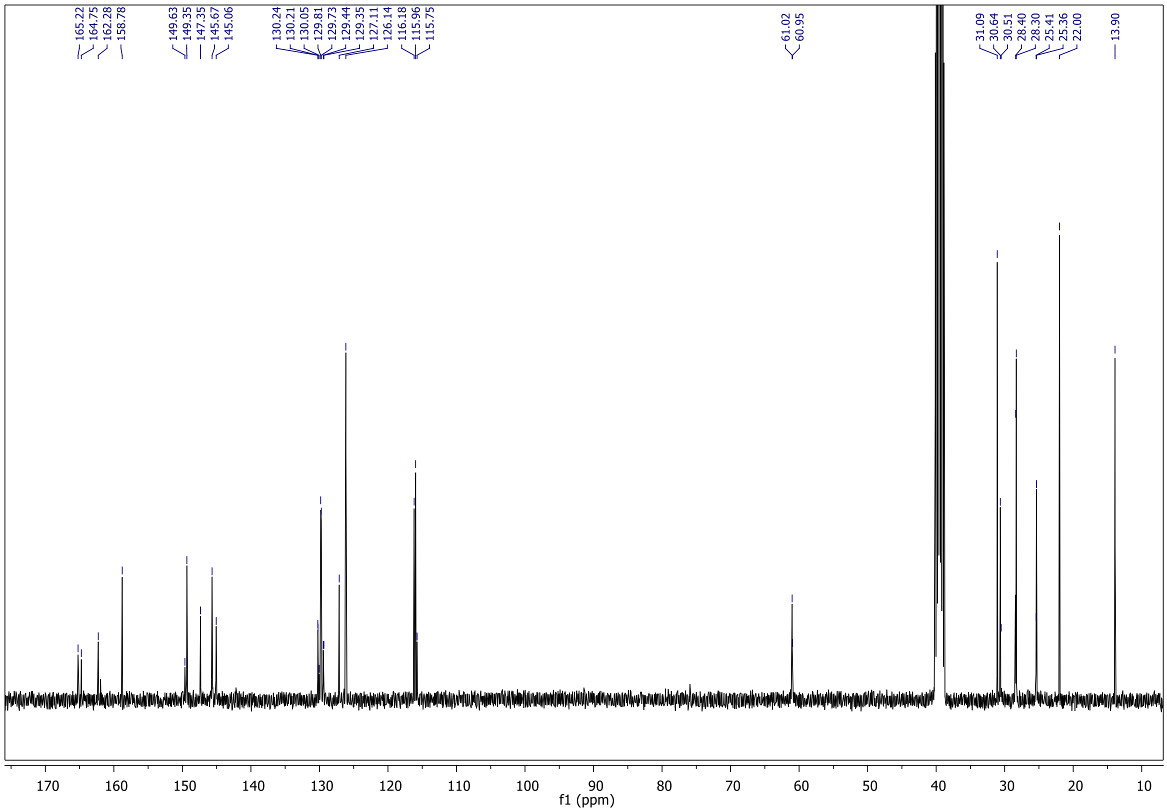

Figure S34. ^{13}CNMR of Compound 10

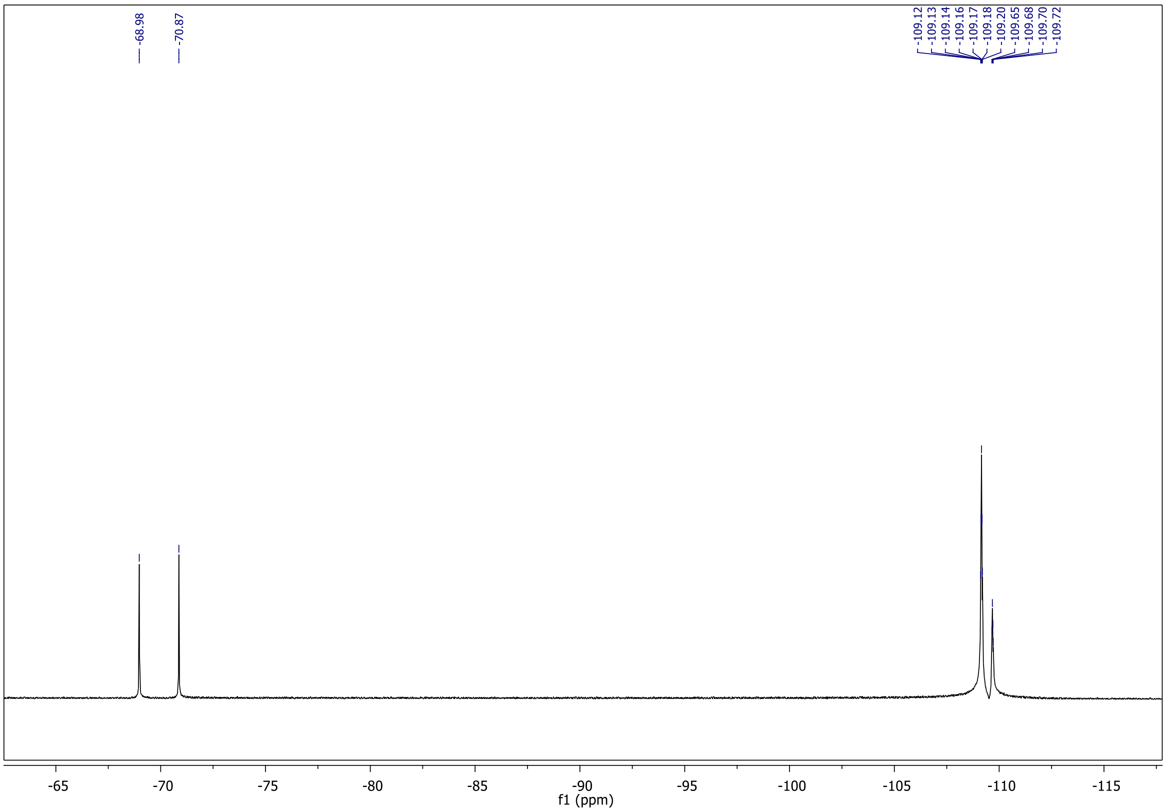

Figure S35. ^{19}FNMR of Compound 10

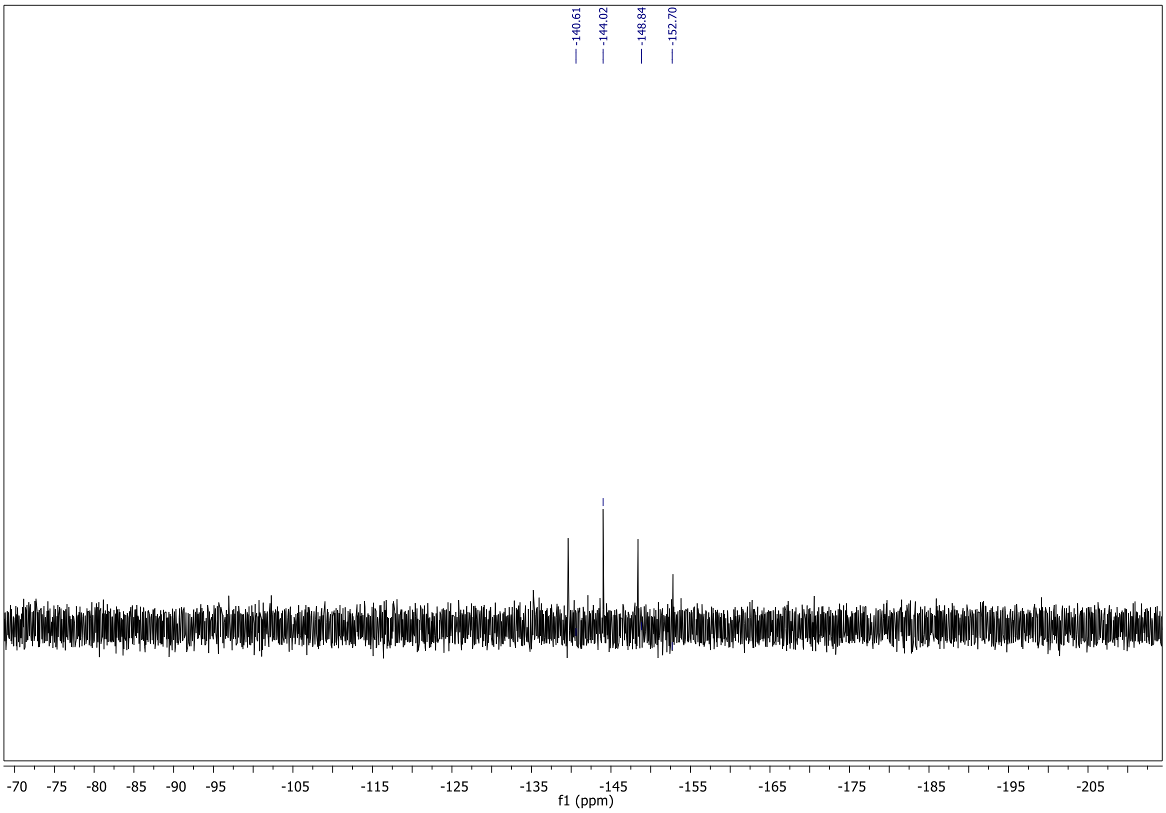

Figure S36. ^{31}PNMR of Compound 10

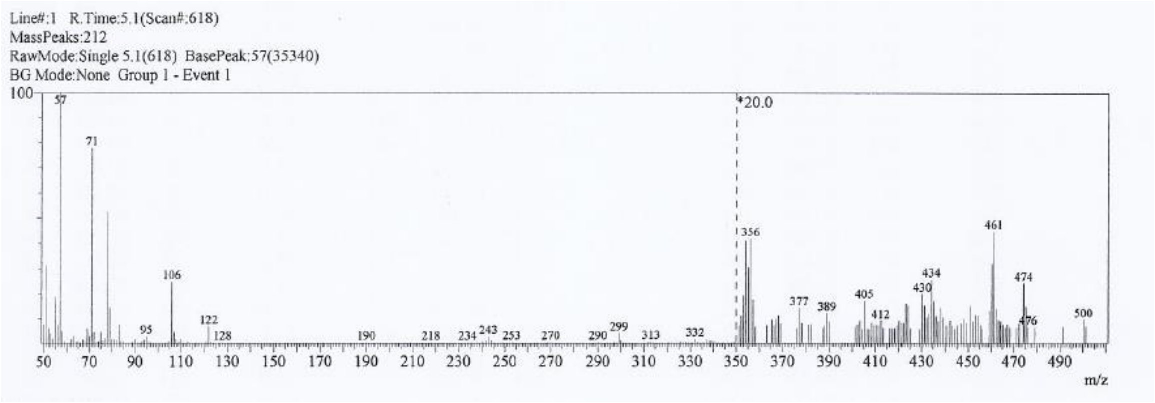

Figure S37. MS (ESI) of Compound 10

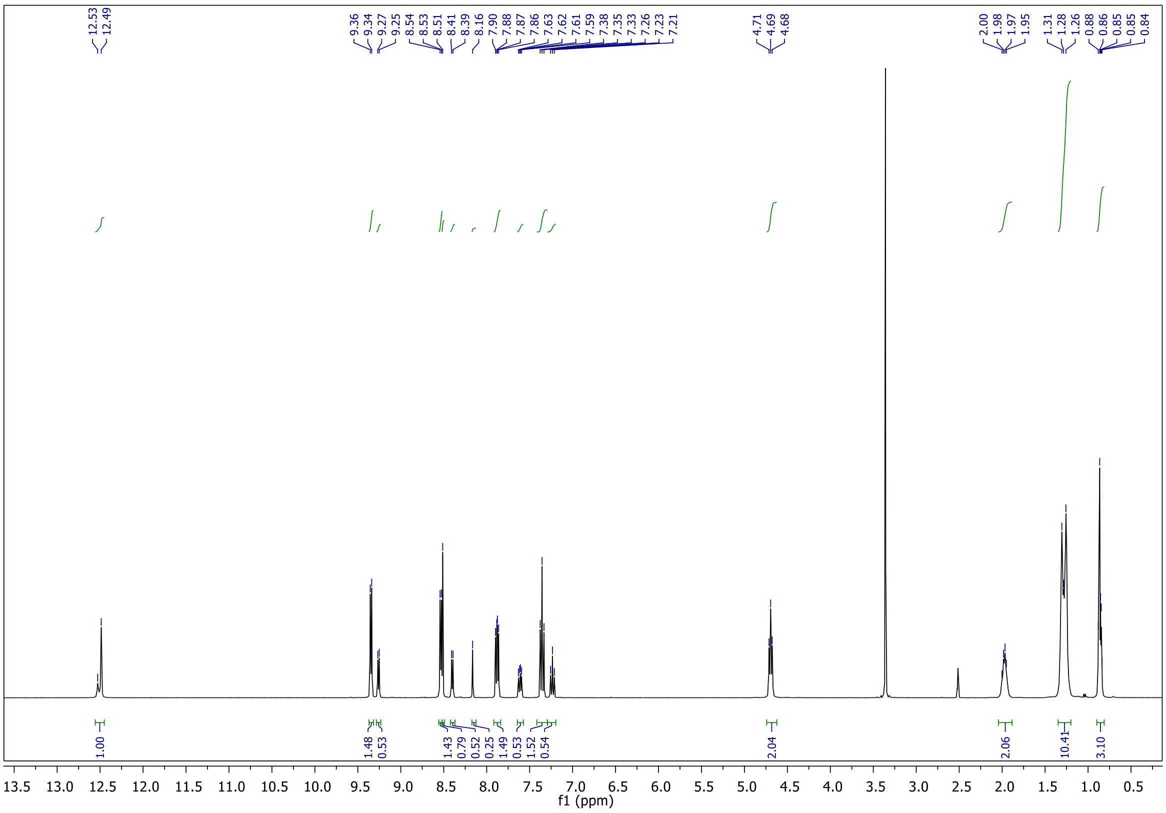

Figure S38. ^{1}HNMR of Compound 11

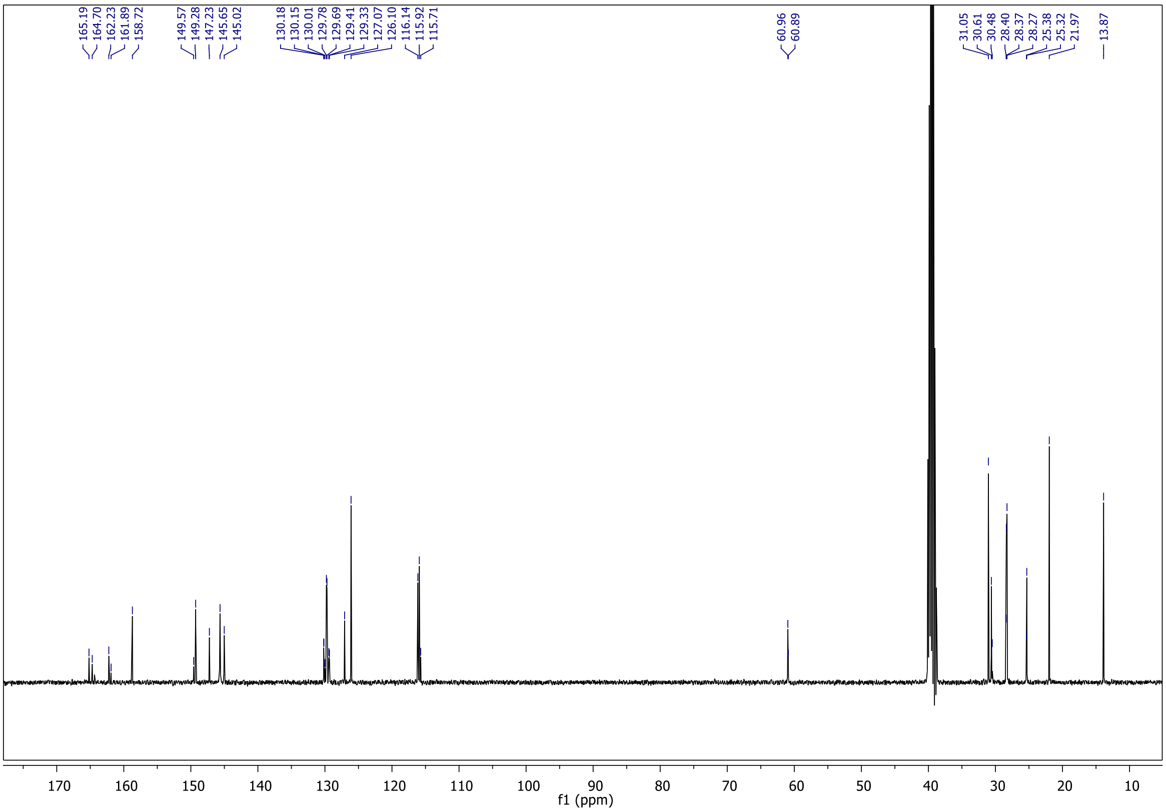

Figure S39. ^{13}CNMR of Compound 11

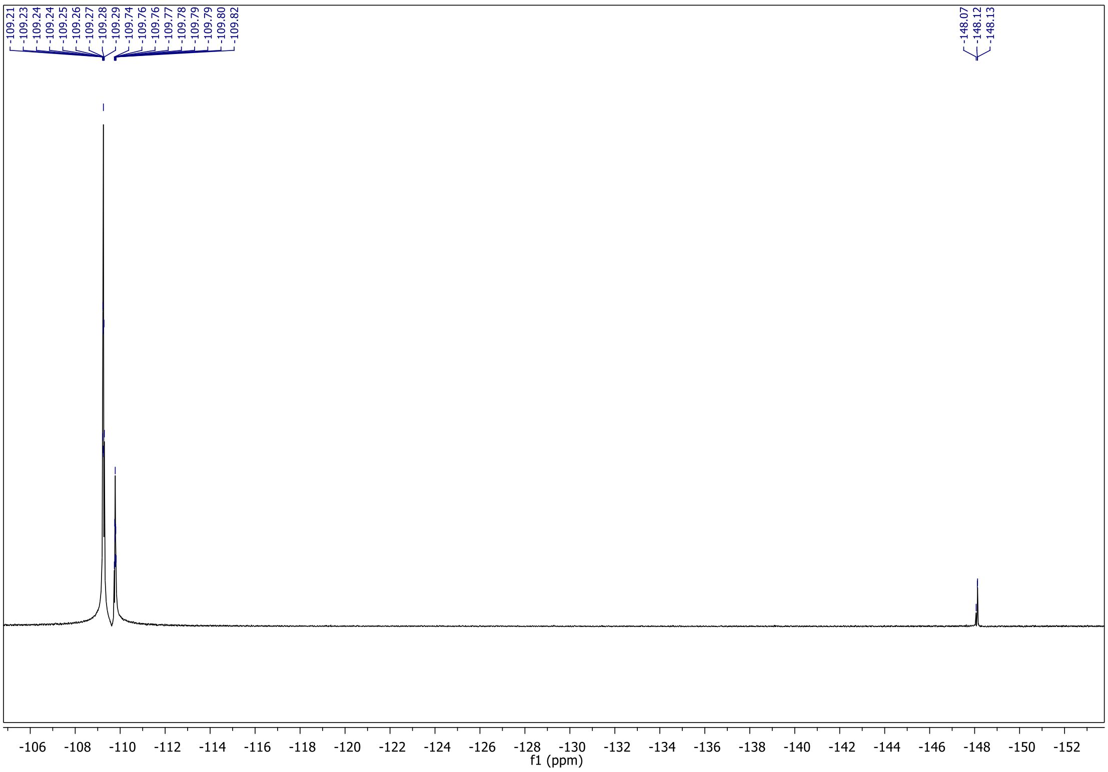

Figure S40. ^{19}FNMR of Compound 11

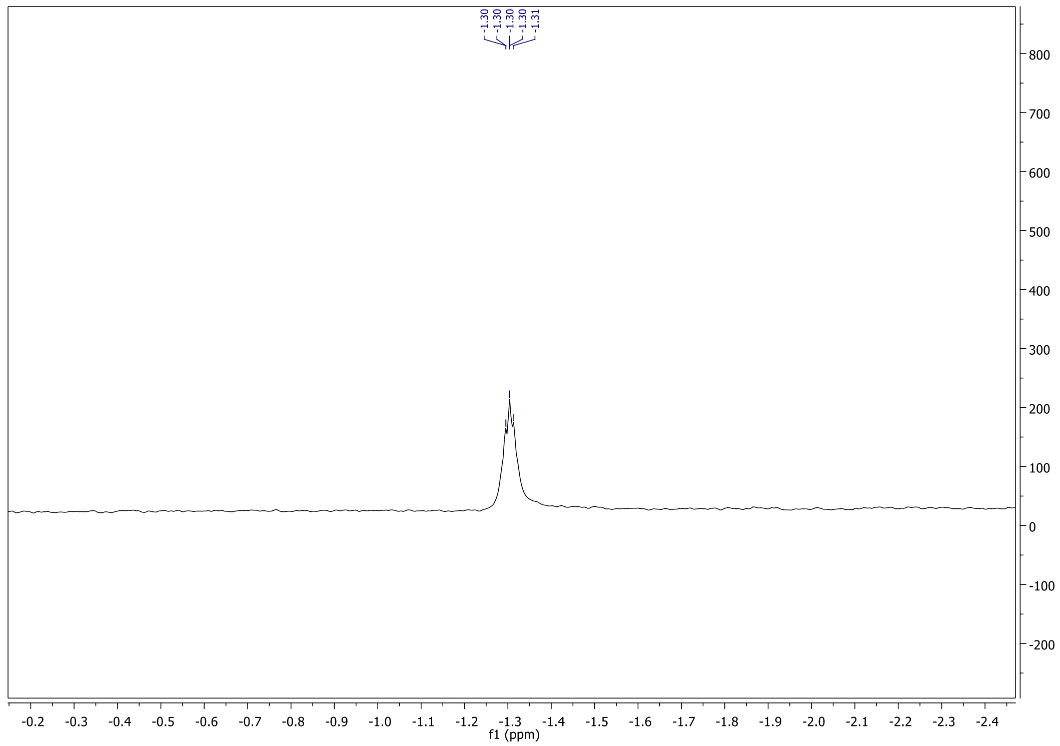

Figure S41. ^{11}BNMR of Compound 11

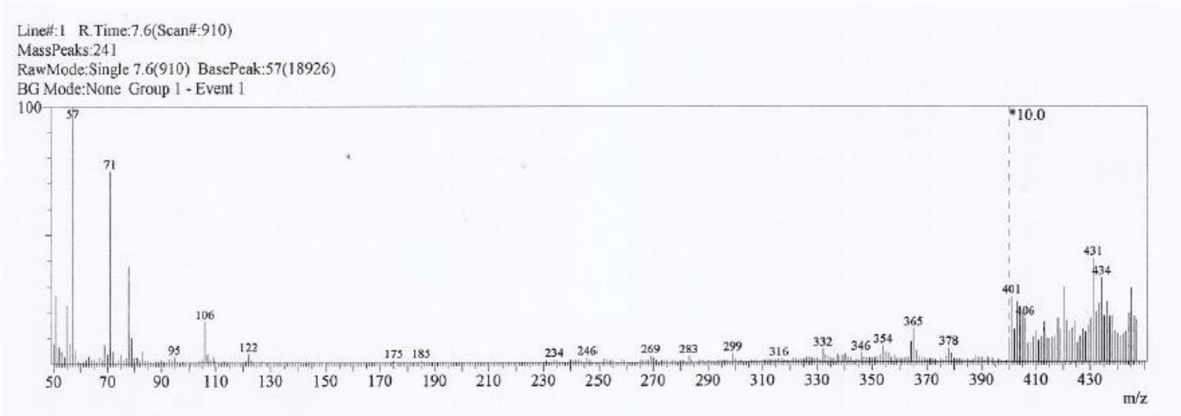

Figure S42. MS (ESI) of Compound 11

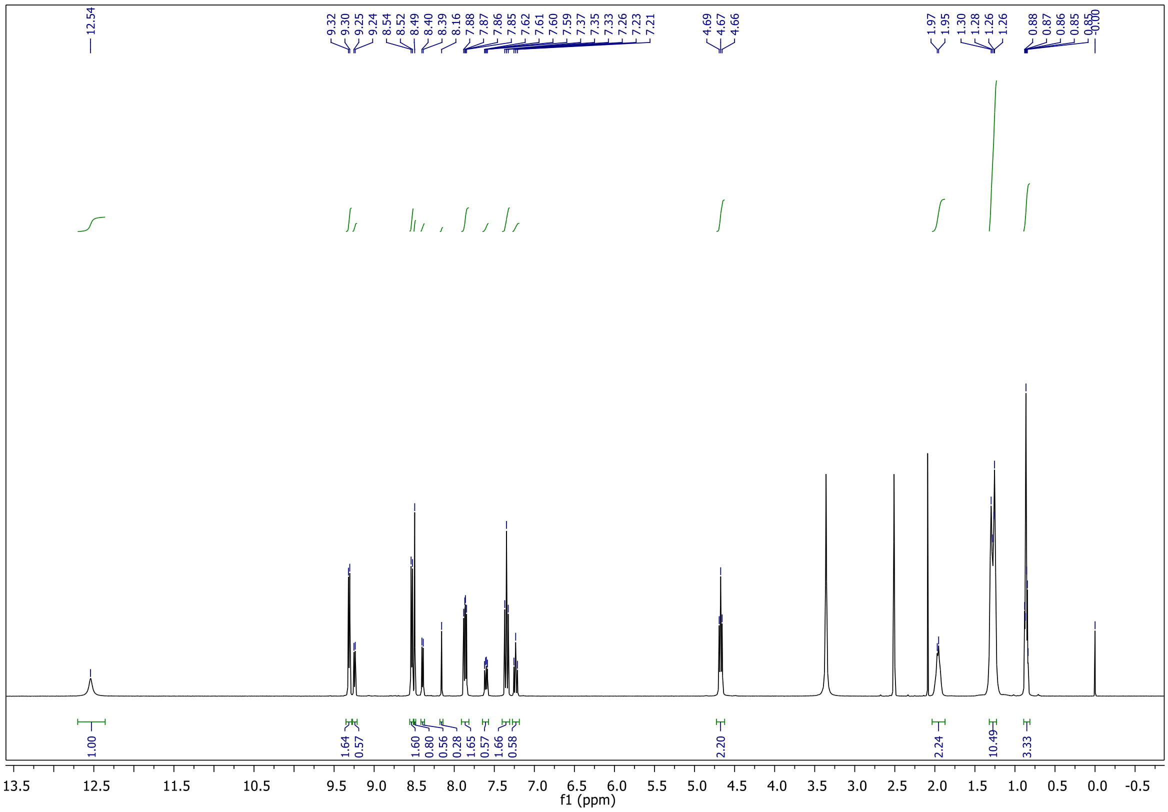

Figure S43. ^{1}HNMR of Compound 12

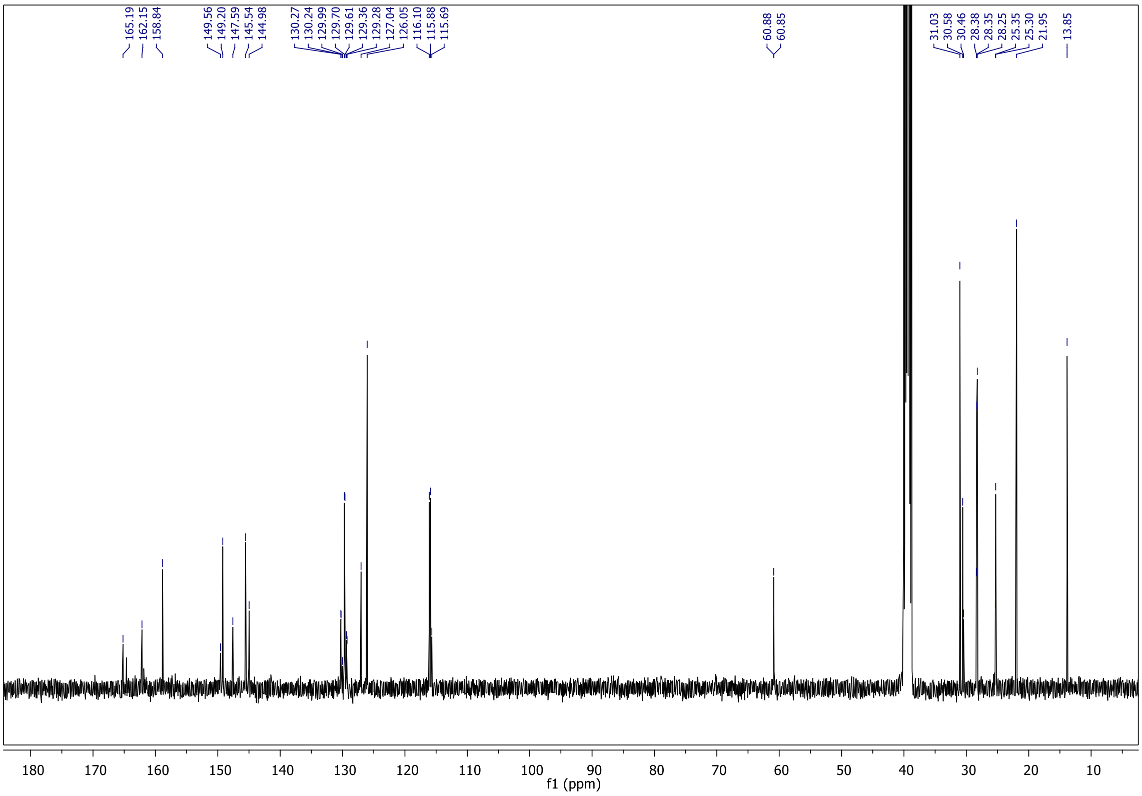

Figure S44. ^{13}CNMR of Compound 12

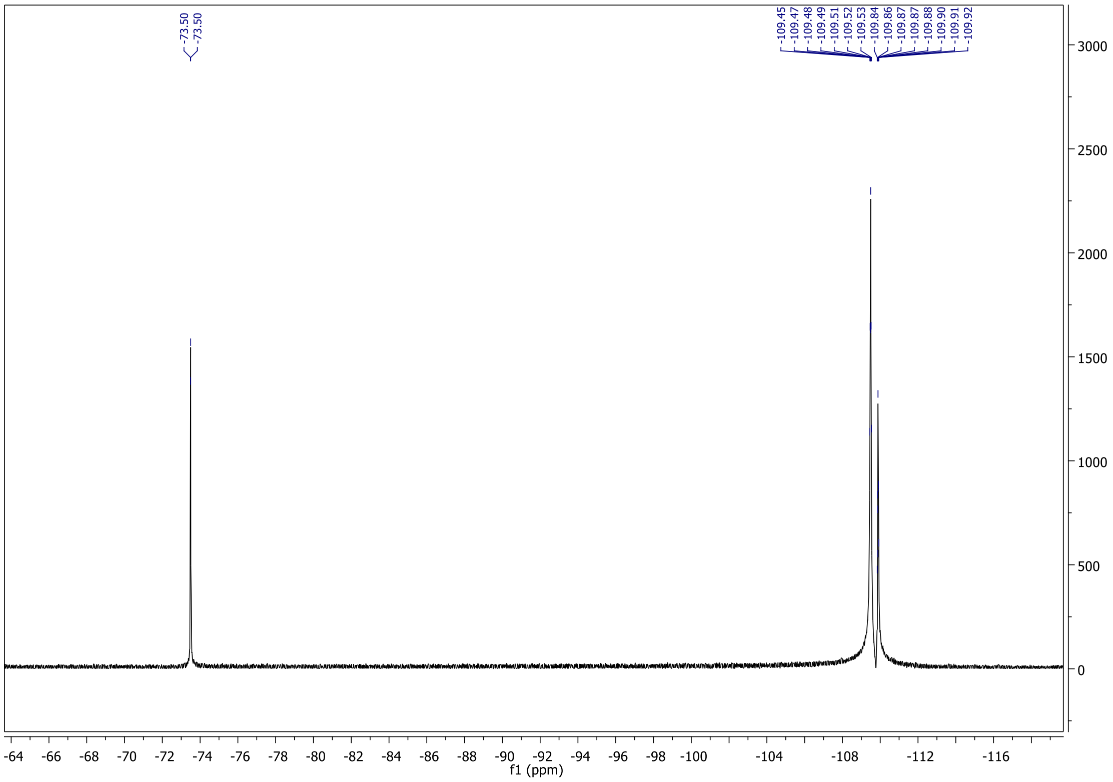

Figure S45. ^{19}FNMR of Compound 12

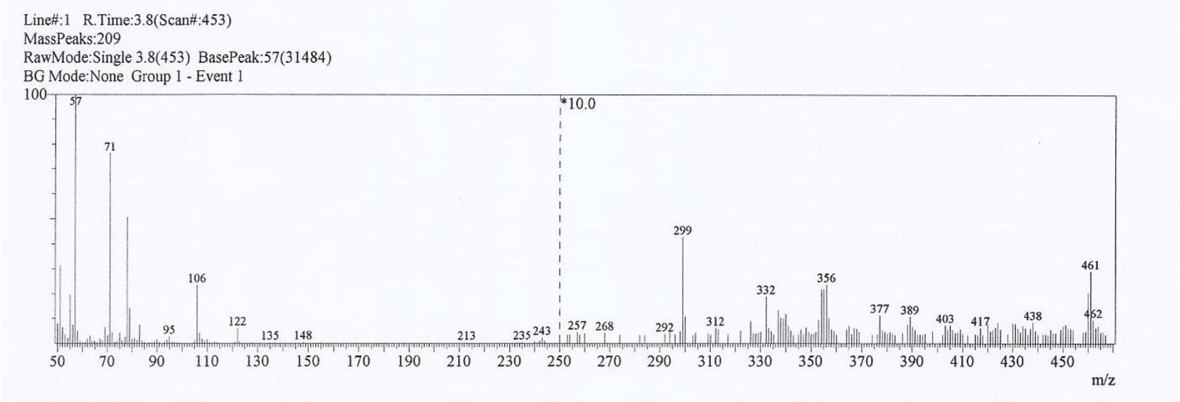

Figure S46. MS (ESI) of Compound 12

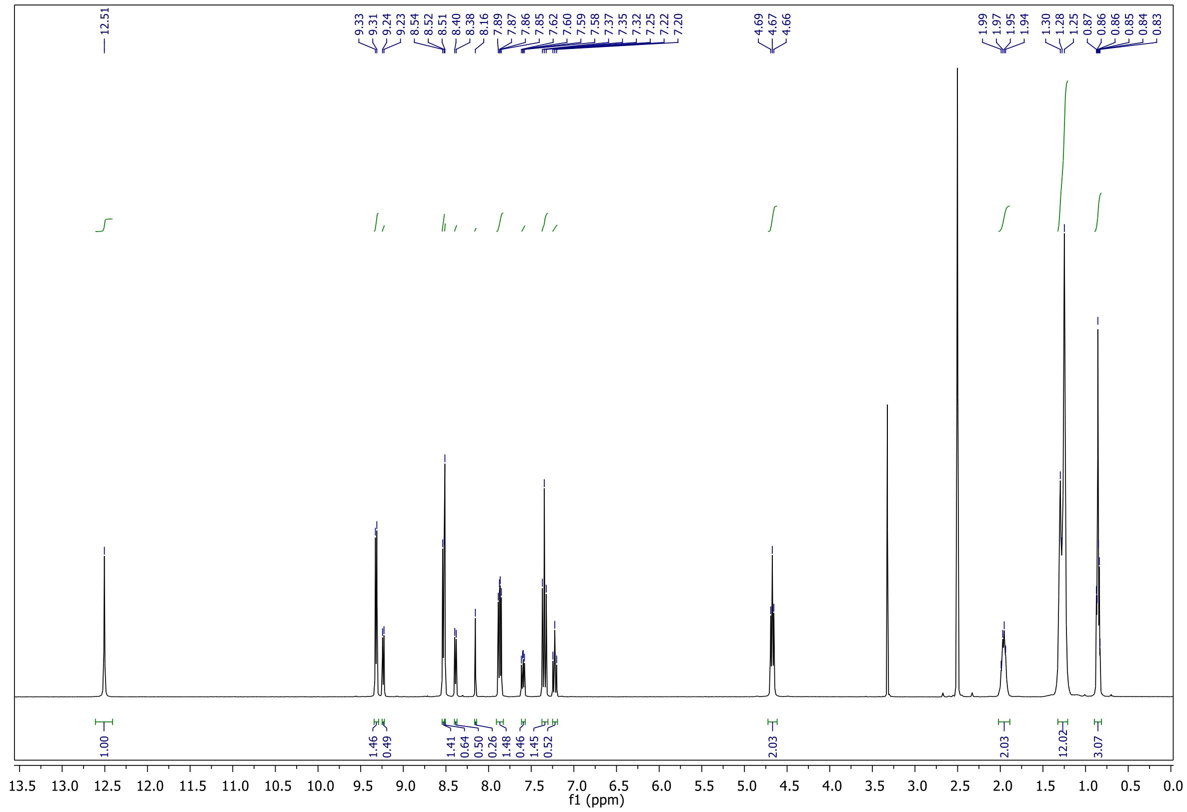

Figure S47. ^{1}HNMR of Compound 13

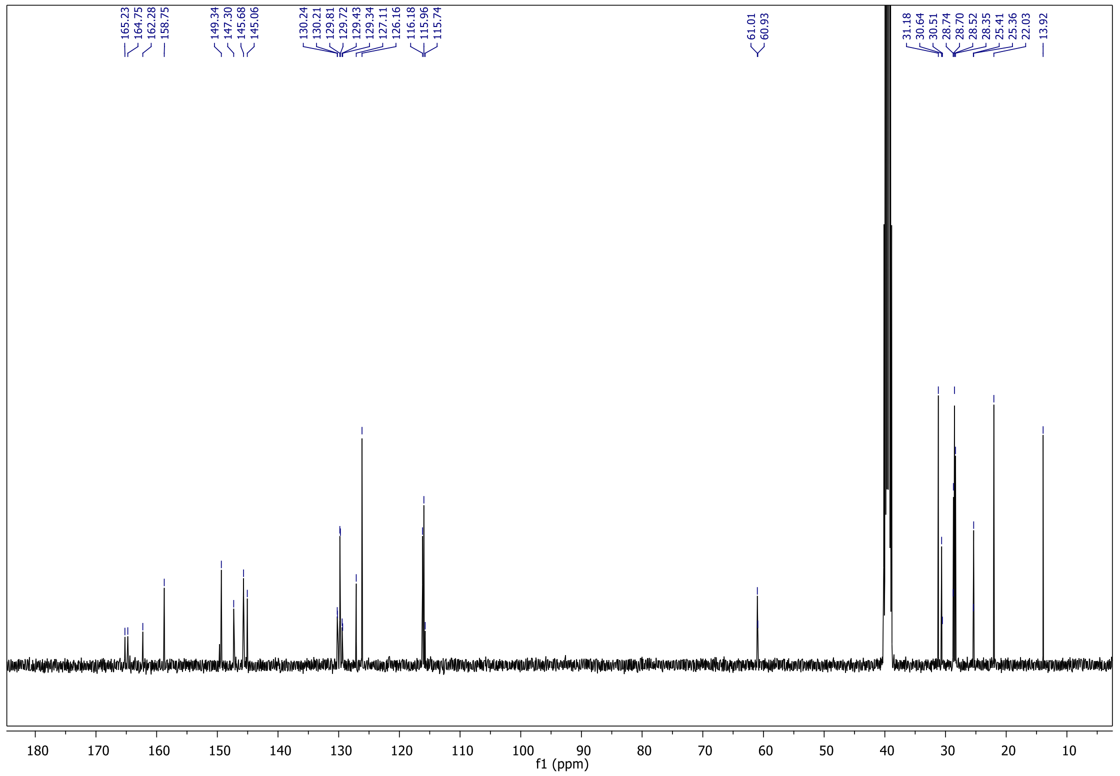

Figure S48. ^{13}CNMR of Compound 13

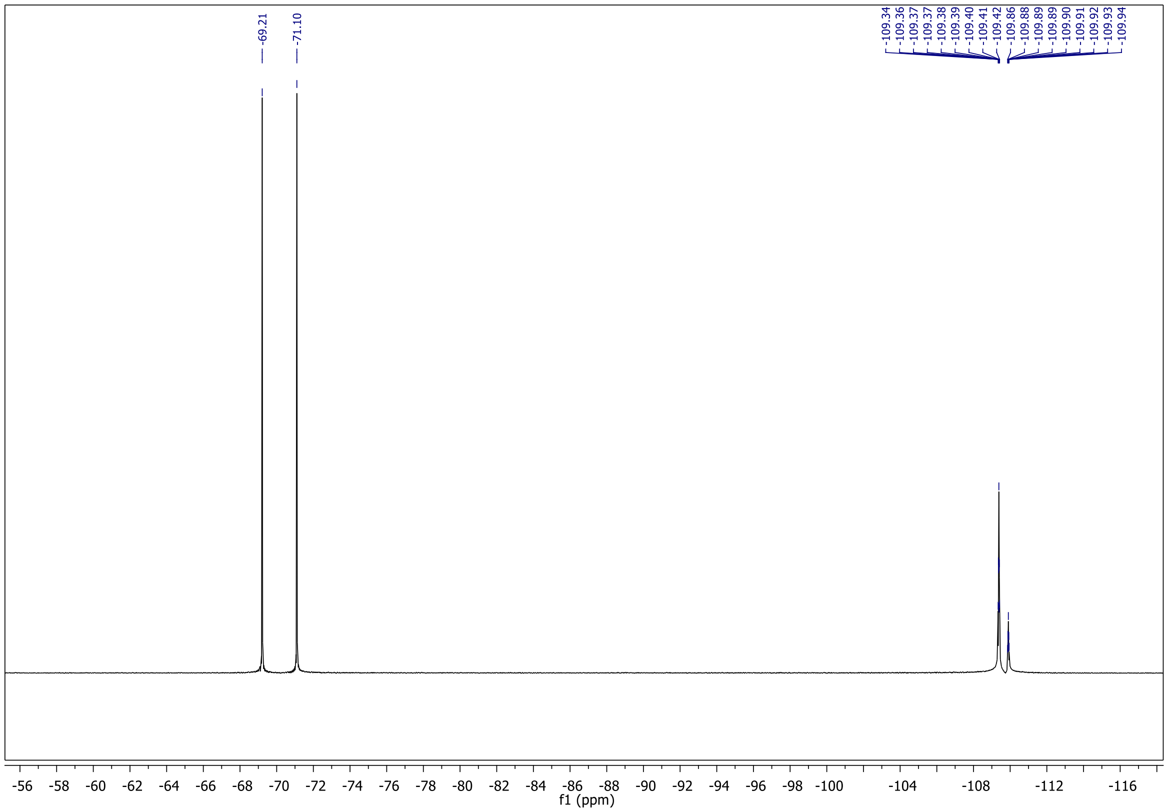

Figure S49. ^{19}FNMR of Compound 13

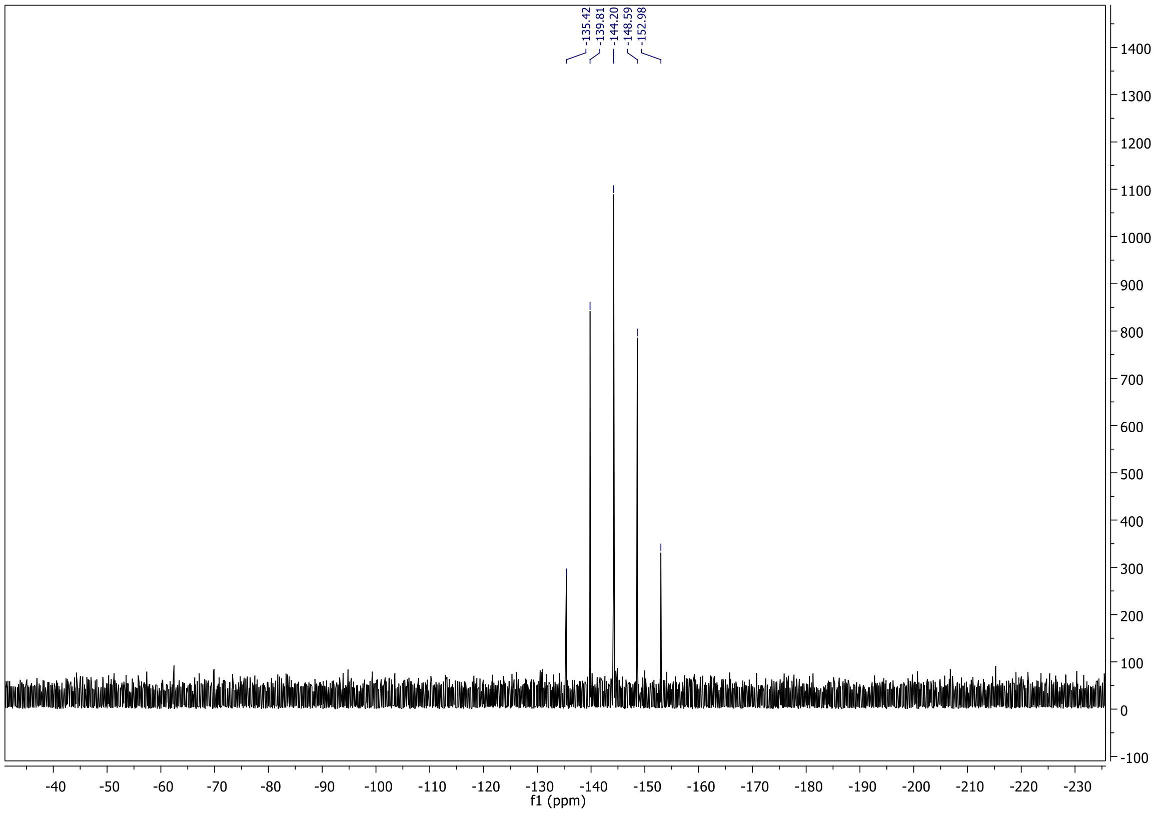

Figure S50. ^{31}PNMR of Compound 13

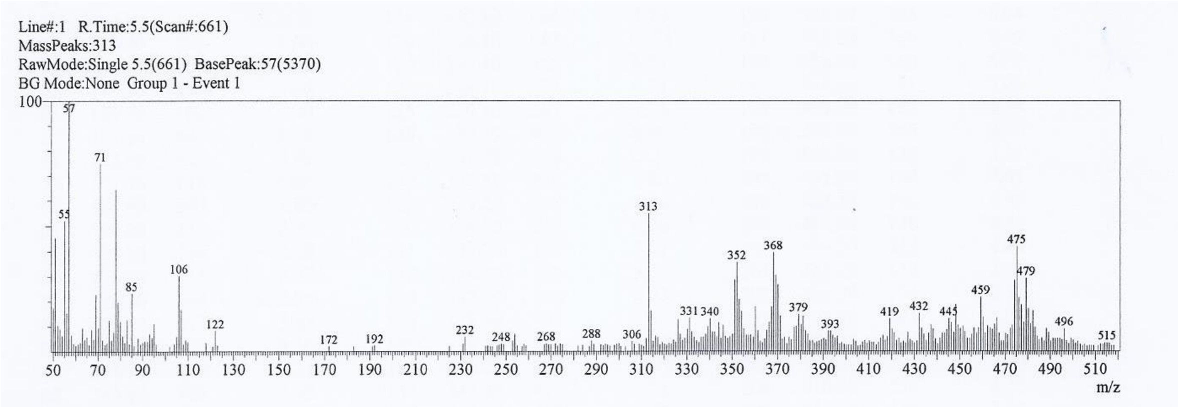

Figure S51. MS (ESI) of Compound 13

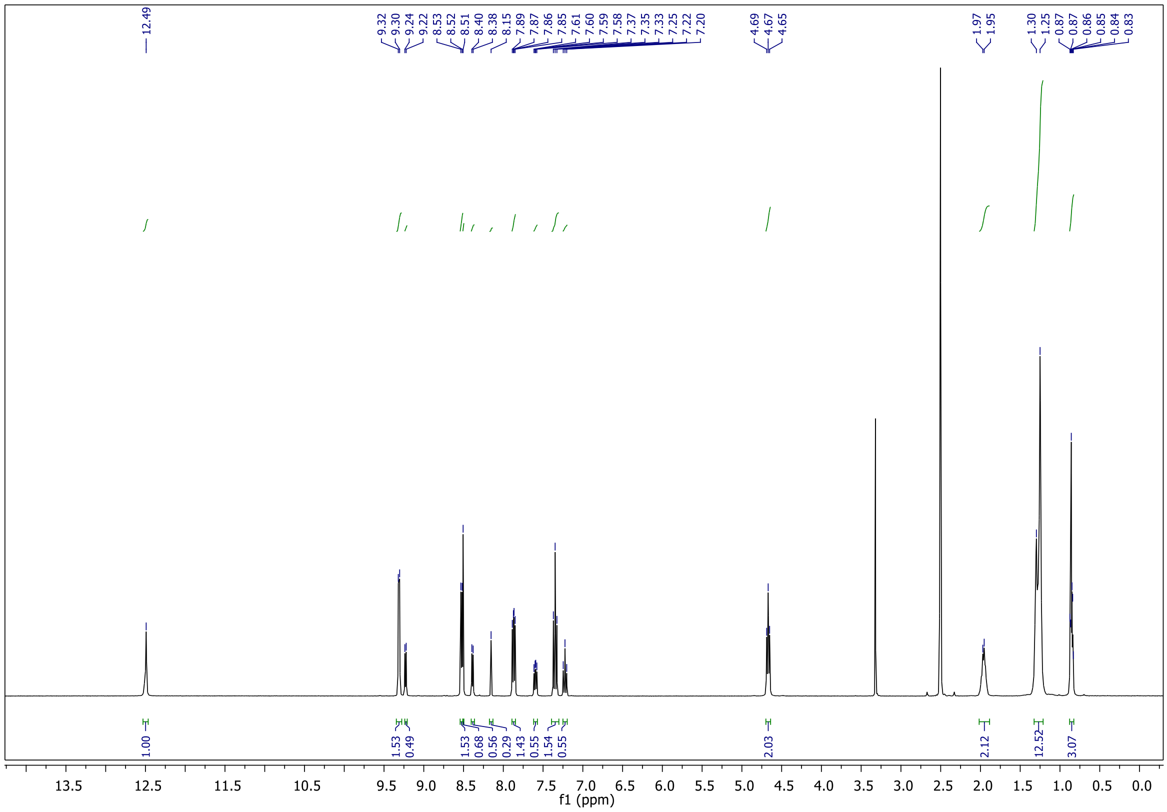

Figure S52. ^{1}HNMR of Compound 14

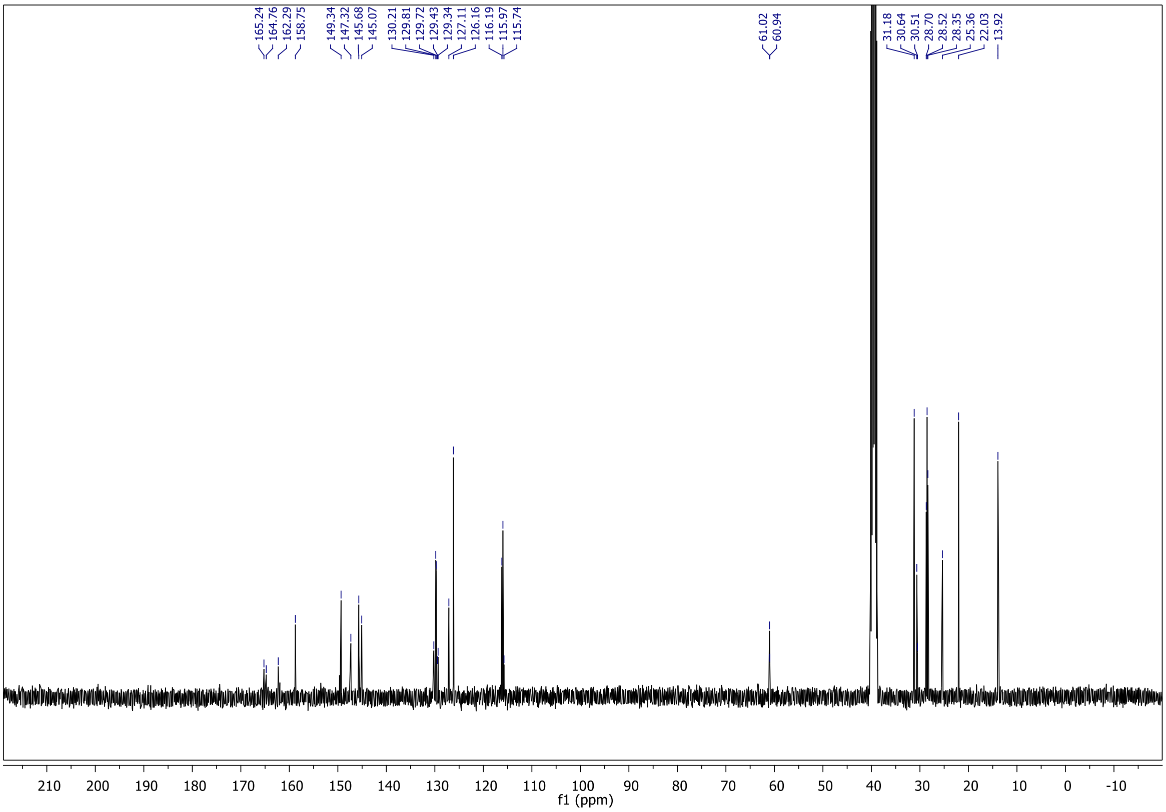

Figure S53. ^{13}CNMR of Compound 14

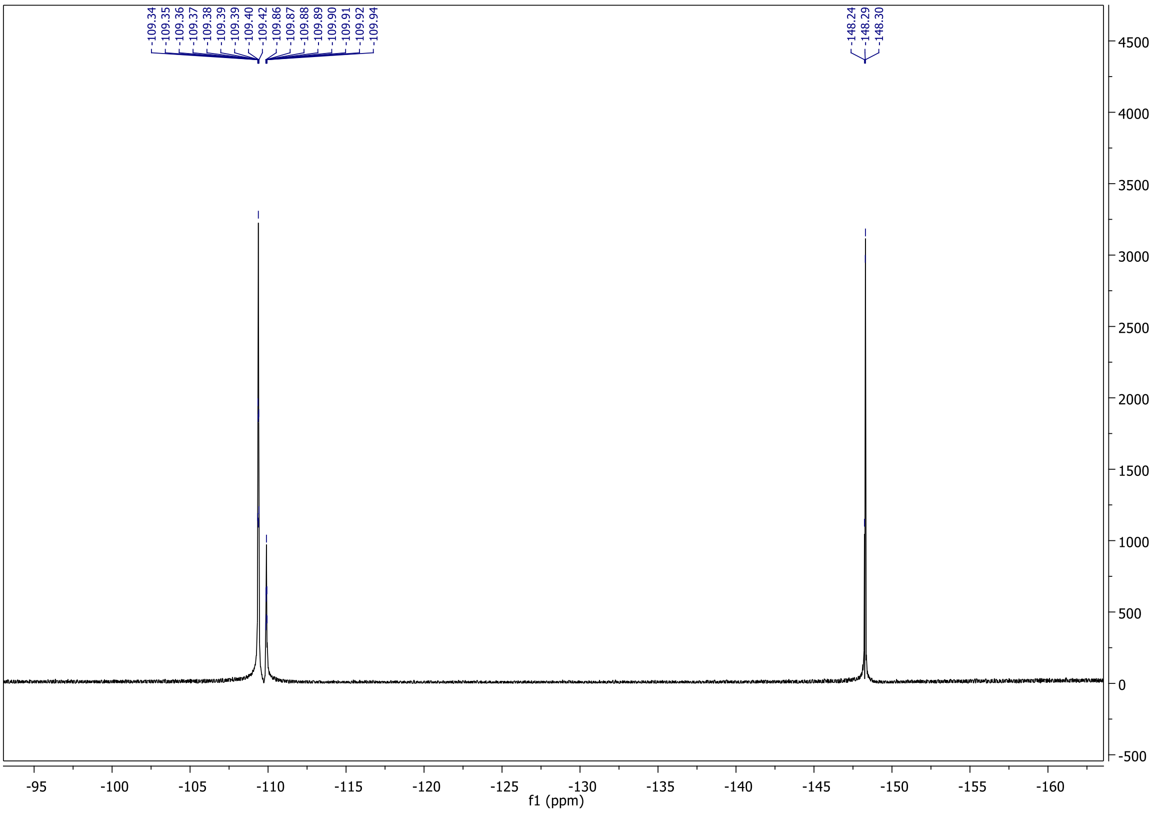

Figure S54. ^{19}FNMR of Compound 14

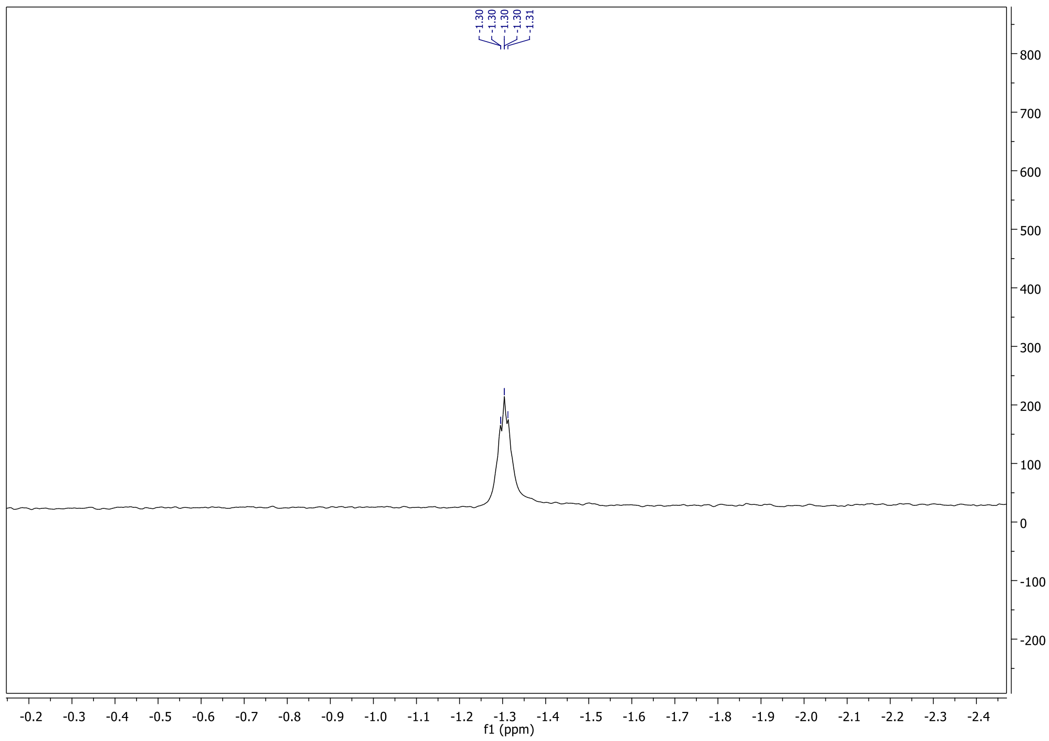

Figure S55. ^{11}BNMR of Compound 14

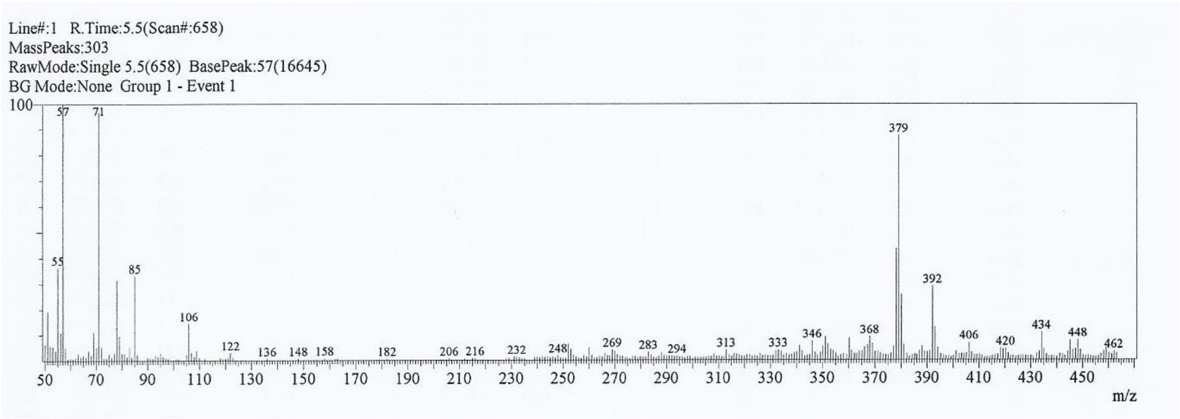

Figure S56. MS (ESI) of Compound 14

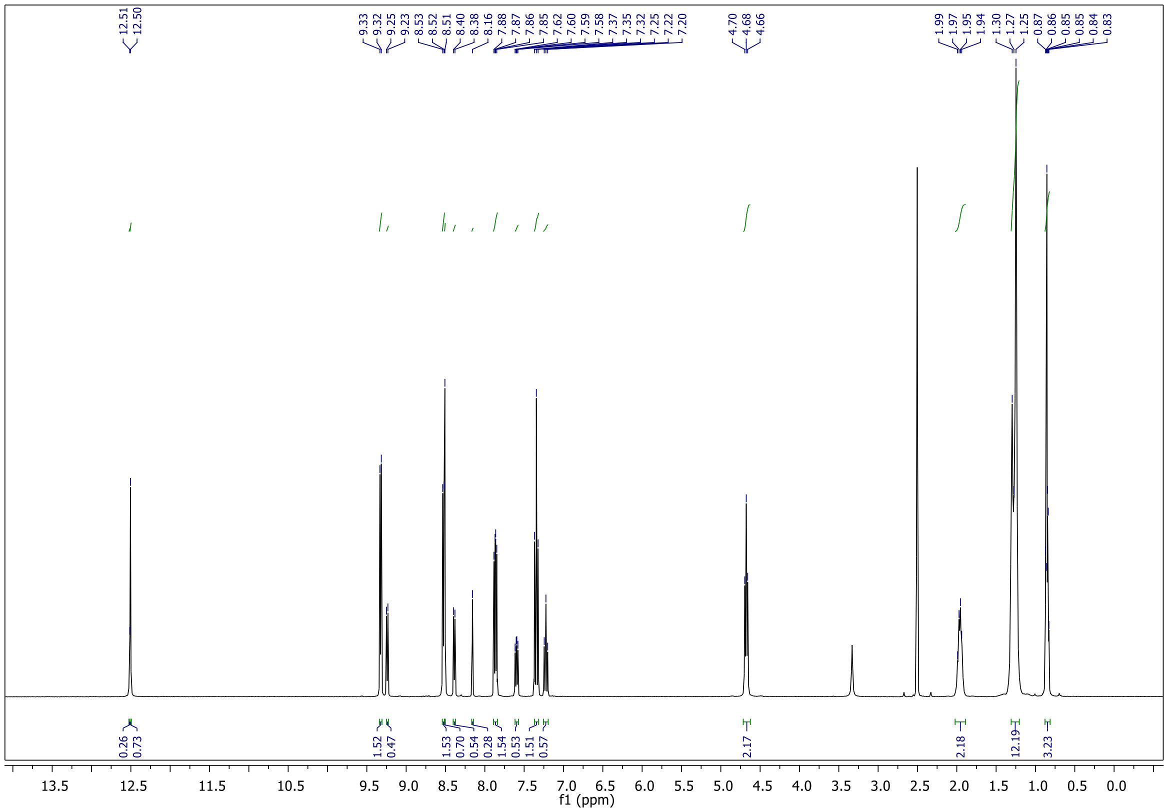

Figure S57. ^{1}HNMR of Compound 15

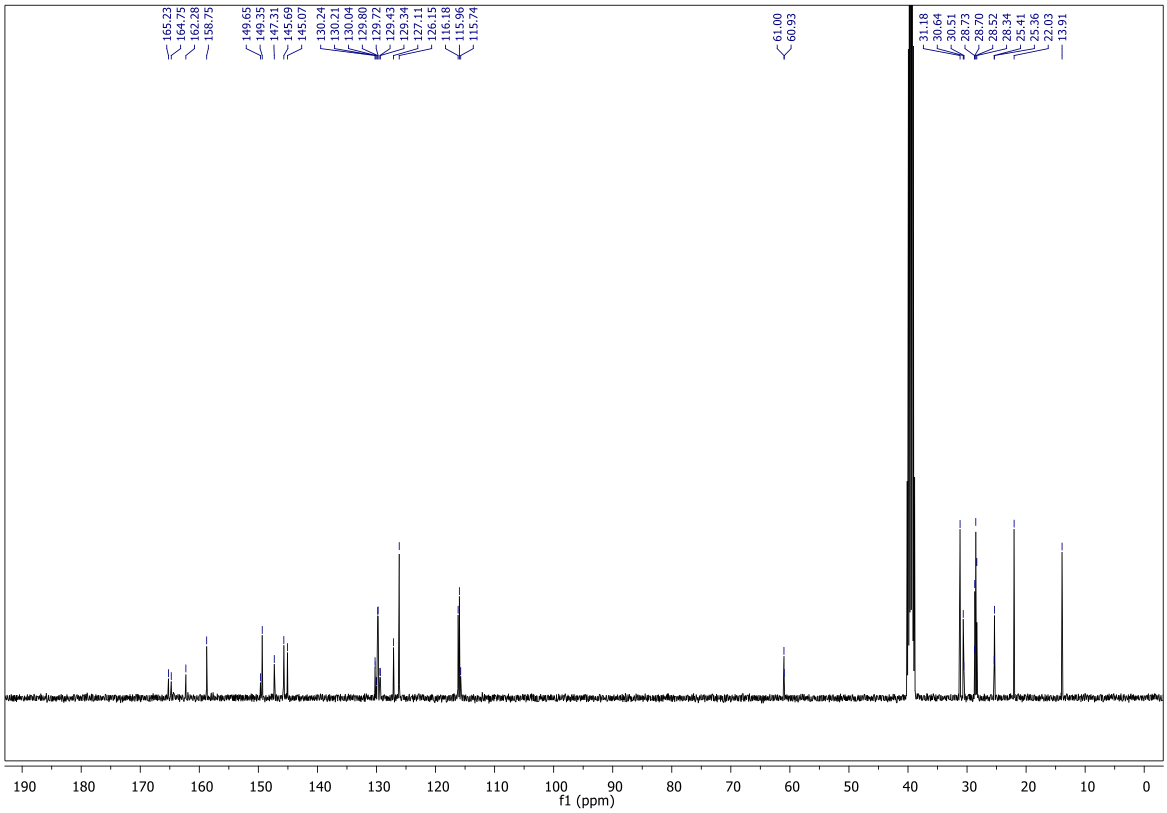

Figure S58. ^{13}CNMR of Compound 15

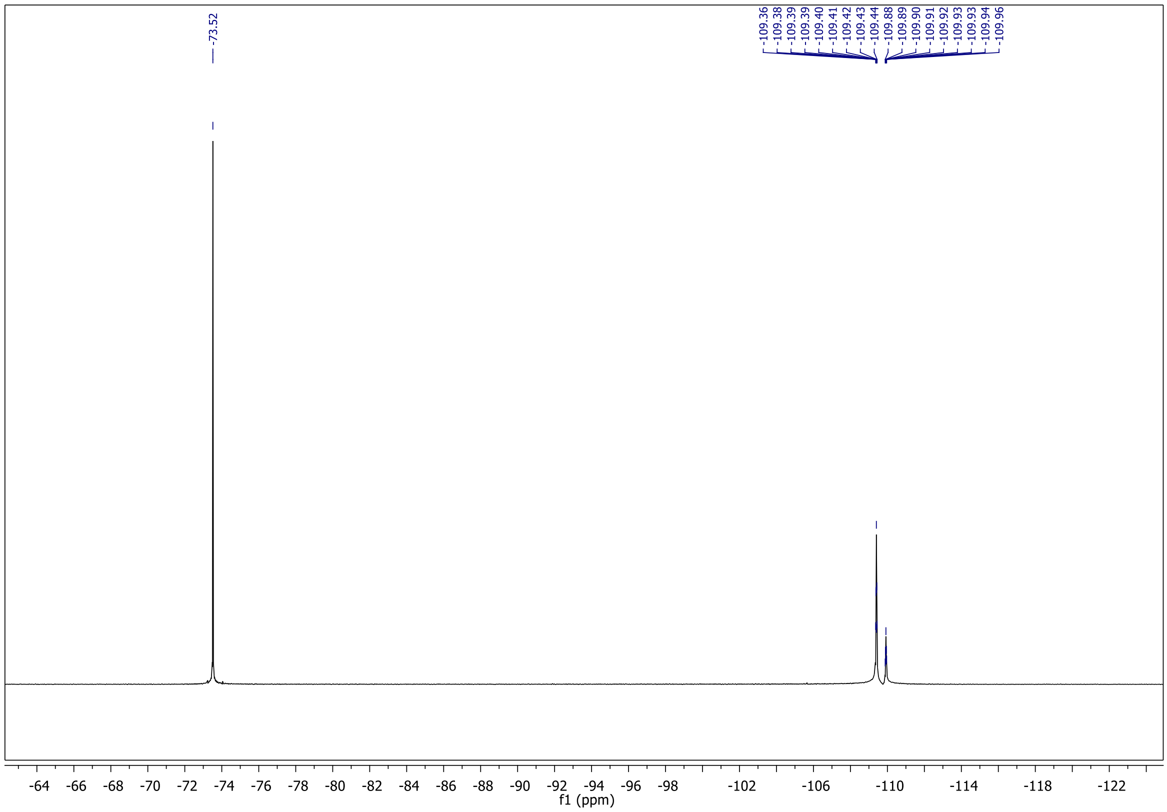

Figure S59. ^{19}FNMR of Compound 15

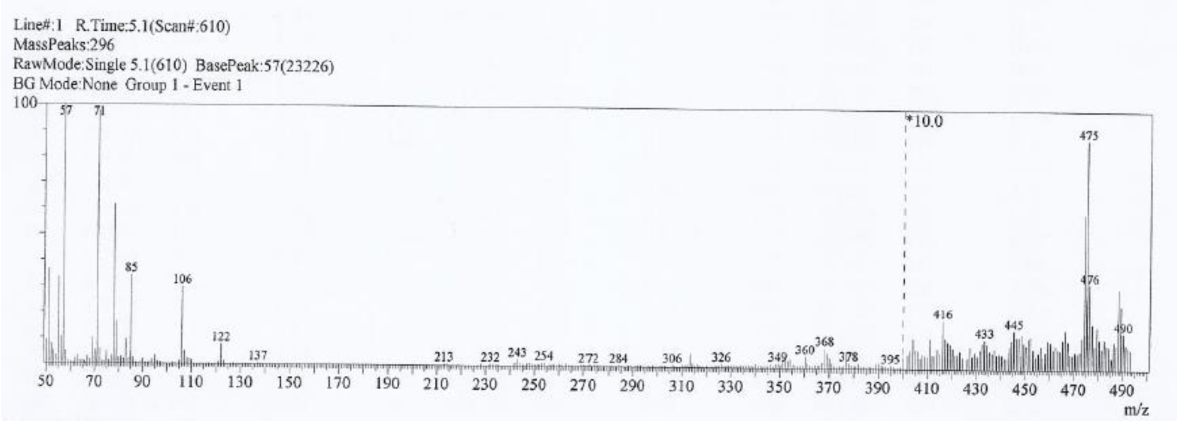

Figure S60. MS (ESI) of Compound 15

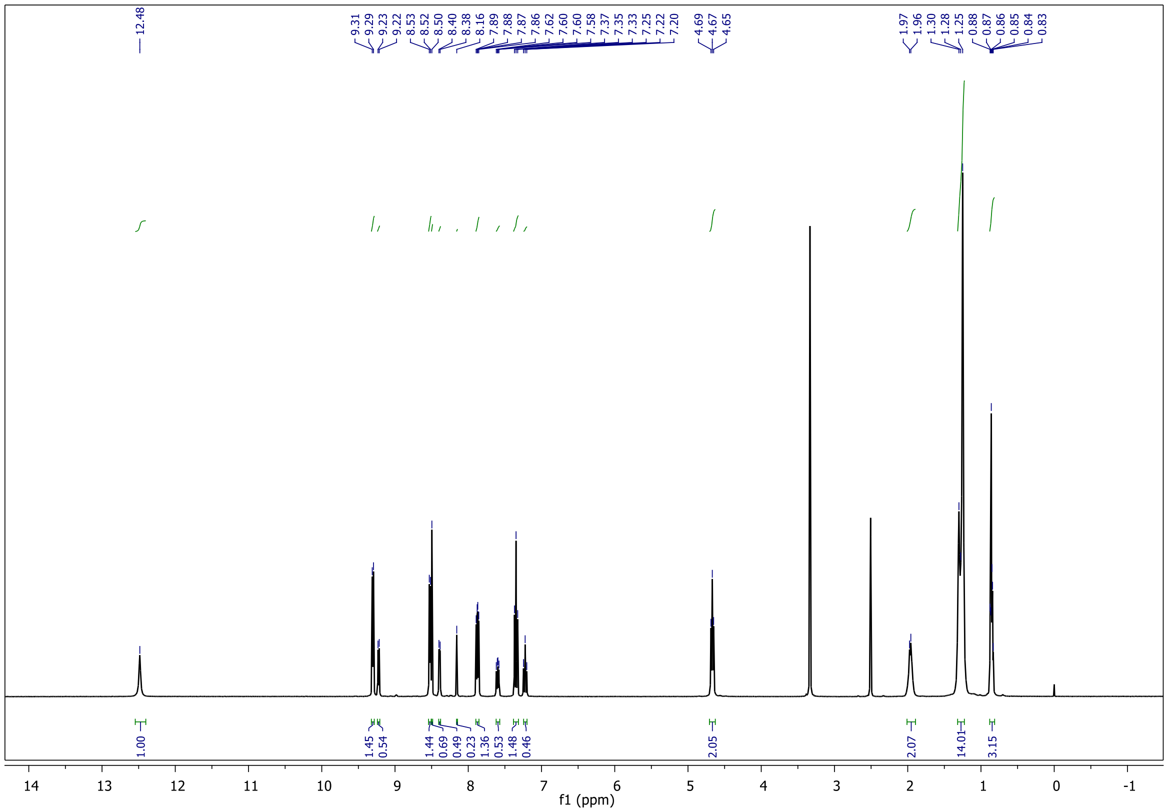

Figure S61. ^{1}HNMR of Compound 16

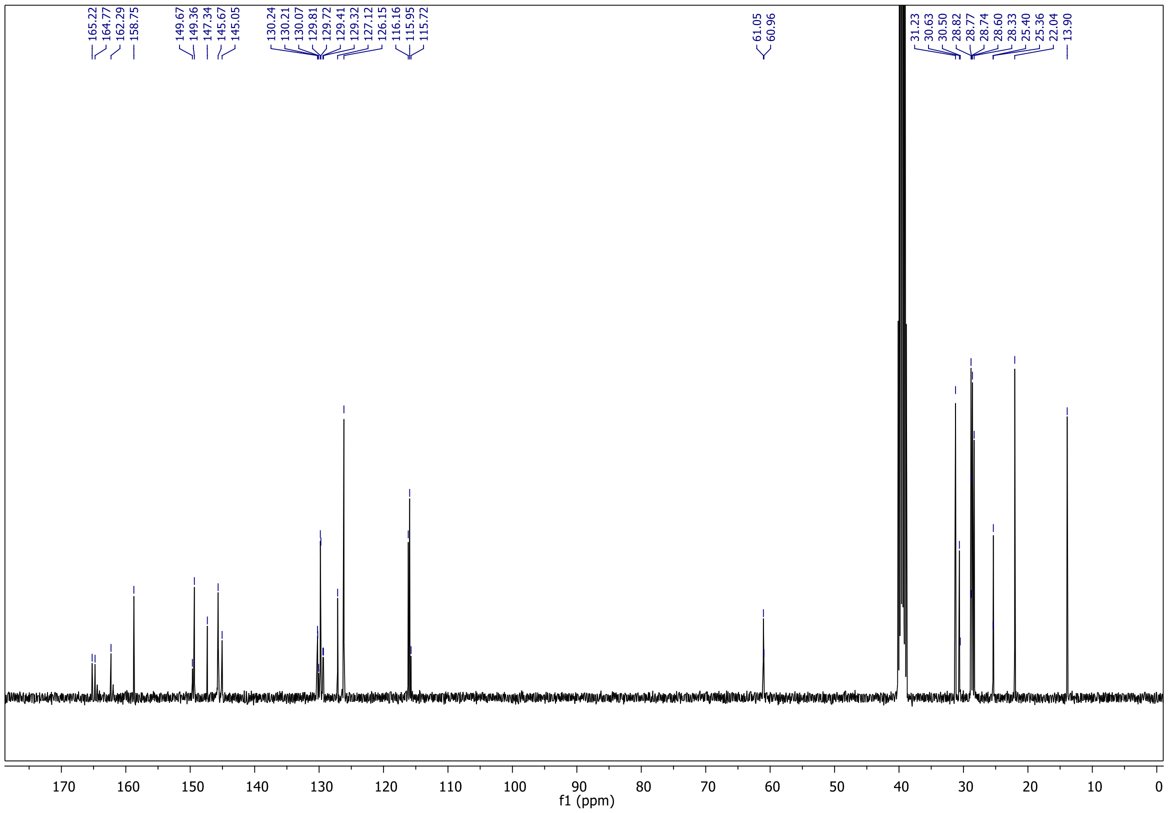

Figure S62. ^{13}CNMR of Compound 16

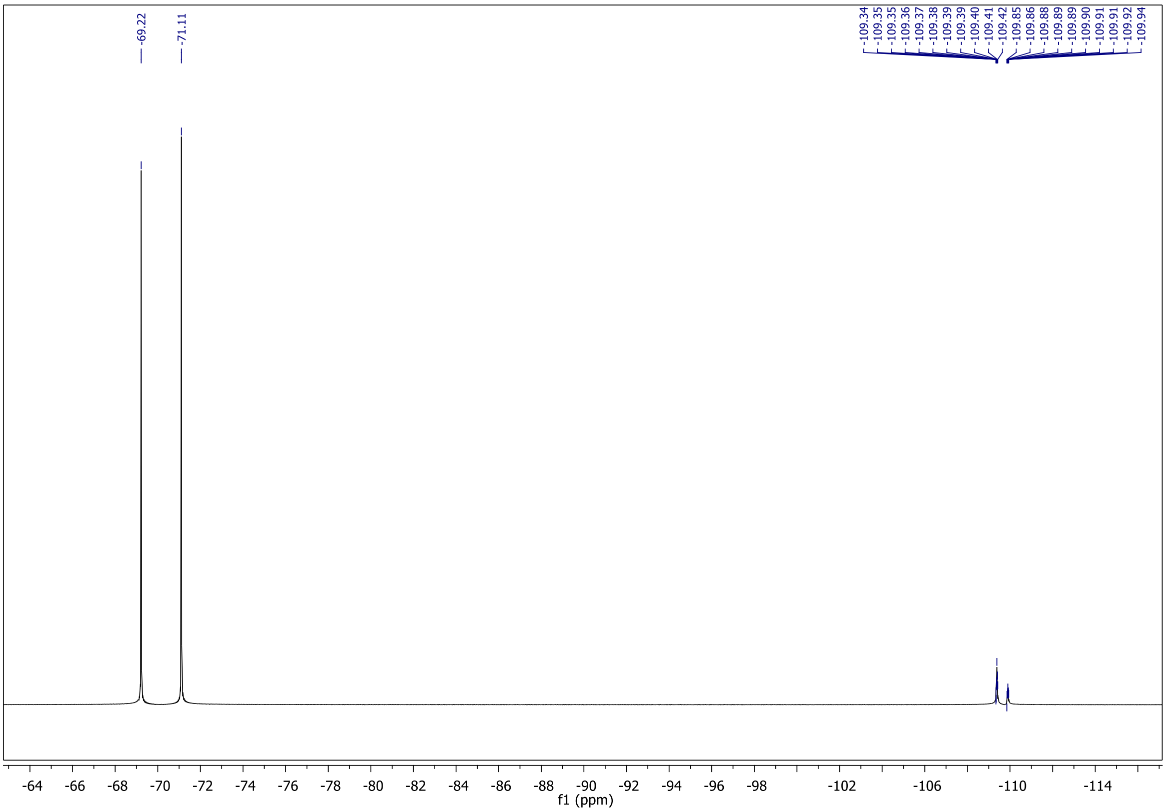

Figure S63. ^{19}FNMR of Compound 16

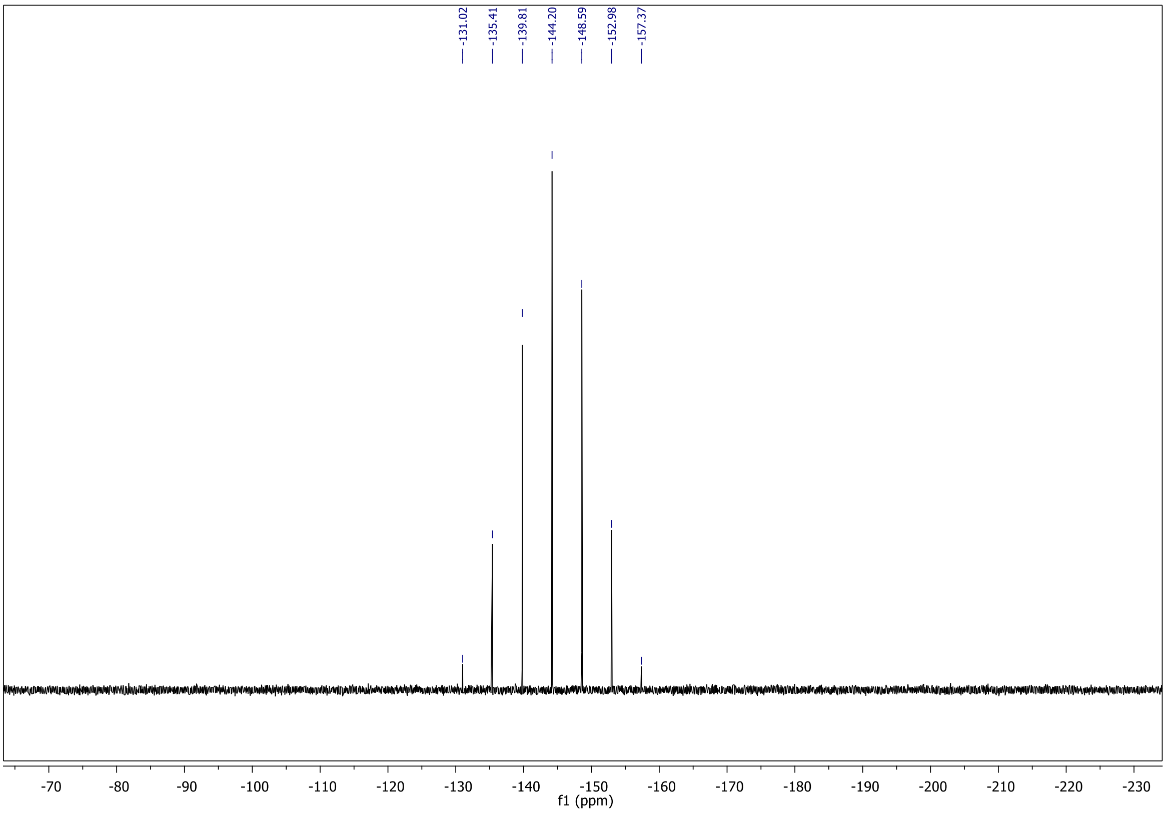

Figure S64. ^{31}PNMR of Compound 16

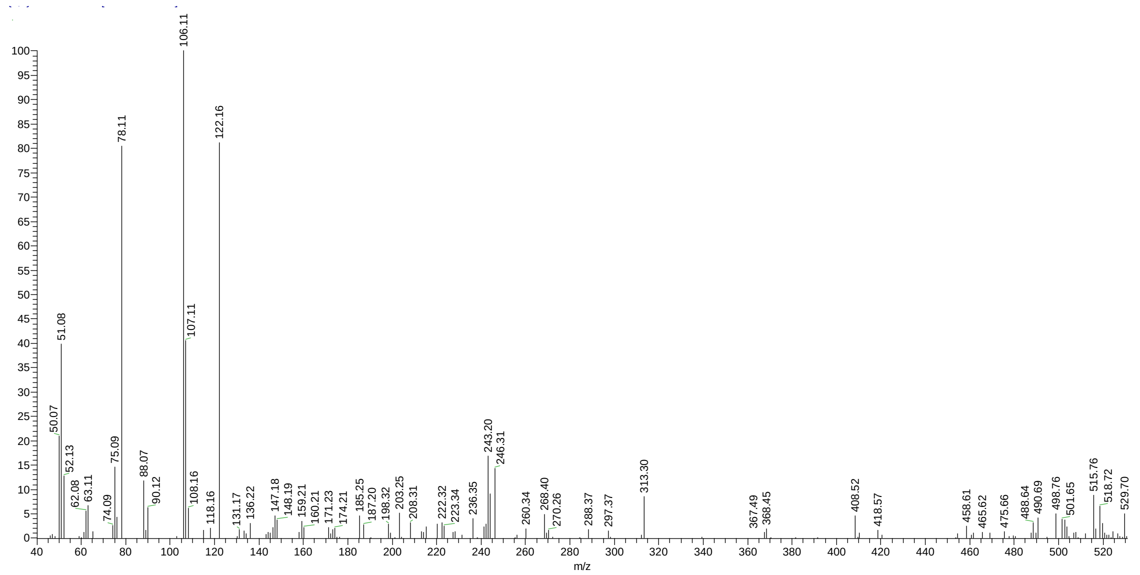

Figure S65. MS (ESI) of Compound 16

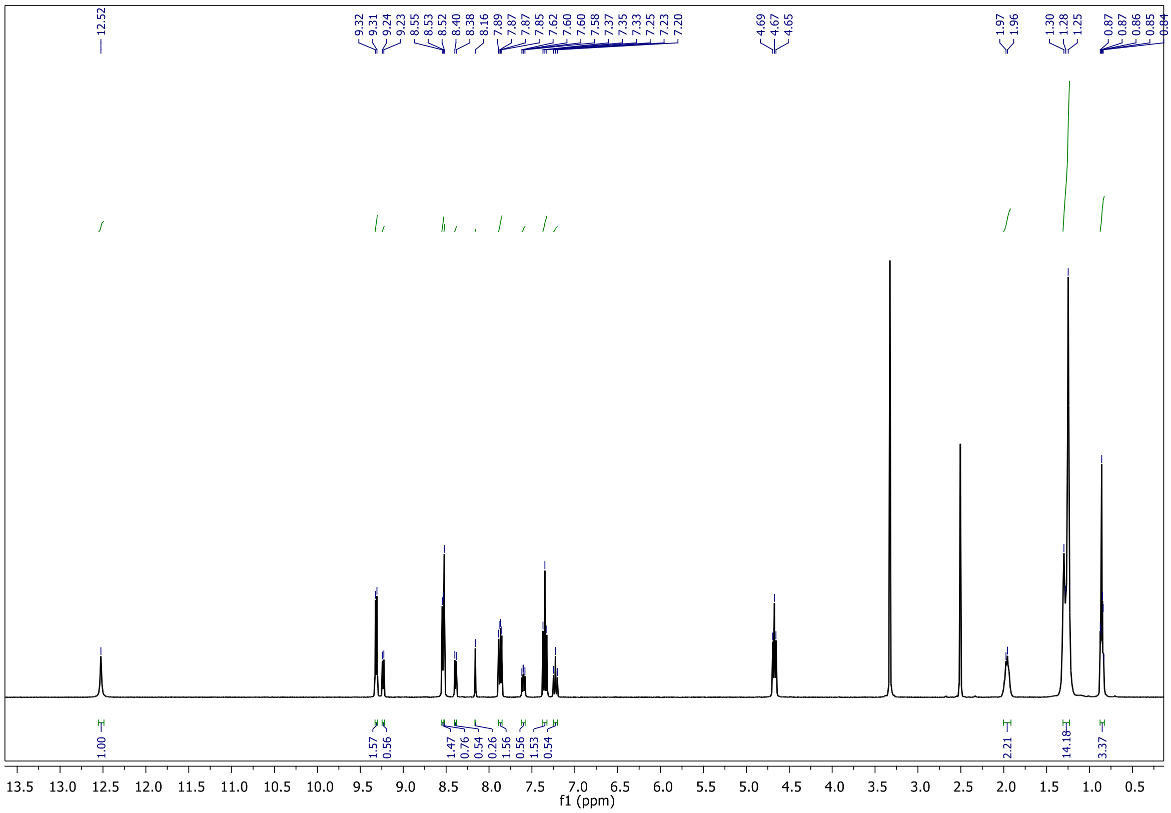

Figure S66. ^{1}HNMR of Compound 17

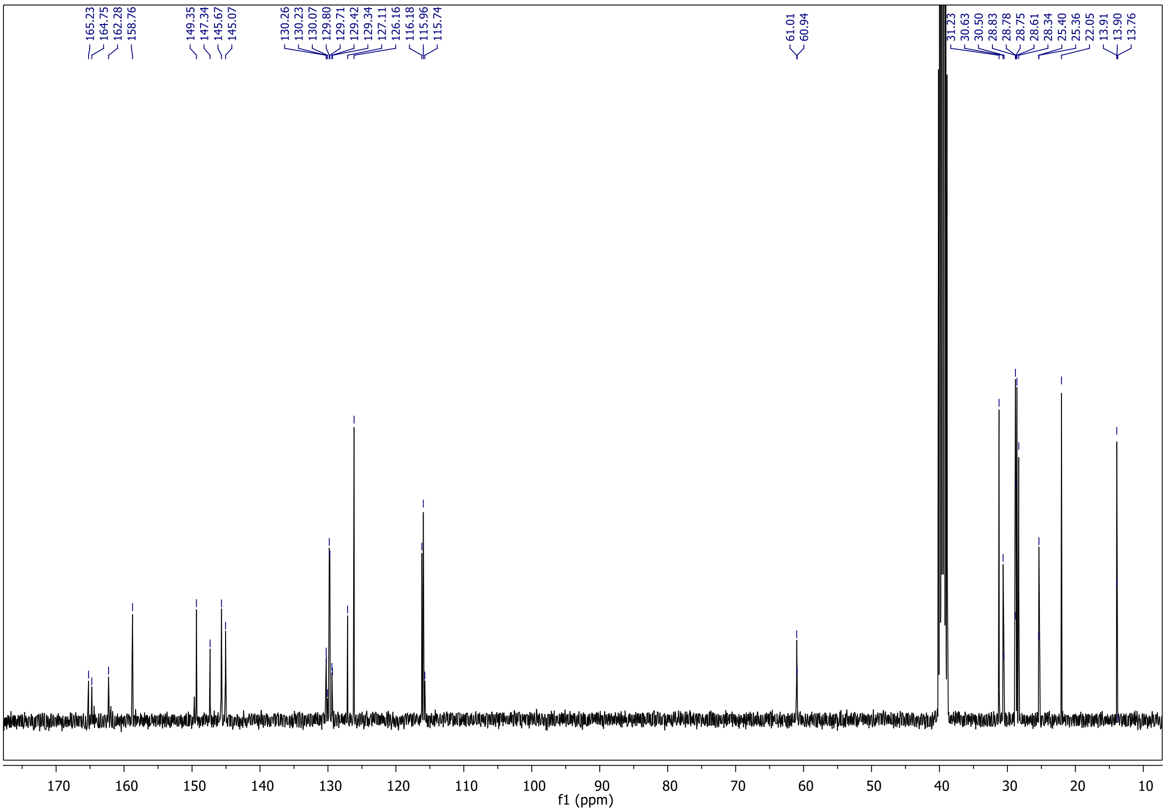

Figure S67. ¹³CNMR of Compound 17

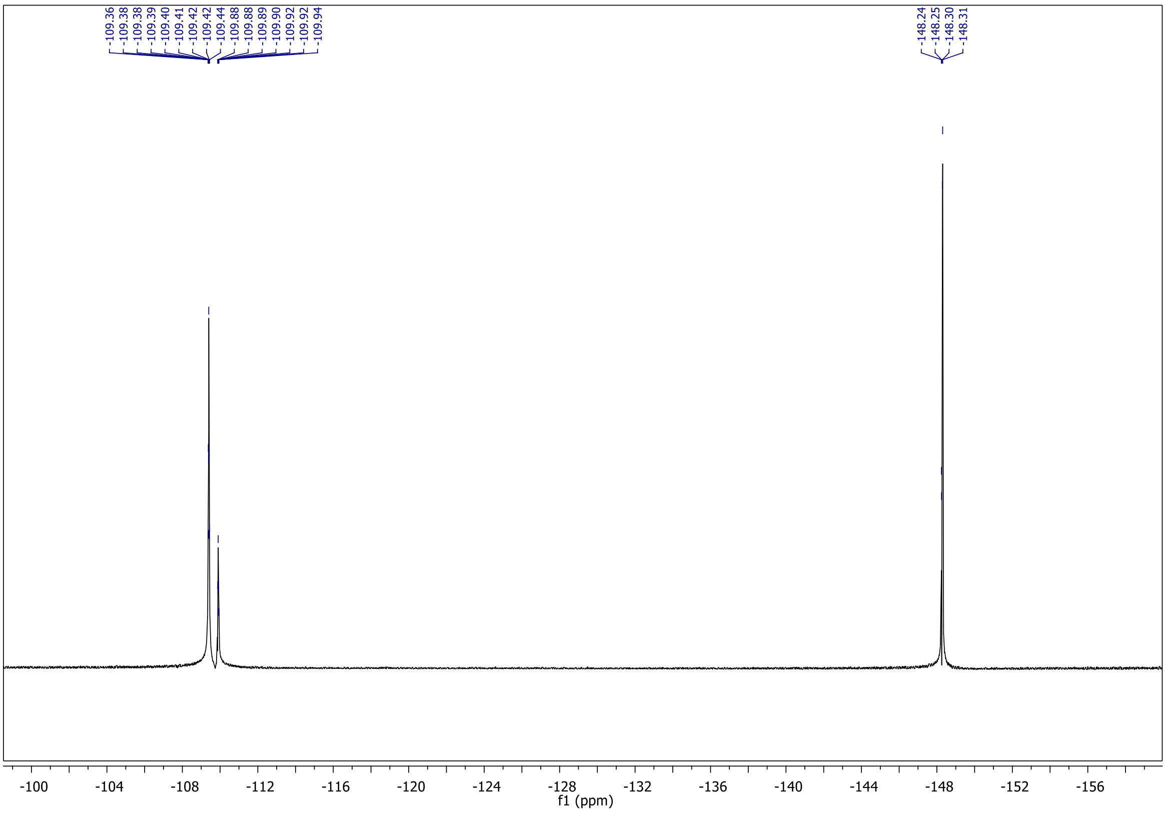

Figure S68. ¹⁹FNMR of Compound 17

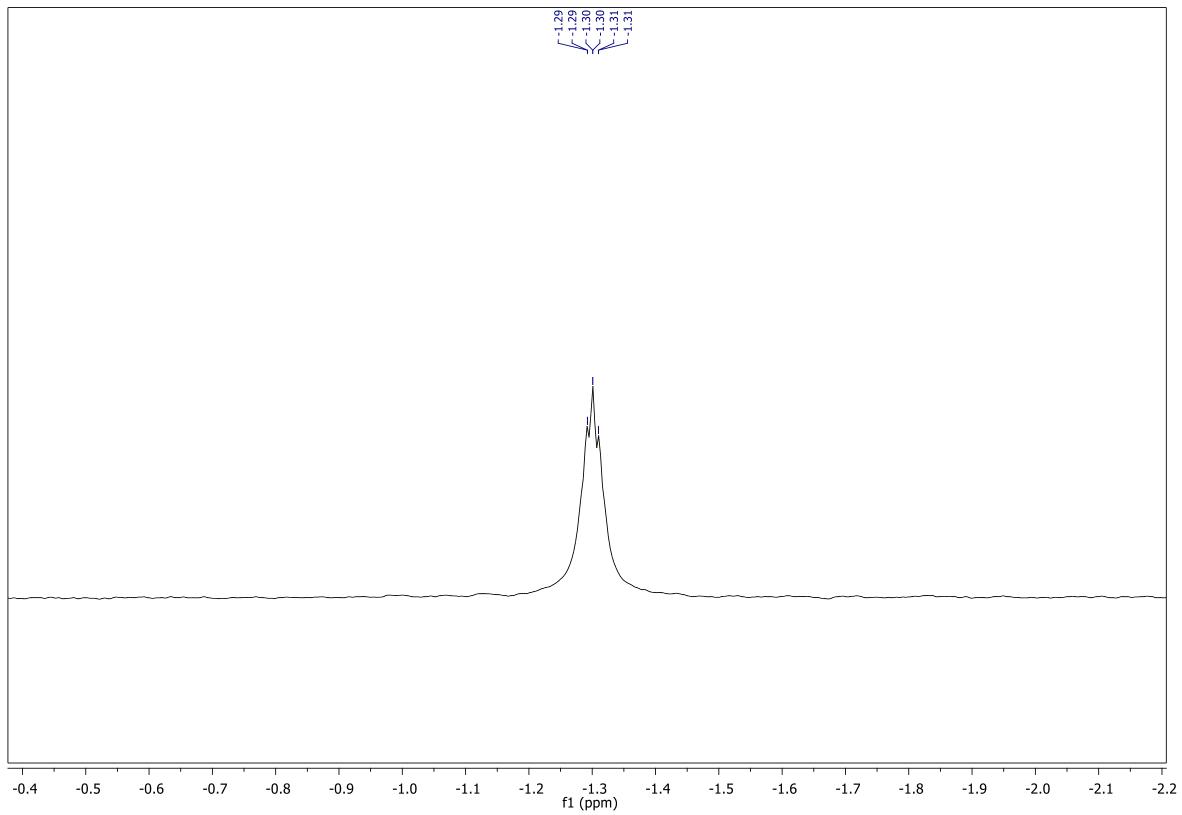

Figure S69. ^{11}BNMR of Compound 17

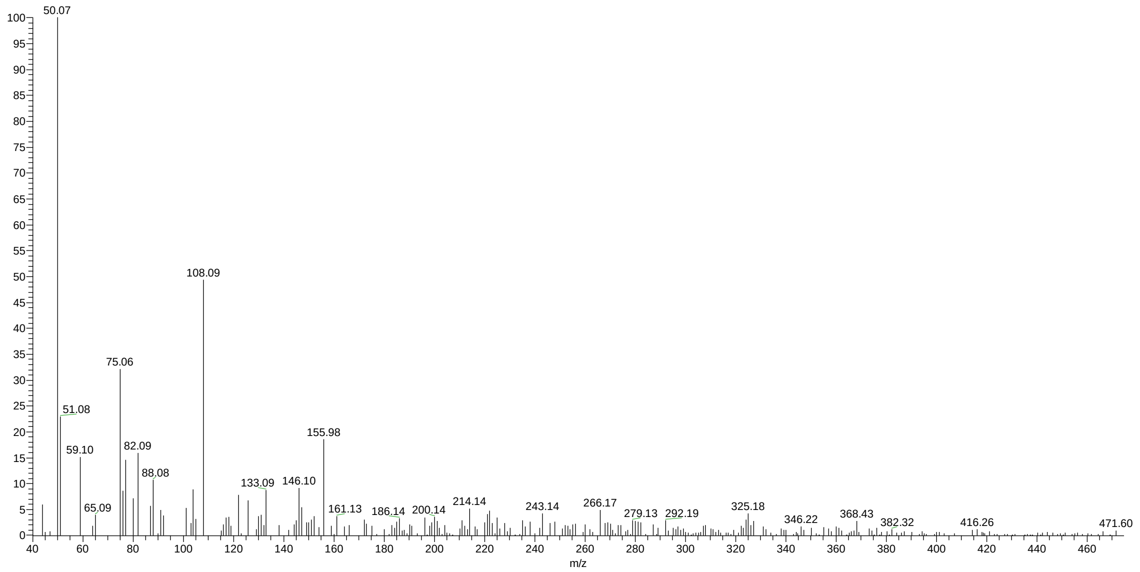

Figure S70. MS (ESI) of Compound 17

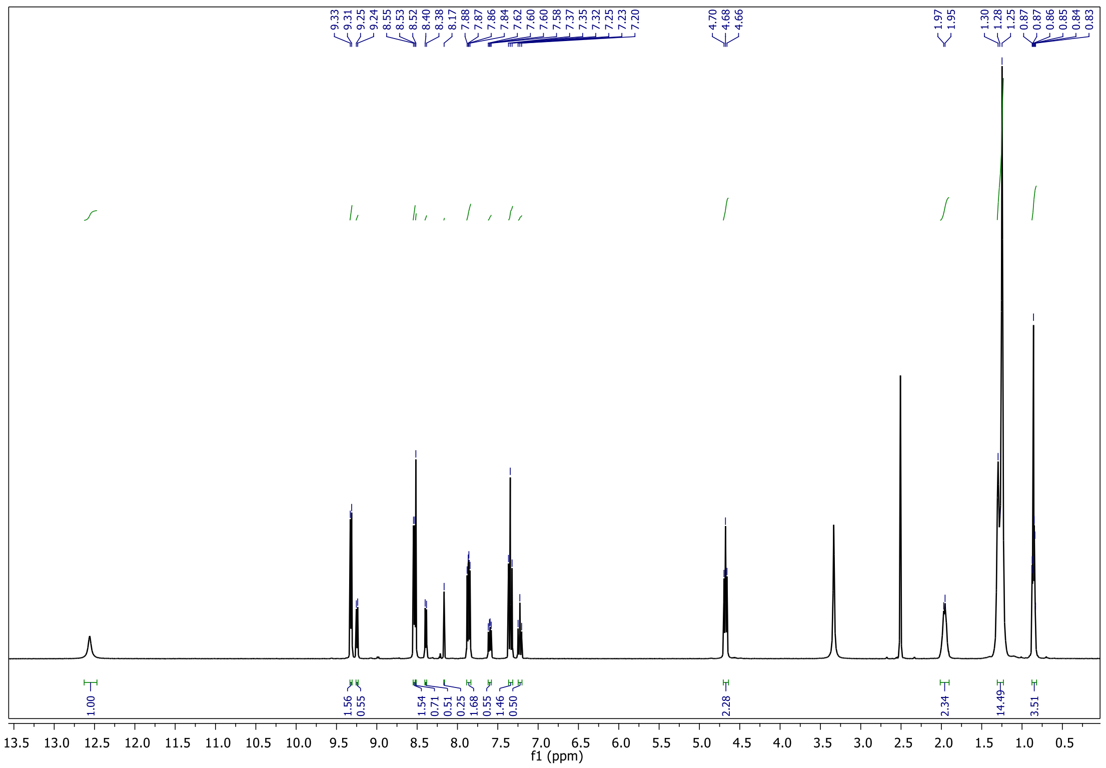

Figure S71. ^{1}HNMR of Compound 18

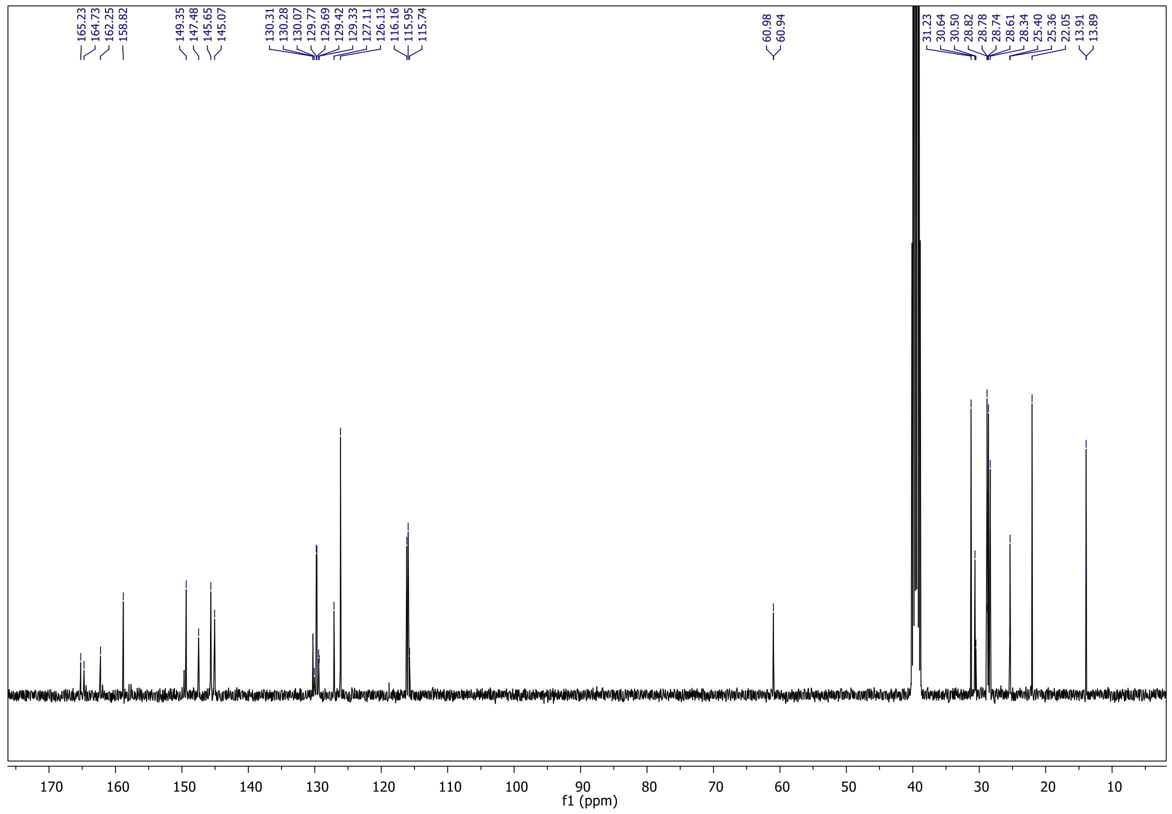

Figure S72. ^{13}CNMR of Compound 18

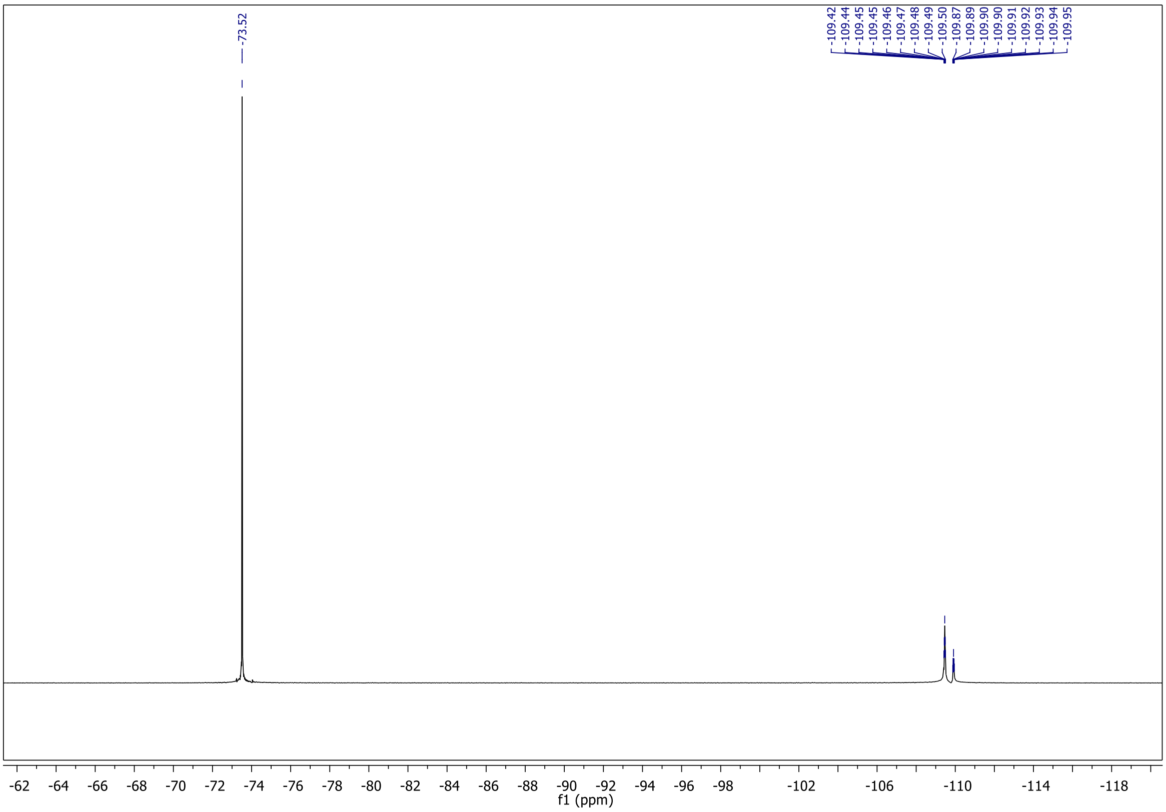

Figure S73. ^{19}FNMR of Compound 18

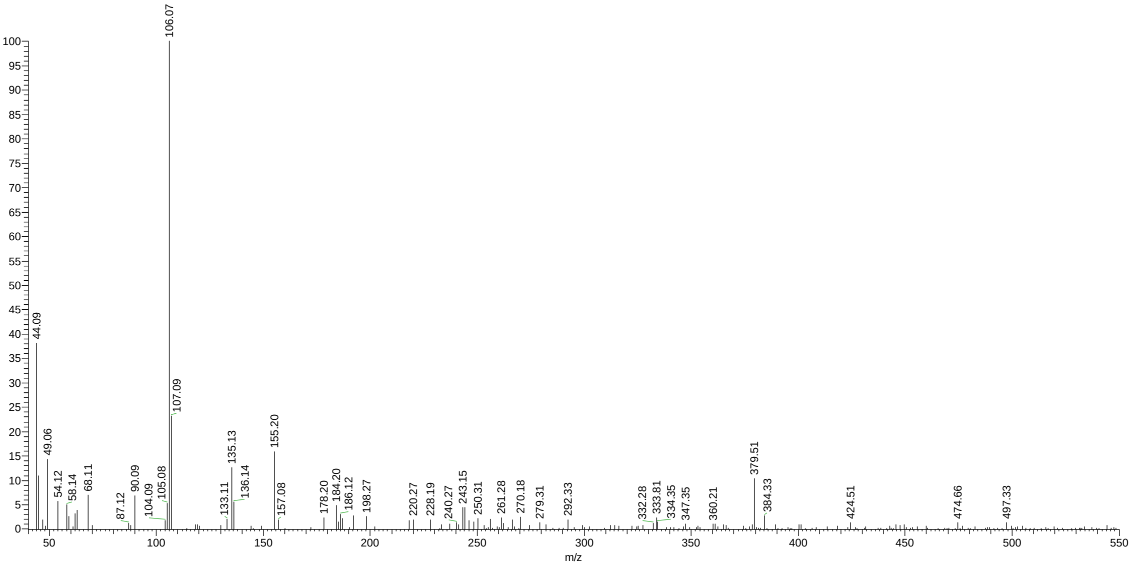

Figure S74. MS (ESI) of Compound 18

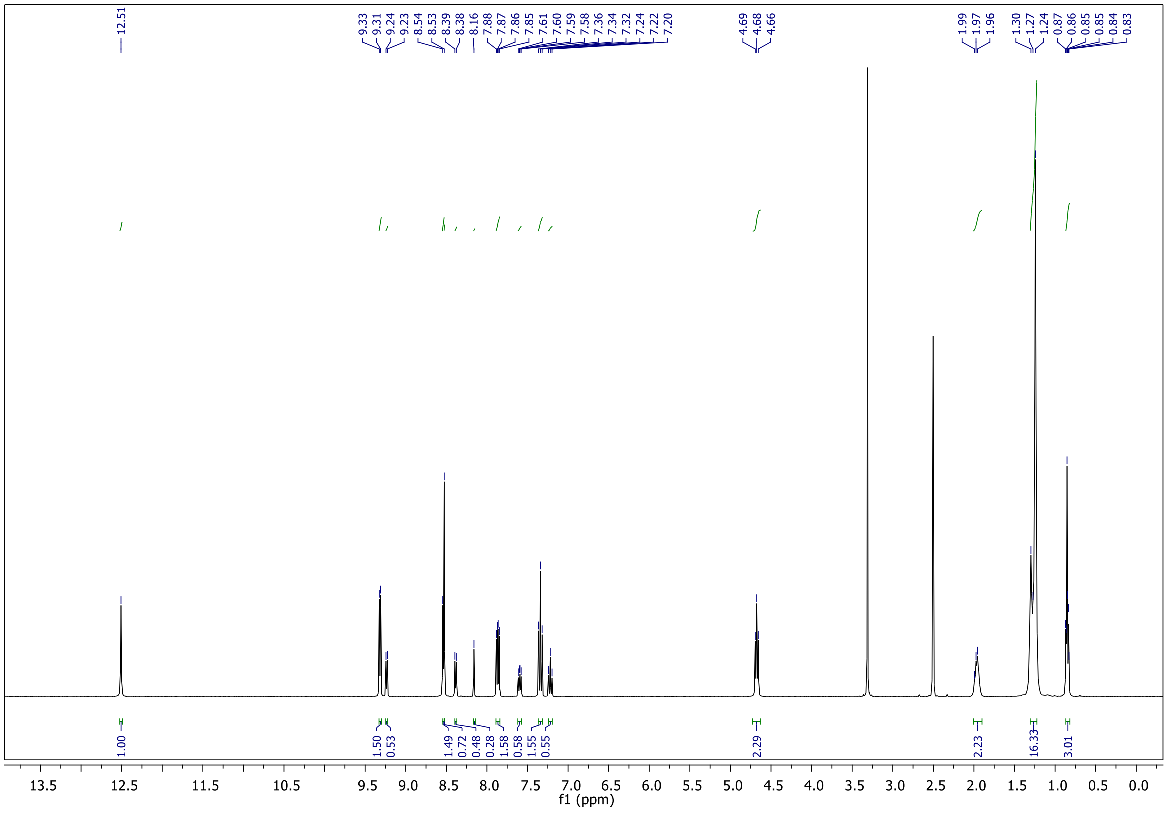

Figure S75. ^{1}HNMR of Compound 19

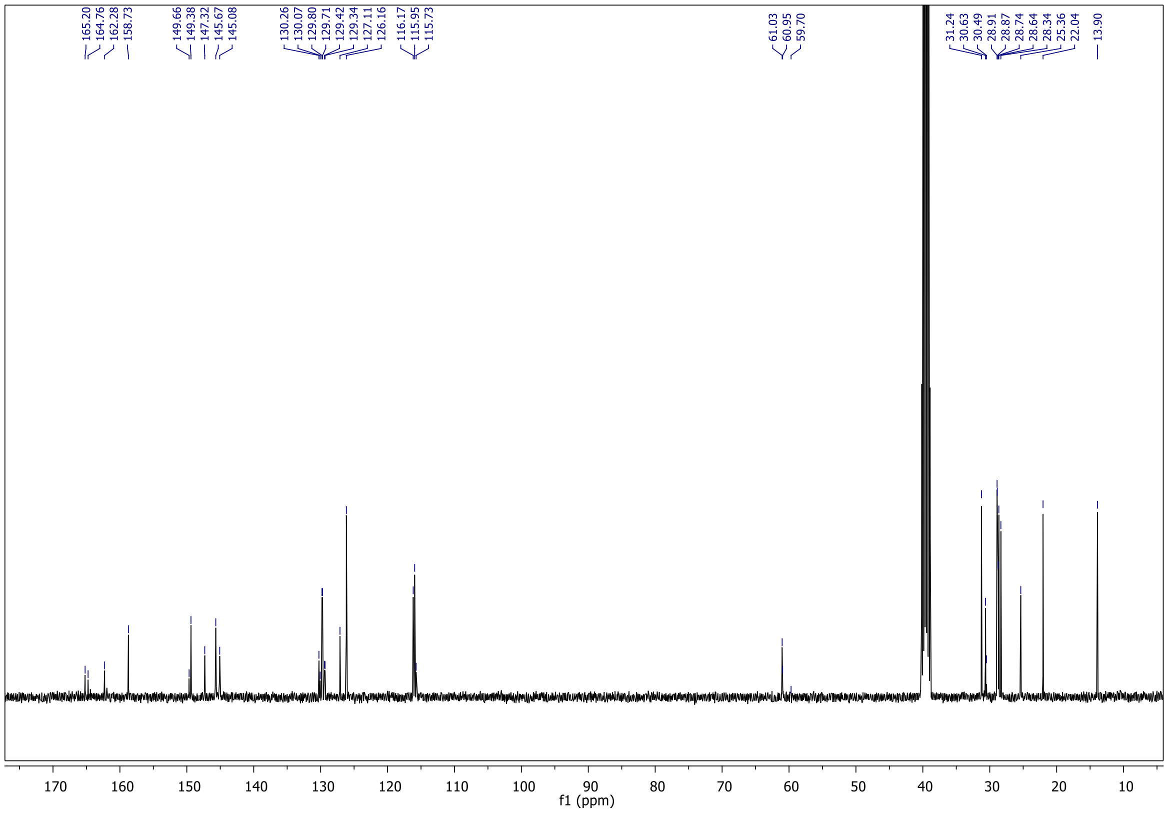

Figure S76. ^{13}CNMR of Compound 19

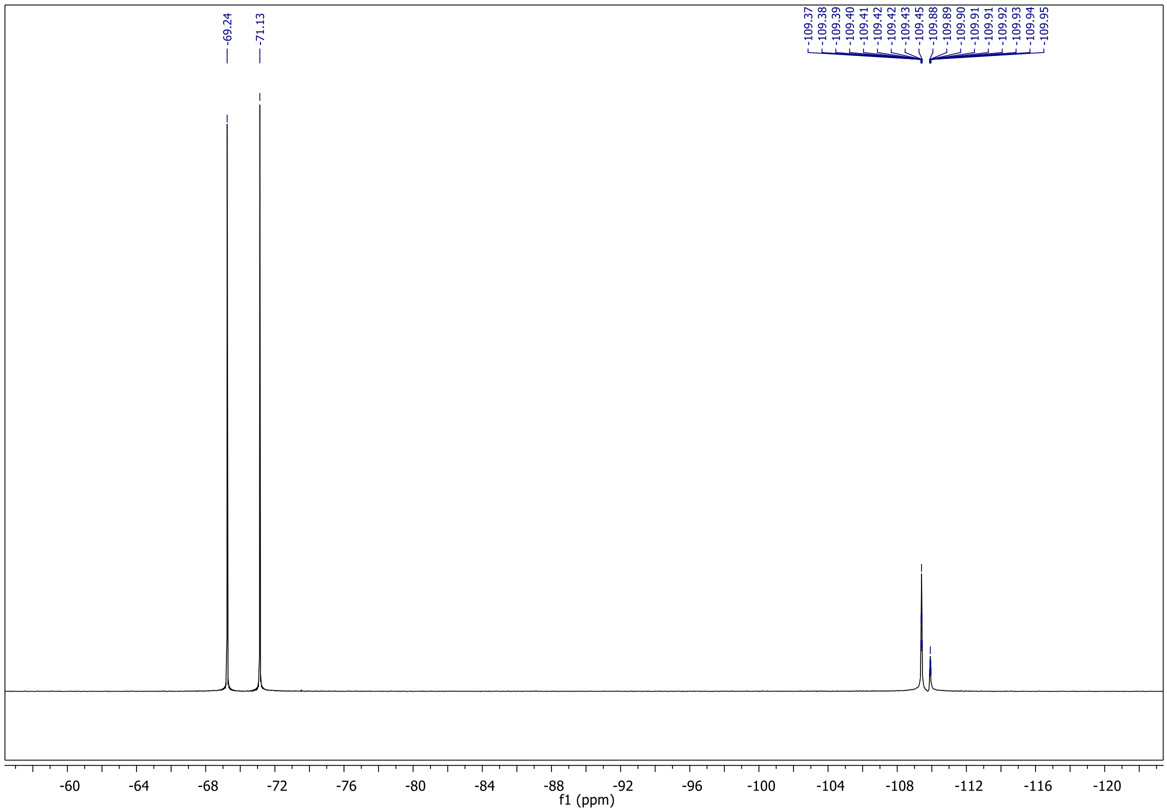

Figure S77. ^{19}FNMR of Compound 19

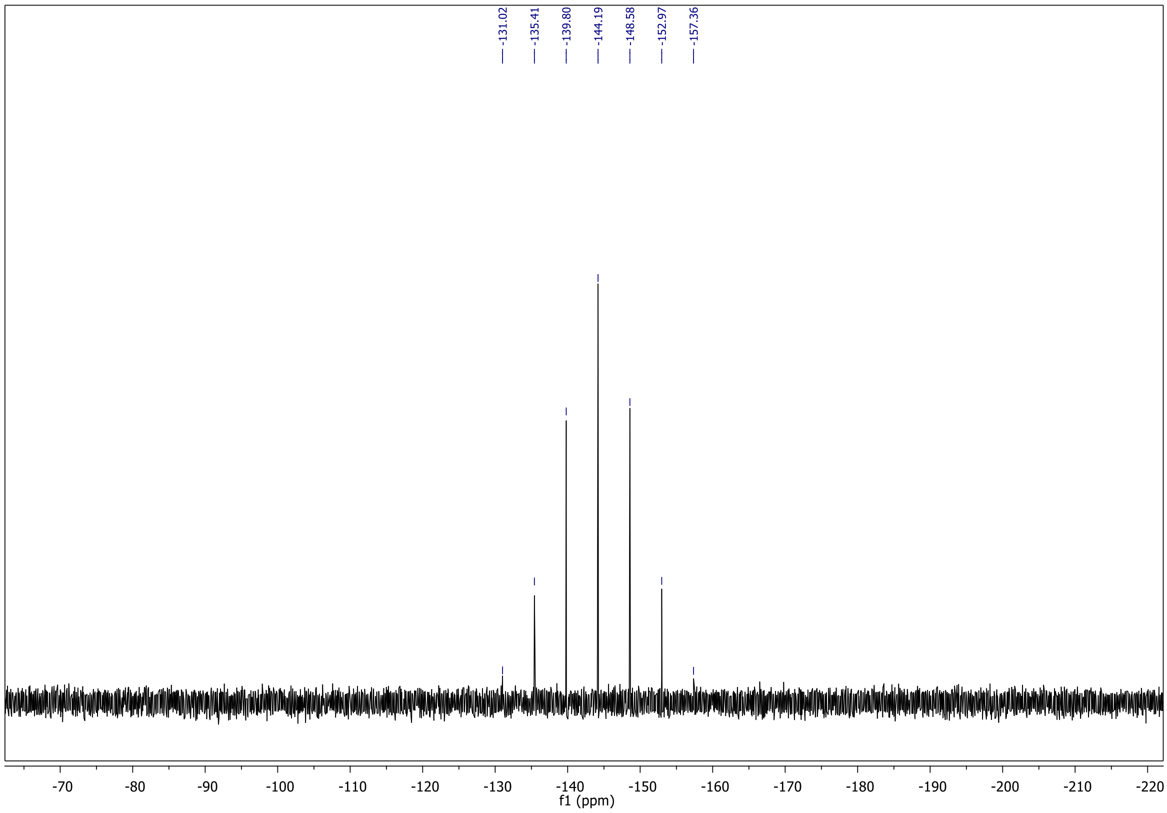

Figure S78. ^{31}PNMR of Compound 19

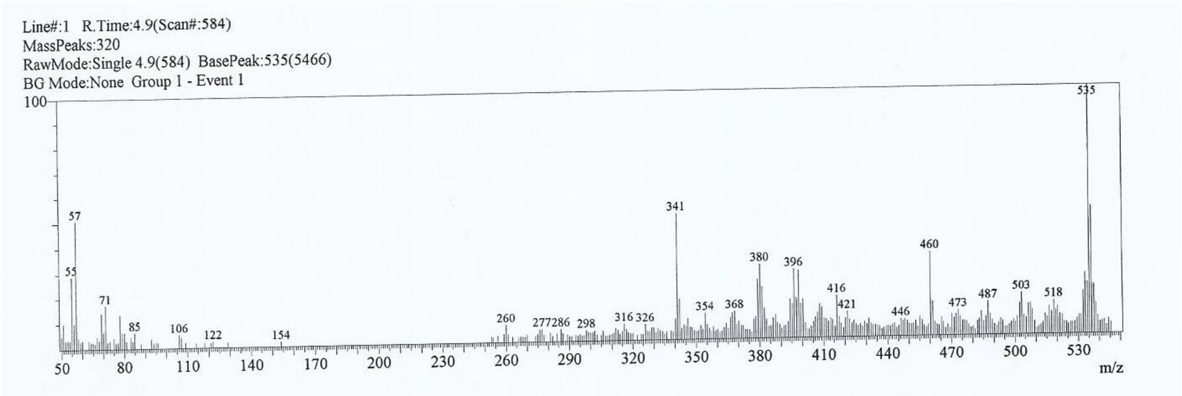

Figure S79. MS (ESI) of Compound 19

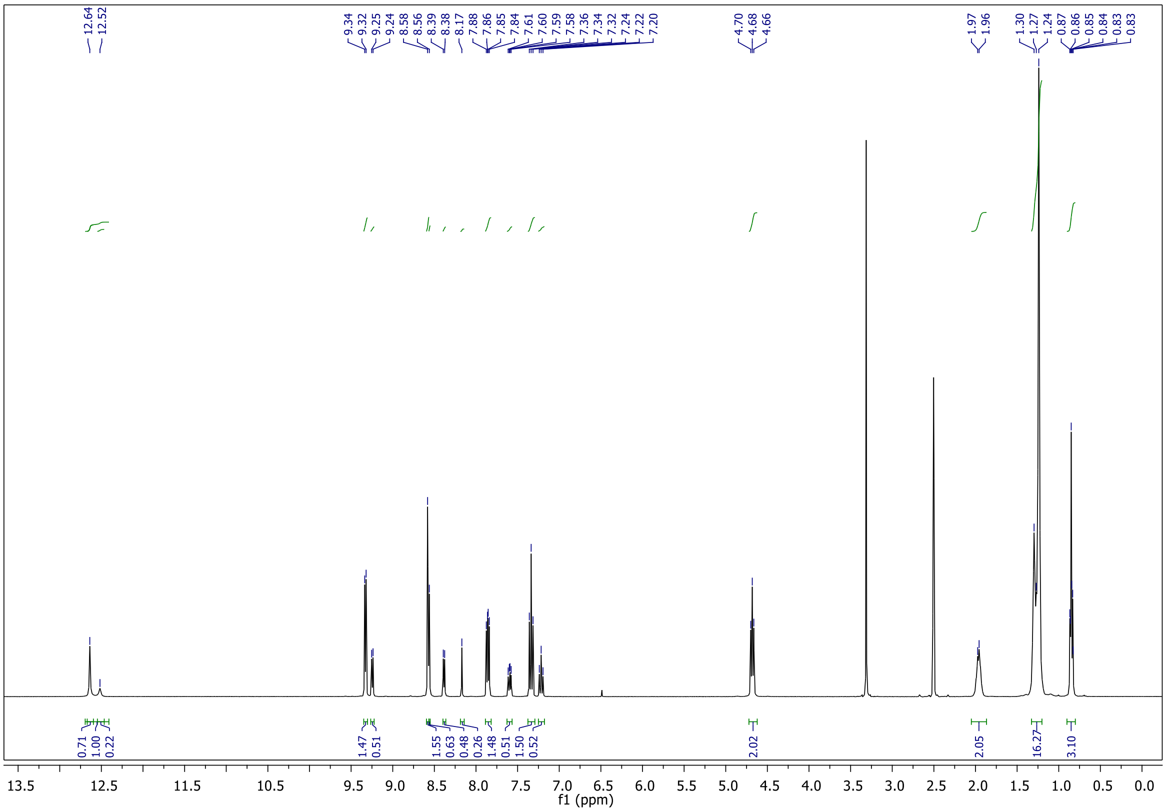

Figure S80. ^{1}HNMR of Compound 20

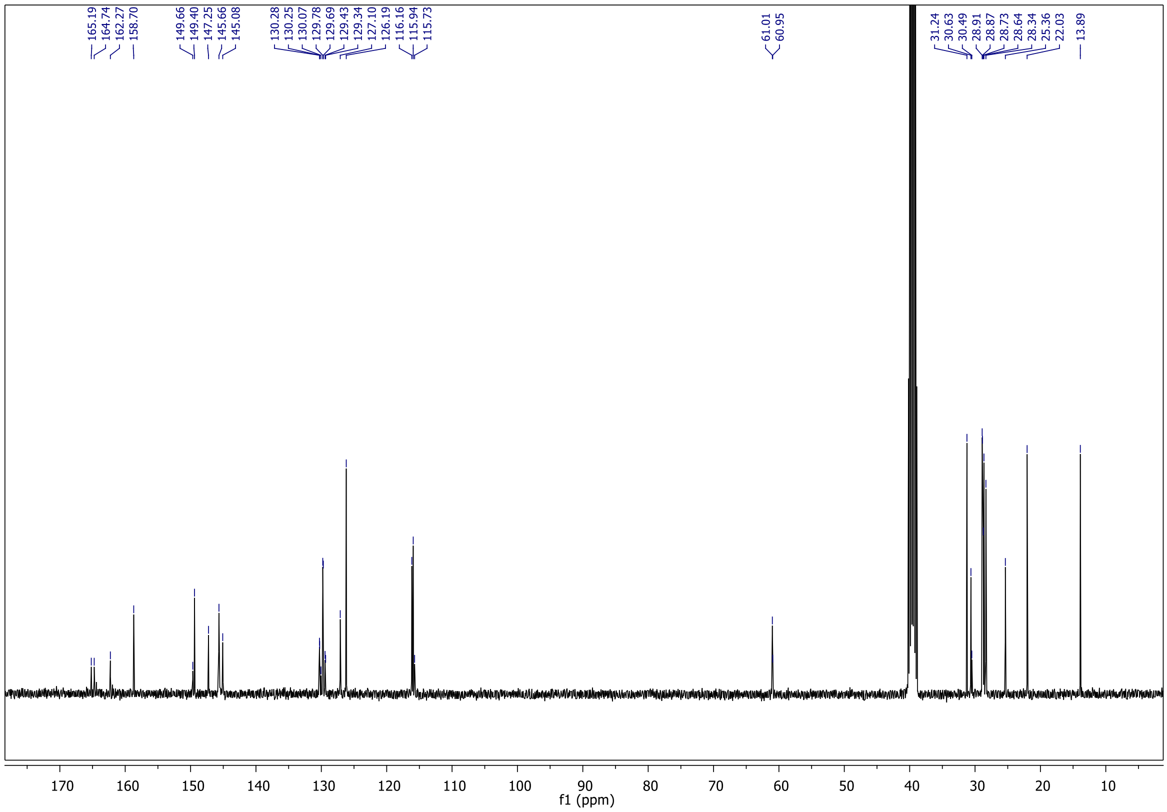

Figure S81. ^{13}CNMR of Compound 20

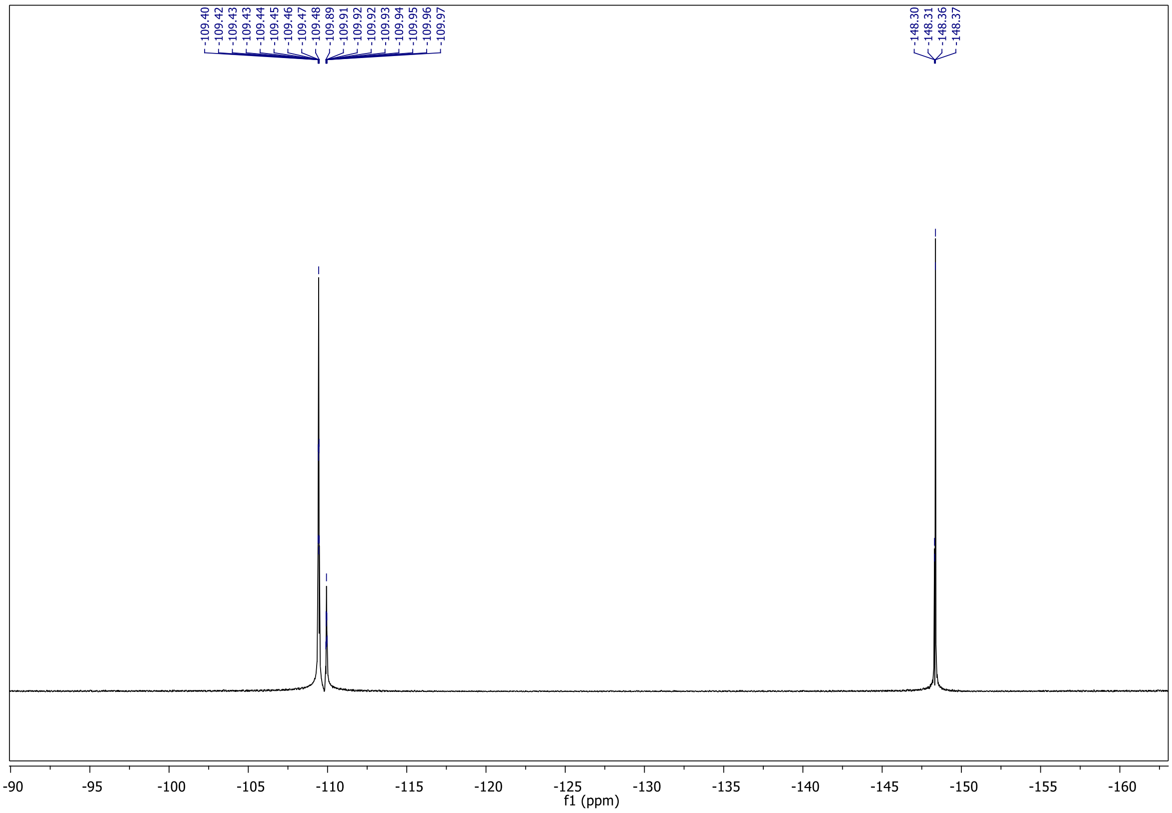

Figure S82. ^{19}FNMR of Compound 20

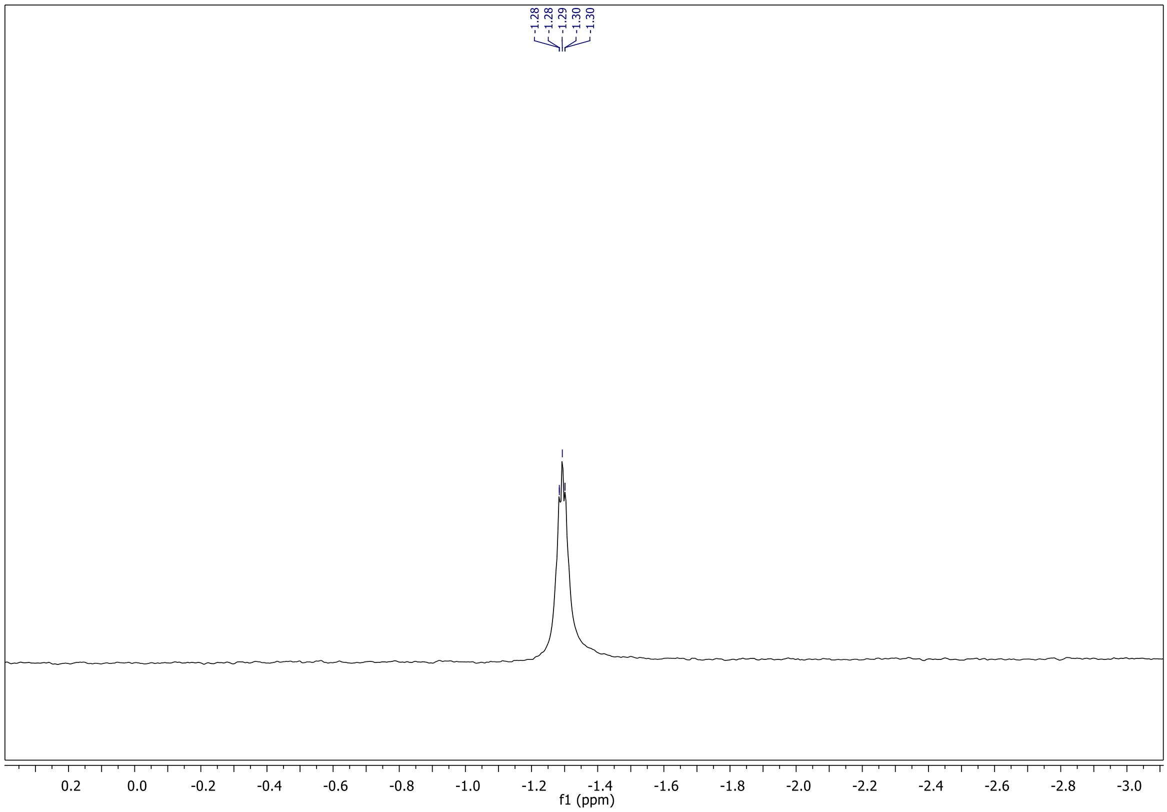

Figure S83. ^{11}BNMR of Compound 20

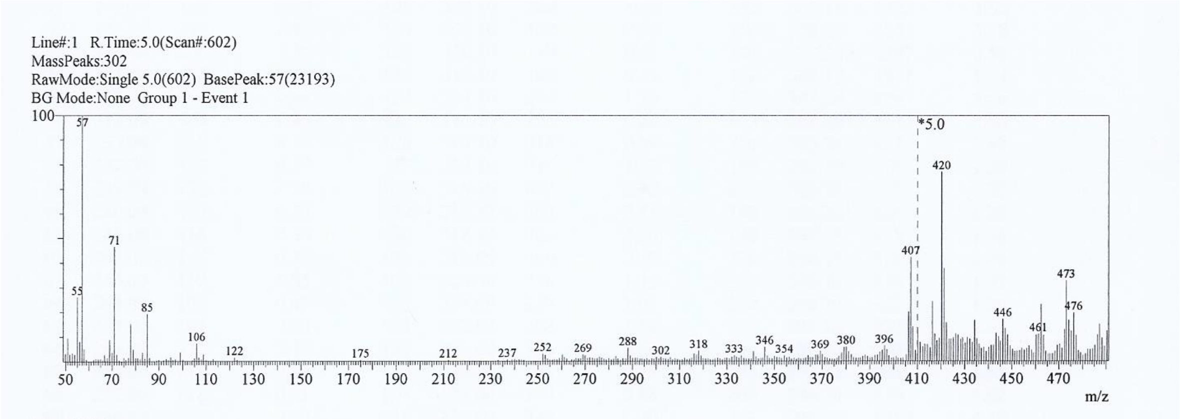

Figure S84. MS (ESI) of Compound 20

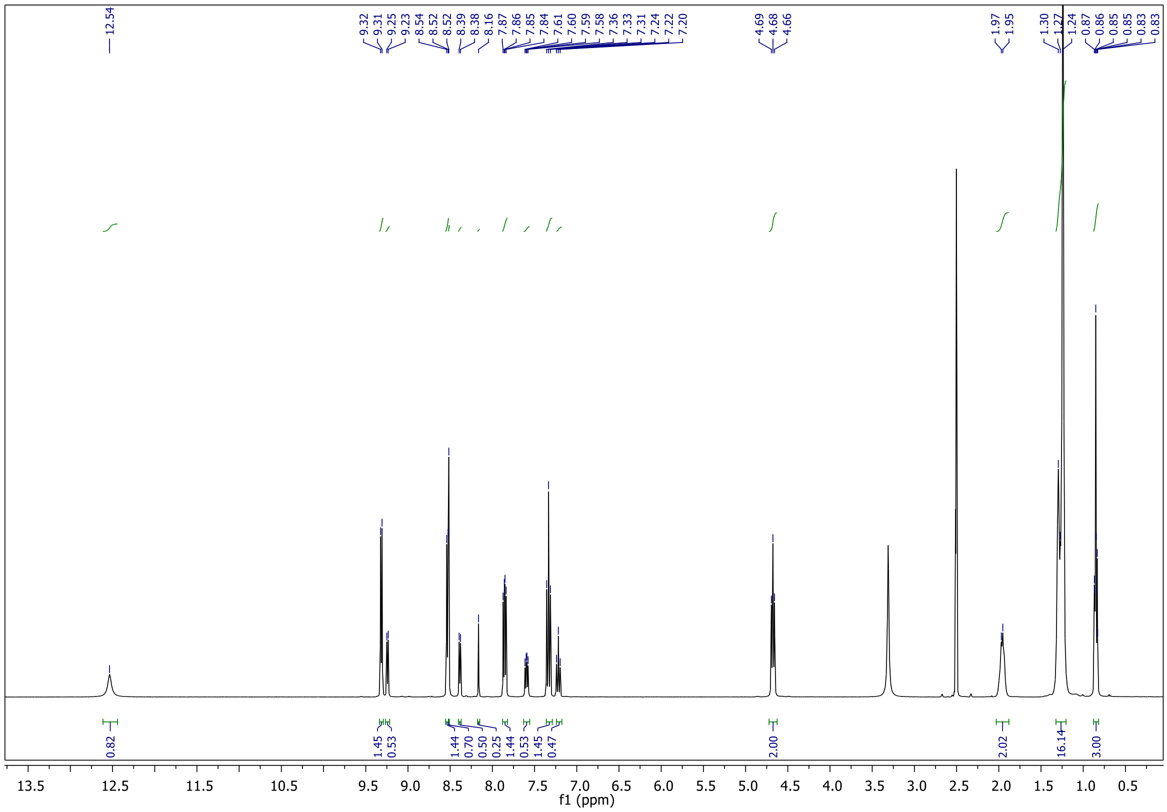

Figure S85. ^{1}HNMR of Compound 21

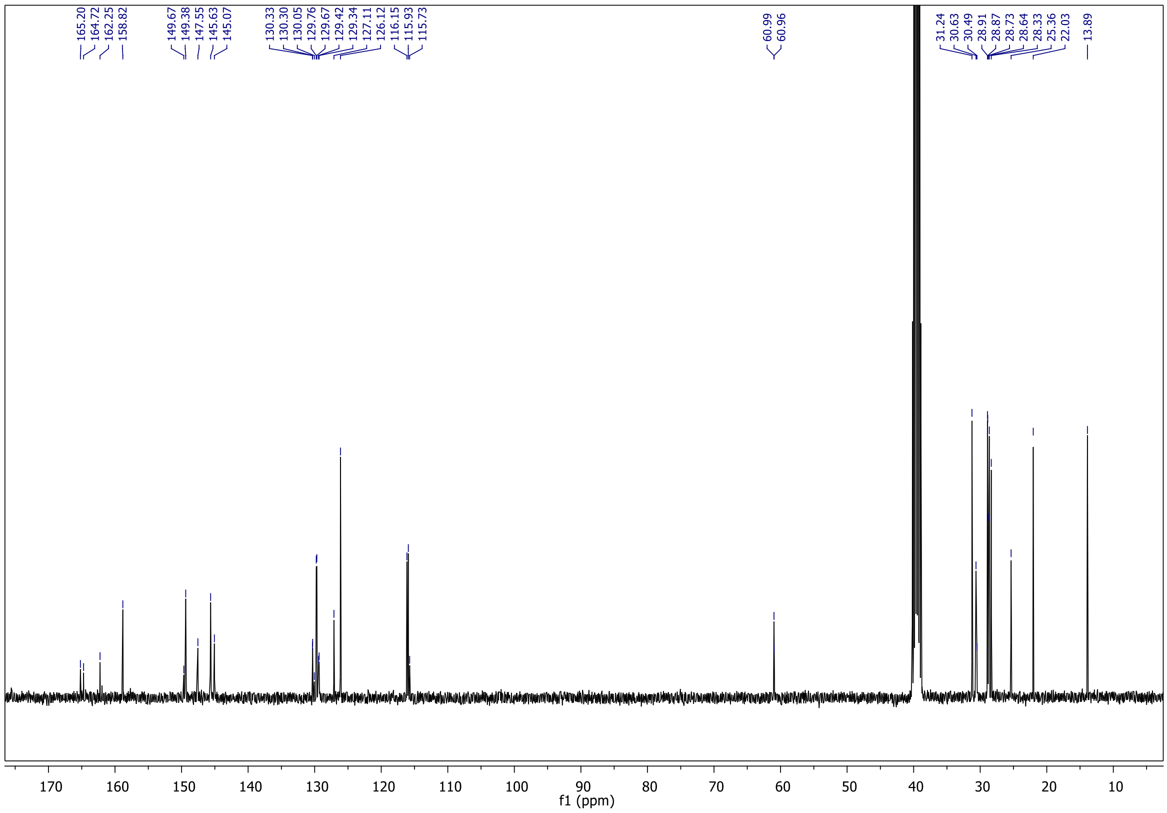

Figure S86. ^{13}CNMR of Compound 21

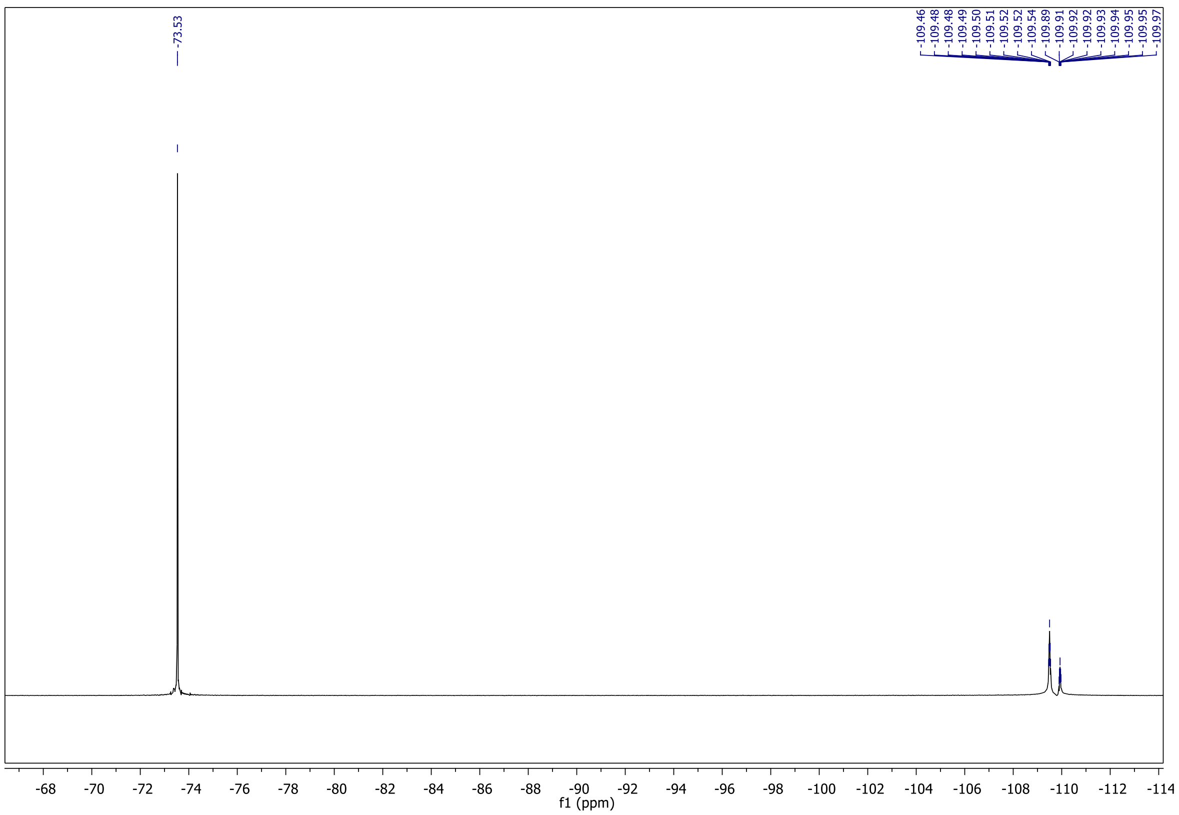

Figure S87. ^{19}FNMR of Compound 21

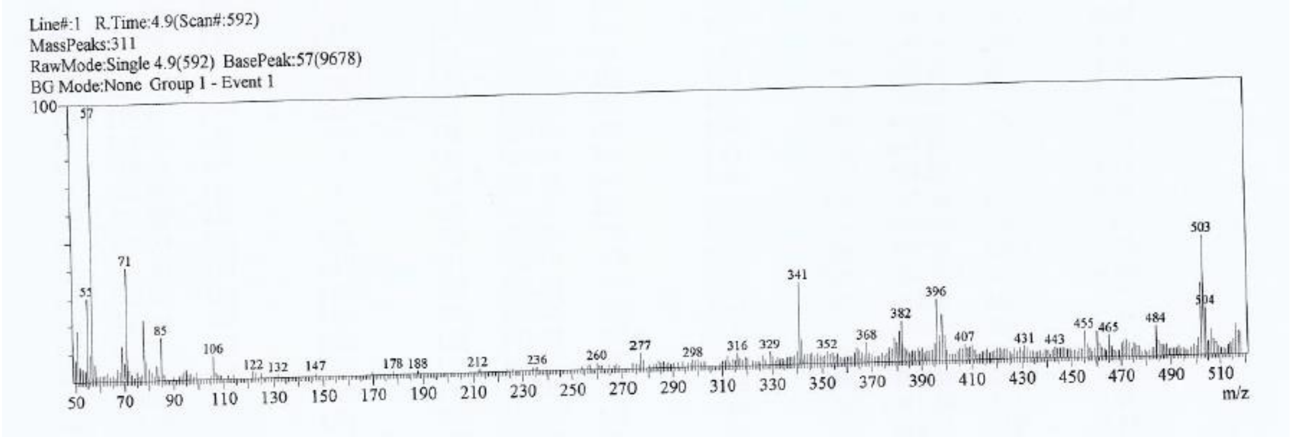

Figure S88. MS (ESI) of Compound 21

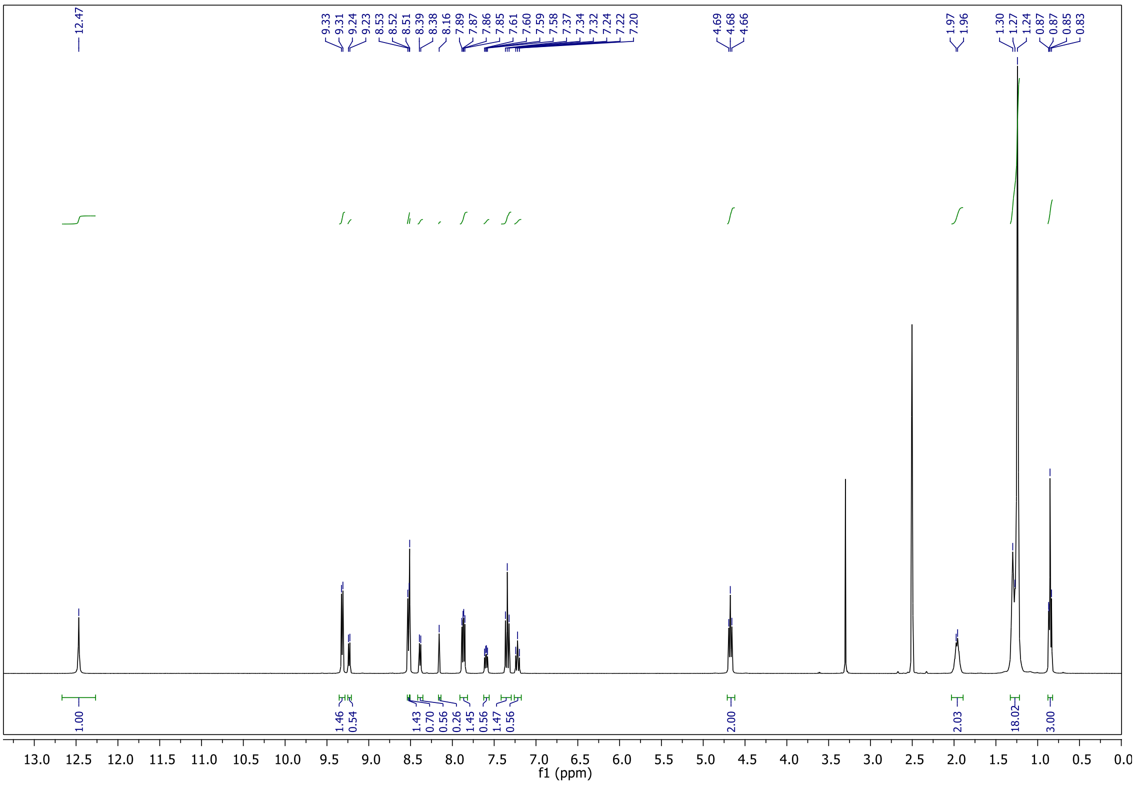

Figure S89. ^{1}HNMR of Compound 22

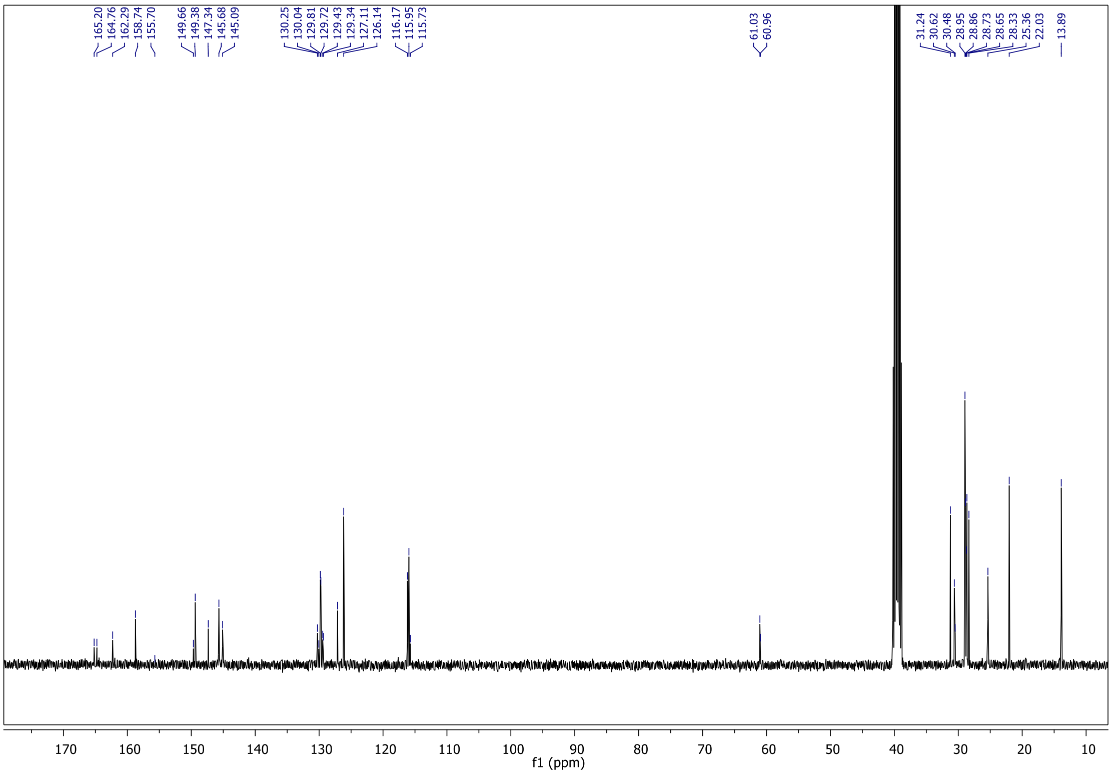

Figure S90. ^{13}CNMR of Compound 22

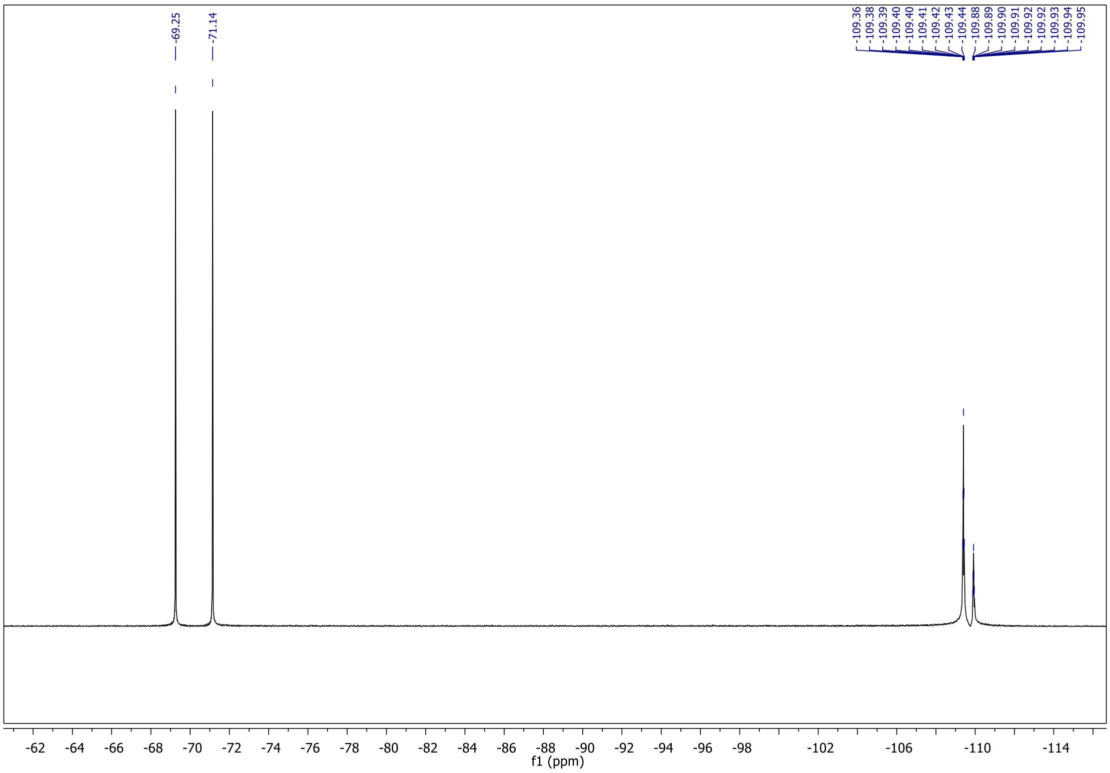

Figure S91. ^{19}FNMR of Compound 22

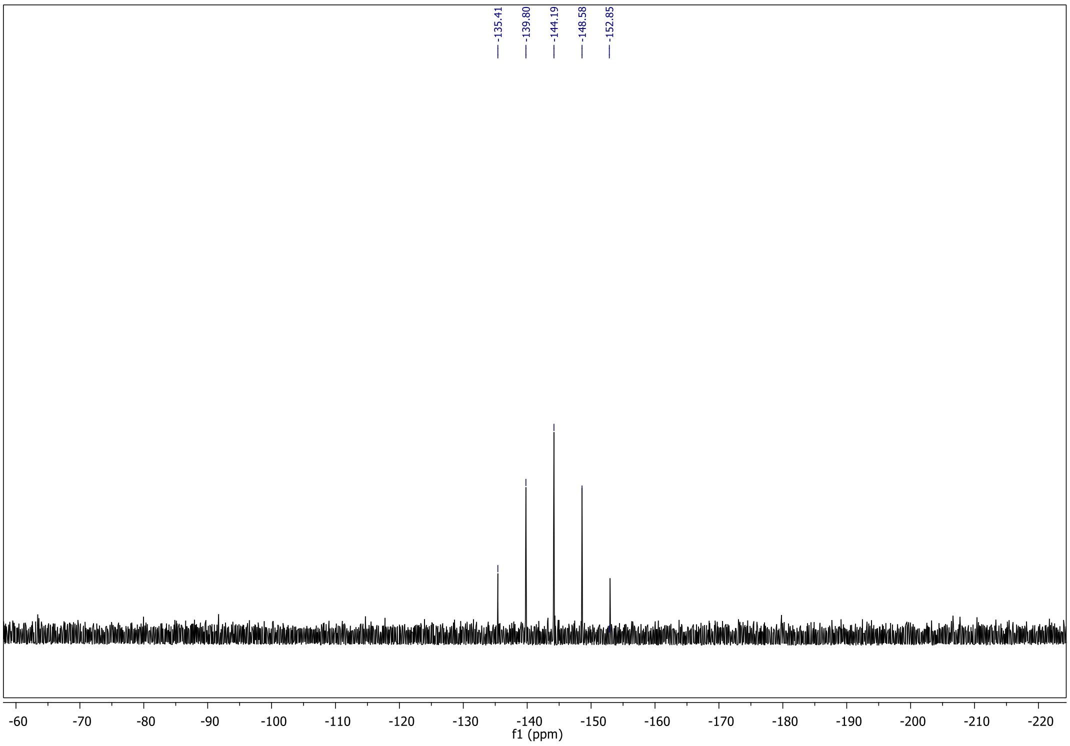

Figure S92. ^{31}PNMR of Compound 22

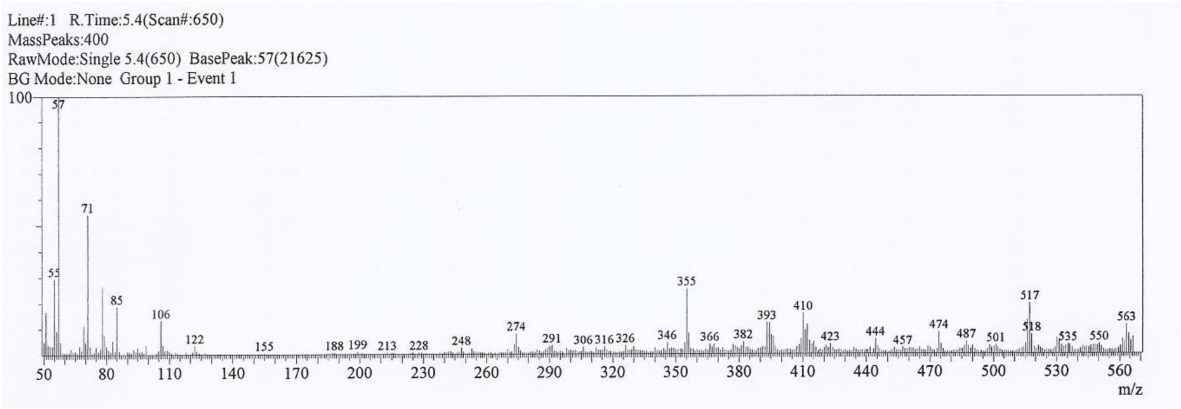

Figure S93. MS (ESI) of Compound 22

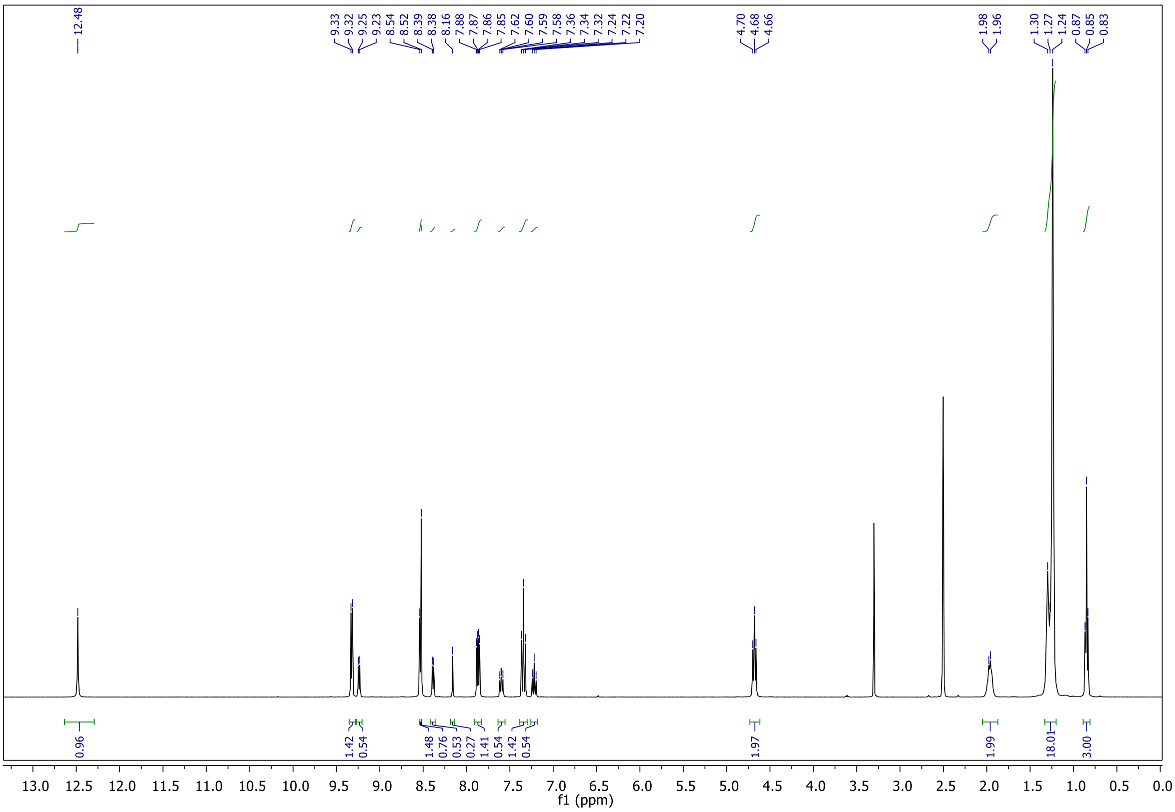

Figure S94. ^{1}HNMR of Compound 23

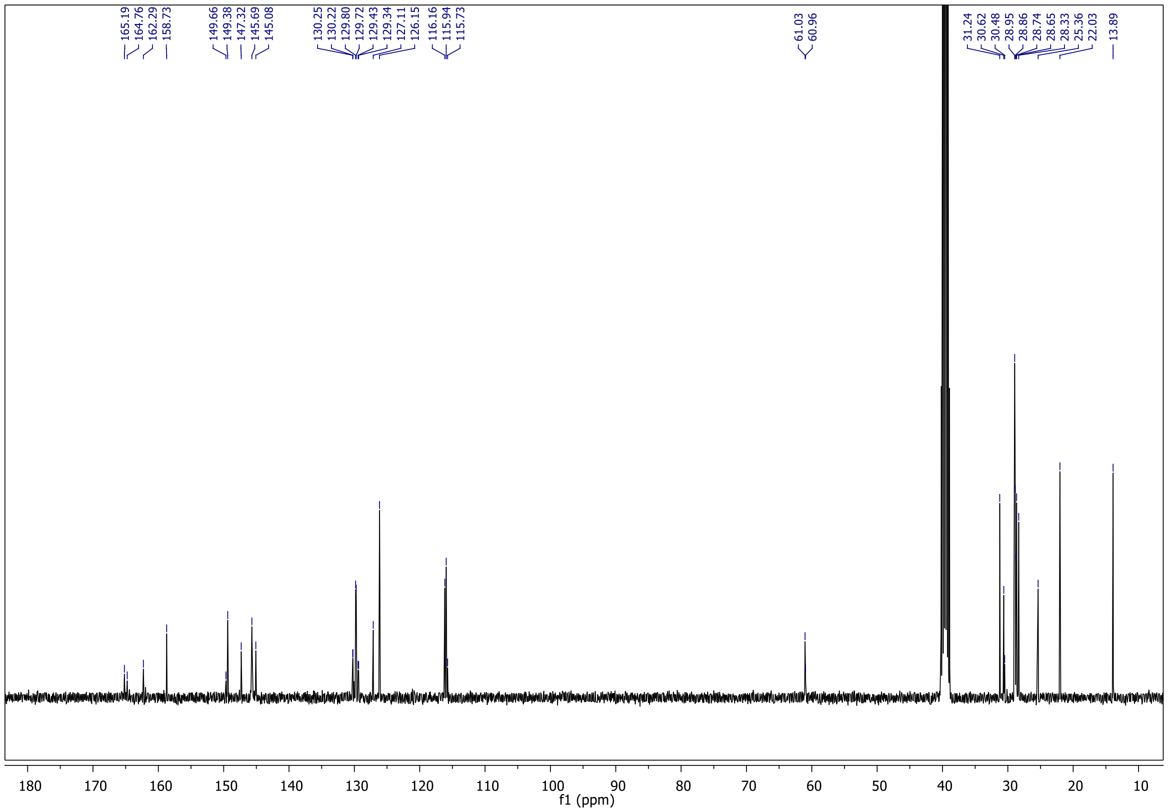

Figure S95. ^{13}CNMR of Compound 23

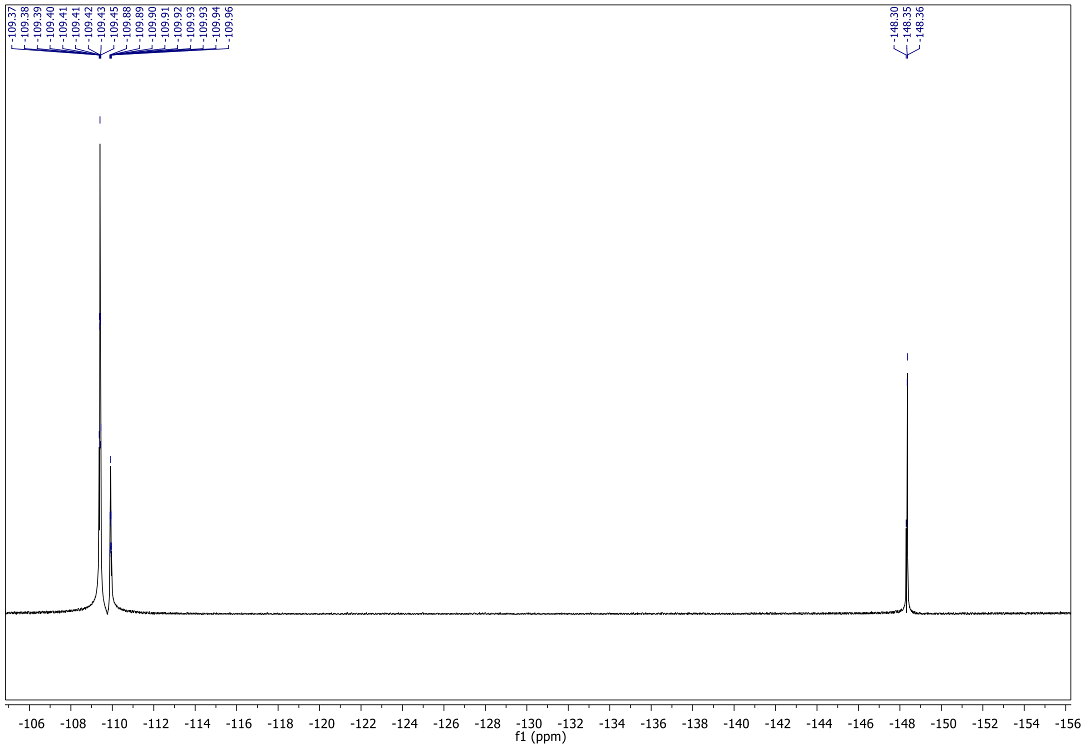

Figure S96. ^{19}FNMR of Compound 23

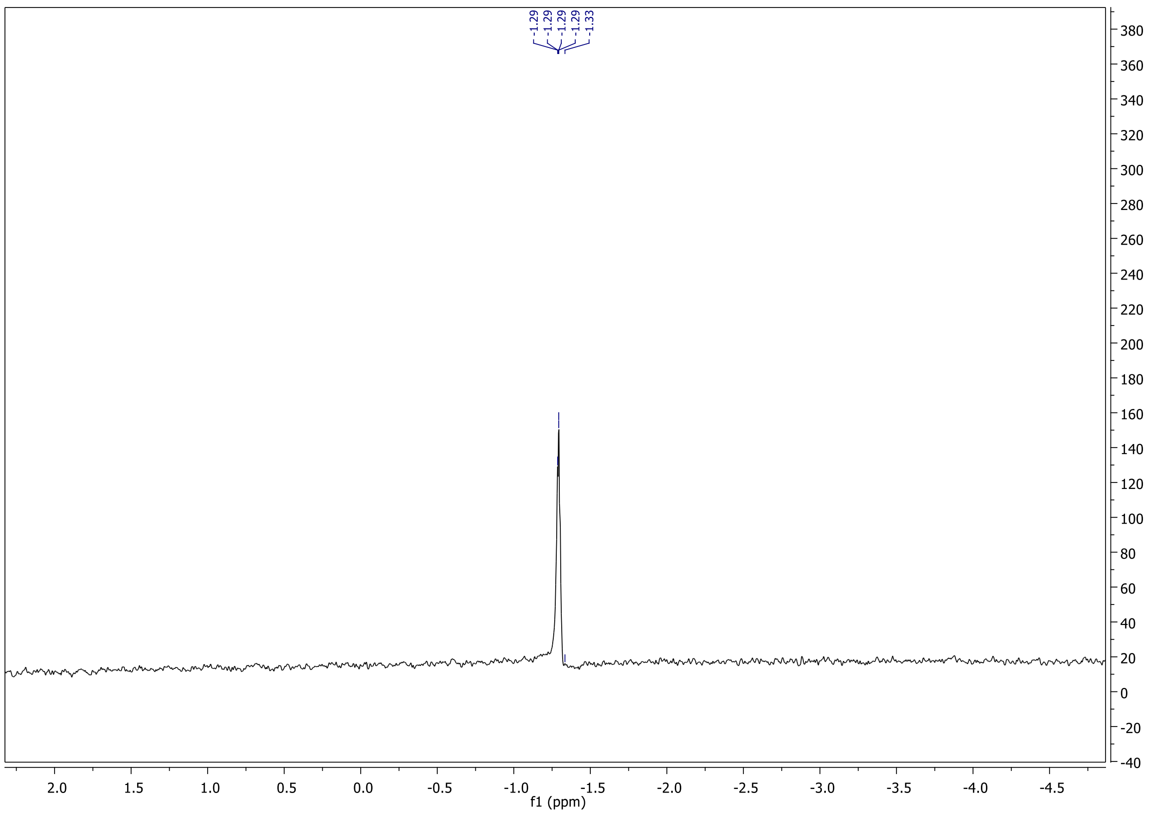

Figure S97. ^{11}BNMR of Compound 23

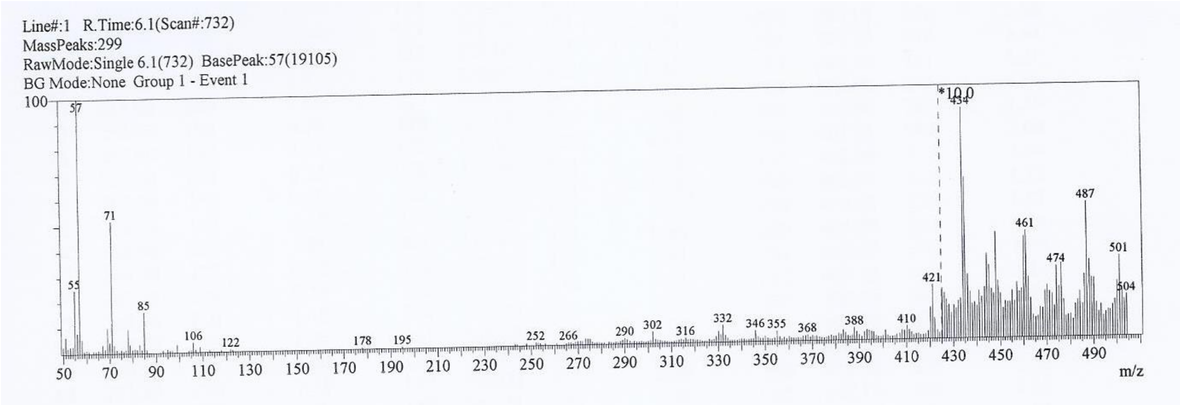

Figure S98. MS (ESI) of Compound 23

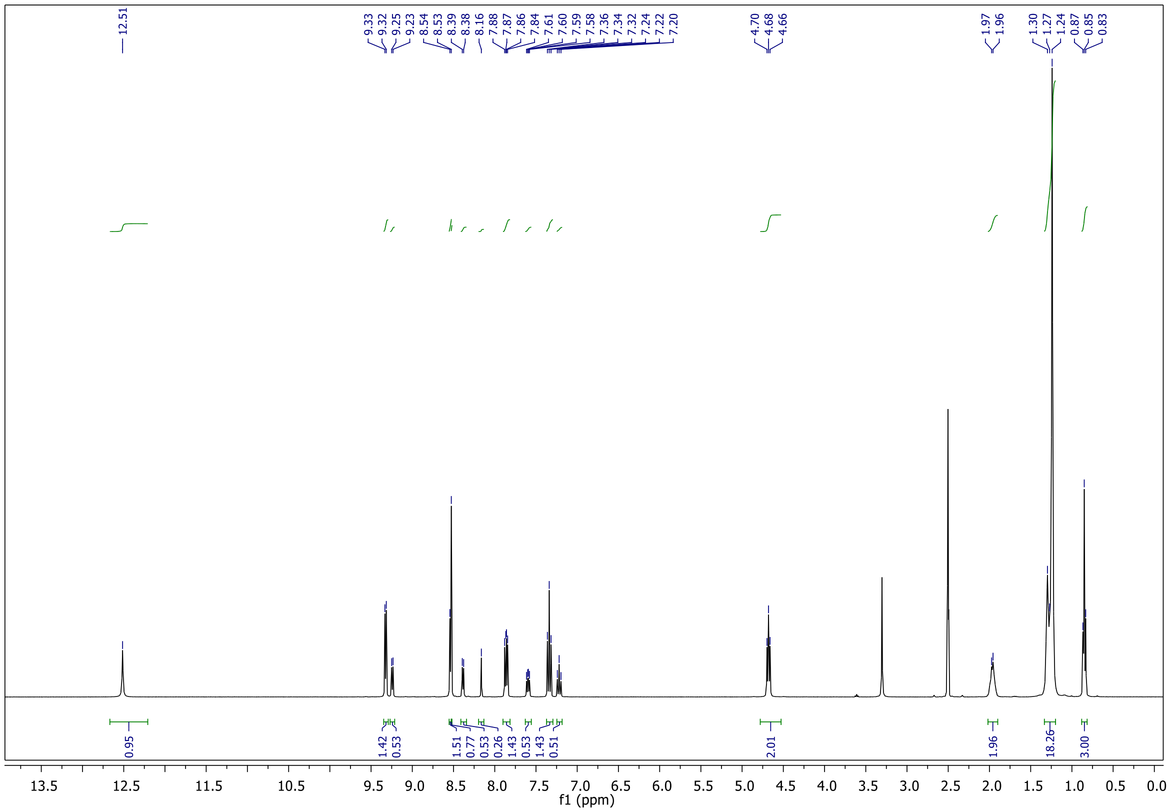

Figure S99. ^{1}HNMR of Compound 24

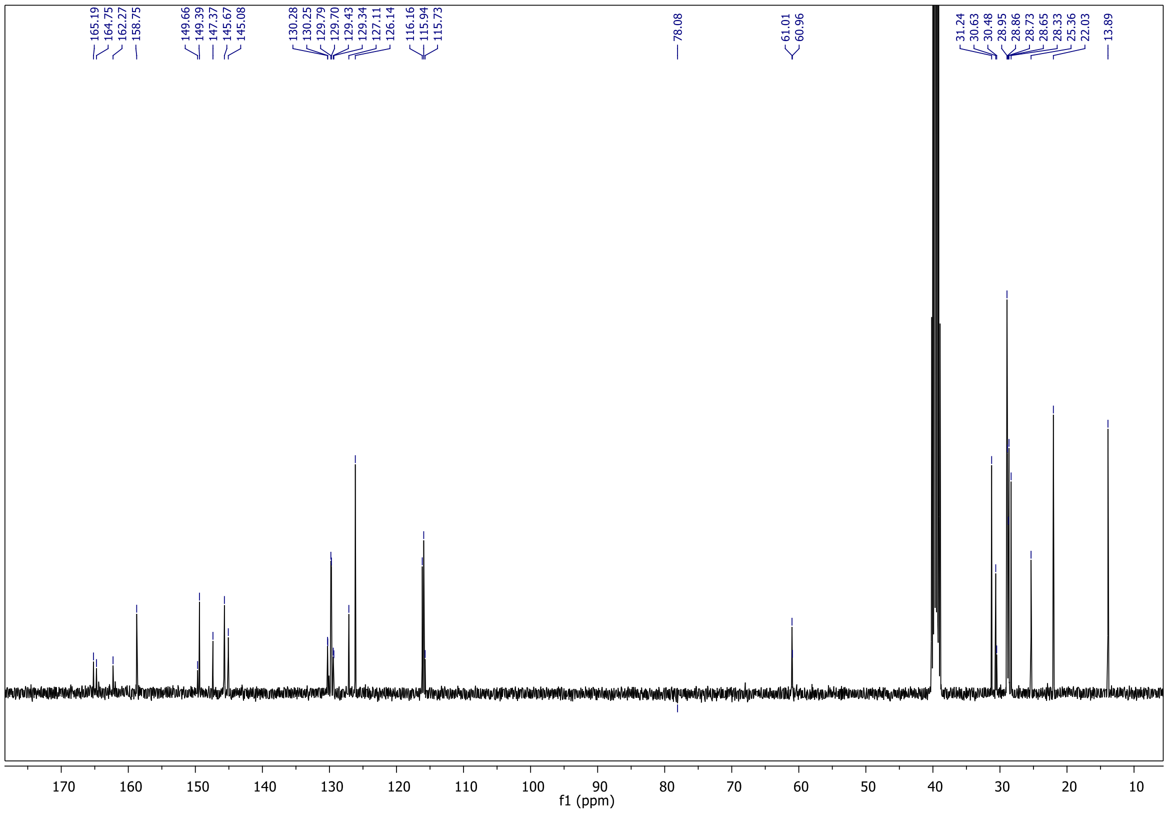

Figure S100. ^{13}CNMR of Compound 24

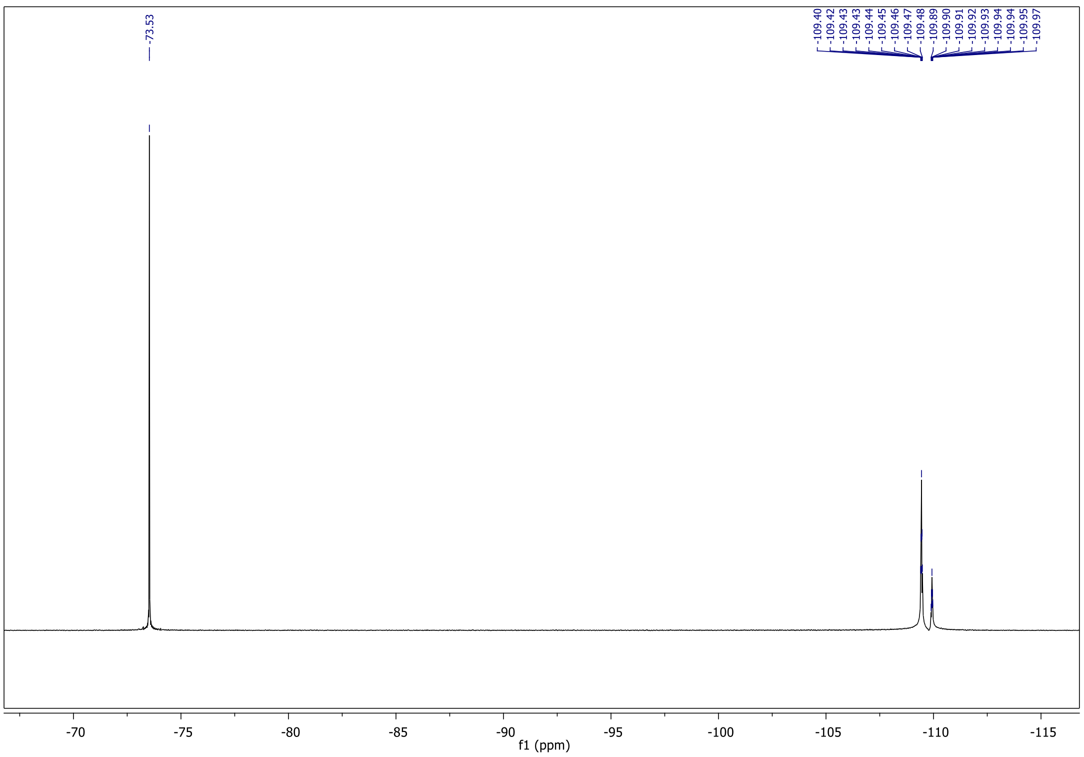

Figure S101. ^{19}FNMR of Compound 24

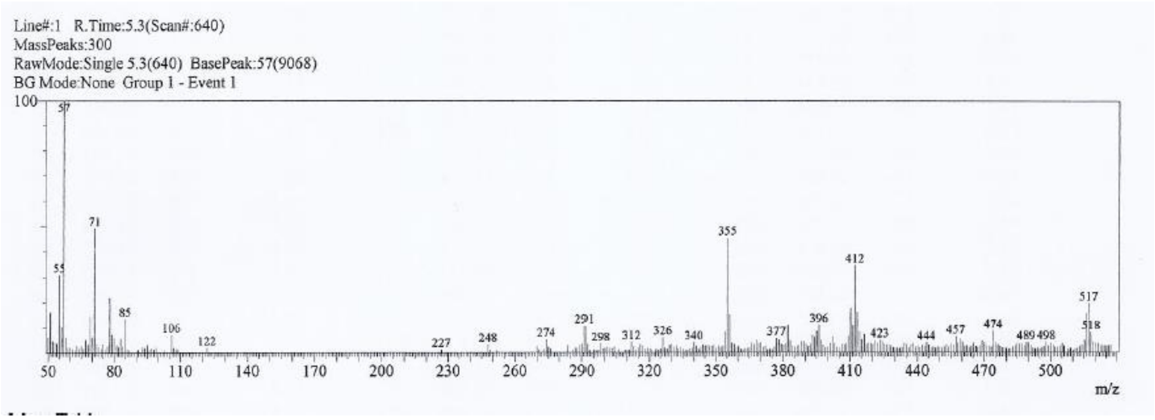

Figure S102. MS (ESI) of Compound 24

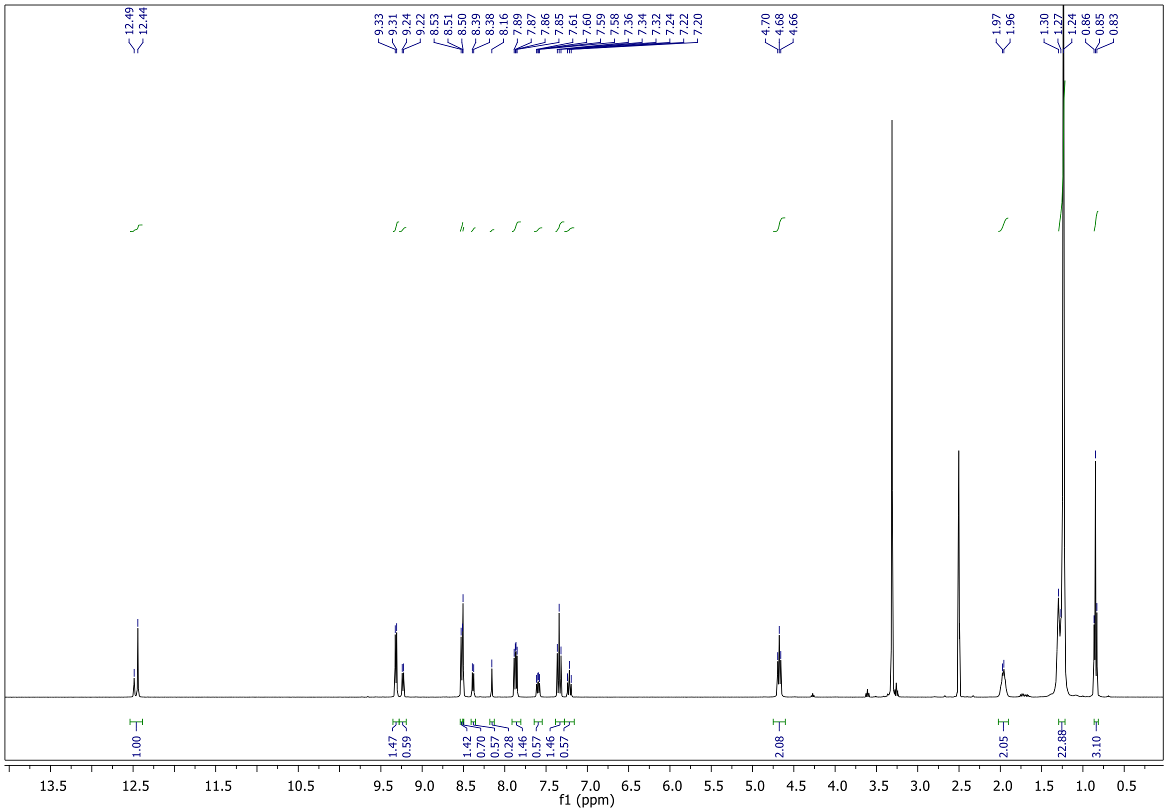

Figure S103. ^{1}HNMR of Compound 25

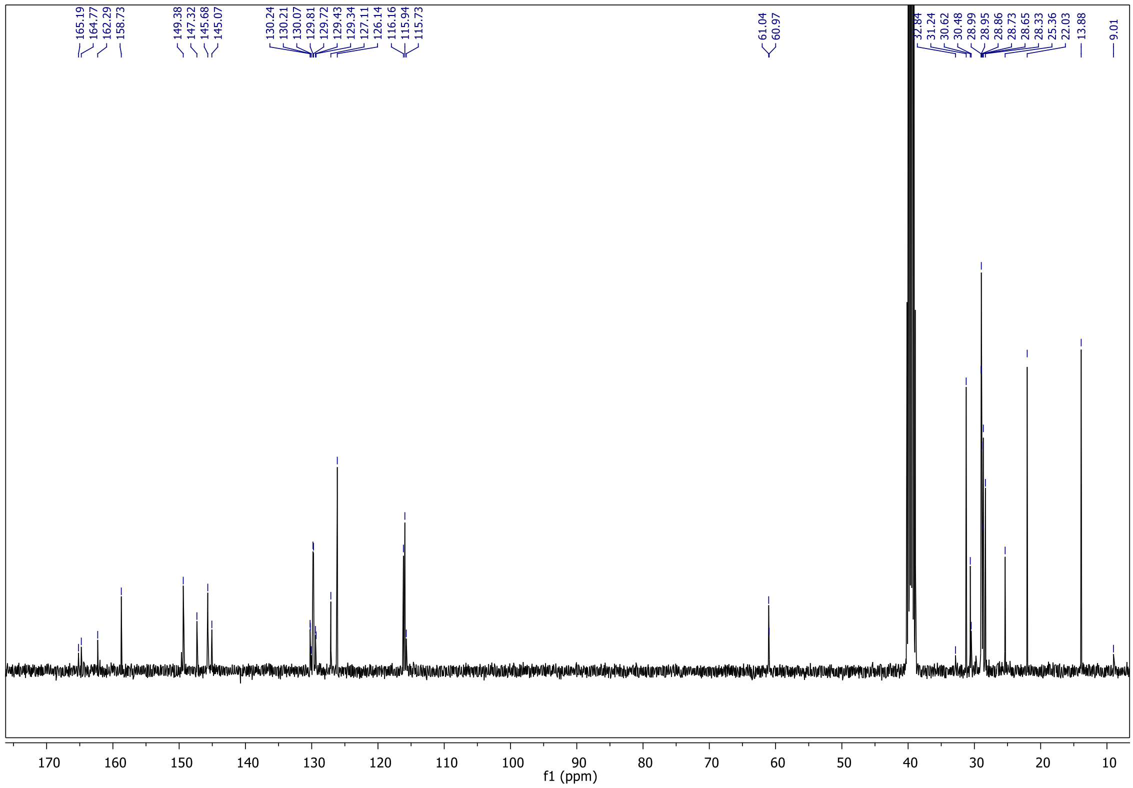

Figure S104. ^{13}CNMR of Compound 25

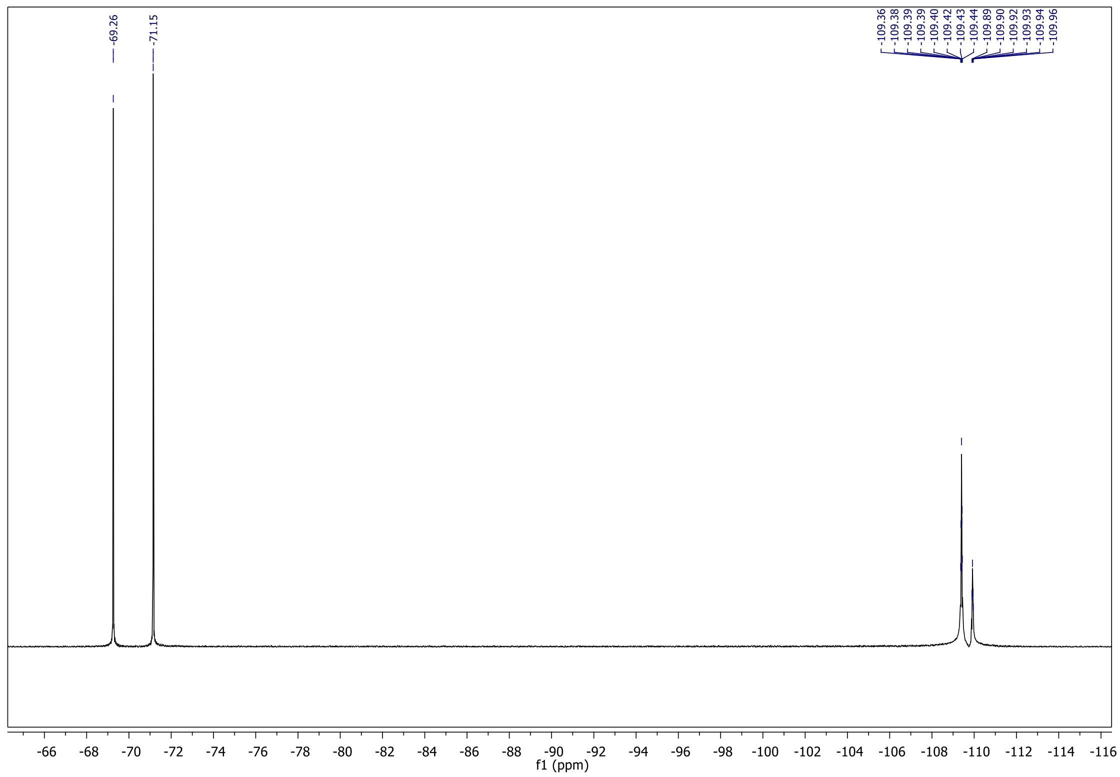

Figure S105. ^{19}FNMR of Compound 25

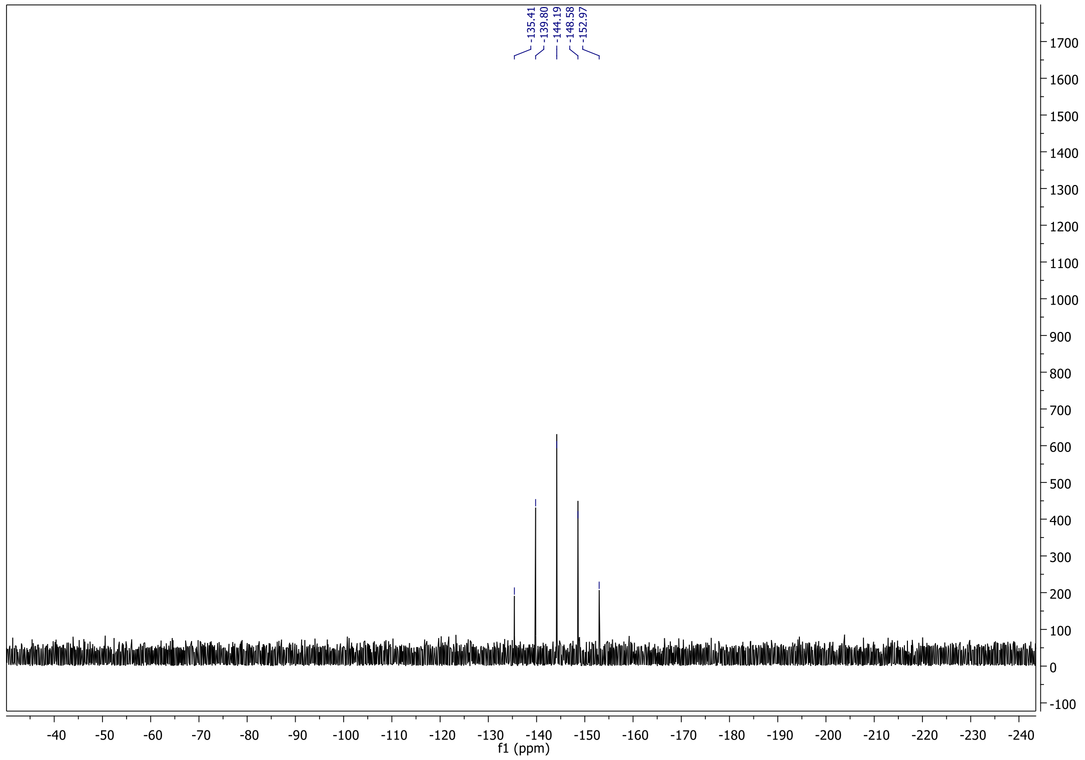

Figure S106. ^{31}PNMR of Compound 25

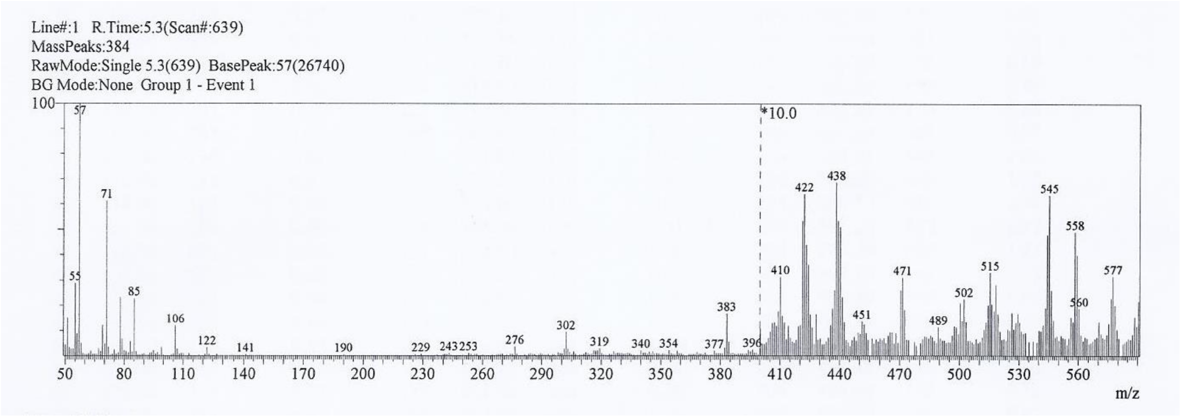

Figure S107. MS (ESI) of Compound 25

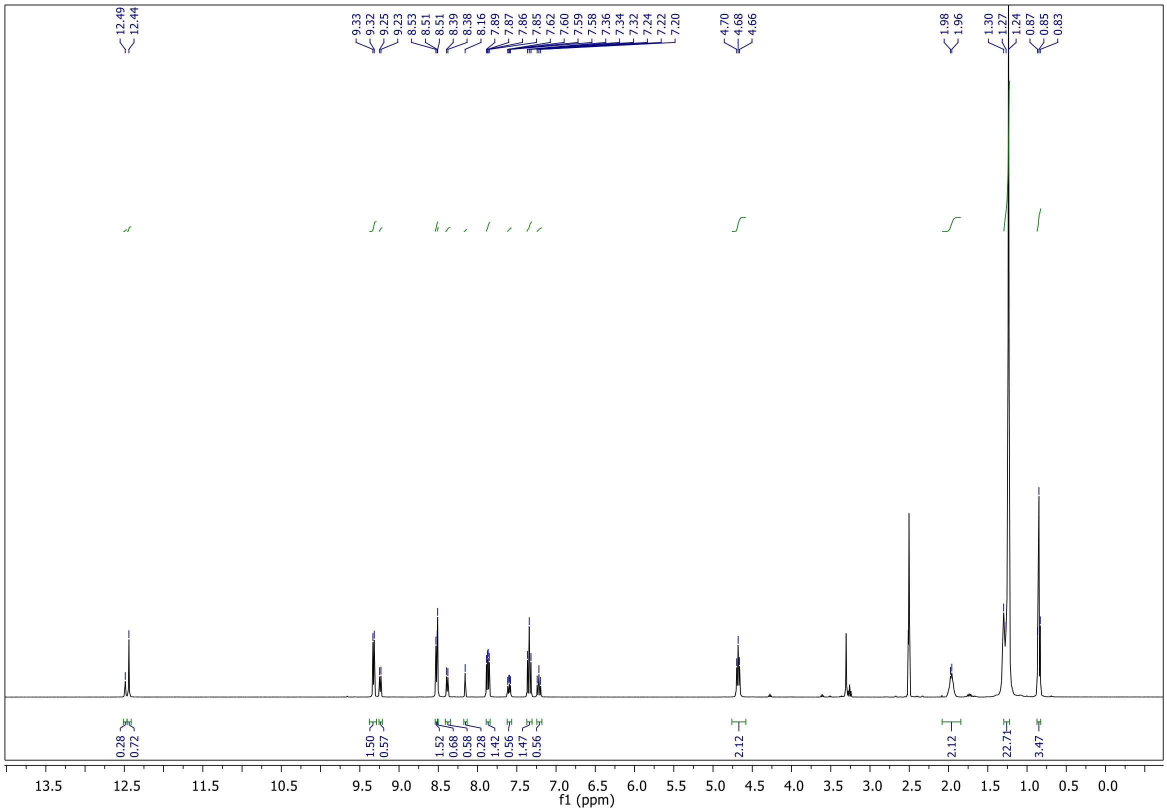

Figure S108. ^{1}HNMR of Compound 26

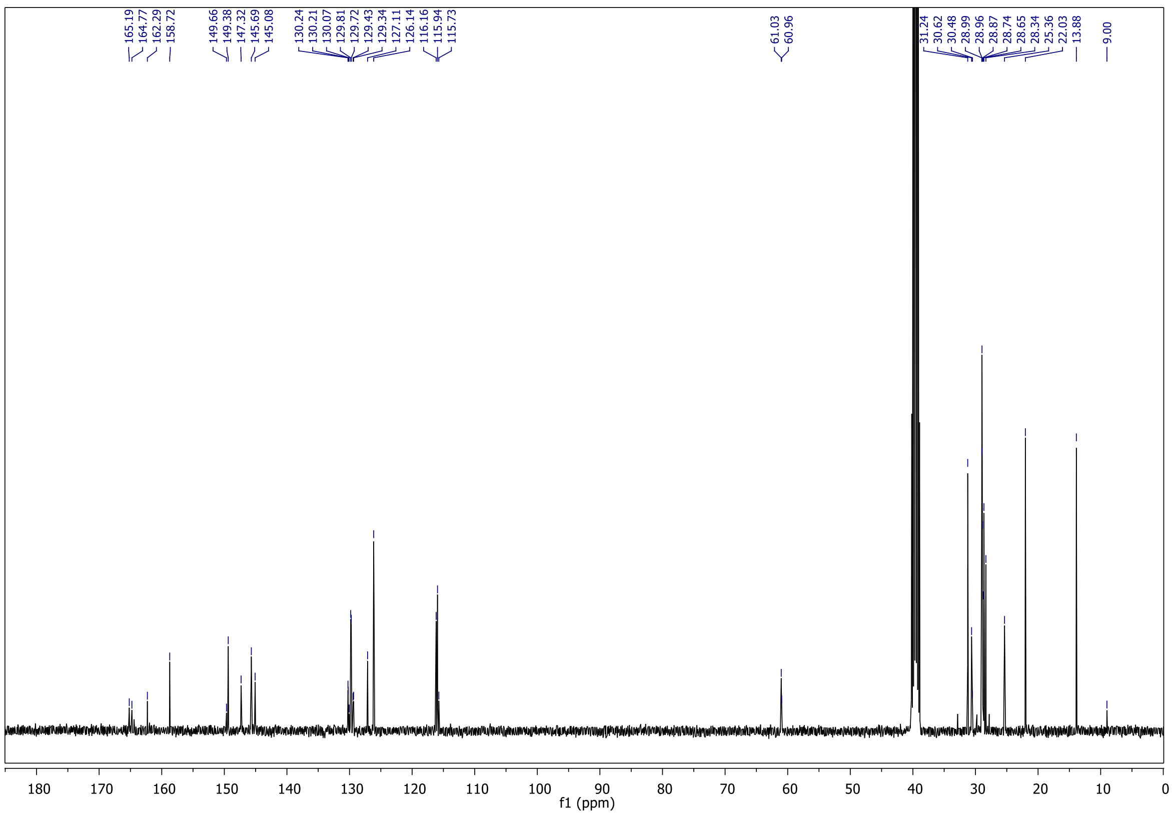

Figure S109. ^{13}CNMR of Compound 26

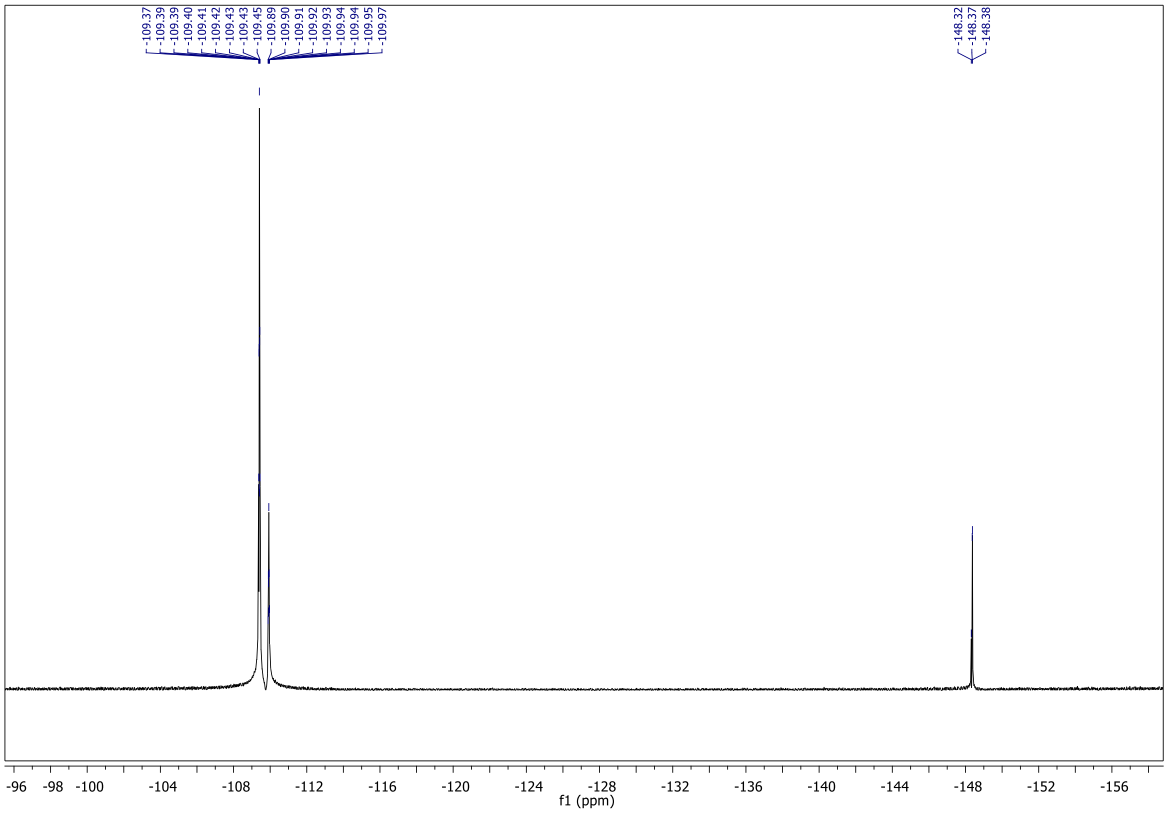

Figure S110. ^{19}FNMR of Compound 26

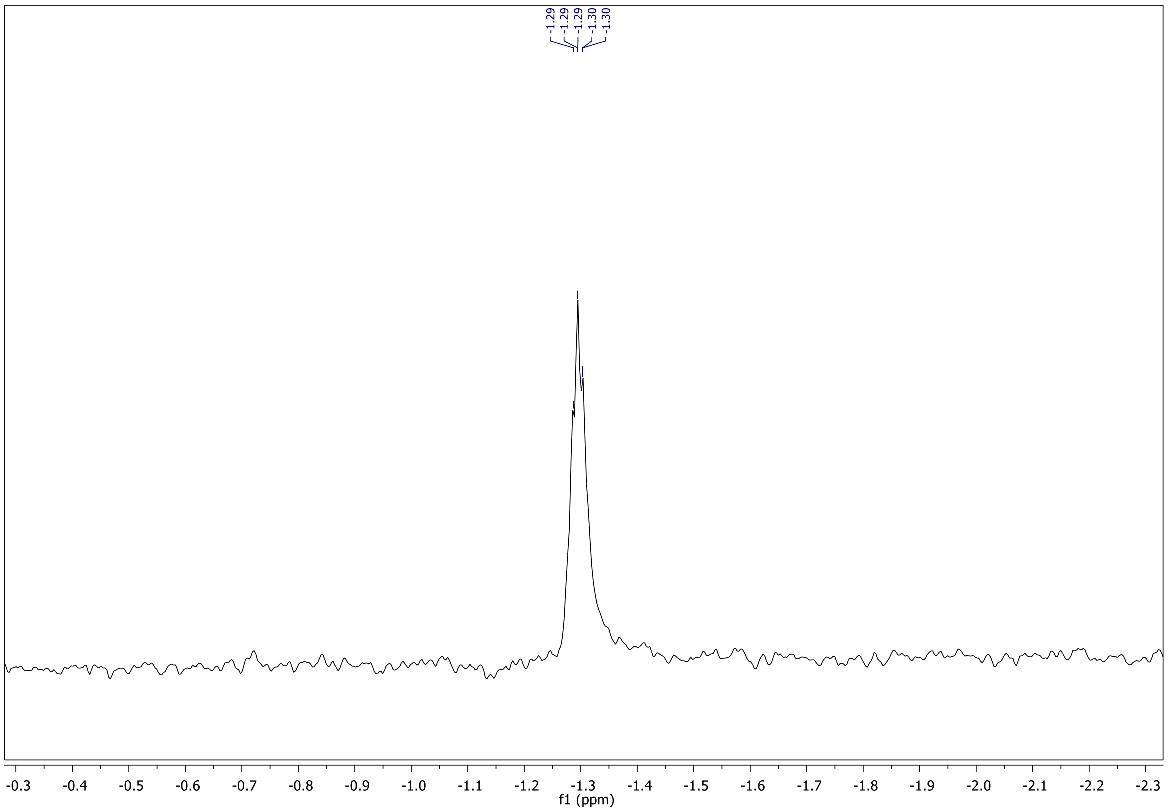

Figure S111. ^{11}BNMR of Compound 26

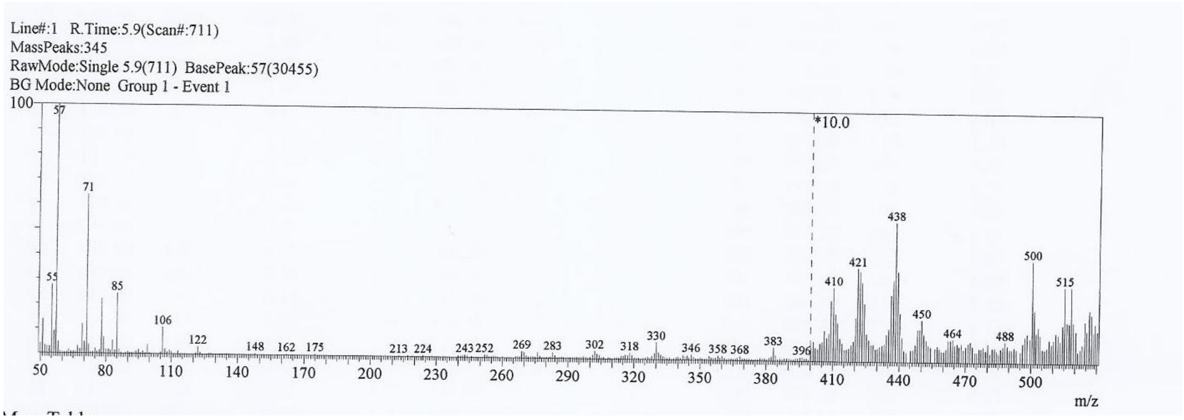

Figure S112. MS (ESI) of Compound 26

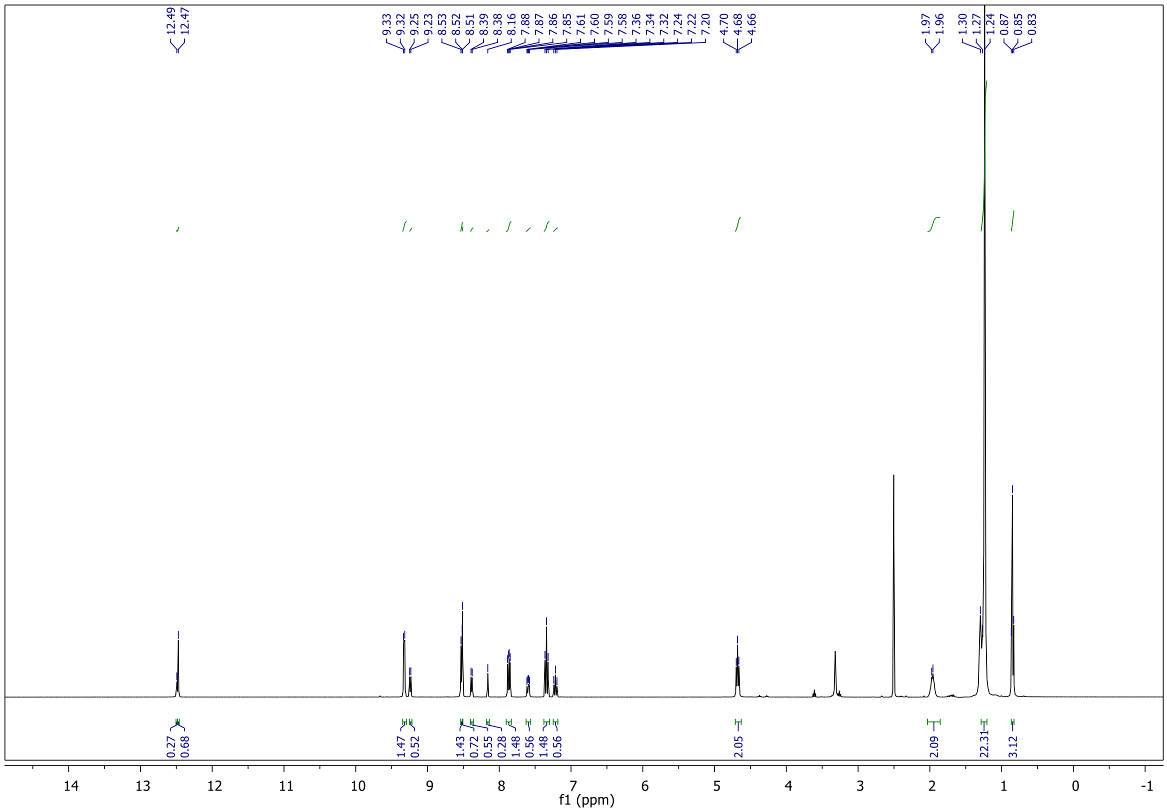

Figure S113. ^{1}HNMR of Compound 27

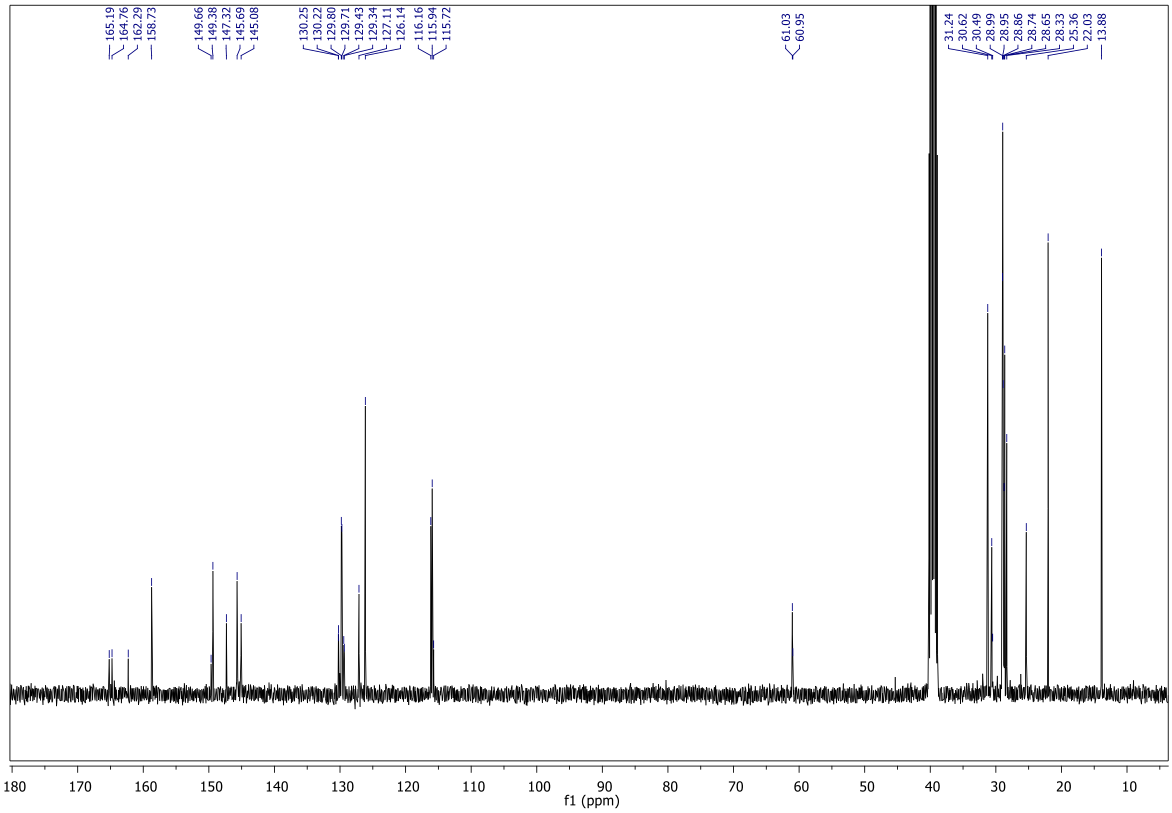

Figure S114. ^{13}CNMR of Compound 27

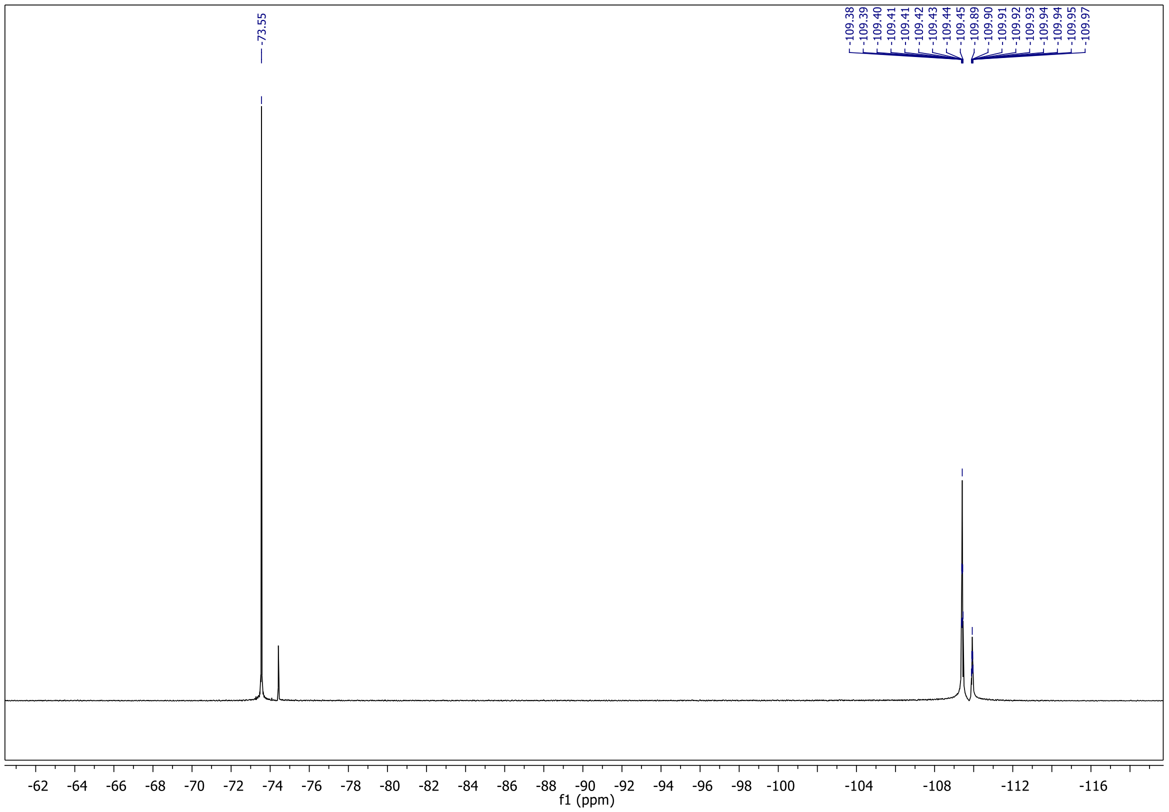

Figure S115. ^{19}FNMR of Compound 27

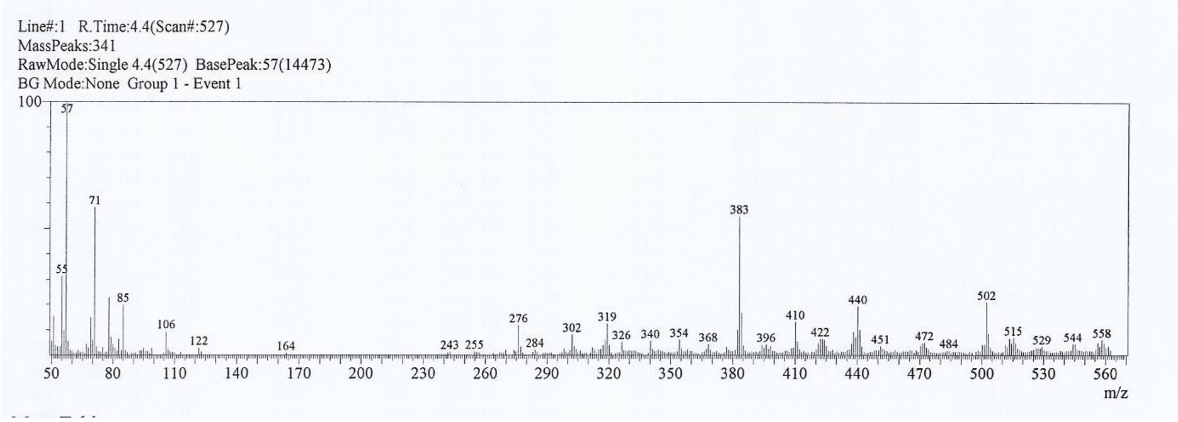

Figure S116. MS (ESI) of Compound 27

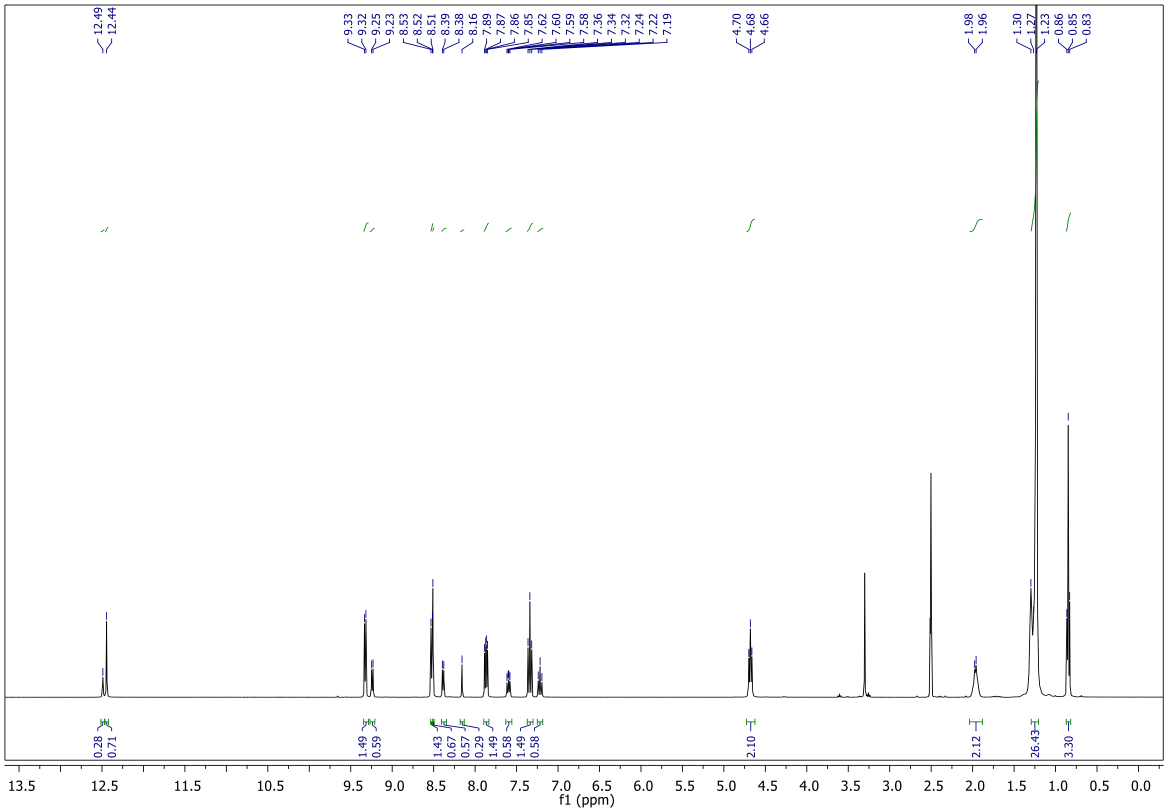

Figure S117. ^{1}HNMR of Compound 28

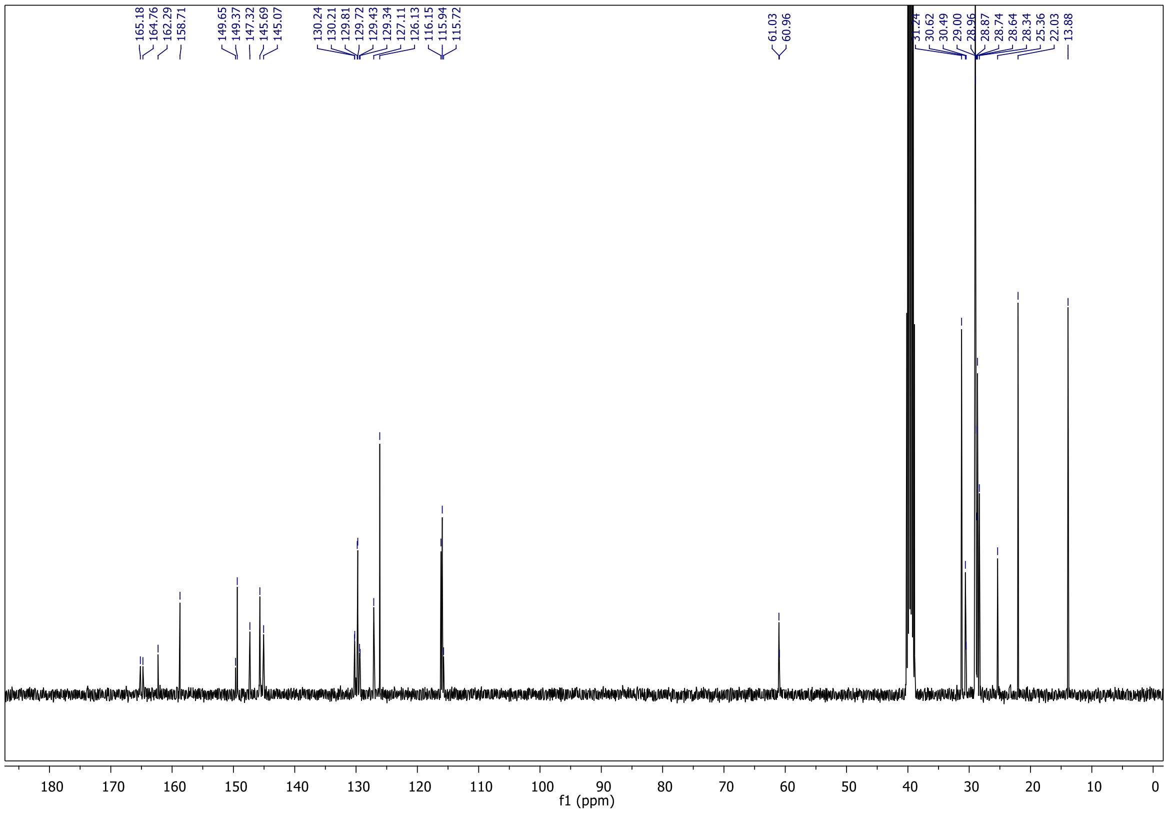

Figure S118. ^{13}CNMR of Compound 28

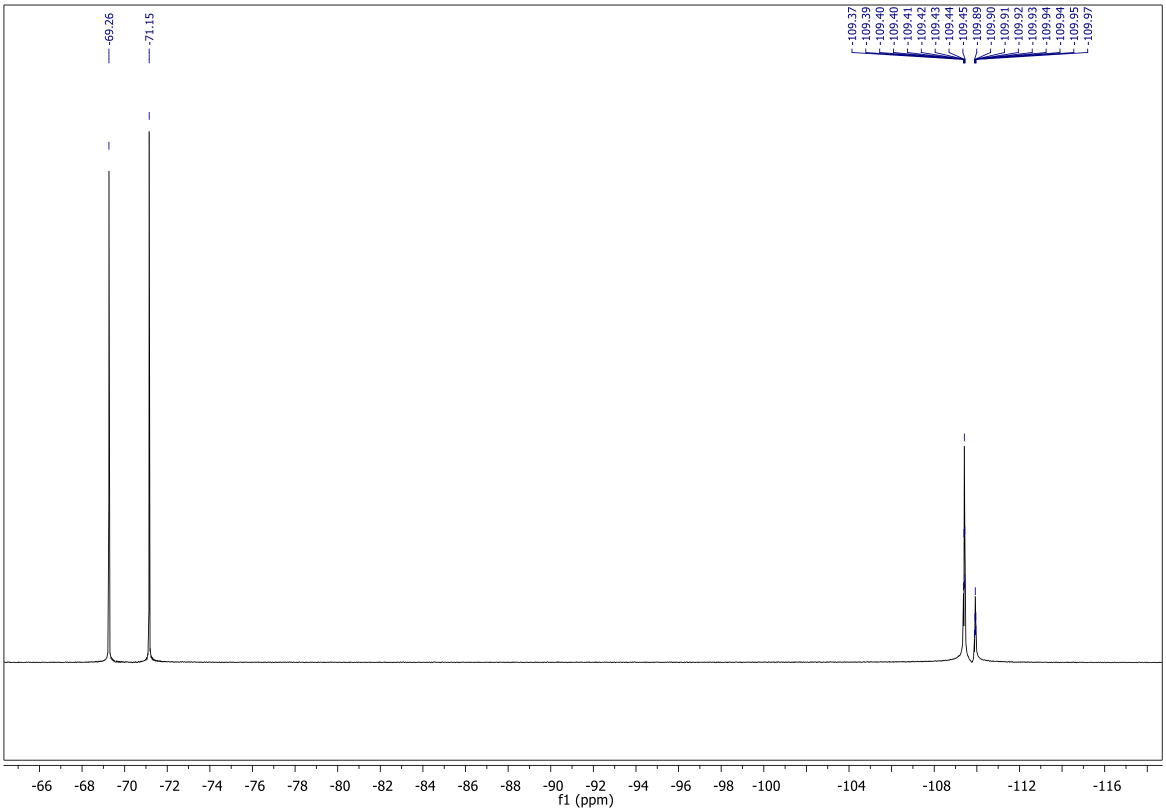

Figure S119. ^{19}FNMR of Compound 28

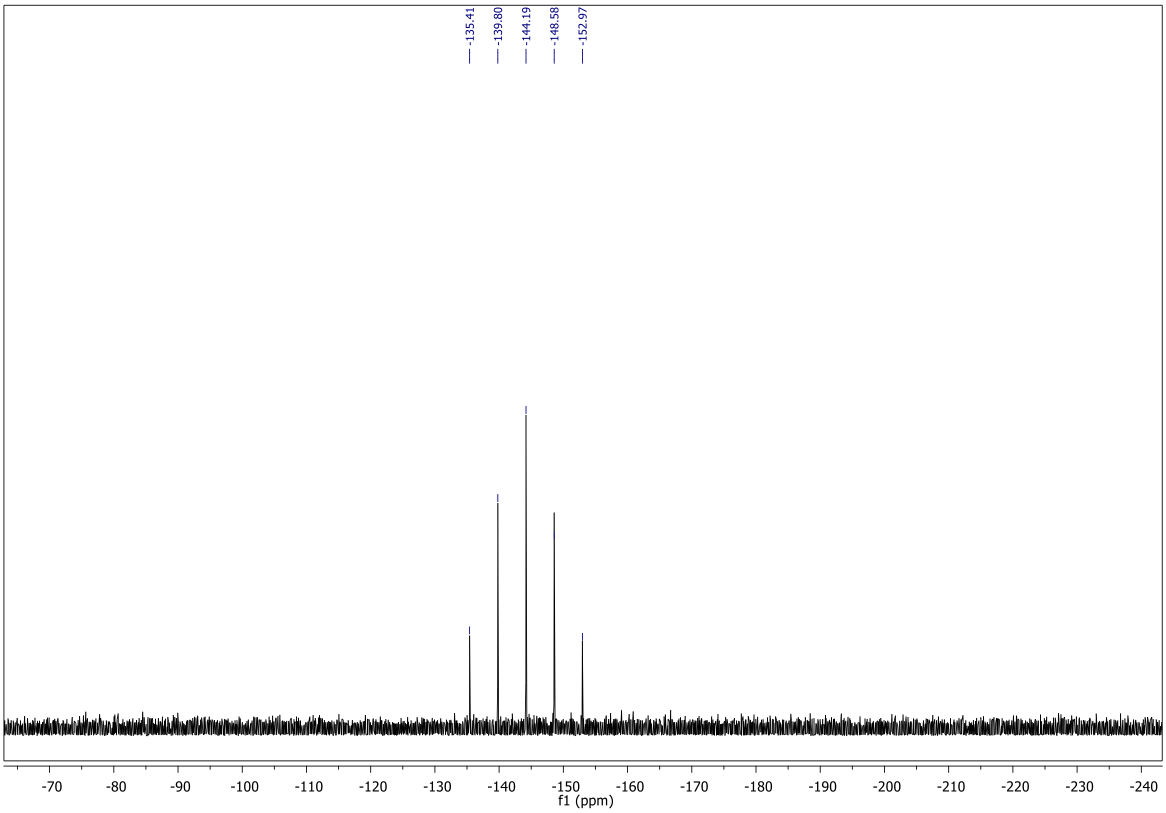

Figure S120. ^{31}PNMR of Compound 28

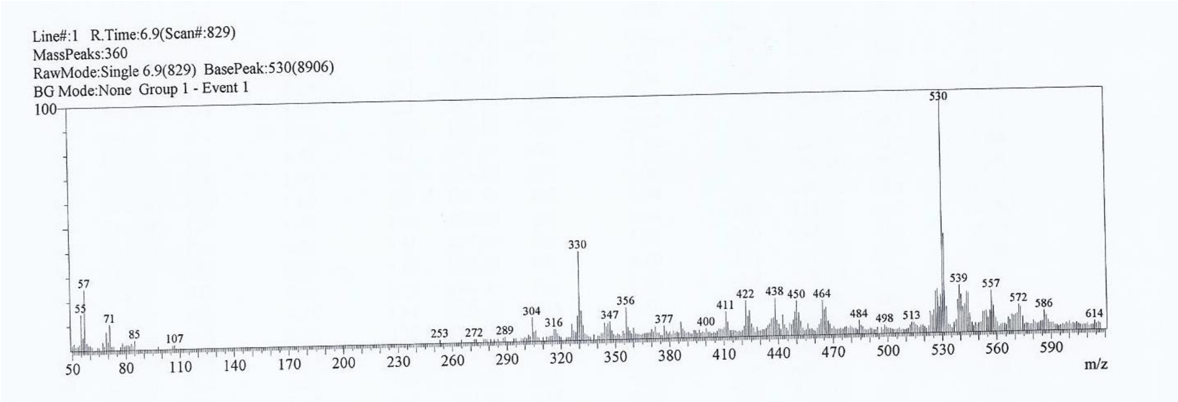

Figure S121. MS (ESI) of Compound 28

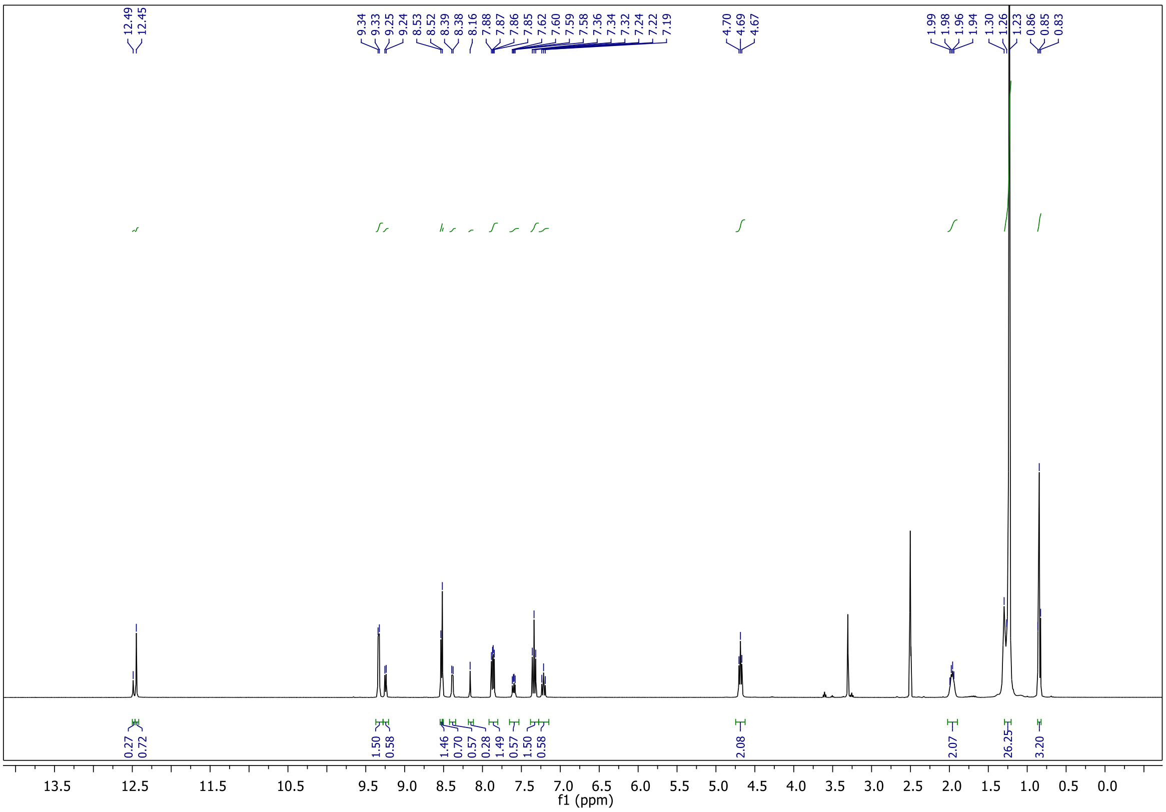

Figure S122. ^{1}HNMR of Compound 29

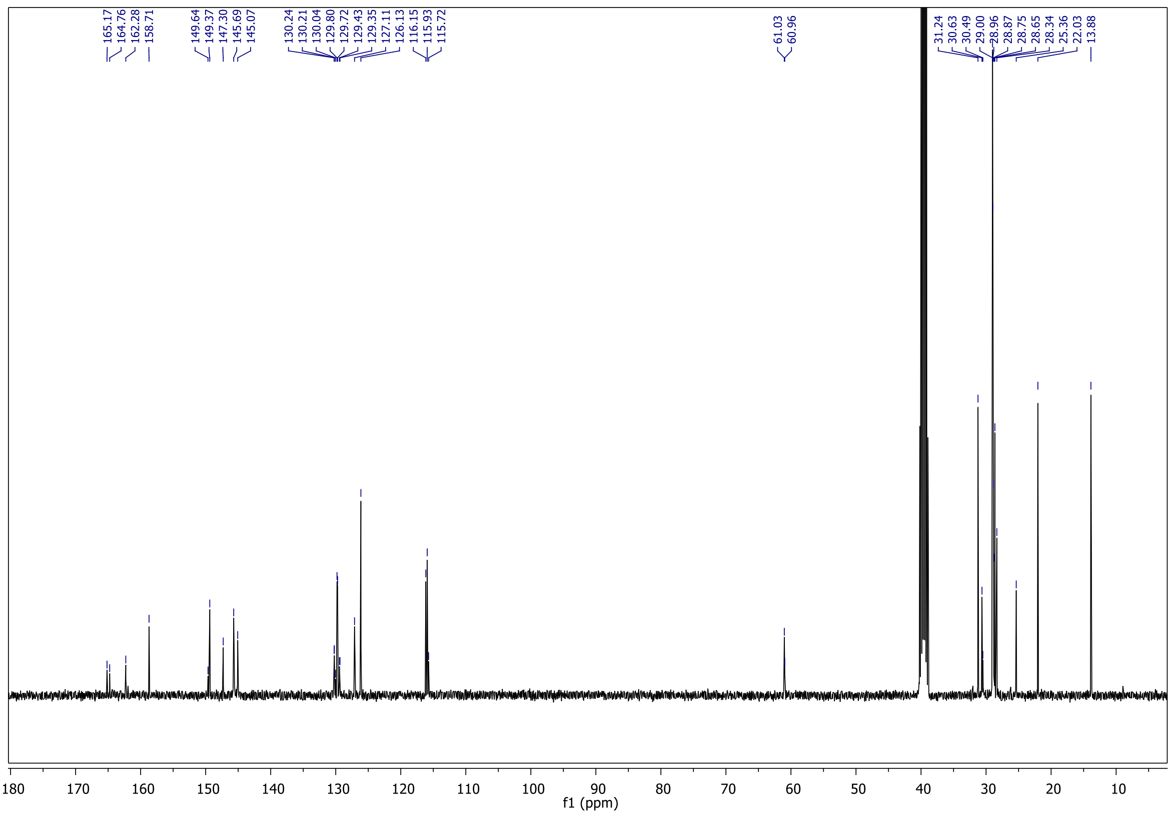

Figure S123. ^{13}CNMR of Compound 29

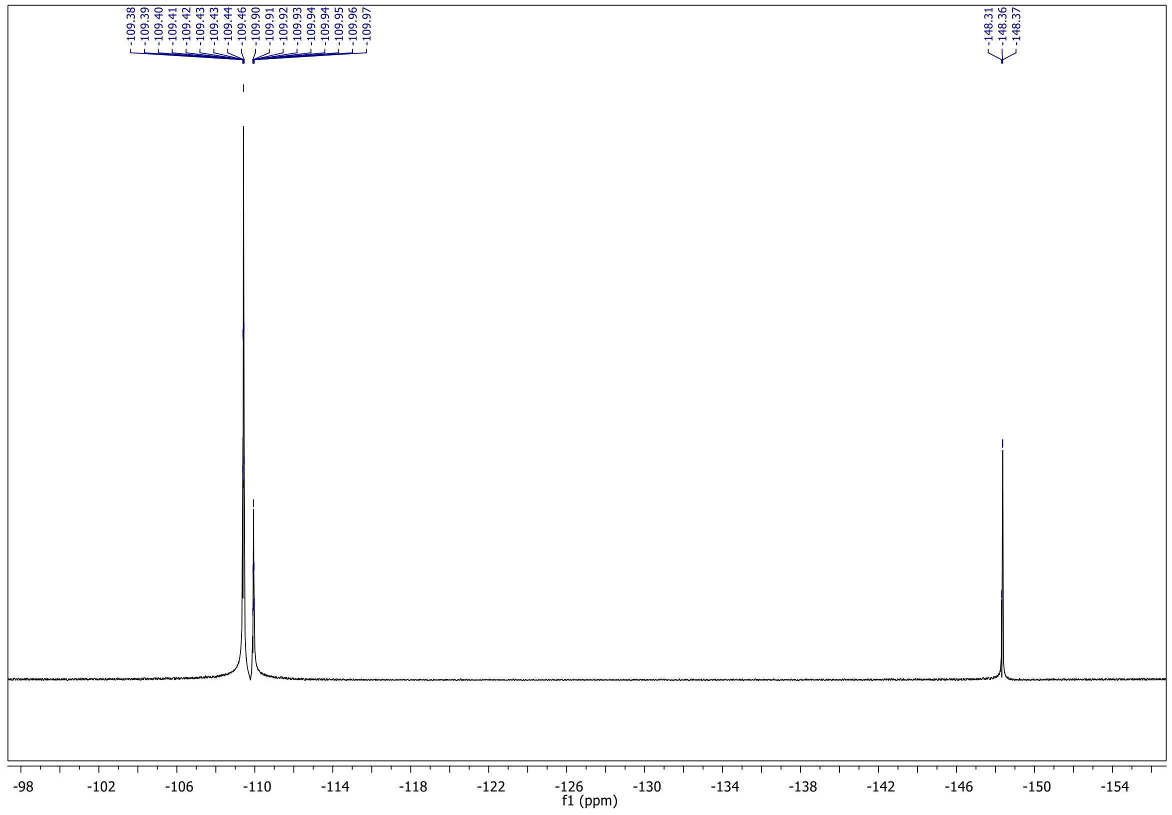

Figure S124. ^{19}FNMR of Compound 29

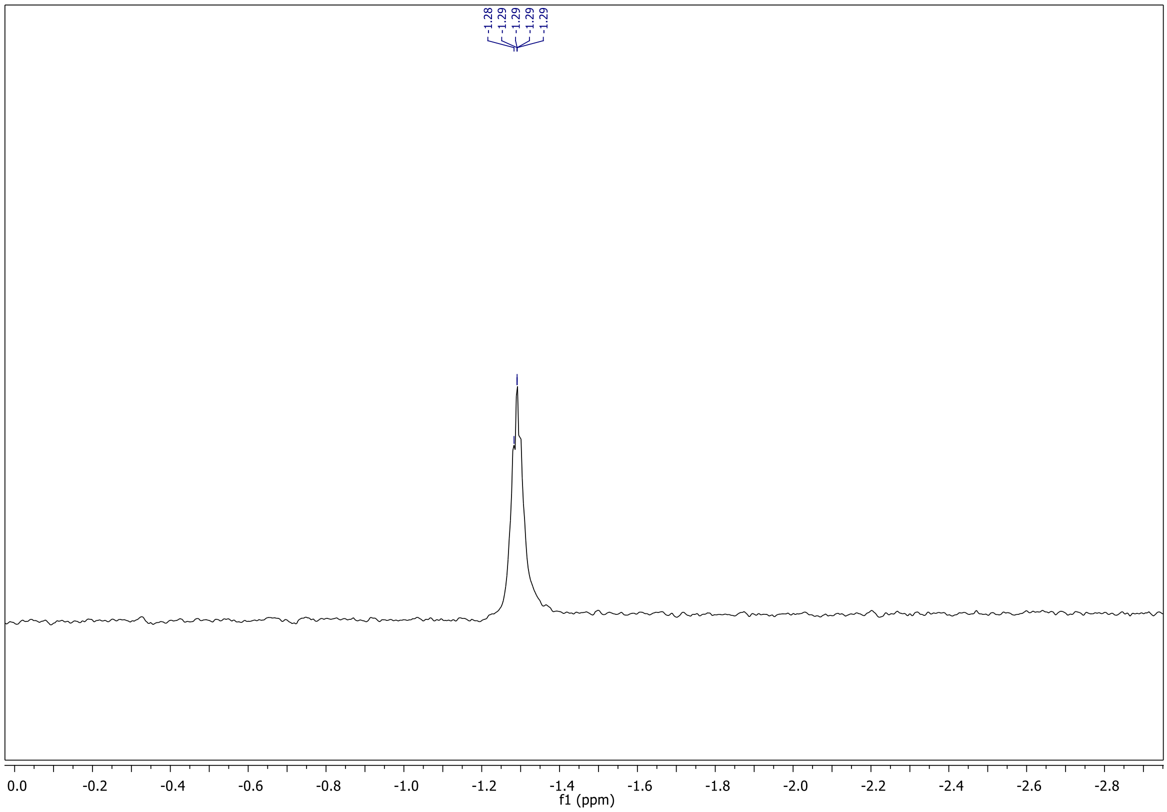

Figure S125. ^{11}BNMR of Compound 29

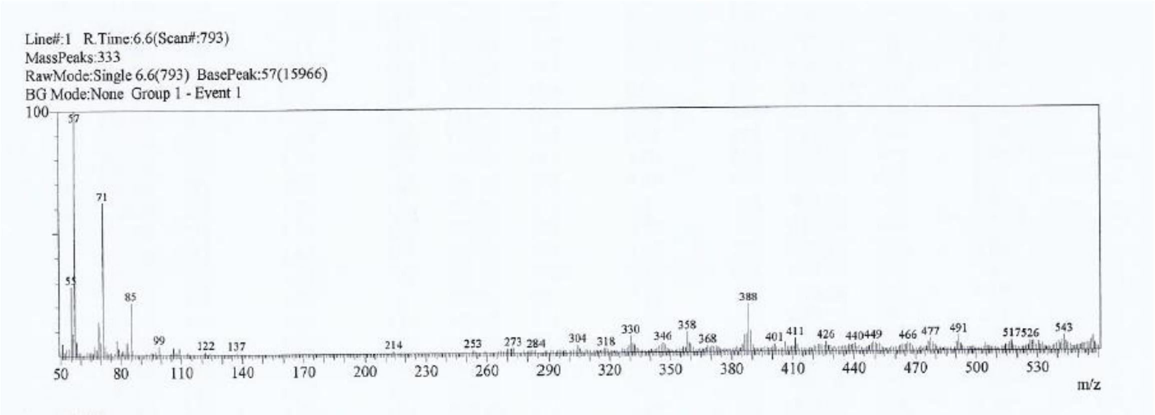

Figure S126. MS (ESI) of Compound 29

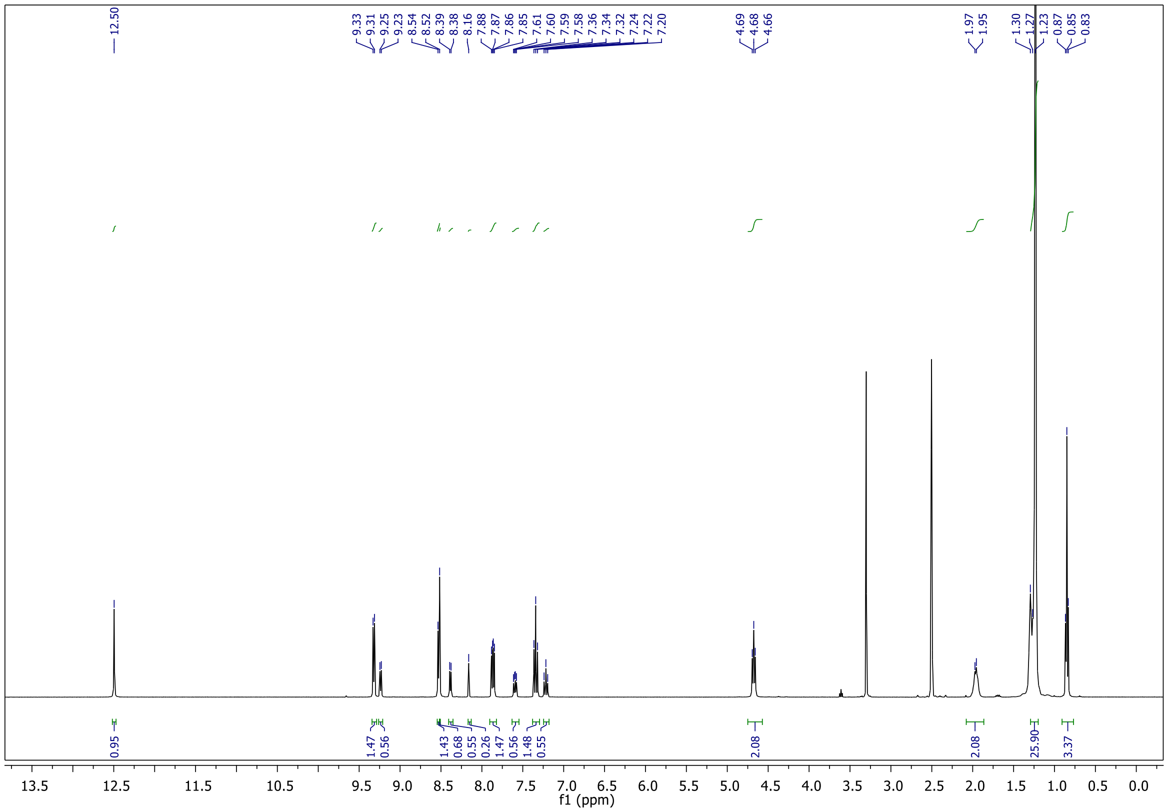

Figure S127. ^{1}HNMR of Compound 30

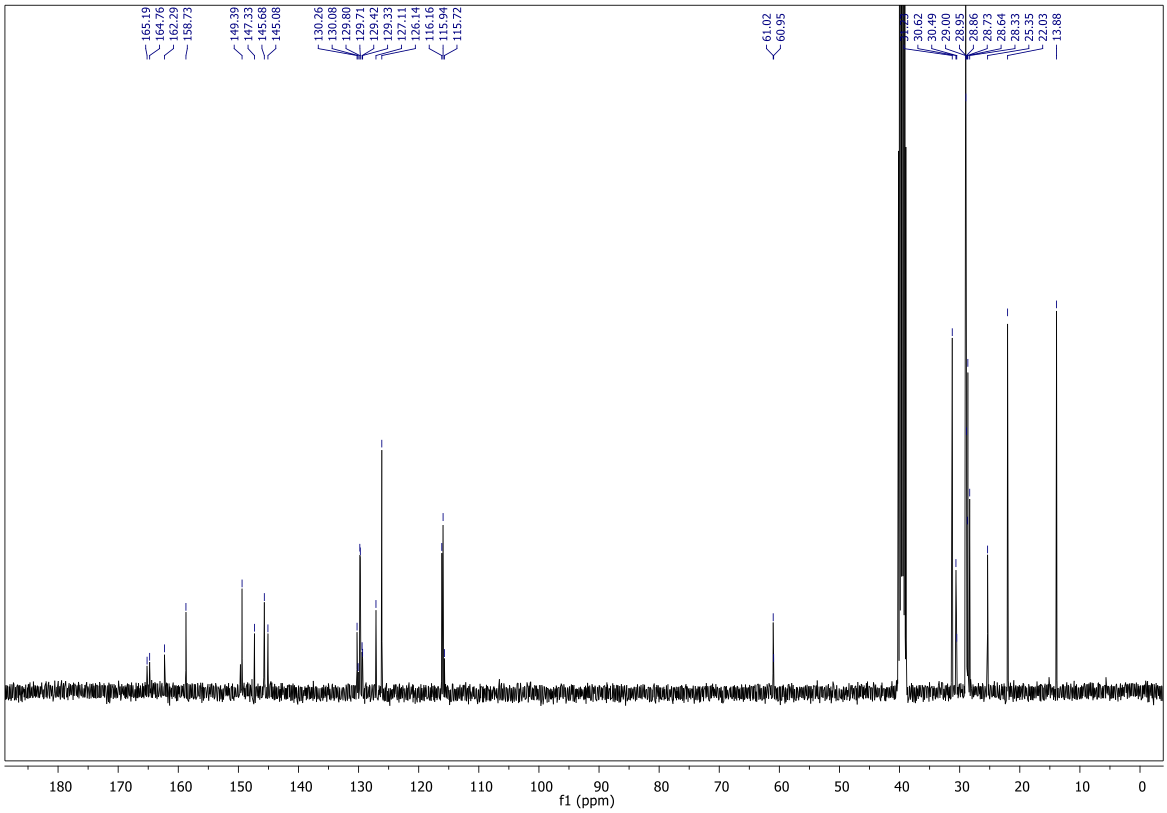

Figure S128. ^{13}CNMR of Compound 30

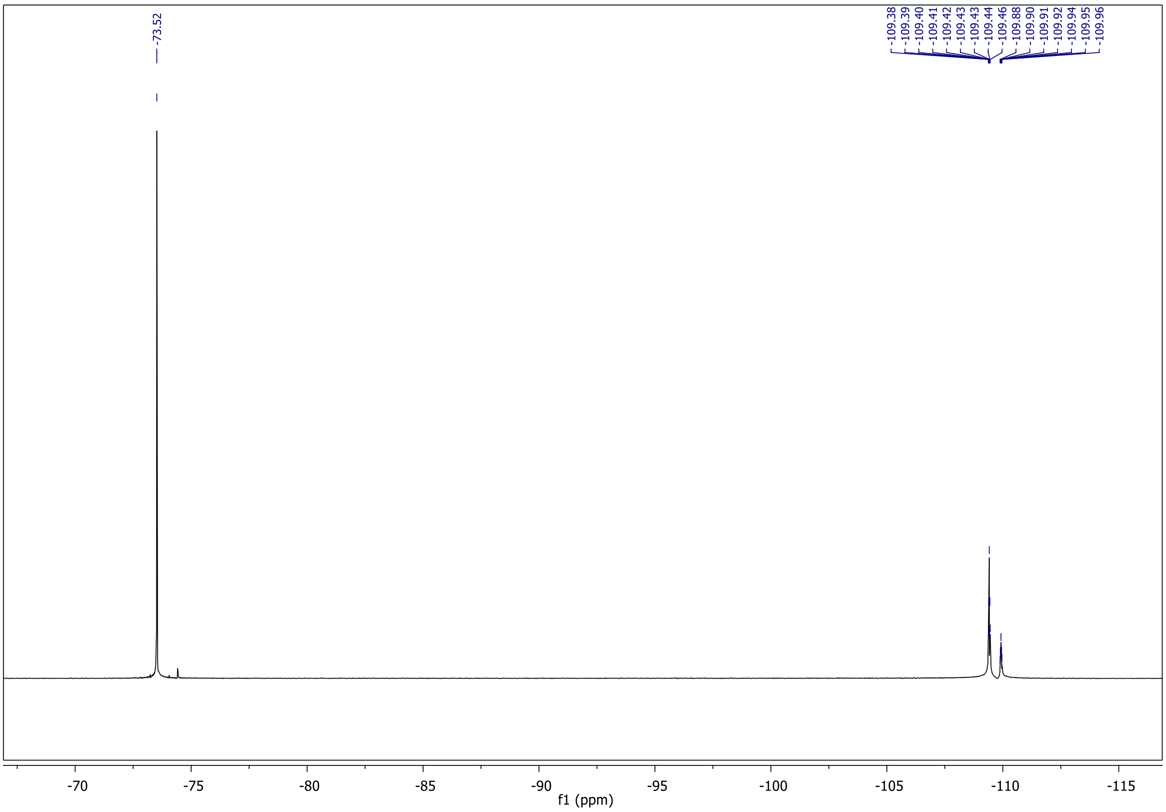

Figure S129. ^{19}FNMR of Compound 30

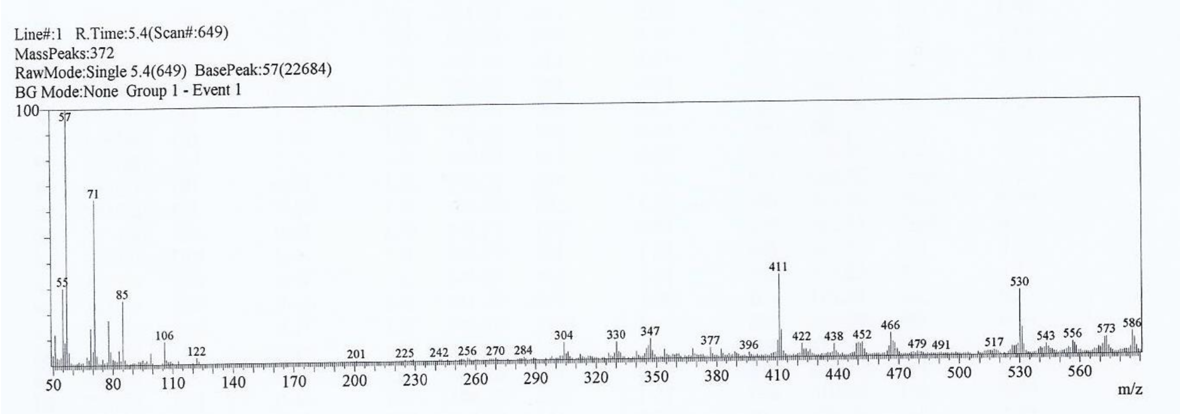

Figure S130. MS (ESI) of Compound 30

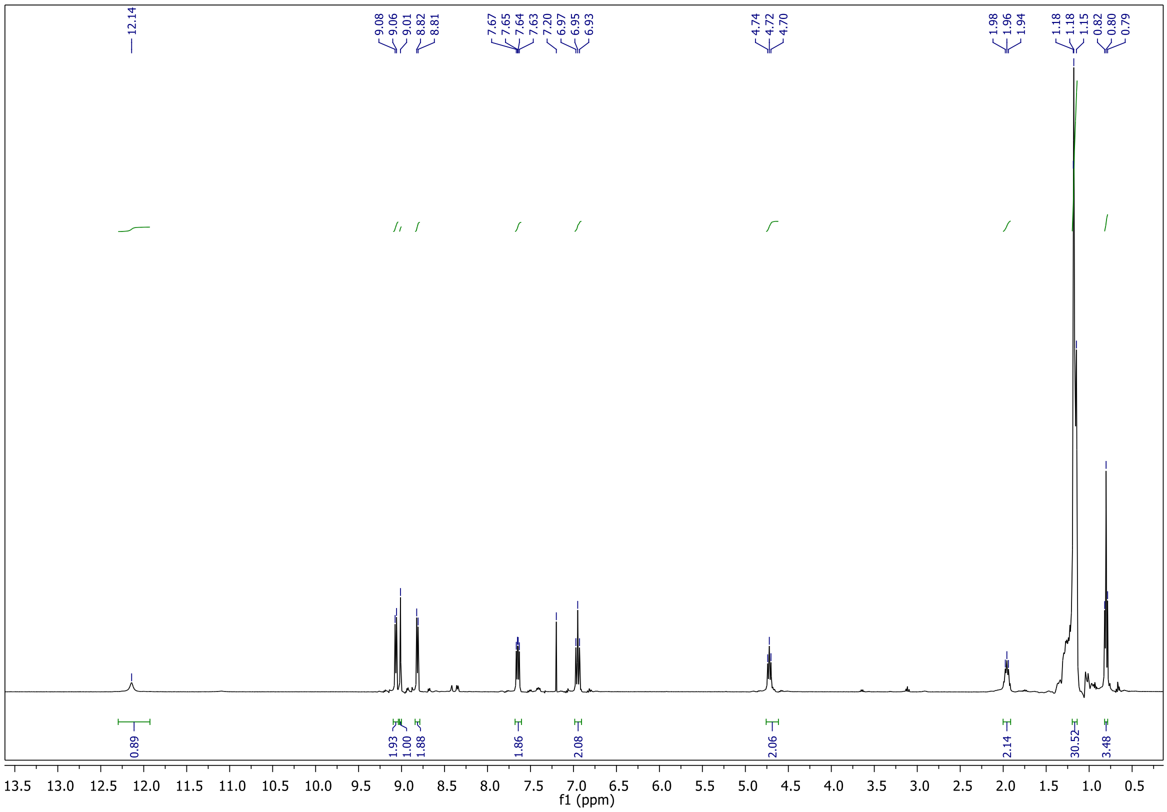

Figure S131. ^{1}HNMR of Compound 31

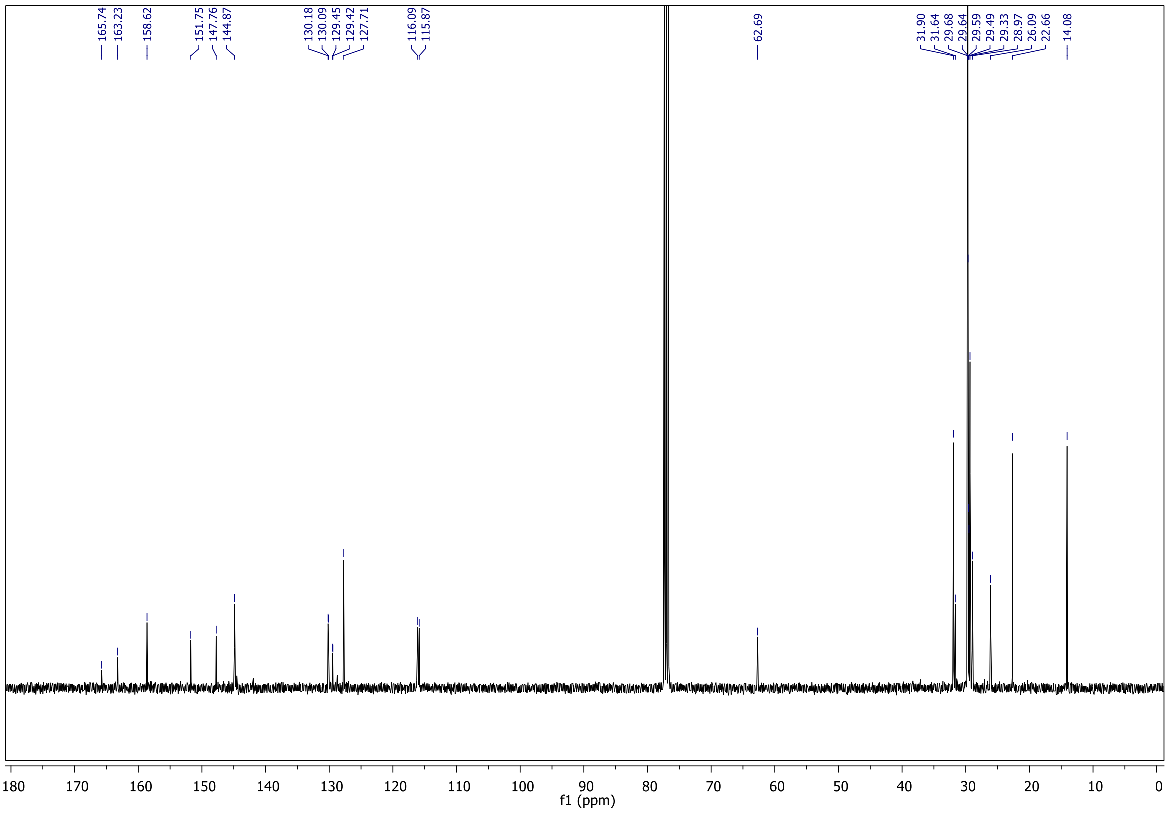

Figure S132. ^{13}CNMR of Compound 31

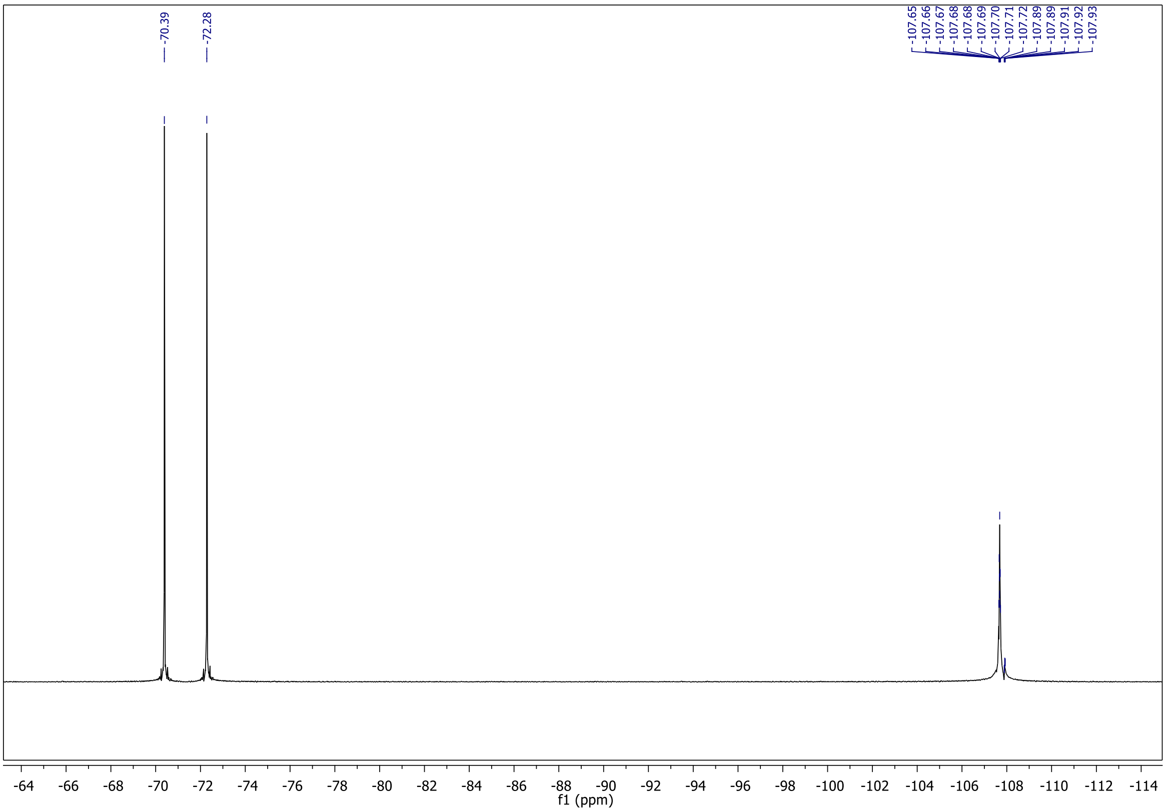

Figure S133. ^{19}FNMR of Compound 31

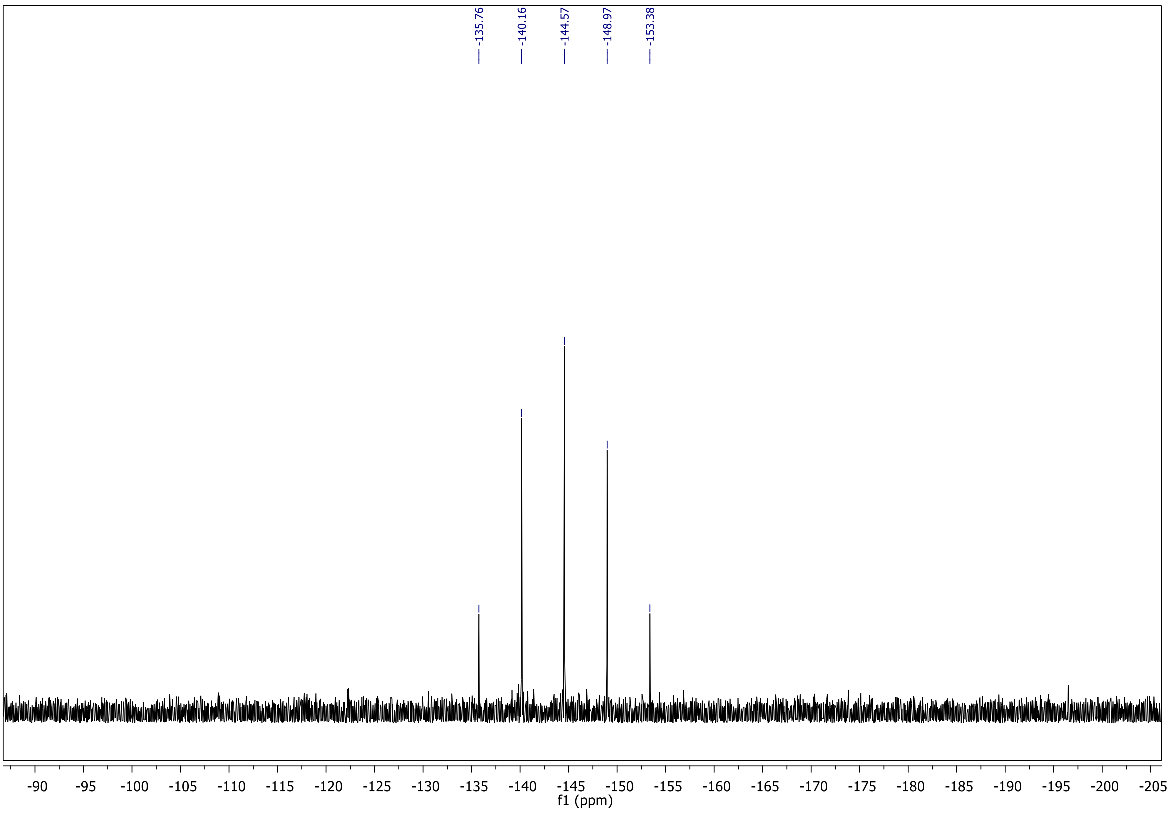

Figure S134. ^{31}PNMR of Compound 31

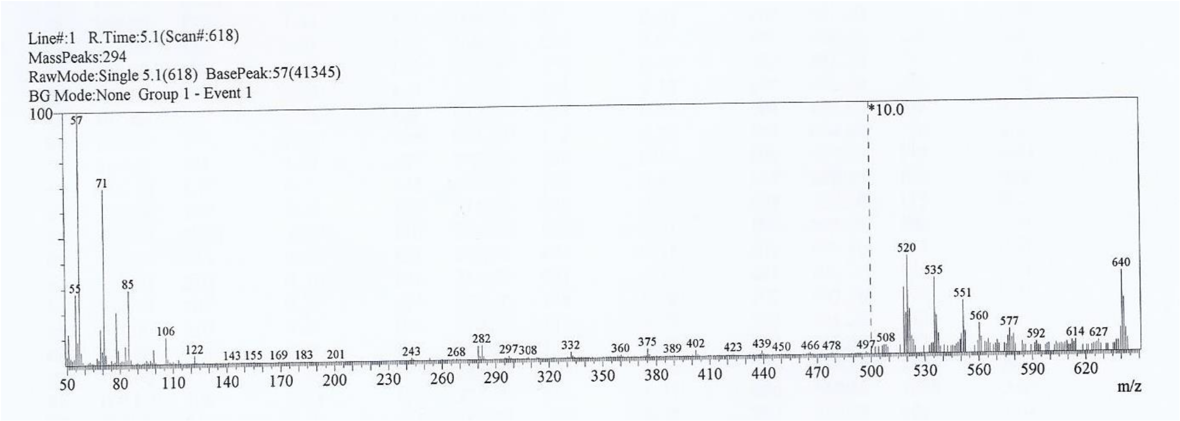

Figure S135. MS (ESI) of Compound 31

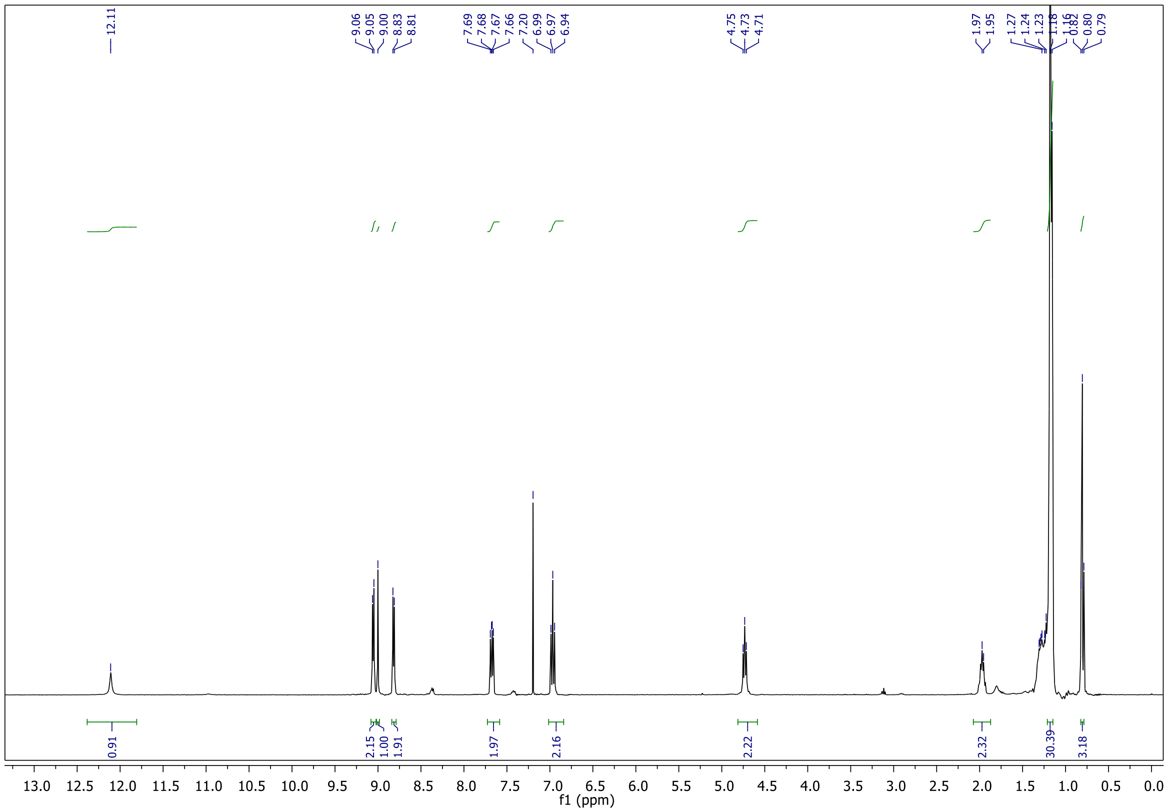

Figure S136. ^{1}HNMR of Compound 32

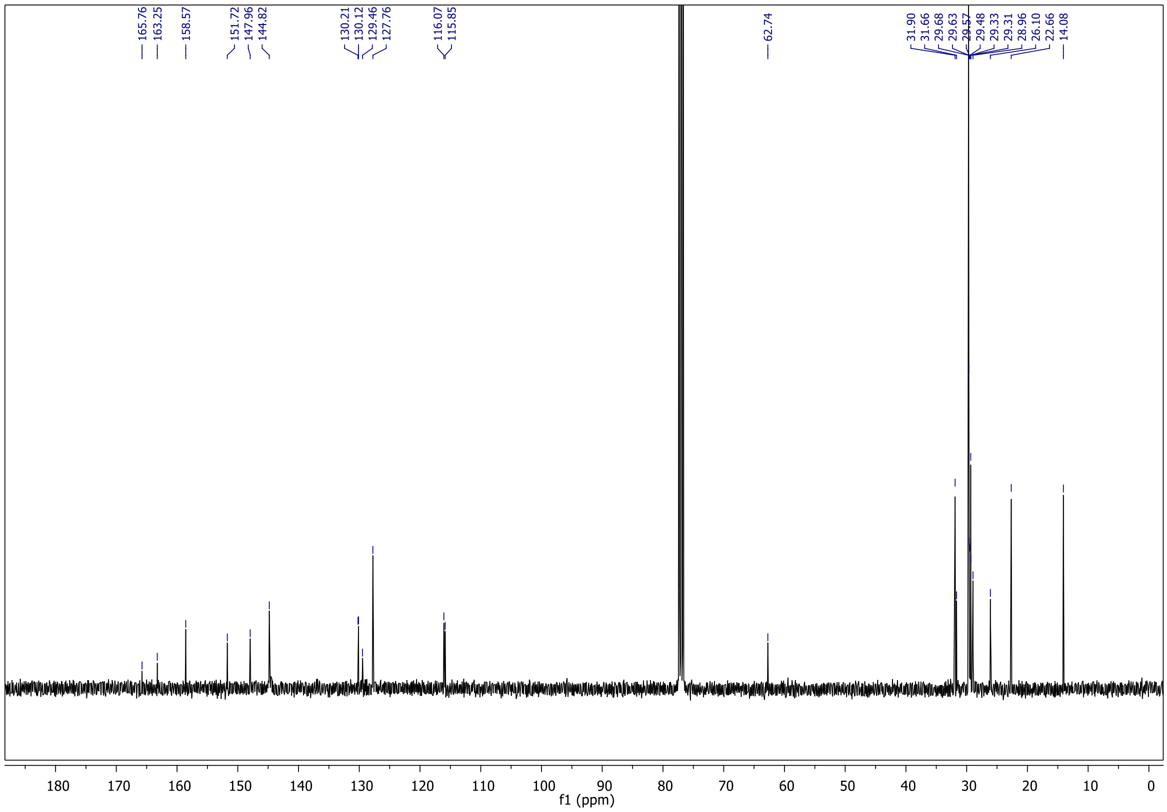

Figure S137. ^13^CNMR of Compound 32

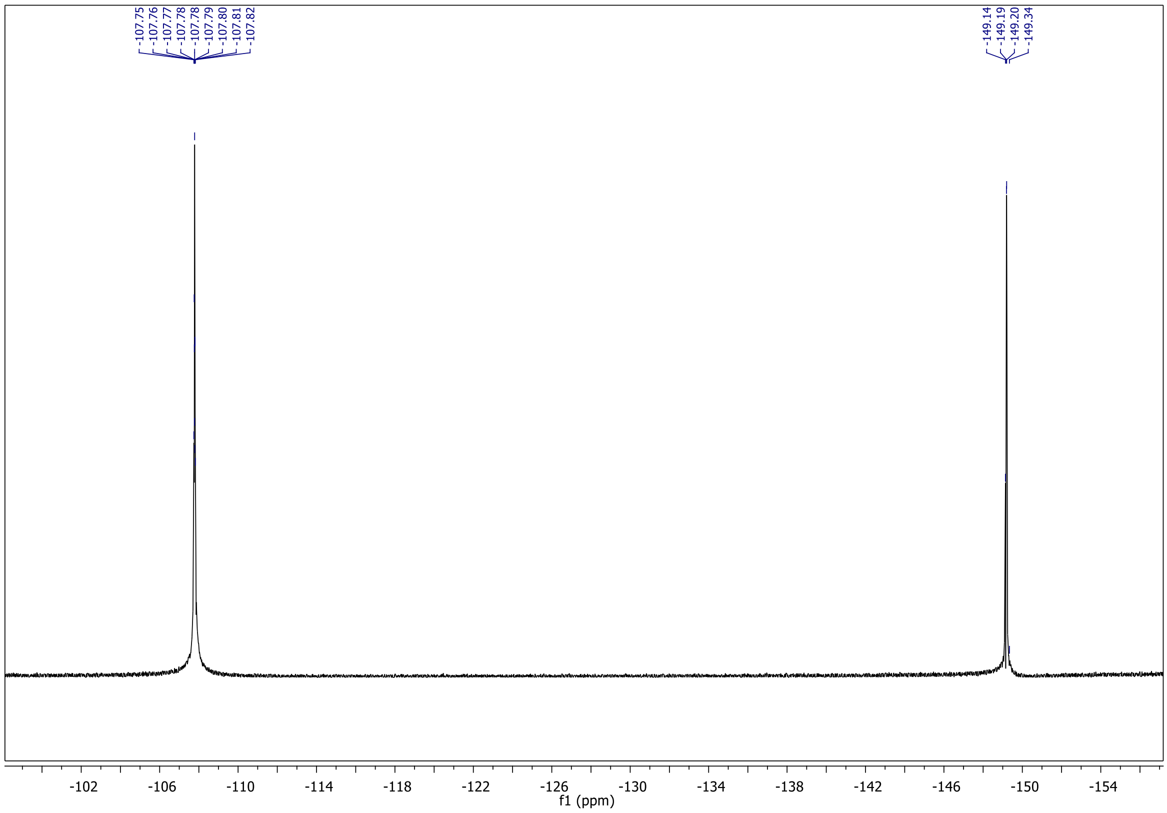

Figure S138. ^19^FNMR of Compound 32

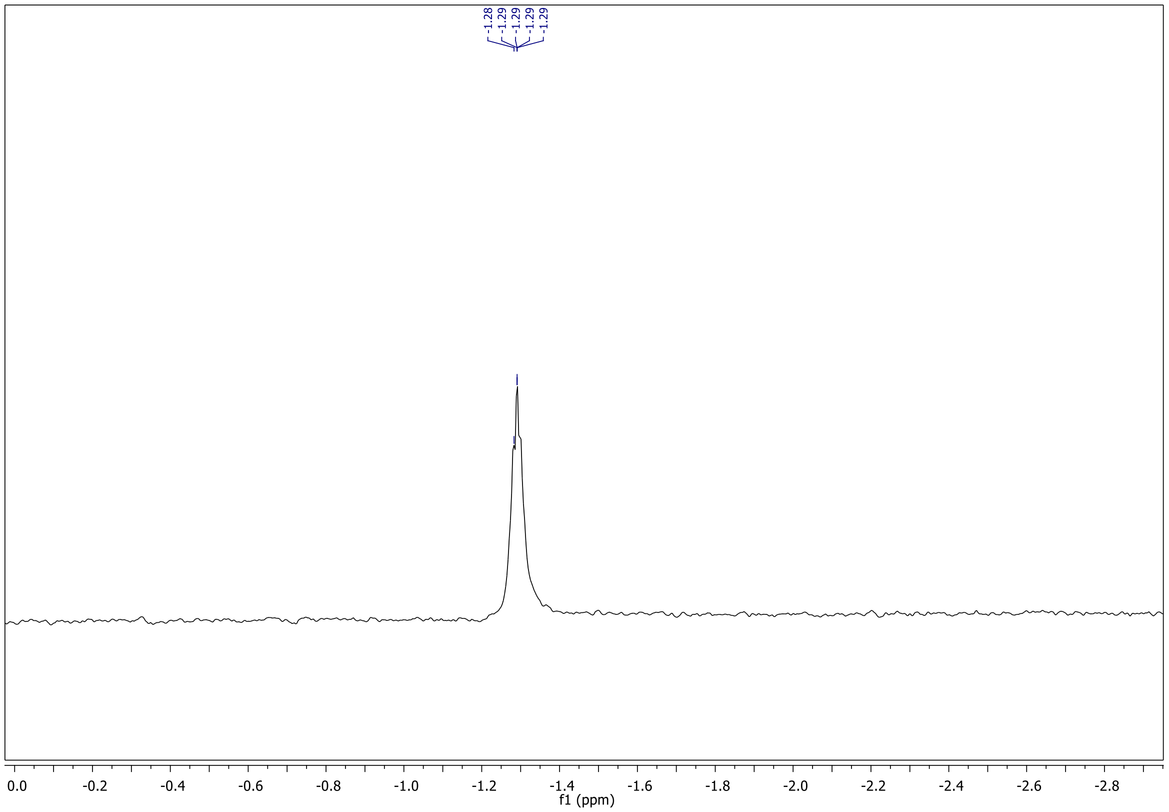

Figure S139. ^{11}BNMR of Compound 32

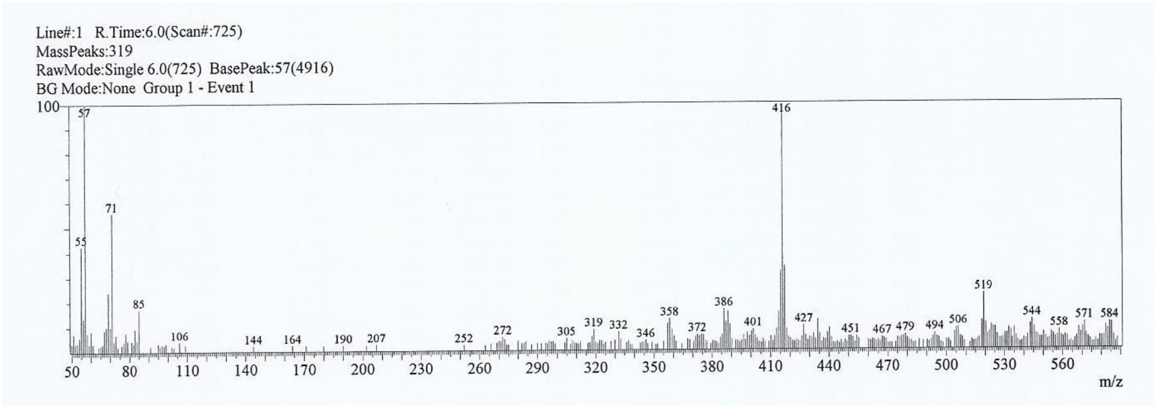

Figure S140. MS (ESI) of Compound 32

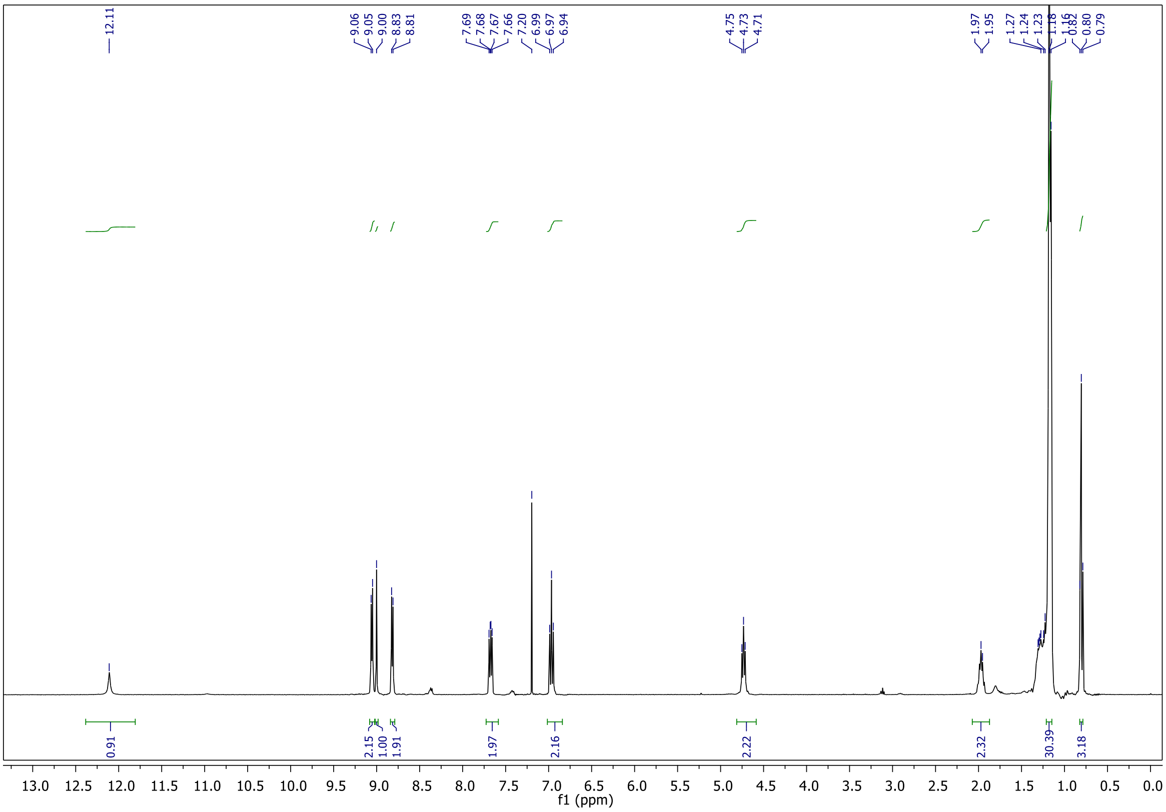

Figure S141. ^{1}HNMR of Compound 33

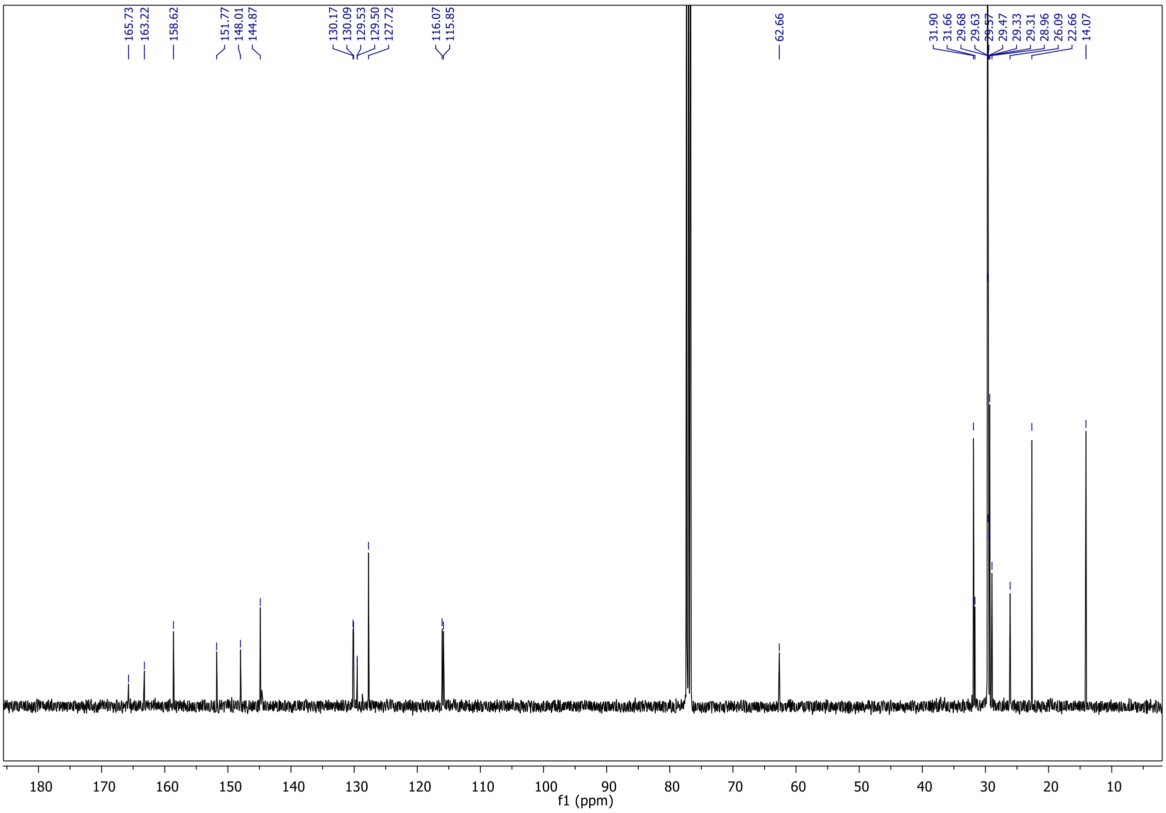

Figure S142. ^{13}CNMR of Compound 33

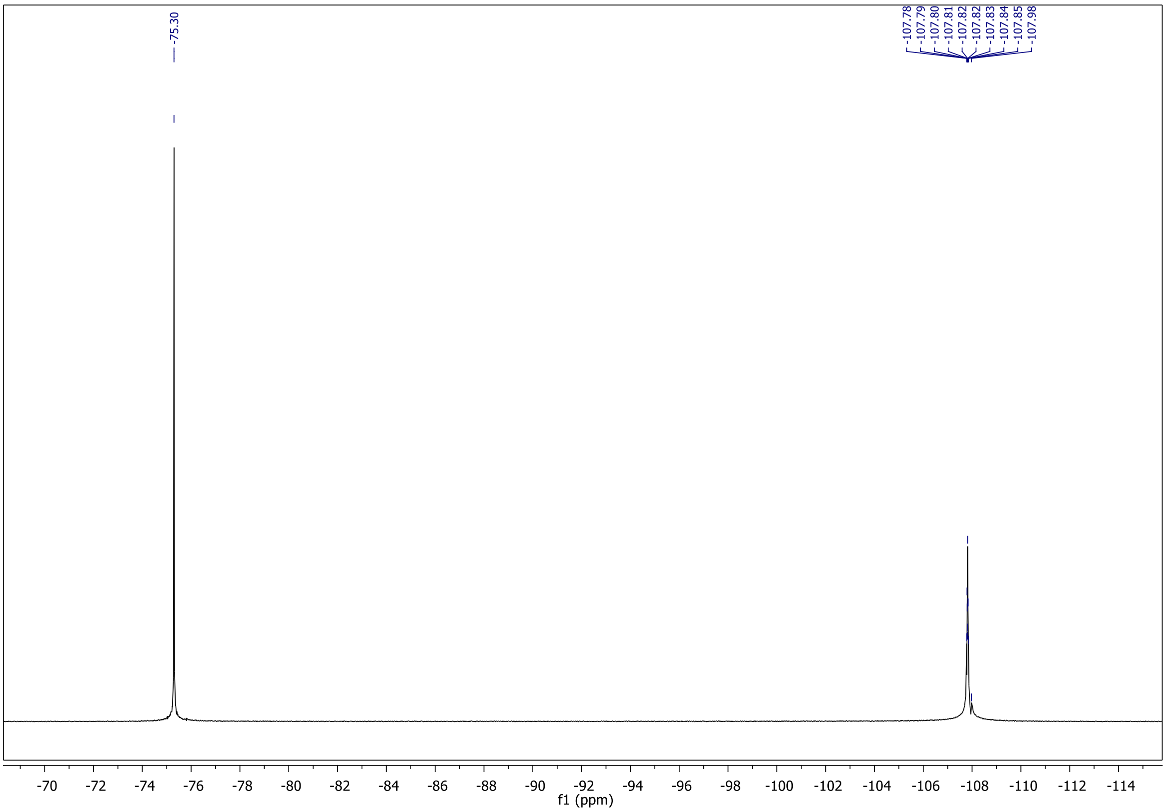

Figure S143. ^{19}FNMR of Compound 33

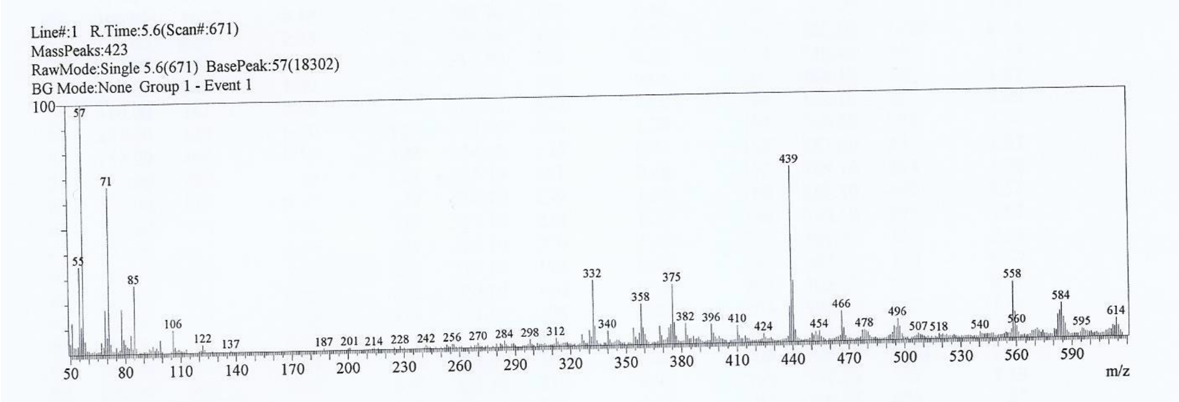

Figure S144. MS (ESI) of Compound 33
